# A Bayesian Network Meta-Analysis for Identifying the Optimal Taxane-Based Chemotherapy Regimens for Treating Gastric Cancer

**DOI:** 10.3389/fphar.2019.00717

**Published:** 2019-07-05

**Authors:** Dan Zhang, Jia-Rui Wu, Xiao-Jiao Duan, Kai-Huan Wang, Yi Zhao, Meng-Wei Ni, Shu-Yu Liu, Xiao-Meng Zhang, Bing Zhang

**Affiliations:** Department of Clinical Chinese Pharmacy, School of Chinese Materia Medica, Beijing University of Chinese Medicine, Beijing, China

**Keywords:** gastric cancer, paclitaxel, docetaxel, chemotherapy, network meta-analysis

## Abstract

**Background:** Several taxane-based chemotherapy regimens are effective in the treatment of gastric cancer; nevertheless, their comparative efficacy and safety remain disputed. This network meta-analysis (NMA) was designed to compare the efficacy and safety of different taxane-based chemotherapy regimens against gastric cancer.

**Methods:** A comprehensive search was conducted to identify all relevant randomized controlled trials (RCTs) in multiple electronic databases. A Bayesian NMA was performed to combine the direct and indirect evidence and estimate the comparative efficacy and safety of different taxane-based chemotherapy regimens simultaneously by utilizing WinBUGS 1.4.3 and Stata 13.1 software. The efficacy outcomes included overall survival rate (OS), progression-free survival (PFS), and overall response rate (ORR), and the safety outcomes were adverse reactions (ADRs), namely, neutropenia, leucopenia, vomiting, and fatigue.

**Results:** A total of 37 RCTs were identified involving 7,178 patients with gastric cancer, and 10 taxane-based chemotherapy regimens (RT, T, TC, TCF, TF, TO, TOF, mTCF, mTF, and mTOF) were collected in gastric cancer therapy. According to the results of cluster analysis, compared with other taxane-based chemotherapy regimens, the regimens of TOF, mTCF, and TF were associated with the most favorable clinical efficacy in improving OS, PFS, and ORR. On the other hand, the regimens of T and mTF had the potential to be the most tolerable and acceptable therapeutic alternative in terms of ADRs.

**Conclusions:** The current NMA provides the evidence that the combination of taxanes (paclitaxel or docetaxel) and fluorouracil is associated with the most preferable and beneficial option for patients with gastric cancer, although additional results from multicenter trials and high-quality studies will be pivotal for supporting our findings.

## Introduction

As one of the most frequently diagnosed cancers, gastric cancer is the second leading cause of cancer mortality worldwide (de Martel et al., 2012; Ferro et al., 2014; Torre et al., 2015). Currently, surgical resection is still the primary curative treatment for gastric cancer. Nevertheless, the majority of patients will suffer from locoregional recurrence; it is a consensus that chemotherapy has been essential for achieving survival advantages and therapeutic benefit (Gunderson, 2002; Cunningham et al., 2006; Miceli et al., 2014). National comprehensive cancer network (NCCN) guidelines recommend that paclitaxel and docetaxel are listed as the standard first-line chemotherapeutic drugs for gastric cancer (Ajani et al., 2016). Paclitaxel and docetaxel are members of drugs called taxanes; taxanes have become key drugs for over a dozen malignancies since their antitumor activity was established in the early 1990s (Kudlowitz and Muggia, 2013). Additionally, paclitaxel, which was originally isolated from the North American Pacific yew tree (*Taxus brevifolia*), was approved by the U.S. Food and Drug Administration as Taxol against advanced ovarian cancer in 1992, and has recently been widely used for the treatment of solid tumors such as gastric cancer (Bocci et al., 2013; Howat et al., 2014; Khanna et al., 2015; Kundranda and Niu, 2015). Similarly, docetaxel has also shown encouraging results in gastric cancer with notable objective responses and meaningful survival advantage (Brower, 2015; Shah et al., 2015). Taxane-based chemotherapy regimens have been validated as promising effective treatments for gastric cancer due to significantly increasing the overall survival compared with placebo and possessing the favorable activity with acceptable adverse toxicities against gastric cancer (Constenla et al., 2002; Wilke et al., 2014; Jiang et al., 2015). There is also increasing concern about the anticancer mechanisms of paclitaxel and docetaxel, and multiple studies have recognized that paclitaxel can arrest mitosis and the cell cycle to induce the death of cells by stabilizing microtubules and interfering with microtubule disassembly during cell division. According to a recent study, the antitumor activity of paclitaxel can be enhanced by exosomes from M1-polarized macrophages through activating macrophage-mediated inflammation (Wang et al., 2019). The encouraging activity of paclitaxel in the treatment of gastric cancer is associated with AKT/ERK activation, the TGF-β/Smad signaling pathway suppression (Tsukada et al., 2013; Wu et al., 2014). Forkhead box transcription factor 1 might be a new therapeutic target in docetaxel-resistant gastric cancer (Li et al., 2013). However, recent evidence has demonstrated that intratumoral concentrations of paclitaxel are too low to cause mitotic arrest and result in multipolar divisions instead (Weaver, 2014; Zhang et al., 2014; Song et al., 2015).

Network meta-analysis (NMA) can simultaneously synthesize direct and indirect comparisons in the absence of direct evidence and also produces inferences regarding the comparative efficacy or safety of multiple treatments and has the potential to rank competing interventions for different outcomes (Salanti et al., 2014; Rücker and Schwarzer, 2015). By virtue of its versatility, NMA is increasingly utilized to address knowledge gaps in medical sciences, especially the field of oncology. Recently, it has been applied to explore adjuvant therapy for pancreatic cancer, the optimal treatment for colorectal cancer, and others (Golfinopoulos et al., 2007; Wang et al., 2012; Liao et al., 2013).

As a cornerstone of chemotherapy for gastric cancer, paclitaxel and docetaxel are used as the standard of care alone or in combination with other anticancer drugs in more than 30 regimens. However, the choice of taxane-based chemotherapy regimens in the initial treatment of gastric cancer is an important issue, and it was still unclear which taxane-based chemotherapy regimens were the most effective and tolerable against gastric cancer. To address these issues, an NMA was designed to summarize the efficacy and safety of different taxane-based chemotherapy regimens, which may aid clinical decision-making.

## Methods

The procedure of the current NMA was conducted in accordance with the Preferred Reporting Items for Systematic reviews and Meta-Analyses (PRISMA) guidelines “NMA extended version” (Hutton et al., 2015). The completed PRISMA checklist was presented as additional file (**Presentation S1**).

### Retrieval Strategies

First, the electronic databases of Embase, PubMed, Cochrane Library, and OVID were searched for all eligible randomized controlled trials (RCTs) from inception to May 29, 2017. There were no limitation for publication years, languages, and blinding methods. For relevant publications, the following terms of gastric cancer were adopted: “Stomach Neoplasms [MeSH Terms],” “Stomach Neoplasm,” “Gastric Neoplasms,” “Gastric Neoplasm,” “Stomach Cancer*,” “Stomach Tumor*,” “Gastric Cancer*,” “Gastric Tumor*,” “Gastric Carcinoma,” and “Stomach Carcinoma.” More specific retrieval strategies were provided in **Presentation S1**. Second, manual searching was supplemented to identify the potential enrolled RCTs from the references of relevant meta-analyses and the retrieved review articles. In addition, the specialists in information retrieval were invited to amend our searching strategies. We appropriately adjusted our retrieval strategies in light of different electronic databases.

### Inclusion and Exclusion Criteria

All the articles were reviewed by two investigators independently. RCTs were included if they satisfied the following criteria: 1) human participants were diagnosed as gastric cancer; 2) taxane-based chemotherapy regimens were used in either arm of the treatment; 3) the presence of a control was treated by the chemotherapeutic drugs in NCCN guideline; 4) the relative efficacy outcomes in the present NMA included OS, progression-free survival (PFS), and overall response rate (ORR), and the safety outcomes were adverse drug reactions (ADRs), such as neutropenia, leucopenia, vomiting, and fatigue; 5) all the trials should be designed as RCTs that compared the relative outcomes of taxane-based chemotherapy regimens.

Two investigators perused the titles and abstracts of the identified RCTs to exclude the irrelevant clinical trials; the exclusion criteria were listed as follows: 1) except for gastric cancer, patients suffered from other cancers; 2) the interventions of trials contained surgery, radiotherapy, or chemotherapeutic drugs that were not recommended by NCCN guidelines; without taxane-based chemotherapy regimens were not in either arm; the arms were different in therapy duration or drug administration; 3) insufficient data were available to estimate the outcomes; 4) type of study was non-RCT, for example, single-arm trial, pharmacological experiments, and reviews; duplications; and unavailable full-text.

### Data Extraction and Quality Assessment

Two investigators screened the initial search results for potentially eligible studies independently. All identified articles were then retrieved in full, and the corresponding data were extracted by Microsoft Excel (Microsoft Corp, Redmond, WA) as follows: 1) the publication information, including the name of first author, publication year, literature databases, and country; 2) the characteristics of the enrolled patients with gastric cancer: number, age, gender, type, and other information of cancer; 3) the information of intervention: the dosage, duration, and treatment cycle; 4) outcomes: the measured data about the efficacy and safety outcomes. The Kaplan–Meier curves of OS and PFS were digitized using Engauge Digitizer (www.digitizer.sourceforge.net). These outcomes were calculated by the following formula: ORR = (number of complete response patients + partial response)/the total number of patients × 100%; the incidence of ADRs = (number of patients occurred ADRs/total number of patients) × 100%; 4) the description of study design: blinding, randomization allocation methods, and other items for quality assessment. For analysis purposes, taxane-based chemotherapy regimens were considered as the experimental arm, and other chemotherapy treatments were considered to be the control arm. Similarly, docetaxel and paclitaxel were merged to the taxanes (T) drug class, and other chemotherapeutic drugs were defined as their initials in the NMA.

The two investigators independently examined the quality of all included trials according to the Cochrane risk of bias tool (Cochrane Handbook for Systematic Reviews of Interventions, version 5.1.0) (Higgins et al., 2011). Discrepancies were resolved either by consensus or through adjudication by a third investigator. The quality evaluation items of each trial included selection bias (random sequence generation and allocation concealment), performance bias (blinding of participants and personnel), detection bias (blinding of outcome assessment), attrition bias (incomplete outcome data), reporting bias (selective reporting), and other biases, and these items were scored as low, high, or unclear risk of bias.

This present NMA does not require ethical approval because it only gathered the data from relevant published trials.

### Statistical Analysis

Odds ratios (ORs) were calculated for dichotomous data with corresponding 95% credible intervals (CIs). On the one hand, a Bayesian NMA was designed to obtain estimates for the comparative efficacy and safety of taxane-based chemotherapy regimens against gastric cancer. WinBUGS 1.4.3 software (MRC Biostatistics Unit, Cambridge, UK) was utilized to perform statistical analysis. The posterior densities were estimated through the Markov Chain Monte Carlo simulations in the random-effects model (Achana et al., 2014; Stephenson et al., 2015; Greco et al., 2016). The choice of random-effects model for outcomes was mainly associated with the within-study and between-study methodological and clinical variation in current NMA (Jackson et al., 2014; Chan, 2016). The results of analysis procedure were based on 200,000 simulation iterations and 10,000 adaptation iterations. On the other hand, Stata version 13.1 software (Stata Corp, College Station, TX) was adopted to present the results and graphs from the NMA (Shim et al., 2017). The network graph could display the relationship of observed comparisons. The thickness of the lines in the network graph was proportional to the number of trials used for comparisons; node sizes corresponded to total sample sizes for treatments (Chaimani et al., 2013; Donegan et al., 2013). Moreover, the Surface Under the Cumulative Ranking (SUCRA) curve was employed to rank the different taxane-based chemotherapy regimens towards each outcome. The value of SUCRA ranged from 0% to 100%, and the larger the SUCRA value of comparisons was regarded as the better treatment option (Rücker and Schwarzer, 2015; Trinquart et al., 2016). In terms of the publication bias, SUCRA values were graphically accessed *via* a comparison-adjusted funnel plot, and Egger’s regression test and Begg regression test were applied to measure the asymmetry; the results of Egger test (*P* > .05) and Begg test (*P* > .05) were defined as non-significant publication bias among included RCTs (Trinquart et al., 2012). Besides, the inconsistency between indirect and direct comparisons was calculated with the inconsistency factors (IFs) and their 95% CIs in node-splitting analysis for each loop of evidence, and it was regarded as a better consistency when the lower bound of 95% CIs was equal to zero (Hans-Peter, 2014; Krahn et al., 2014; Mavridis et al., 2014). Additionally, the cluster analysis was conducted for choosing the optimal taxane-based chemotherapy regimens in consideration of two different outcomes simultaneously, and the interventions located in the upper right corner were superior to others (Veroniki et al., 2015).

## Results

### Literature Search and Study Characteristics

**Figure 1:**
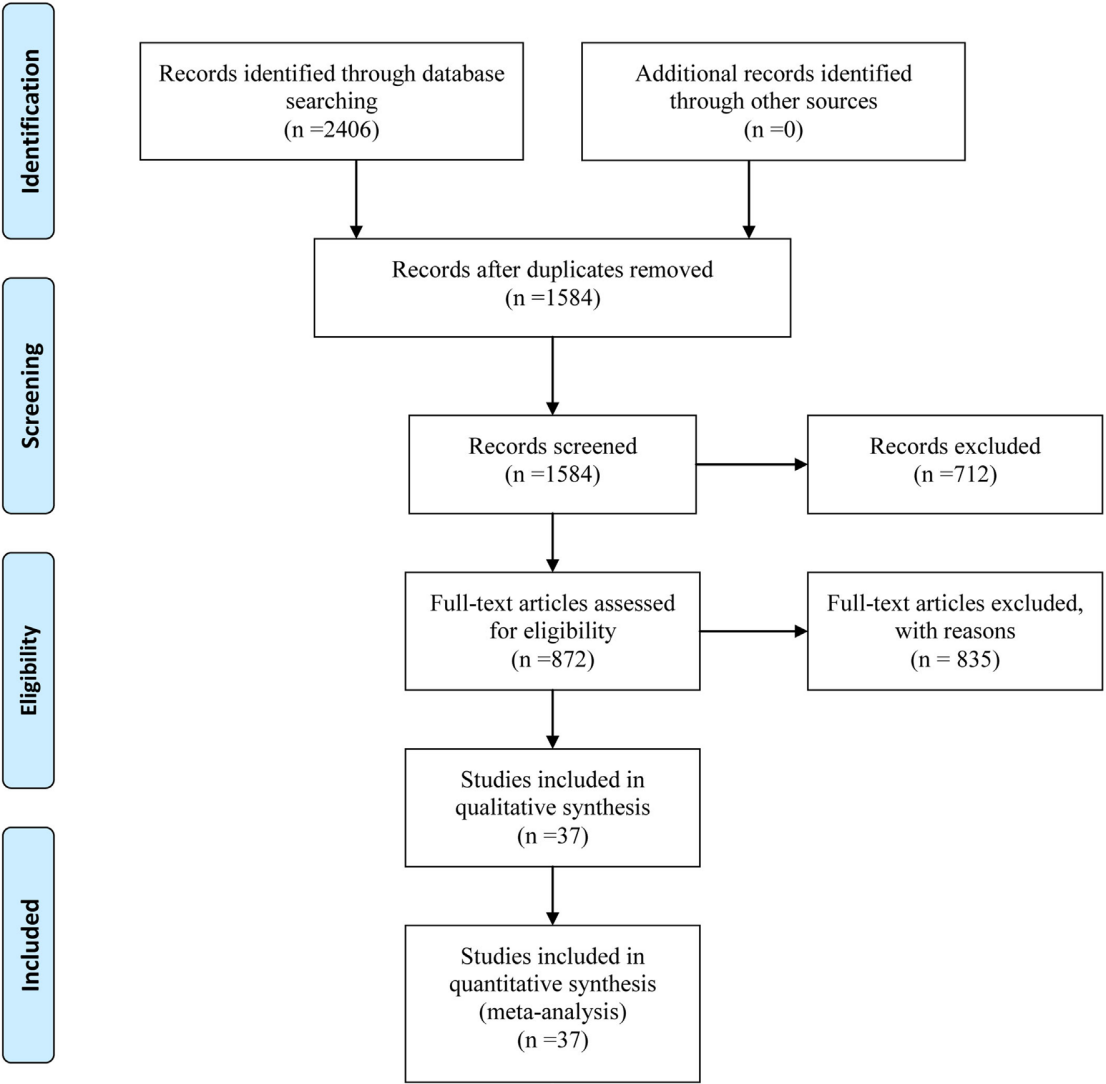
Flow chart of the search for eligible studies.

Initially, a total of 2406 citations were yielded through comprehensive searching according to the searching strategy as mentioned. After screening the titles and abstracts, we excluded the irrelevant and duplicate articles; 872 potentially eligible papers were selected for full-text reading. Ultimately, we included 37 RCTs, which were subject to data extraction and analysis. All the 37 eligible studies were published between 1999 and 2016. In addition, this NMA incorporated 10 taxane-based chemotherapy regimens (RT, T, TC, TCF, TF, TO, TOF, mTCF, mTF, and mTOF). The process of the study selection is shown in **Figure 1**. The references and reasons for excluding articles from full-text assessment are listed in **Presentation S1**.

**Figure 2:**
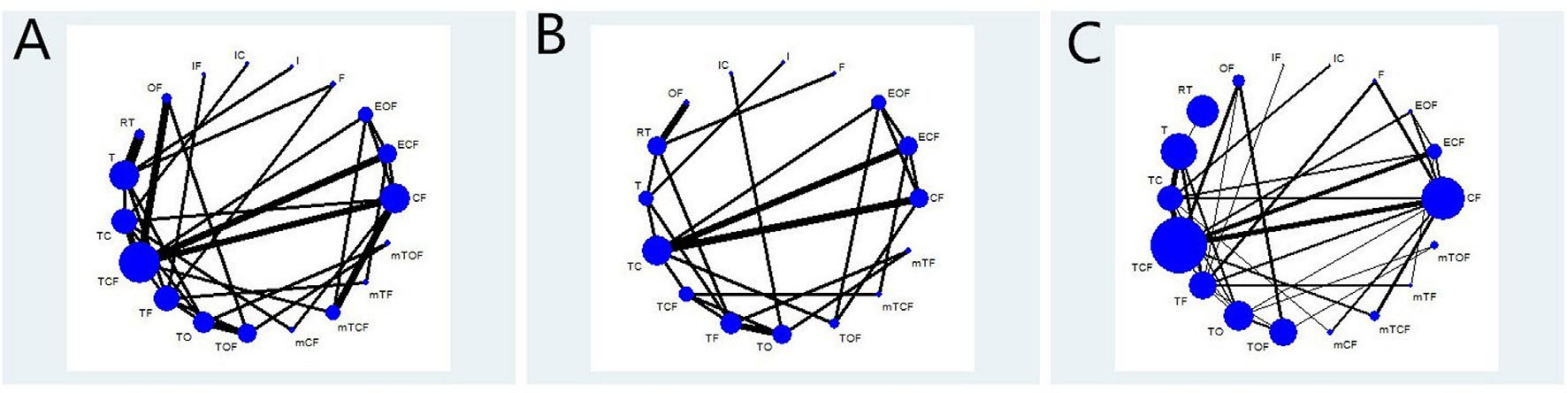
Network graph of the efficacy outcomes. Node sizes indicate total sample sizes for treatments. Line thicknesses correspond to the number of trials used for comparisons. **(A)** OS; **(B)** PFS; **(C)** ORR.

**Table 1** summarizes the baseline characteristics of RCTs included in the NMA from 13 different countries. Overall, 7,178 patients with gastric cancer from 37 RCTs were involved, and the number of participants in the trials varied from 24 to 714 and their ages ranged from 19 to 87 years old. The network plots of evidence with respect to efficacy outcome are illustrated in **Figure 2**.

**Table 1:** Characteristics of included RCTs.

Study ID	Country	Size	M/F	Age (median/range)	Regimen	Intervention	Duration	Outcome
Ajani et al., 2005	USA	76/79	114/41	57/21–83	TC/TCF	TC (docetaxel 85 mg/m^2^ + cisplatin 75 mg/m^2^); TCF (docetaxel 85 mg/m^2^ + cisplatin 75 mg/m^2^ + 5-FU 750 mg/m^2^)	3 w	OS, PFS, ORR, ADRs
Al-Batran et al., 2013	Germany	71/72	96/47	69.5	OF/TOF	OF (oxaliplatin 85 mg/m^2^ + leucovorin 200 mg/m^2^ + 5-FU 2600 mg/m^2^); TOF (oxaliplatin 85 mg/m^2^ + leucovorin 200 mg/m^2^ + docetaxel 50 mg/m^2^ + 5-FU 2600 mg/m^2^)	3 w	OS, PFS, ORR, ADRs
Casak et al., 2015	USA	330/335	/	/	RT/T	RT (paclitaxel 80 mg/m^2^ + ramucirumab 8 mg/kg); T (paclitaxel 80 mg/m^2^)	4 w	OS
Gómez-Martin et al., 2012	Spain	41/32/27/58	112/46	61/20–79	CF/ECF/EOF/TCF	CF (cisplatin 80 mg/m^2^/day + capecitabine); ECF (epirubicin 50 mg/m^2^ + cisplatin 60 mg/m^2^ + capecitabine); EOF (epirubicin 50 mg/m^2^ + oxaliplatin 130 mg/m^2^ + capecitabine); TCF (docetaxel 60 mg/m^2^/day + cisplatin 60 mg/m^2^ + capecitabine)	3 w	OS, PFS, ORR, ADRs
Gubanski et al., 2010	Sweden	39/39	60/18	63.5/39–79	IF/TF	IF (docetaxel 45 mg/m^2^ + irinotecan 180 mg/m^2^); TF (docetaxel 45 mg/m^2^ + 5-FU 750 mg/m^2^)	2 w	OS, ORR, ADRs
Guo et al., 2015	China	174/96/127	286/111	/	TF/TO/TOF	TF (paclitaxel 135 mg/m^2^ + 5-FU 2400 mg/m^2^ + leucovorin 400 mg/m^2^); TO (paclitaxel 135 mg/m^2^ + oxaliplatin 85 mg/m^2^ + leucovorin 400 mg/m^2^)/TOF (paclitaxel 135 mg/m^2^ + oxaliplatin 85 mg/m^2^ + 5-FU 2400 mg/m^2^ + leucovorin 400 mg/m^2^)	3 w	OS, PFS, ORR, ADRs
Hironaka et al., 2013	Japan	108/111	171/48	65/37–75	T/I	T (paclitaxel 80 mg/m^2^); I (irinotecan 150 mg/m^2^)	4 w	OS, PFS, ADRs
Inal et al., 2012	Turkey	85/22	69/38	54/23–76	TCF/mTCF	TCF (docetaxel 75 mg/m^2^ + cisplatin + 5-FU 750 mg/m^2^); mTCF (docetaxel 60 mg/m^2^ + cisplatin + 5-FU 600 mg/m^2^)	3 w	OS, PFS, ORR, ADRs
Kim et al., 2015	Korea	27/25	42/10	/	T/TO	T (docetaxel 36 mg/m^2^); TO (docetaxel 36 mg/m^2^ + oxaliplatin 80 mg/m^2^)	3 w	OS, PFS, ORR, ADRs
Kim et al., 2014	Korea	38/39	54/22	57/35–75	TC/TO	TC (docetaxel 35 mg/m^2^ + cisplatin 60 mg/m^2^); TO (docetaxel 35 mg/m^2^ + oxaliplatin 120 mg/m^2^)	3 w	OS, PFS, ORR, ADRs
Kos et al., 2011	Turkey	30/40	48/22	53.5/23–69	CF/mTCF	CF (cisplatin 50 mg/m^2^ + leucovorin 200 mg/m^2^ + 5-FU 400 mg/m^2^); mTCF (docetaxel 60 mg/m^2^ + cisplatin 60 mg/m^2^ + 5-FU l600 mg/m^2^)	3 w	OS, PFS, ORR, ADRs
Li et al., 2009	China	52/52	63/41	/	TCF/OF	TCF (docetaxel 75 mg/m^2^ + oxaliplatin 15 mg/m^2^ + 5-FU 500 mg/m^2^); OF (oxaliplatin 85 mg/m^2^ + leucovorin 200 mg/m^2^ + 5-FU 400 mg/m^2^)	3 w	OS, ORR, ADRs
Li et al., 2011	China	50/44	63/31	58.5/20–75	TCF/OF	TCF (paclitaxel 50 mg/m^2^ + cisplatin 20 mg/m^2^ + 5-FU 750 mg/m^2^); OF (oxaliplatin 85 mg/m^2^ + leucovorin 200 mg/m^2^ + 5-FU 400 mg/m^2^)	4 w	OS, ORR, ADRs
Lim et al., 2010	Korea	77/72/37	116/70	57	mCF/TC/CF	mCF (cisplatin 60–100 mg/m^2^ + capecitabine 1000 mg/m^2^); TC (docetaxel 75 mg/m^2^ + cisplatin 60–100 mg/m^2^); CF (cisplatin 60–100 mg/m^2^ + 5-FU 800–1000 mg/m^2^)	3 w	OS, ORR, ADRs
Liu et al., 2015	China	57/63	59/61	58.9/46–75	TC/TCF	TC (docetaxel 65–75 mg/m^2^ + cisplatin 15–20 mg/m^2^); TCF (docetaxel 65–75 mg/m^2^ + cisplatin 15–20 mg/m^2^ + capecitabine 1000 mg/m^2^)	3 w	OS, ORR, ADRs
Lu et al., 2016	China	34/30	38/26	64/38–77	TCF/TF	TCF (paclitaxel 135 mg/m^2^ + capecitabine 2000 mg/m^2^ + 5-FU 350 mg/m^2^); TF (paclitaxel 135 mg/m^2^ + 5-FU 350 mg/m^2^)	3 w	OS, PFS, ORR, ADRs
Maruta et al., 2007	Japan	12/12	18/6	62	T/TF	T (docetaxel 60 mg/m^2^); TF (docetaxel 60 mg/m^2^ + 5-FU 600 mg)	3 w	OS, ORR, ADRs
Muro et al., 2016	Argentina	330/335	472/190	61/24–84	RT/T	RT (ramucirumab 8 mg/kg + paclitaxel 80 mg/m^2^); T (paclitaxel 80 mg/m^2^)	3 w	OS, PFS, ORR, ADRs
Nishina et al., 2016	Japan	49/51	33/16 36/15	59/30–74 64/39–75	F/T	F (5-FU 800 mg/m^2^); T (paclitaxel 80 mg/m^2^)	4 w	OS, PFS, ADRs
Ochenduszko et al., 2015	Poland	29/27	29/27	59	EOF/mDCF	EOF (epirubicin 50 mg/m^2^ + oxaliplatin 130 mg/m^2^ + capecitabine 625 mg/m^2^); mDCF (docetaxel 40 mg/m^2^ + leucovorin 400 mg/m^2^ + 5-FU 400 mg/m^2^ + cisplatin 40 mg/m^2^)	3 w	OS, PFS, ADRs
Roth and Ajani, 2003	Switzerland	76/79	117/164	54.5	TC/TCF	TC (docetaxel 85 mg/m^2^ + cisplatin 75 mg/m^2^); TCF (docetaxel 75 mg/m^2^ + cisplatin 75 mg/m^2^ + 5-FU 750 mg/m^2^)	3 w	ORR, ADRs
Roth et al., 2007	Switzerland	40/38/41	89/30	59/32–78	ECF/TC/TCF	ECF (epirubicin 50 mg/m^2^ + cisplatin 60 mg/m^2^ + 5-FU 200 mg/m^2^); TC (docetaxel 85 mg/m^2^ + cisplatin 75 mg/m^2^); TCF (docetaxel 85 mg/m^2^ + cisplatin 75 mg/m^2^ + 5-FU 300 mg/m^2^)	3 w	ORR, ADRs
Teker et al., 2014	Turkey	44/42	52/34	56/25–77	TCF/ECF	TCF (docetaxel 50–75 mg/m^2^ + cisplatin 50–75 mg/m^2^ + 5-FU 500–750 mg/m^2^); ECF (epirubicin 50 mg/m^2^ + cisplatin 60 mg/m^2^ + 5-FU 200 mg/m^2^)	3 w	OS, PFS, ORR, ADRs
Thuss-Patience et al., 2005	Germany	45/45	65/25	62/34–75	mTF/ECF	mTF (docetaxel 75 mg/m^2^ + 5-FU 200 mg/m^2^); ECF (epirubicin 50 mg/m^2^ + cisplatin 60 mg/m^2^ + 5-FU 200 mg/m^2^)	3 w	OS, PFS, ORR, ADRs
Thuss-Patience et al., 2011	Germany	40/51	68/23	62/32–79	mTF/TF	mTF (docetaxel 60 mg/m^2^ + capecitabine 800 mg/m^2^); TF (docetaxel 75 mg/m^2^ + capecitabine 1000 mg/m^2^)	3 w	OS, PFS, ORR, ADRs
Tsuburaya et al., 2014	Japan	359/355	486/228	/	F/TF	F (5-FU 267 mg/m^2^); TF (paclitaxel 80 mg/m^2^ + 5-FU 267 mg/m^2^)	3 w	OS, ADRs
Van Cutsem et al., 2006	Russia	221/224	317/128	55/25–79	TCF/CF	TCF (docetaxel 75 mg/m^2^ + cisplatin 75 mg/m^2^ + 5-FU 750 mg/m^2^); CF (cisplatin 100 mg/m^2^ + 5-FU 1,000 mg/m^2^)	4 w	OS, PFS, ORR, ADRs
Van Cutsem et al., 2015	Belgium	79/89/86	175/79	59	TO/TOF/mTOF	TO (docetaxel 75 mg/m^2^ + oxaliplatin 130 mg/m^2^); TOF (docetaxel 50 mg/m^2^ + oxaliplatin 85 mg/m^2^ + 5-FU 2400 mg/m^2^ + leucovorin 400 mg/m^2^); mTOF (docetaxel 50 mg/m^2^ + oxaliplatin 100 mg/m^2^ + capecitabine 625 mg/m^2^)	3 w	OS, PFS, ORR, ADRs
Wang et al., 2016	China	119/115	169/65	56.1/19–80	CF/mTCF	CF (cisplatin 75 mg/m^2^ + 5-FU 600 mg/m^2^); mTCF (docetaxel 60 mg/m^2^ + cisplatin 60 mg/m^2^ + 5-FU 600 mg/m^2^)	3 w	OS, ORR, ADRs
Wilke et al., 2014	Germany	330/335	472/193	61/25–84	RT/T	RT (ramucirumab 8 mg/kg + paclitaxel 80 mg/m^2^); T (paclitaxel 80 mg/m^2^)	4 w	OS, PFS, ORR, ADRs
Yang et al., 2005	China	60/60/60	118/62	62/22–87	CF/TCF/TO	CF (cisplatin 50 mg/m^2^ + 5-FU 200 mg/m^2^); TCF (Taxol 100 mg/m^2^ + cisplatin 50 mg/m^2^ + 5-FU 200 mg/m^2^); TO (Taxol 100 mg/m^2^ + oxaliplatin 100 mg/m^2^)	3 w	ORR, ADRs
Ye et al., 2008	China	60/72	86/46	51	OF/TF	OF (oxaliplatin 85 mg/m^2^ + leucovorin 200 mg/m^2^ + 5-FU 400 mg/m^2^); TF (paclitaxel 75 mg/m^2^ + leucovorin 200 mg/m^2^ + 5-FU 400 mg/m^2^)	4 w	ORR, ADRs
Zhang et al., 2009	China	37/30/35	71/31	/	F/TF/CF	F (capecitabine 1000 mg/m^2^); TF (paclitaxel 175 mg/m^2^ + capecitabine 1000 mg/m^2^); CF (cisplatin 15-20 mg/m^2^ + capecitabine 1000 mg/m^2^)	3 w	ORR, ADRs
Zhao et al., 2016	China	78/78	80/76	39.40	CF/TCF	CF (cisplatin 25 mg/m^2^ + 5-FU 400 mg/m^2^); TCF (paclitaxel 80 mg/m^2^ + cisplatin 25 mg/m^2^ + 5-FU 400 mg/m^2^)	3 w	ORR, ADRs
Zhou et al., 2015	China	40/40	51/29	35–68	OF/TOF	OF (oxaliplatin 130 mg/m^2^ + leucovorin 200 mg/m^2^ + 5-FU 400 mg/m^2^); TOF (docetaxel 75 mg/m^2^ + oxaliplatin 130 mg/m^2^ + leucovorin 200 mg/m^2^ + 5-FU 400 mg/m^2^)	3 w	ORR, ADRs
Zhu et al., 2016	China	51/43	58/36	55/31–73	TC/IC	TC (docetaxel 35 mg/m^2^ + cisplatin 30 mg/m^2^); IC (irinotecan 65 mg/m^2^ + cisplatin 30 mg/m^2^)	3 w	OS, PFS, ORR, ADRs
Lee et al., 2017	Korea	23/24	38/8	55/34–74	T/TC	T (docetaxel 75 mg/m^2^); TC (docetaxel 60 mg/m^2^ + cisplatin 60 mg/m^2^)	3 w	OS, PFS, ORR, ADRs

### Assessment of Methodological Quality

**Figure 3:**
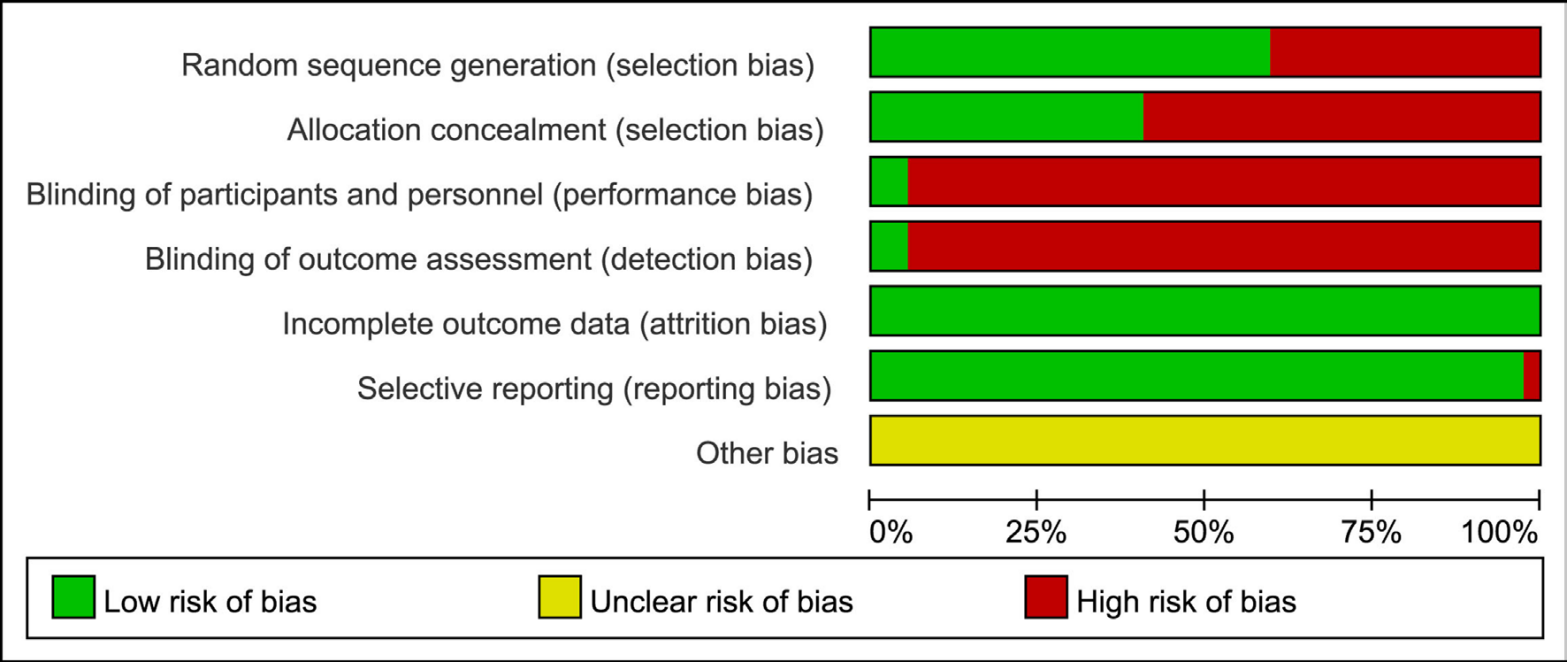
Risk of bias graph.

We critically appraised the methodological quality of the included RCTs in accordance with the Cochrane risk of bias tool. In random sequence generation, a total of 21 RCTs (56.76%) were rated as low risk in randomization owing to the fact that authors stated the principles of randomization in detail, and the remaining 16 trials were defined as high risk. Only 14 trials (37.84%) provided information on allocation concealment, and thus they were regarded as low risk. Among included RCTs, the appropriate blinding procedure was introduced in 2 RCTs (5.41%); therefore, they were evaluated as low risk in performance bias and detection bias. Since all the trials included in the NMA disclosed the specific information about withdrawals, the attrition bias was minimized. In terms of selective reporting, only 1 RCT (2.70%) explicitly had a reporting bias. Other bias sources were not identified. A summary of the risk of bias for each included RCT is shown in **Figure 3**.

### The Efficacy Outcomes

The 1-year OS data were available for 30 RCTs involving 19 chemotherapy regimens (CF, ECF, EOF, F, I, IC, IF, OF, RT, T, TC, TCF, TF, TO, TOF, mCF, mTCF, mTF, and mTOF). According to the results of NMA illustrated in **Table 2**, there were 11 comparisons with statistically significant differences with respect to 1-year OS, namely, TO vs. mTCF (OR = 3.04, 95% CI = 1.13–7.75), T vs. TF (OR = 2.72, 95% CI = 1.19–6.33), TC vs. TF (OR = 2.79, 95% CI = 1.26–5.87), I vs. TF (OR = 3.88, 95% CI = 1.27–12.98), TO vs. TOF (OR = 2.90, 95% CI = 1.63–5.04), TF vs. TO (OR = 0.25, 95% CI = 0.14–0.48), TF vs. mTOF (OR = 0.28, 95% CI = 0.11–0.77), TCF vs. TO (OR = 0.40, 95% CI = 0.20–0.86), TOF vs. mTOF (OR = 0.39, 95% CI = 0.17–0.89), RT vs. T (OR = 0.61, 95% CI = 0.41–0.91), and OF vs. TO (OR = 0.41, 95% CI = 0.19–0.93). Based on the SUCRA in **Figure S1**, the TF regimen had the greatest possibility of achieving a considerable improvement in 1-year OS. In addition, the taxane-based chemotherapy regimens were ranked as follows: TF (92.15%) > mTCF (78.88%) > TOF (75.49%) > TCF (68.06%) > mTF (66.67%) > RT (61.47%) > TC (34.35%) > T (32.13%) > mTOF (24.22%) > TO (16.31%).

**Table 2:** The NMA result of comparisons with significant difference.

Outcome	Comparison	OR (95% CI)	Outcome	Comparison	OR (95% CI)	Outcome	Comparison	OR (95% CI)
OS	TO vs. mTCF	3.04 (1.13,7.75)	ORR	F vs. IC	7.83 (1.34,78.44)	Neutropenia	RT vs. mTCF	0.043 (0.0034,0.51)
OS	T vs. TF	2.72 (1.19,6.33)	ORR	F vs. mCF	7.08 (1.38,62.19)	Neutropenia	RT vs. T	0.19 (0.063,0.53)
OS	TC vs. TF	2.79 (1.26,5.87)	ORR	CF vs. TO	2.57 (1.43,4.59)	Neutropenia	TOF vs. mTCF	0.067 (0.0064,0.62)
OS	I vs. TF	3.88 (1.27,12.98)	ORR	F vs. TC	6.49 (1.44,57.02)	Neutropenia	TCF vs. mTCF	0.18 (0.041,0.77)
OS	TO vs.TOF	2.90 (1.63,5.04)	ORR	F vs. TCF	6.44 (1.46,54.55)	Neutropenia	TC vs. mTCF	0.16 (0.030,0.87)
OS	TF vs. TO	0.25 (0.14,0.48)	ORR	CF vs. TF	2.63 (1.57,4.42)	Leukopenia	CF vs. EOF	40.09 (1.01,17)
OS	TF vs. mTOF	0.28 (0.11,0.77)	ORR	F vs. TO	9.00 (1.96,80.7)	Leukopenia	IF vs. mTCF	31.71 (1.06,1145)
OS	TCF vs. TO	0.40 (0.20,0.86)	ORR	F vs. TF	9.32 (2.02,81.72)	Leukopenia	ECF vs. mTCF	35.27 (1.73,1004)
OS	TOF vs. mTOF	0.39 (0.17,0.89)	ORR	EOF vs. F	0.085 (0.0079,0.53)	Leukopenia	IC vs. mTCF	5.87 (1.88,2292)
OS	RT vs. T	0.61 (0.41,0.91)	ORR	TF vs. mTCF	0.32 (0.14,0.72)	Leukopenia	I vs. mTCF	78.15 (2.09,3545)
OS	OF vs. TO	0.41 (0.19,0.93)	ORR	OF vs. RT	0.49 (0.33,0.75)	Leukopenia	TOF vs. mTCF	31.66 (2.09,705.4)
PFS	TO vs. TOF	3.80 (1,17.87)	ORR	TO vs. mTCF	0.32 (0.14,0.77)	Leukopenia	TO vs. mTCF	31.77 (2.36,629.7)
PFS	F vs. RT	24.38 (1.07,1227)	ORR	EOF vs. mTCF	0.25 (0.072,0.91)	Leukopenia	TF vs. mTCF	41.55 (3.14,819.4)
PFS	F vs. TOF	41.09 (1.09,3852)	ORR	TC vs. mTCF	0.45 (0.20,0.97)	Leukopenia	TCF vs. mTCF	39.38 (3.43,699)
ORR	F vs. OF	6.73 (1.01,70.23)	ORR	TCF vs. mTCF	0.46 (0.21,0.98)	Leukopenia	T vs. mTCF	71.42 (3.58,1889)
ORR	ECF vs. TC	1.65 (1.02,2.67)	Neutropenia	CF vs. TOF	8.58 (1.10,71.92)	Leukopenia	TC vs. mTCF	52.76 (3.68,1145)
ORR	F vs. TOF	5.69 (1.03,54.36)	Neutropenia	CF vs. RT	13.6 (1.31,134.9)	Leukopenia	OF vs. mTCF	79.97 (5.26,1821)
ORR	I vs. TF	3.42 (1.04,11.04)	Neutropenia	I vs. mTOF	44 (1.53,1576)	Leukopenia	F vs. mTCF	120.6 (7.20,2709)
ORR	IF vs. TF	1.81 (1.04,3.06)	Neutropenia	TF vs. mTOF	47.1 (1.65,1685)	Leukopenia	CF vs. mTCF	75.34 (7.90,1085)
ORR	CF vs. TCF	1.81 (1.06,3.15)	Neutropenia	F vs. mTOF	45.58 (1.66,1546)	Leukopenia	mTCF vs. mTF	0.016 (0.00058,0.34)
ORR	IF vs. TO	1.76 (1.06,2.83)	Neutropenia	CF vs. TO	9.09 (2.01,41.02)	Leukopenia	mTCF vs. mTOF	0.029 (0.00093,0.63)
ORR	T vs. TC	1.62 (1.08,2.33)	Neutropenia	TO vs. mTOF	19.06 (2.04,249.3)	Leukopenia	EOF vs. F	0.016 (0.00025,0.88)
ORR	F vs. mTF	5.94 (1.10,57.33)	Neutropenia	TOF vs. mTOF	19.9 (2.18,258)	Vomiting	EOF vs. TOF	13.35 (1.15,518.5)
ORR	F vs. IF	5.13 (1.12,46.13)	Neutropenia	EOF vs. mTOF	113.3 (3.41,4490)	Vomiting	TF vs. TOF	4.18 (1.29,12.63)
ORR	CF vs. EOF	3.26 (1.13,9.66)	Neutropenia	TC vs. mTOF	49.29 (4.02,837.2)	Vomiting	ECF vs. TOF	5.50 (1.31,29.62)
ORR	ECF vs. TO	2.29 (1.17,4.53)	Neutropenia	T vs. mTOF	69.65 (4.26,1502)	Vomiting	TO vs. TOF	6.87 (2.15,25.18)
ORR	CF vs. mCF	1.97 (1.19,3.37)	Neutropenia	TCF vs. mTOF	55.13 (4.67,883.8)	Vomiting	IF vs. TOF	19.34 (270,157.5)
ORR	T vs. TO	2.25 (1.21,4.07)	Neutropenia	OF vs. mTOF	100.2 (7.10,1751)	Vomiting	F vs. IF	0.029 (0.00083,0.46)
ORR	ECF vs. TF	2.36 (1.24,4.47)	Neutropenia	CF vs. mTOF	174.7 (12.56,3152)	Vomiting	CF vs. TO	0.29 (0.11,0.90)
ORR	CF vs. TC	1.84 (1.33,2.60)	Neutropenia	mTCF vs. mTOF	305.8 (18.62,6592)	Vomiting	CF vs. IF	0.11 (0.013,0.94)
ORR	T vs. TF	2.31 (1.34,3.95)	Neutropenia	TO vs. mTCF	0.063 (0.010,0.37)	Vomiting	TOF vs. mTCF	0.21 (0.042,0.99)

Regarding the endpoint of 1-year PFS, 21 eligible RCTs with 17 chemotherapy regimens (CF, ECF, EOF, F, I, IC, OF, RT, T, TC, TCF, TF, TO, TOF, mTCF, mTF, and mTOF) reported the 1-year PFS. As the results indicate in **Supplementary File 3**, the significant differences were observed between the chemotherapy regimens of TO vs. TOF (OR = 3.80, 95% CI = 1.00–17.87), F vs. RT (OR = 24.38, 95% CI = 1.07–1227), as well as F vs. TOF (OR = 41.09, 95% CI = 1.09–3852). Besides that, the TOF regimen was associated with the remarkable option for improving the 1-year PFS in the light of SUCRA (**Figure S1**), and the ranks of different taxane-based chemotherapy regimens in the 1-year PFS were listed below: TOF (81.29%) > mTOF (79.13%) > RT (56.87%) > mTCF (56.22%) > TF (53.06%) > TO (46.95%) > TCF (44.96%) > mTF (44%) > TC (37.59%) > T (32.69%).

A total of 32 RCTs included 17 chemotherapy regimens (CF, ECF, EOF, F, I, IC, OF, RT, T, TC, TCF, TF, TO, TOF, mTCF, mTF, and mTOF) that provided sufficient information for estimating the ORR. The results of NMA suggested that 32 comparisons exhibited significant differences in this outcome, and 26 of them contained the following taxane-based chemotherapy regimens: ECF vs. TC, F vs. TOF, I vs. TF, IF vs. TF, CF vs. TCF, IF vs. TO, T vs. TC, F vs. mTF, ECF vs. TO, T vs. TO, ECF vs. TF, CF vs. TC, T vs. TF, F vs. mCF, CF vs. TO, F vs. TC, F vs. TCF, CF vs. TF, F vs. TO, F vs. TF, TF vs. mTCF, OF vs. RT, TO vs. mTCF, EOF vs. mTCF, TC vs. mTCF, and TCF vs. mTCF, and the OR and 95% CI are presented in **Supplementary File 3**. Moreover, the TF regimen was believed to be particularly beneficial for improving ORR for patients with gastric cancer according to the SUCRA. The rankings of taxane-based chemotherapy regimens based on their SUCRA value were as follows: TF (85.91%) > TO (84.14%) > TC (64.3%) > TCF (62.34%) > mTF (56.84%) > TOF (54.09%) > T (32.09%) > RT (26.91%) > mTCF (18.77%).

### The Safety Outcomes

Twenty-five RCTs involving 16 chemotherapy regimens (CF, ECF, EOF, F, I, OF, RT, T, TC, TCF, TF, TO, TOF, mTCF, mTF, and mTOF) described neutropenia. The pooled results in **Table 2** showed that 16 comparisons were associated with significant differences in neutropenia as follows: CF vs. TOF, CF vs. RT, I vs. mTOF, TF vs. mTOF, F vs. mTOF, CF vs. TO, TO vs. mTOF, TOF vs. mTOF, EOF vs. mTOF, TC vs. mTOF, T vs. mTOF, TCF vs. mTOF, OF vs. mTOF, CF vs. mTOF, mTCF vs. mTOF, TO vs. mTCF, RT vs. mTCF, RT vs. T, TOF vs. mTCF, TCF vs. mTCF, and TC vs. mTCF. The SUCRA of neutropenia for different taxane-based chemotherapy regimens was arranged as follows: mTCF (93.51%) > T (65.52%) > TCF (57.45%) > TC (54.09%) > TF (53.82%) > TOF (31.22%) > TO (27.93%) > mTF (24.13%) > RT (21.09%) > mTOF (1.70%); these results indicated that the mTCF regimen had the highest probability of being the most favorable treatment in terms of relieving neutropenia.

There were 29 trials with 18 chemotherapy regimens (CF, ECF, EOF, F, I, IC, IF, OF, RT, T, TC, TCF, TF, TO, TOF, mTCF, mTF, and mTOF) concerning leucopenia. As summarized in **Table 2**, there were significant differences between 17 comparisons, and 15 of them were taxane-based chemotherapy regimens, namely, IF vs. mTCF, ECF vs. mTCF, IC vs. mTCF, I vs. mTCF, TOF vs. mTCF, TO vs. mTCF, TF vs. mTCF, TCF vs. mTCF, T vs. mTCF, TC vs. mTCF, OF vs. mTCF, F vs. mTCF, CF vs. mTCF, mTCF vs. mTF, and mTCF vs. mTOF. According to SUCRA for leucopenia, taxane-based chemotherapy regimens were ranked as follows: T (68.23%) > mTF (63.16%) > TC (58.86%) > TF (49.33%) > TCF (46.91%) > mTOF (44.96%) > TOF (39.4%) > TO (38.9%) > RT (33.26%) > mTCF (2.629%). It was suggested that only receiving paclitaxel or docetaxel appeared to have the highest SUCRA value of the decrease in the risk of leucopenia.

The analysis of vomiting included the data from 29 trials with 18 chemotherapy regimens (CF, ECF, EOF, F, I, IC, IF, OF, RT, T, TC, TCF, TF, TO, TOF, mTCF, mTF, and mTOF). The results of NMA demonstrated in **Table 2** that significant differences were detected between these nine comparisons: EOF vs. TOF, TF vs. TOF, ECF vs. TOF, TO vs. TOF, IF vs. TOF, F vs. IF, CF vs. TO, CF vs. IF, and TOF vs. mTCF. Furthermore, TO regimen possessed the great possibility of significantly reduced risk of vomiting compared to other taxane-based chemotherapy regimens, and their ranks were presented as follows based on SUCRA: TO (74.08%) > mTF (63.35%) > mTCF (61.83%) > mTOF (58.02%) > TF (56.27%) > TC (45.72%) > TCF (37.87%) > RT (37.34%) > T (35.02%) > TOF (14.51%).

With respect to fatigue, 29 trials involving 17 chemotherapy regimens (CF, ECF, EOF, F, IC, IF, OF, RT, T, TC, TCF, TF, TO, TOF, mTCF, mTF, and mTOF) were enrolled. Disappointingly, the results revealed that no significant difference was found among these comparisons (**Table 2**). Moreover, mTF exhibited great possibility with the lowest risk of fatigue incidence, and the rankings of taxane-based chemotherapy regimens based on their SUCRA value were as follows: mTF (82.26%) > mTCF (53.82%) > TC (53.11%) > TCF (52.9%) > TF (50.71%) > T (49.82%) > TO (42.88%) > TOF (38.1%) > mTOF (32.87%) > RT (27.8%).

Additionally, the SUCRA values of each chemotherapy regimen for efficacy and safety outcomes are summarized in **Table 3**, and the NMA results from outcomes are described in **Presentation S1**.

**Table 3:** The SUCRA values of each regimen for outcomes.

Intervention	OS	PFS	ORR	Neutropenia	Leukopenia	Vomiting	Fatigue
CF	48.83	39.62	23.82	**84.34**	**72.7**	29.67	**74.86**
ECF	51.51	49.26	31.37	38.94	44.77	67.72	**77.23**
EOF	58.26	66.09	**86.74**	70.69	9.822	**80.6**	34.8
F	54.55	3.541	3.719	52.54	**83.96**	13.8	58.21
I	20.66	52.94	20.49	51.32	67.36	67.8	
IC	1.26E-02	34.36	70.03		58.64	21.39	43.1
IF	67.29		47.31		43.05	**88.35**	25.71
OF	65.03	**71.43**	62.83	**71.71**	**74.08**	46.65	51.82
RT	61.47	56.87	26.91	21.09	33.26	37.34	27.8
T	32.13	32.69	32.09	65.52	68.23	35.02	49.82
TC	34.35	37.59	64.3	54.09	58.86	45.72	53.11
TCF	68.06	44.96	62.34	57.45	46.91	37.87	52.9
TF	**92.15**	53.06	85.91	53.82	49.33	56.27	50.71
TO	16.31	46.95	84.14	27.93	38.9	**74.08**	42.88
TOF	**75.49**	**81.29**	54.09	31.22	39.4	14.51	38.1
mCF	34.11		68.28				
mTCF	**78.88**	56.22	18.77	**93.51**	2.629	61.83	53.82
mTF	66.67	44	56.84	24.13	63.16	63.35	**82.26**
mTOF	24.22	**79.13**		1.699	44.96	58.02	32.87

The values in bold font have higher SUCRA values for different outcomes.

### Cluster Analysis

**Figure 4:**
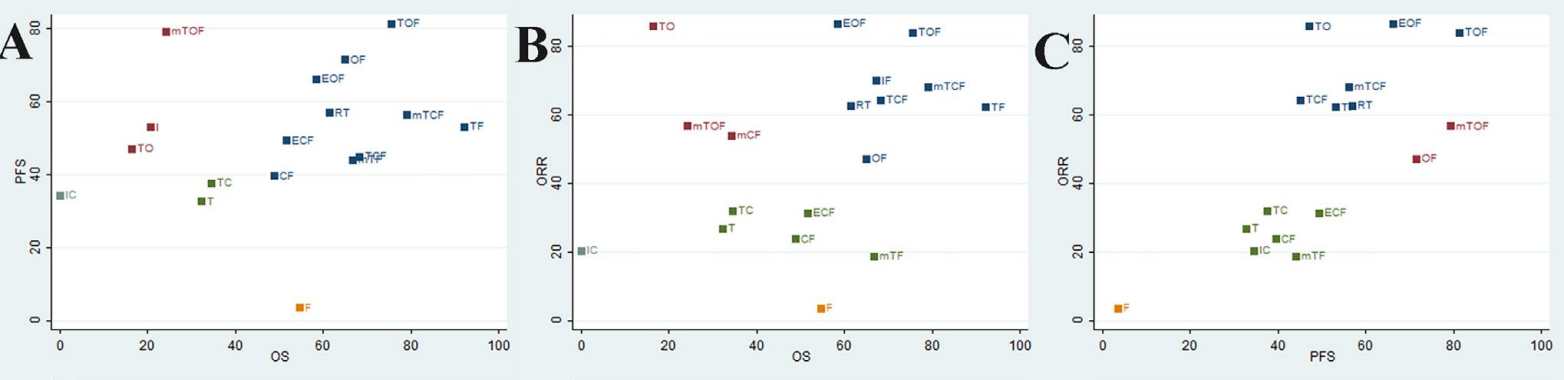
Cluster analysis plot of the efficacy outcomes. The interventions located in the upper right corner were superior to others. **(A)** OS (*X* axis) and PFS (*Y* axis); **(B)** OS (*X* axis) and ORR (*Y* axis); **(C)** ORR (*X* axis) and PFS (*Y* axis).

To categorize the different chemotherapy regimens into distinctive groups and estimate the most effective and safe taxane-based chemotherapy regimens, we conducted a cluster analysis for these RCTs that simultaneously described the details of several outcomes. On the one hand, the results of cluster analysis that are shown in **Figure 4** revealed that the regimen of TOF, mTCF, and TF were associated with the most favorable clinical efficacy in improving OS, PFS, and ORR compared with other taxane-based chemotherapy regimens. On the other hand, the regimens of T and mTF had the potential to be the most tolerable and acceptable therapeutic alternative in terms of ADRs. Overall, the combination of taxanes (paclitaxel or docetaxel) and fluorouracil had the potential to be the most preferable and beneficial option for patients with gastric cancer in consideration of both efficacy and safety.

### Publication Bias

**Figure 5:**
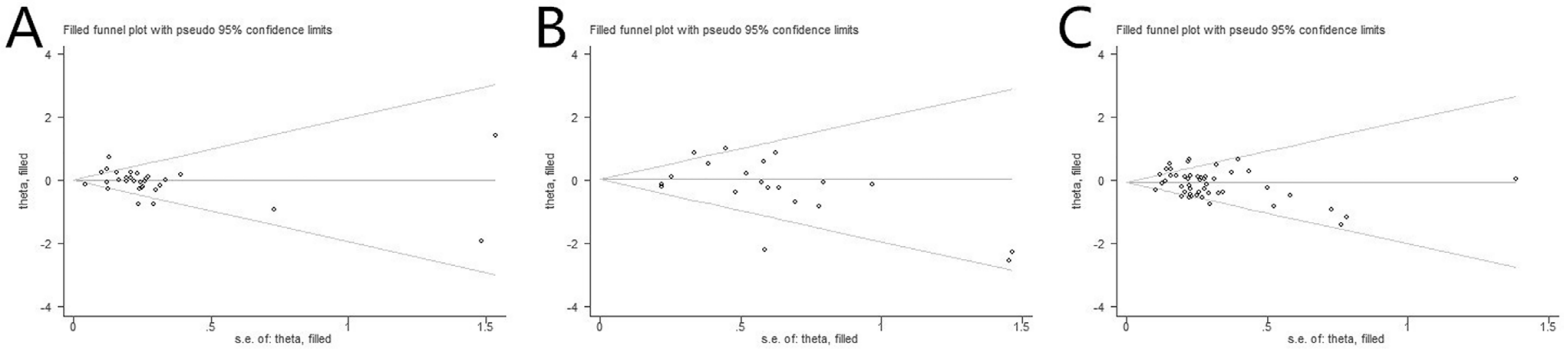
Funnel plot of the efficacy outcomes among included RCTs. **(A)** OS; **(B)** PFS; **(C)** ORR.

As depicted in **Figure 5**, the publication bias of included RCTs was measured by funnel plots and Begg’s and Egger’s tests. The results of 1-year OS, 1-year PFS, and ORR were as follows: Egger test (*t* = −0.08, *P* = .939 > .05) and Begg test (*z* = 1.44, *P* = .149 > .05), Egger test (*t* = −1.20, *P* = .247 > .05) and Begg test (*z* = 1.75, *P* = .090 > .05), and Egger test (*t* = −1.88, *P* = .067 > .05) and Begg test (*z* = 1.60, *P* = .109 > .05), respectively. Also, the symmetric remaining part was used to estimate the center value of the funnel plot, and the parts along the center sides made up the sheared part and missing parts. According to the funnel plot, after being patched the actual value of the combined effect was estimated, and the number of RCTs increased while no qualitative change was produced in the results of publication bias ultimately. Thus, there was no significant publication bias among the included RCTs in the present NMA.

### Consistency Test

The consistency test was preformed for the outcome of 1-year OS (**Figure S2**); this NMA involved 12 triangular loops and 4 quadrangle loops. The 95% CIs of IF values were truncated at zero for 15 closed loops, indicating that there is no evidence of significant inconsistency. Nevertheless, the significant inconsistency was observed in the quadrangle loop of (TC-TCF-TF-TO) (IF = 2.13, 95% CI = 0.57–3.69). Collectively, there was some inconsistency in this study.

## Discussion

For the comparative efficacy and safety of different taxane-based chemotherapy regimens against gastric cancer, we adopted the approach of NMA for providing the overwhelming evidence from published RCTs. In summary, the results of the present NMA indicated that the combination of taxanes (paclitaxel or docetaxel) and fluorouracil was associated with the most preferable and beneficial option for patients with gastric cancer in consideration of both efficacy and safety. Moreover, the choice of specific taxane-based chemotherapy regimens should simultaneously rely on the high-quality evidence-based research, the clinical practice of oncologists, and the physique of patients with gastric cancer. Attention should be given to the ADRs caused by paclitaxel and docetaxel to achieve the highest clinical benefits to avoid or decrease the occurrence of adverse effects.

Throughout the past century, gastric cancer is one of the most common malignant tumors of the digestive system worldwide (Chen et al., 2016). Over the past three decades, the survival time of patients with gastric cancer has prolonged and their performance status has improved with the development of different therapeutic strategies for the treatment of advanced gastric cancer (MacDonald et al., 1980; Cocconi et al., 1994; Chao et al., 2004). Among various chemotherapeutic drugs, paclitaxel is an effective anticancer drug against a wide range of solid tumors (Blagosklonny and Fojo, 1999). The antitumor activity of paclitaxel was discovered in the 1970s and has been approved as a microtubule stabilizing agent since 1992, and evidence clearly indicates that paclitaxel can block progression of mitosis, promote tubulin polymerization, and stabilize microtubules from depolymerizing (De Furia, 1997; Rodríguez-Antona, 2010; Meng et al., 2016). Recently, it was reported that the mechanism of paclitaxel is associated with the downregulation of COX-2 expression to inhibit migration and invasion of gastric cancer cells more effectively (Sun et al., 2015). Additionally, docetaxel resistance can be reversed *via* the inhibition of FOXM1, which might be a useful marker for predicting and monitoring docetaxel response and a new therapeutic target in docetaxel-resistant gastric cancer (Li et al., 2014). Based on several clinical trials, paclitaxel and docetaxel have been identified to improve the outcomes of patients with gastric cancer; in addition, paclitaxel or docetaxel significantly increases OS compared with placebo and has promising activity with acceptable adverse toxicities (Goel, 2012; Tsuburaya et al., 2013; Yamaguchi et al., 2013; Meng et al., 2014; Bang et al., 2015). Despite recent advancements aimed at optimizing taxane-based regimens, with respect to safety, anaphylactic reactions and hematologic toxicity have been frequently reported as the main adverse effects of paclitaxel or docetaxel, and these reactions could diminish with corticosteroids and antihistamine premedication (Blagosklonny and Fojo, 1999; Raisch et al., 2011).

To the best of our knowledge, this is the first systematic review with NMA that investigated the comparative efficacy and safety of the taxane-based chemotherapy regimens against gastric cancer. A total of 10 regimens (RT, T, TC, TCF, TF, TO, TOF, mTCF, mTF, and mTOF) were evaluated for the efficacy and safety outcomes. The efficacy outcomes in the present study involve the 1-year OS, 1-year PFS, and ORR, and the safety outcomes were ADRs, such as neutropenia, leucopenia, vomiting, and fatigue. The hierarchy was calculated based on the SUCRA to identify the optimal treatment for each outcome; the cluster analysis was performed to estimate the superior taxane-based regimen account for both efficacy and safety. Besides, our search strategies were comprehensive to support our results, and the inclusion criteria were formulated and established strictly through the selection process of potential and eligible RCTs. Moreover, this NMA only focused on the chemotherapeutic drugs in NCCN guidelines to avoid clinical heterogeneity. Finally, the methodological quality assessment was conducted for the included RCTs; the comparison-adjusted funnel plots and Egger’s test and Begg test were adopted to measure publication bias; the consistency test in node-splitting analysis for each loop was used to explore the reliability and credibility of both direct and indirect evidence.

Several limitations of this current NMA should be taken into consideration. First, the survival time or follow-up data were regarded as important for judging the therapeutic effects of patients with cancer; however, the majority of included RCT only provided the information on 1-year OR and PFS. The insufficient data about long-term endpoint were susceptible to interference in clinical heterogeneity; further study with final OS data will be essential. Therefore, the clinical trials of patients with cancer should focus on more meaningful endpoints. Besides, the information of intention-to-treat analysis was also not enough to perform the NMA among included trials. Second, the majority of RCTs included in the study exhibited a relatively high risk of bias in inadequate allocation concealment and blinding. Finally, we merged docetaxel and paclitaxel into the drug class of taxanes, and these two drugs might have slight differences for treating gastric cancer. We did not conduct a subgroup analysis for Asian and non-Asian patients because several of the included RCTs enrolled both Asian and non-Asian patients with gastric cancer. Hence, head-to-head RCTs comparing docetaxel and paclitaxel would be valuable in identifying the clinical benefits for the former and the latter. Future studies should be designed to address if Asian or non-Asian patients produce changes in receiving taxanes against gastric cancer.

## Conclusion

In conclusion, the current evidence suggests that the combination of taxanes (paclitaxel or docetaxel) and fluorouracil was associated with the most preferable and beneficial option for patients with gastric cancer, although additional results from multicenter trials and high-quality studies will be pivotal for supporting our findings.

## Author Contributions

DZ, J-RW, K-HW, and X-JD contributed to the conception and design. DZ, J-RW, K-HW, X-JD, YZ, M-WN, X-MZ and S-YL contributed to the development of methodology. DZ, J-RW, K-HW, X-JD, YZ, M-WN, S-YL, and BZ contributed to the acquisition of data. DZ, J-RW, K-HW, X-JD, YZ, M-WN, S-YL, and BZ contributed to the analysis and interpretation of data. DZ, J-RW, and BZ performed the writing, review, and/or revision of the manuscript. Administrative, technical, or material support was provided by DZ, J-RW, K-HW, X-JD, X-MZ, and YZ. DZ, J-RW, K-HW, and X-JD performed study supervision.

## Funding

This work was funded by the National Natural Science Foundation of China (Nos. 81473547 and 81673829) and the Young Scientists Training Program of Beijing University of Chinese Medicine.

## Conflict of Interest Statement

The authors declare that the research was conducted in the absence of any commercial or financial relationships that could be construed as a potential conflict of interest.

## References

[B1] Achana F. A. Cooper N. J. Bujkiewicz S. Hubbard S. J. Kendrick D. Jones D. R. (2014). Network meta-analysis of multiple outcome measures accounting for borrowing of information across outcomes. BMC Med. Res. Methodol. 14, 92. 10.1186/1471-2288-14-92 25047164 PMC4142066

[B2] Ajani J. A. D’Amico T. A. Almhanna K. Bentrem D. J. D’Amico T. A. Das P. (2016). Gastric cancer, version 3.2016, NCCN clinical practice guidelines in oncology. J. Natl. Compr. Canc. Netw. 10, 1286–1312. 10.6004/jnccn.2016.0137 27697982

[B3] Ajani J. A. Fodor M. B. Tjulandin S. A. Moiseyenko V. M. Chao Y. Cabral Filho S. (2005). Phase II multi-institutional randomized trial of docetaxel plus cisplatin with or without fluorouracil in patients with untreated, advanced gastric, or gastroesophageal adenocarcinoma. J. Clin. Oncol. 24, 5660–5667. 10.1200/JCO.2005.17.376 16110025

[B4] Al-Batran S. E. Pauligk C. Homann N. Hartmann J. T. Moehler M. Probst S. (2013). The feasibility of triple-drug chemotherapy combination in older adult patients with oesophagogastric cancer: a randomised trial of the Arbeitsgemeinschaft Internistische Onkologie (FLOT65+). Eur. J. Cancer. 4, 835–842. 10.1016/j.ejca.2012.09.025 23063354

[B5] Bang Y. J. Im S. A. Lee K. W. Cho J. Y. Song E. K. Lee K. H. (2015). Randomized, double-blind phase II trial with prospective classification by ATM protein level to evaluate the efficacy and tolerability of olaparib plus paclitaxel in patients with recurrent or metastatic gastric cancer. J. Clin. Oncol. 33, 3858–3865. 10.1200/JCO.2014.60.0320 26282658

[B6] Blagosklonny M. V. Fojo T. (1999). Molecular effects of paclitaxel: myths and reality (a critical review). Int. J. Cancer 2, 151–156. 10.1002/(SICI)1097-0215(19991008)83:2<151::AID-IJC1>3.0.CO;2-5 10471519

[B7] Bocci G. Di Paolo A. Danesi R. (2013). The pharmacological bases of the antiangiogenic activity of paclitaxel. Angiogenesi. 3, 481–492. 10.1007/s10456-013-9334-0 PMC3682088 23389639

[B8] Brower V. (2015). Modified gastric cancer chemotherapy: more effective, less toxic. Lancet Oncol. 16, e590. 10.1016/S1470-2045(15)00442-8 26511647

[B9] Casak S. J. Fashoyin-Aje I. Lemery S. J. Zhang L. Jin R. Li H. (2015). FDA approval summary: ramucirumab for gastric cancer. Clin. Cancer Res. 15, 3372–3376. 10.1158/1078-0432.CCR-15-0600 26048277

[B10] Chaimani A. Higgins J. P. Mavridis D. Spyridonos P. Salanti G. (2013). Graphical tools for network meta-analysis in STATA. PLoS One 10, e76654. 10.1371/journal.pone.0076654 PMC3789683 24098547

[B11] Chan J. S. (2016). Bayesian informative dropout model for longitudinal binary data with random effects using conditional and joint modeling approaches. Biom. J. 3, 549–569. 10.1002/bimj.201400064 26467236

[B12] Chao Y. Yeh K. H. Chang C. J. Chen L. T. Chao T. Y. Wu M. F. (2004). Phase II study of weekly oxaliplatin and 24-h infusion of high-dose 5-fluorouracil and folinic acid in the treatment of advanced gastric cancer. Br. J. Cancer. 3, 453–458. 10.1038/sj.bjc.6601985 PMC2409850 15226770

[B13] Chen W. Zheng R. Baade P. D. Zhang S. Zeng H. Bray F. (2016). Cancer statistics in China, 2015. CA Cancer J. Clin. 66, 115–132. 10.3322/caac.21338 26808342

[B14] Cocconi G. Bella M. Zironi S. Algeri R. Costanzo F. De Lisi V. (1994). Fluorouracil, doxorubicin, and mitomycin combination versus PELF chemotherapy in advanced gastric cancer: a prospective randomized trial of the Italian Oncology Group for Clinical Research. J. Clin. Oncol. 12, 2687–2893. 10.1200/JCO.1994.12.12.2687 7989945

[B15] Constenla M. Garcia-Arroyo R. Lorenzo I. Carrete N. Campos B. Palacios P. (2002). Docetaxel, 5-fluorouracil, and leucovorin as treatment for advanced gastric cancer: results of a phase II study. Gastric Cancer 3, 142–147. 10.1007/s101200200025 12378340

[B16] Cunningham D. Allum W. H. Stenning S. P. Thompson J. N. de Velde C. J. Nicolson M. (2006). Perioperative chemotherapy versus surgery alone for resectable gastroesophageal cancer. N. Engl. J. Med. 1, 11–20. 10.1056/NEJMoa055531 16822992

[B17] De Furia M. D. (1997). Paclitaxel (Taxol^®^): a new natural product with major anticancer activity. Phytomedicine 4, 273–282. 10.1016/S0944-7113(97)80081-5 23195489

[B18] de Martel C. Ferlay J. Franceschi S. Vignat J. Bray F. Forman D. (2012). Global burden of cancers attributable to infections in 2008: a review and synthetic analysis. Lancet Oncol. 6, 607–615. 10.1016/S1470-2045(12)70137-7 22575588

[B19] Donegan S. Williamson P. D’Alessandro U. Tudur Smith C. (2013). Assessing key assumptions of network meta-analysis: a review of methods. Res. Synth. Methods. 4, 291–323. 10.1002/jrsm.1085 26053945

[B20] Ferro A. Peleteiro B. Malvezzi M. Bosetti C. Bertuccio P. Levi F. (2014). Worldwide trends in gastric cancer mortality (1980-2011), with predictions to 20715, and incidence by subtype. Eur. J. Cancer. 7, 1330–1344. 10.1016/j.ejca.2014.01.029 24650579

[B21] Goel G. (2012). Long term complete remission in advanced gastric adenocarcinoma with docetaxel, oxaliplatin and capecitabine combination regimen. World J. Oncol. 3, 124–126. 10.4021/wjon469w 29147293 PMC5649791

[B22] Golfinopoulos V. Salanti G. Pavlidis N. Ioannidis J. P. A. (2007). Survival and disease-progression benefits with treatment regimens for advanced colorectal cancer: a meta-analysis. Lancet Oncol. 8, 898–911. 10.1016/S1470-2045(07)70281-4 17888735

[B23] Gómez-Martin C. Sánchez A. Irigoyen A. Llorente B. Pérez B. Serrano R. (2012). Incidence of hand-foot syndrome with capecitabine in combination with chemotherapy as first-line treatment in patients with advanced and/or metastatic gastric cancer suitable for treatment with a fluoropyrimidine-based regimen. Clin. Transl. Oncol. 9, 689–697. 10.1007/s12094-012-0858-3 22855151

[B24] Greco T. Landoni G. Biondi-Zoccai G. D’Ascenzo F. Zangrillo A. (2016). A Bayesian network meta-analysis for binary outcome: how to do it. Stat. Methods Med. Res. 5, 1757–1773. 10.1177/0962280213500185 23970014

[B25] Gubanski M. Johnsson A. Fernebro E. Kadar L. Karlberg I. Flygare P. (2010). Randomized phase II study of sequential docetaxel and irinotecan with 5-fluorouracil/folinic acid (leucovorin) in patients with advanced gastric cancer: the GATAC trial. Gastric Cancer 3, 155–161. 10.1007/s10120-010-0553-4 20820984

[B26] Gunderson L. L. (2002). Gastric cancer-patterns of relapse after surgical resection. Semin. Radiat. Oncol. 12, 150–161. 10.1053/srao.2002.30817 11979416

[B27] Guo Z. Wang X. Lin R. Chen L. Fan N. Chen Y. (2015). Paclitaxel-based regimens as first-line treatment in advanced gastric cancer. J Chemother. 2, 94–98. 10.1179/1973947814Y.0000000169 24548091

[B28] Hans-Peter P. (2014). Network-meta analysis made easy: detection of inconsistency using factorial analysis-of-variance models. BMC Med. Res. Methodol. 14, 61. 10.1186/1471-2288-14-61 24885590 PMC4049370

[B29] Higgins J. P. Altman D. G. Gøtzsche P. C. Jüni P. Moher D. Oxman A. D. (2011). The Cochrane Collaboration’s tool for assessing risk of bias in randomised trials. BMJ. 343, d5928. 10.1136/bmj.d5928 22008217 PMC3196245

[B30] Hironaka S. Ueda S. Yasui H. Nishina T. Tsuda M. Tsumura T. (2013). Randomized, open-label, phase III study comparing irinotecan with paclitaxel in patients with advanced gastric cancer without severe peritoneal metastasis after failure of prior combination chemotherapy using fluoropyrimidine plus platinum: WJOG 4007 trial. J. Clin. Oncol. 35, 4438–4444. 10.1200/JCO.2012.48.5805 24190112

[B31] Howat S. Park B. Oh I. S. Jin Y. W. Lee E. K. Loake G. J. (2014). Paclitaxel: biosynthesis, production and future prospects. N. Biotechnol. 3, 242–5. 10.1016/j.nbt.2014.02.010 24614567

[B32] Hutton B. Salanti G. Caldwell D. M. Chaimani A. Schmid C. H. Cameron C. (2015). The PRISMA extension statement for reporting of systematic reviews incorporating network meta-analyses of health care interventions: checklist and explanations. Ann. Intern. Med. 162, 777–784. 10.7326/M14-2385 26030634

[B33] Inal A. Kaplan M. A. Kucukoner M. Isikdogan A. (2012). Docetaxel and cisplatin plus fluorouracil compared with modified docetaxel, cisplatin, and 5-fluorouracil as first-line therapy for advanced gastric cancer: a retrospective analysis of single institution. Neoplasma 2, 233–236. 10.4149/neo_2012_030 22248282

[B34] Jackson D. Turner R. Rhodes K. Viechtbauer W. (2014). Methods for calculating confidence and credible intervals for the residual between-study variance in random effects meta-regression models. BMC Med. Res. Methodol. 14, 103. 10.1186/1471-2288-14-103 25196829 PMC4160560

[B35] Jiang H. Qian J. Zhao P. Zhang X. Zheng Y. Mao C. (2015). A phase II study of biweekly S-1 and paclitaxel (SPA) as first-line chemotherapy in patients with metastatic or advanced gastric cancer. Cancer Chemother. Pharmacol. 1, 197–203. 10.1007/s00280-015-2782-z 26013324

[B36] Khanna C. Rosenberg M. Vail D. M. (2015). A review of paclitaxel and novel formulations including those suitable for use in dogs. J. Vet. Intern. Med. 4, 1006–1012. 10.1111/jvim.12596 PMC4895360 26179168

[B37] Kim J. Y. Ryoo H. M. Bae S. H. Kang B. W. Chae Y. S. Yoon S. (2015). Multi-center randomized phase II study of weekly docetaxel versus weekly docetaxel-plus-oxaliplatin as a second-line chemotherapy for patients with advanced gastric cancer. Anticancer Res. 6, 3531–3536. 26026121

[B38] Kim Y. S. Sym S. J. Park S. H. Park I. Hong J. Ahn H. K. (2014). A randomized phase II study of weekly docetaxel/cisplatin versus weekly docetaxel/oxaliplatin as first-line therapy for patients with advanced gastric cancer. Cancer Chemother. Pharmacol. 1, 163–169. 10.1007/s00280-013-2334-3 24202666

[B39] Kos F. T. Uncu D. Ozdemir N. Budakoglu B. Odabaş H. Abali H. (2011). Comparison of cisplatin-5-fluorouracil-folinic acid versus modified docetaxel-cisplatin-5-fluorouracil regimens in the first-line treatment of metastatic gastric cancer. Chemotherapy 3, 230–235. 10.1159/000327840 21597287

[B40] Krahn U. Binder H. König J. (2014). Visualizing inconsistency in network meta-analysis by independent path decomposition. BMC Med. Res. Methodol. 14, 131. 10.1186/1471-2288-14-131 25510877 PMC4279676

[B41] Kudlowitz D. Muggia F. (2013). Defining risks of taxane neuropathy: insights from randomized clinical trials. Clin. Cancer Res. 17, 4570–4577. 10.1158/1078-0432.CCR-13-0572 23817688

[B42] Kundranda M. N. Niu J. (2015). Albumin-bound paclitaxel in solid tumors: clinical development and future directions. Drug Des. Devel. Ther. 9, 3767–77. 10.2147/DDDT.S88023 PMC4521678 26244011

[B43] Lee K. W. Kim B. J. Kim M. J. Han H. S. Kim J. W. Park Y. I. (2017). A multicenter randomized phase II study of docetaxel vs. Cancer Res. Treat. 3, 706–716. 10.4143/crt.2016.216 PMC5512362 27764906

[B44] Li J. Li B. Zhong M. (2009). The efficacy of different combined chemotherapy regimens for advanced gastric carcinoma. Chin. J. Clin. Oncol. 8, 205–207.

[B45] Li X. Yao R. Yue L. Qiu W. Qi W. Liu S. (2014). FOXM1 mediates resistance to docetaxel in gastric cancer via up-regulating Stathmin. J. Cell. Mol. Med. 5, 811–823. 10.1111/jcmm.12216 PMC4119387 24628949

[B46] Li X. D. Shen H. Jiang J. T. Zhang H. Z. Zheng X. Shu Y. Q. (2011). Paclitaxel based vs oxaliplatin based regimens for advanced gastric cancer. World J. Gastroenterol. 8, 1082–1087. 10.3748/wjg.v17.i8.1082 PMC3057154 21448363

[B47] Liao W. C. Chien K. L. Lin Y. L. Wu M. S. Lin J. T. Wang H. P. (2013). Adjuvant treatments for resected pancreatic adenocarcinoma: a systematic review and network meta-analysis. Lancet Oncol. 14, 1095–1103. 10.1016/S1470-2045(13)70388-7 24035532

[B48] Lim D. H. Park S. H. Park K. W. Kang J. H. Oh S. Y. Hwang I. G. (2010). Retrospective analyses of cisplatin-based doublet combination chemotherapy in patients with advanced gastric cancer. BMC Cancer 10, 583. 10.1186/1471-2407-10-583 20977739 PMC2978206

[B49] Liu Y. P. Li G. Q. Chen H. H. (2015). Capecitabine for treatment of patients with advanced gastric cancer: curative efficacy and effect on serum levels of MMP-2 and MMP-9. World Chi. J. Digestology 7, 1136–1140. 10.11569/wcjd.v23.i7.1136

[B50] Li X. Qiu W. Liu B. Yao R. Liu S. Yao Y. (2013). Forkhead box transcription factor 1 expression in gastric cancer: FOXM1 is a poor prognostic factor and mediates resistance to docetaxel. J. Transl. Med. 11, 204. 10.1186/1479-5876-11-204 24004449 PMC3766246

[B51] Lu M. Wang T. Wang J. (2016). Effects of paclitaxel liposome and capecitabine in the treatment of advanced gastric cancer by clinical observation. Int. J. Clin. Pharmacol. Ther. 9, 693–697. 10.5414/CP202568 27390051

[B52] MacDonald J. S. Schein P. S. Woolley P. V. Smythe T. Ueno W. Hoth D. (1980). 5-Fluorouracil, doxorubicin, and mitomycin (FAM) combination chemotherapy for advanced gastric cancer. Ann. Intern. Med. 93, 533–536. 10.7326/0003-4819-93-4-533 7436184

[B53] Maruta F. Ishizone S. Hiraguri M. Fujimori Y. Shimizu F. Kumeda S. (2007). A clinical study of docetaxel with or without 5′DFUR as a second-line chemotherapy for advanced gastric cancer. Med. Oncol. 1, 71–75. 10.1007/BF02685905 17673814

[B54] Mavridis D. Welton N. J. Sutton A. Salanti G. (2014). A selection model for accounting for publication bias in a full network meta-analysis. Stat. Med. 30, 5399–5412. 10.1002/sim.6321 25316006

[B55] Meng C. Yin H. Sun Z. Zhou J. Chen S. Bai C. (2014). Adjuvant chemotherapy with docetaxel, cisplatin, and continuous-infusion 5-fluorouracil for gastric cancer: a phase II study. Transl. Oncol. 2, 277–283. 10.1016/j.tranon.2014.02.014 PMC4101342 24704535

[B56] Meng Z. Lv Q. Lu J. Yao H. Lv X. Jiang F. (2016). Prodrug strategies for paclitaxel. Int. J. Mol. Sci. 5, E796. 10.3390/ijms17050796 PMC4881612 27223283

[B57] Miceli R. Tomasello G. Bregni G. Di Bartolomeo M. Pietrantonio F. (2014). Adjuvant chemotherapy for gastric cancer: current evidence and future challenges. World J. Gastroenterol. 28, 16:4516–25. 10.3748/wjg.v20.i16.4516 PMC4000488 24782604

[B58] Muro K. Oh S. C. Shimada Y. Lee K. W. Yen C. J. Chao Y. (2016). Subgroup analysis of East Asians in RAINBOW: a phase 3 trial of ramucirumab plus paclitaxel for advanced gastric cancer. J. Gastroenterol. Hepatol. 3, 581–589. 10.1111/jgh.13153 26317322

[B59] Nishina T. Boku N. Gotoh M. Shimada Y. Hamamoto Y. Yasui H. (2016). Randomized phase II study of second-line chemotherapy with the best available 5-fluorouracil regimen versus weekly administration of paclitaxel in far advanced gastric cancer with severe peritoneal metastases refractory to 5-fluorouracil-containing regimens (JCOG0407). Gastric Cancer 3, 902–910. 10.1007/s10120-015-0542-8 26386560

[B60] Ochenduszko S. Puskulluoglu M. Konopka K. Fijorek K. Urbanczyk K. Budzynski A. (2015). Comparison of efficacy and safety of first-line palliative chemotherapy with EOX and mDCF regimens in patients with locally advanced inoperable or metastatic HER2-negative gastric or gastroesophageal junction adenocarcinoma: a randomized phase 3 trial. Med. Oncol. 10, 242. 10.1007/s12032-015-0687-7 PMC4564435 26354521

[B61] Raisch D. W. Campbell W. Garg V. Qureshi Z. P. Bookstaver P. B. Norris L. B. (2011). Description of anaphylactic reactions to paclitaxel and docetaxel reported to the FDA, with a focus on the role of premedication. Expert Opin. Drug. Saf. 4, 521–528. 10.1517/14740338.2011.582865 21595611

[B62] Rodríguez-Antona C. (2010). Pharmacogenomics of paclitaxel. Pharmacogenomics 5, 621–623. 10.2217/pgs.10.32 20415548

[B63] Roth A. D. Ajani J. (2003). Docetaxel-based chemotherapy in the treatment of gastric cancer. Ann. Oncol. 14 (SUPPL. 2), i41–44. 10.1093/annonc/mdg728 12810457

[B64] Roth A. D. Fazio N. Stupp R. Falk S. Bernhard J. Saletti P. (2007). Docetaxel, cisplatin, and fluorouracil; docetaxel and cisplatin; and epirubicin, cisplatin, and fluorouracil as systemic treatment for advanced gastric carcinoma: a randomized phase II trial of the Swiss Group for Clinical Cancer Research. J. Clin. Oncol. 22, 3217–32123. 10.1200/JCO.2006.08.0135 17664469

[B65] Rücker G. Schwarzer G. (2015). Ranking treatments in frequentist network meta-analysis works without resampling methods. BMC Med. Res. Methodol. 15, 58. 10.1186/s12874-015-0060-8 26227148 PMC4521472

[B66] Salanti G. Giovane C. Chaimani A. Caldwell D. M. Higgins J. P. (2014). Evaluating the quality of evidence from a network meta-analysis. PLoS One 7, e99682. 10.1371/journal.pone.0099682 PMC4084629 24992266

[B67] Shah M. A. Janjigian Y. Y. Stoller R. Shibata S. Kemeny M. Krishnamurthi S. (2015). Randomized multicenter phase II study of modified docetaxel, cisplatin, and fluorouracil (DCF) versus DCF plus growth factor support in patients with metastatic gastric adenocarcinoma: a study of the US gastric cancer consortium. J. Clin. Oncol. 33, 3874–3879. 10.1200/JCO.2015.60.7465 26438119

[B68] Shim S. Yoon B. H. Shin I. S. Bae J. M. (2017). Network meta-analysis: application and practice using Stata. Epidemiol Health. 39, e2017047. 10.4178/epih.e2017047 29092392 PMC5733388

[B69] Song T. Chai G. Liu Y. Xie M. Chen Q. Yu X. (2015). Mechanism of synergy of BH3 mimetics and paclitaxel in chronic myeloid leukemia cells: Mcl-1 inhibition. Eur. J. Pharm. Sci. 70, 64–71. 10.1016/j.ejps.2015.01.003 25596561

[B70] Stephenson M. Fleetwood K. Yellowlees A. (2015). Alternatives to Winbugs for network meta-analysis. Value Health 7, A720. 10.1016/j.jval.2015.09.2730

[B71] Sun K. Tang X. H. Xie Y. K. (2015). Paclitaxel combined with harmine inhibits the migration and invasion of gastric cancer cells through downregulation of cyclooxygenase-2 expression. Oncol. Lett. 3, 1649–1654. 10.3892/ol.2015.3425 PMC4533729 26622726

[B72] Teker F. Yilmaz B. Kemal Y. Kut E. Yucel I. (2014). Efficacy and safety of docetaxel or epirubicin, combined with cisplatin and fluorouracil (DCF and ECF), regimens as first line chemotherapy for advanced gastric cancer: a retrospective analysis from Turkey. Asian Pac. J. Cancer Prev. 16, 6727–6732. 10.7314/APJCP.2014.15.16.6727 25169516

[B73] Thuss-Patience P. C. Kretzschmar A. Dogan Y. Dogan Y. Rothmann F. Blau I. (2011). Docetaxel and capecitabine for advanced gastric cancer: investigating dose-dependent efficacy in two patient cohorts. Br. J. Cancer. 4, 505–512. 10.1038/bjc.2011.278 PMC3170974 21792201

[B74] Thuss-Patience P. C. Kretzschmar A. Repp M. Kingreen D. Hennesser D. Micheel S. (2005). Docetaxel and continuous-infusion fluorouracil versus epirubicin, cisplatin, and fluorouracil for advanced gastric adenocarcinoma: a randomized phase II study. J. Clin. Oncol. 3, 494–501. 10.1200/JCO.2005.02.163 15659494

[B75] Torre L. A. Bray F. Siegel R. L. Ferlay J. Lortet-Tieulent J. Jemal A. (2015). Global cancer statistics, 2012. CA Cancer J. Clin. 65, 87–108. 10.3322/caac.21262 25651787

[B76] Trinquart L. Chatellier G. Ravaud P. (2012). Adjustment for reporting bias in network meta-analysis of antidepressant trials. BMC Med. Res. Methodol. 12, 150. 10.1186/1471-2288-12-150 23016799 PMC3537713

[B77] Trinquart L. Attiche N. Bafeta A. Porcher R. Ravaud P. (2016). Uncertainty in treatment rankings: reanalysis of network meta-analyses of randomized trials. Ann. Intern. Med. 10, 666–673. 10.7326/M15-2521 27089537

[B78] Tsuburaya A. Nagata N. Cho H. Hirabayashi N. Kobayashi M. Kojima H. (2013). Phase II trial of paclitaxel and cisplatin as neoadjuvant chemotherapy for locally advanced gastric cancer. Cancer Chemother. Pharmacol. 5, 1309–1314. 10.1007/s00280-013-2130-0 23463482

[B79] Tsuburaya A. Yoshida K. Kobayashi M. Yoshino S. Takahashi M. Takiguchi N. (2014). Sequential paclitaxel followed by tegafur and uracil (UFT) or S-1 versus UFT or S-1 monotherapy as adjuvant chemotherapy for T4a/b gastric cancer (SAMIT): a phase 3 factorial randomised controlled trial. Lancet Oncol. 8, 886–893. 10.1016/S1470-2045(14)70025-7 24954805

[B80] Tsukada T. Fushida S. Harada S. Terai S. Yagi Y. Kinoshita J. (2013). Low-dose paclitaxel modulates tumour fibrosis in gastric cancer. Int. J. Oncol. 42, 1167–1174. 10.3892/ijo.2013.1801 23443842 PMC3622657

[B81] Van Cutsem E. Boni C. Tabernero J. Massuti B. Middleton G. Dane F. (2015). Docetaxel plus oxaliplatin with or without fluorouracil or capecitabine in metastatic or locally recurrent gastric cancer: a randomized phase II study. Ann. Oncol. 1, 149–156. 10.1093/annonc/mdu496 25416687

[B82] Van Cutsem E. Moiseyenko V. M. Tjulandin S. Majlis A. Constenla M. Boni C. (2006). Phase III study of docetaxel and cisplatin plus fluorouracil compared with cisplatin and fluorouracil as first-line therapy for advanced gastric cancer: a report of the V325 Study Group. J. Clin. Oncol. 31, 4991–4997. 10.1200/JCO.2006.06.8429 17075117

[B83] Veroniki A. A. Soobiah C. Tricco A. C. Elliott M. J. Straus S. E. (2015). Methods and characteristics of published network meta-analyses using individual patient data: Protocol for a scoping review. BMJ Open 4, e007103. 10.1136/bmjopen-2014-007103 PMC4420933 25926144

[B84] Wang J. Xu R. Li J. Bai Y. Liu T. Jiao S. (2016). Randomized multicenter phase III study of a modified docetaxel and cisplatin plus fluorouracil regimen compared with cisplatin and fluorouracil as first-line therapy for advanced or locally recurrent gastric cancer. Gastric Cancer 1, 234–244. 10.1007/s10120-015-0457-4 PMC4688303 25604851

[B85] Wang S.-Y. Chu H. Shamliyan T. Jalal H. Kuntz K. M. Kane R. L. (2012). Network meta-analysis of margin threshold for women with ductal carcinoma in situ. J. Natl. Cancer Inst. 104, 507–516. 10.1093/jnci/djs142 22440677 PMC3916966

[B86] Wang P. Wang H. Huang Q. Peng C. Yao L. Chen H. (2019). Exosomes from M1-polarized macrophages enhance paclitaxel antitumor activity by activating macrophages-mediated inflammation. Theranostics 9, 1714–1727. 10.7150/thno.30716 31037133 PMC6485189

[B87] Weaver B. A. (2014). How Taxol/paclitaxel kills cancer cells. Mol. Biol. Cell 18, 2677–2681. 10.1091/mbc.e14-04-0916 PMC4161504 25213191

[B88] Wilke H. Clingan P. Ananda S. Oh S. C. Bodoky G. Shimada Y. (2014). Rainbow: a global, phase 3, double-blind study of ramucirumab plus paclitaxel versus placebo plus paclitaxel in the treatment of gastric cancer following disease progression: Western population subgroup. Ann. Oncol. 25, i106. 10.1093/annonc/mdu193.6

[B89] Wilke H. Muro K. Van Cutsem E. Oh S. C. Bodoky G. Shimada Y. (2014). Ramucirumab plus paclitaxel versus placebo plus paclitaxel in patients with previously treated advanced gastric or gastro-oesophageal junction adenocarcinoma (RAINBOW): a double-blind, randomised phase 3 trial. Lancet Oncol. 11, 1224–1235. 10.1016/S1470-2045(14)70420-6 25240821

[B90] Wu G. Qin X. Q. Guo J. J. Li T. Y. Chen J. H. (2014). AKT/ERK activation is associated with gastric cancer cell resistance to paclitaxel. Int. J. Clin. Exp. Pathol. 7, 1449–1458. 24817940 PMC4014224

[B91] Yamaguchi H. Kitayama J. Ishigami H. Emoto S. Yamashita H. Watanabe T. (2013). A phase 2 trial of intravenous and intraperitoneal paclitaxel combined with S-1 for treatment of gastric cancer with macroscopic peritoneal metastasis. Cancer 18, 3354–3558. 10.1002/cncr.28204 23798046

[B92] Yang J. W. Chen Y. G. Chen Q. Fan N. F. Guo Z. Q. Cai X. C. (2005). A randomized controlled trial of taxol-based combination regimens for advanced gastric cancer. Ai zheng. 12, 1531–1536. 16351808

[B93] Ye S. Rong J. Lin T. Y. Xiao J. Huang Y. Zhai L. Z. (2008). FOLFOX versus PLF regimen in treatment of advanced gastric adenocarcinoma. Nan Fang Yi Ke Da Xue Xue Bao. 9, 1599–1602. 18819876

[B94] Zhang D. Yang R. Wang S. Dong Z. (2014). Paclitaxel: new uses for an old drug. Drug Des. Devel. Ther. 8, 279–284. 10.2147/DDDT.S56801 PMC3934593 24591817

[B95] Zhang J. Xiao Y. Lu M. Zhang X. D. Li Y. Shen L. (2009). Retrospective study on regimens of capecitabine-based chemotherapy in the treatment for advanced gastric cancer. Zhonghua Zhong Liu Za Zhi 4, 312–315. 19615292

[B96] Zhao X. L. Zhang X. Y. Gao J. H. (2016). Clinical efficacy of paclitaxel in the treatment of mid-stage and advanced malignant gastric cancer, and effect of nursing interventions. TROP. J. PHARM. RES. 9, 2035–2039. 10.4314/tjpr.v15i9.31

[B97] Zhou D. Cao G. Cheng Z. Ji H. Fu W. (2015). The clinical efficacy of docetaxel combined with modified FOLFOX regimen in the treatment of advanced gastric cancer. AntiTumor Pharm. 1, 38–41.

[B98] Zhu Y. P. Sheng L. L. Wang L. (2016). Analysis on clinical efficacy of docetaxel combined with cisplatin and irinotecan combined with cisplatin in second-line treatment of patients with advanced gastric cancer. Chin. J. Cancer Prev. Treat. 11, 732–738.

