# Supplementary material for: A Bayesian Network Meta-Analysis for Identifying the Optimal Taxane-Based Chemotherapy Regimens for Treating Gastric Cancer
Source: Front Pharmacol. 2019 Jul 5;10:717. doi: 10.3389/fphar.2019.00717 (PMC6624233; doi:10.3389/fphar.2019.00717)
Supplement: Supplementary file 1 [file Presentation_1.pdf]

Supplementary Material

A Bayesian network meta-analysis for identifying the optimal taxanes-based chemotherapy regimens for treating gastric cancer

Dan Zhang, Jia-Rui Wu, Xiao-Jiao Duan, Kai-Huan Wang, Yi Zhao, Meng-Wei Ni, Shu-Yu Liu, Xiao-Meng Zhang, Bing Zhang

PRISMA checklist for network meta-analysis:

Section/topic	#	Checklist item	Reported on page #
**TITLE**			
Title	1	Identify the report as a systematic review incorporating a network meta-analysis (or related form of meta-analysis).	1
**ABSTRACT**			
Structured summary	2	Provide a structured summary including, as applicable:<br>Background: main objectives<br>Methods: data sources; study eligibility criteria, participants, and interventions; study appraisal; and synthesis methods, such as network meta-analysis.<br>Results: number of studies and participants identified; summary estimates with corresponding confidence/credible intervals; treatment rankings may also be discussed. Authors may choose to summarize pairwise comparisons against a chosen treatment included in their analyses for brevity.<br>Discussion/Conclusions: limitations; conclusions and implications of findings.<br>Other: primary source of funding; systematic review registration number with registry name.	2
**INTRODUCTION**			
Rationale	3	Describe the rationale for the review in the context of what is already known, including mention of why a network meta-analysis has been conducted	2-3
Objectives	4	Provide an explicit statement of questions being addressed with reference to participants, interventions, comparisons, outcomes, and study design (PICOS).	2-3
**METHODS**			
Protocol and registration	5	Indicate if a review protocol exists and if and where it can be accessed (e.g., Web address), and, if available, provide registration information including registration number.	/

Eligibility criteria	6	Specify study characteristics (e.g., PICOS, length of follow-up) and report characteristics (e.g., years considered, language, publication status) used as criteria for eligibility, giving rationale. Clearly describe eligible treatments included in the treatment network, and note whether any have been clustered or merged into the same node (with justification).	3
Information sources	7	Describe all information sources (e.g., databases with dates of coverage, contact with study authors to identify additional studies) in the search and date last searched.	2-3
Search	8	Present full electronic search strategy for at least one database, including any limits used, such that it could be repeated.	2-3
Study selection	9	State the process for selecting studies (i.e., screening, eligibility, included in systematic review, and, if applicable, included in the meta-analysis).	4-5
Data collection process	10	Describe method of data extraction from reports (e.g., piloted forms, independently, in duplicate) and any processes for obtaining and confirming data from investigators.	4-5
Data items	11	List and define all variables for which data were sought (e.g., PICOS, funding sources) and any assumptions and simplifications made.	5
Geometry of the network	12	Describe methods used to explore the geometry of the treatment network under study and potential biases related to it. This should include how the evidence base has been graphically summarized for presentation, and what characteristics were compiled and used to describe the evidence base to readers	5
Risk of bias within individual studies	13	Describe methods used for assessing risk of bias of individual studies (including specification of whether this was done at the study or outcome level), and how this information is to be used in any data synthesis.	5
Summary measures	14	State the principal summary measures (e.g., risk ratio, difference in means). Also describe the use of additional summary measures assessed, such as treatment rankings and surface under the cumulative ranking curve (SUCRA) values, as well as modified approaches used to present summary findings from meta-analyses.	5
Planned methods of analysis	15	Describe the methods of handling data and combining results of studies for each network meta-analysis. This should include, but not be limited to: Handling of multigroup trials; Selection of variance structure; Selection of prior distributions in Bayesian analyses; and Assessment of model fit.	5
Assessment of inconsistency	16	Describe the statistical methods used to evaluate the agreement of direct and indirect evidence in the treatment network(s) studied. Describe efforts taken to address its presence when found.	5
Risk of bias across studies	17	Specify any assessment of risk of bias that may affect the cumulative evidence (e.g., publication bias, selective reporting within studies)	5
Additional analyses	18	Describe methods of additional analyses if done, indicating which were prespecified. This may include, but not be limited to, the following: Sensitivity or subgroup	5

		analyses; Meta-regression analyses; Alternative formulations of the treatment network; and Use of alternative prior distributions for Bayesian analyses (if applicable).	
**RESULTS**			
Study selection	19	Give numbers of studies screened, assessed for eligibility, and included in the review, with reasons for exclusions at each stage, ideally with a flow diagram.	6
Presentation of network structure	20	Provide a network graph of the included studies to enable visualization of the geometry of the treatment network.	6
Summary of network geometry	21	Provide a brief overview of characteristics of the treatment network. This may include commentary on the abundance of trials and randomized patients for the different interventions and pairwise comparisons in the network, gaps of evidence in the treatment network, and potential biases reflected by the network structure.	6
Study characteristics	22	For each study, present characteristics for which data were extracted (e.g., study size, PICOS, follow-up period) and provide the citations.	6
Risk of bias within studies	23	Present data on risk of bias of each study and, if available, any outcome level assessment.	6
Results of individual studies	24	For all outcomes considered (benefits or harms), present, for each study: 1) simple summary data for each intervention group, and 2) effect estimates and confidence intervals. Modified approaches may be needed to deal with information from larger networks.	6-8
Synthesis of results	25	Present results of each meta-analysis done, including confidence/credible intervals. In larger networks, authors may focus on comparisons versus a particular comparator (e.g., placebo or standard care), with full findings presented in an appendix. League tables and forest plots may be considered to summarize pairwise comparisons. If additional summary measures were explored (such as treatment rankings), these should also be presented.	6-8
Exploration for inconsistency	26	Describe results from investigations of inconsistency. This may include such information as measures of model fit to compare consistency and inconsistency models, P values from statistical tests, or summary of inconsistency estimates from different parts of the treatment network.	9
Risk of bias across studies	27	Present results of any assessment of risk of bias across studies for the evidence base being studied.	9
Results of additional analyses	28	Give results of additional analyses, if done (e.g., sensitivity or subgroup analyses, meta-regression analyses, alternative network geometries studied, alternative choice of prior distributions for Bayesian analyses, and so forth).	8-9
**DISCUSSION**			

Summary of evidence	29	Summarize the main findings, including the strength of evidence for each main outcome; consider their relevance to key groups (e.g., health care providers, researchers, and policymakers).	9-10
Limitations	30	Discuss limitations at study and outcome level (e.g., risk of bias), and at review level (e.g., incomplete retrieval of identified research, reporting bias). Comment on the validity of the assumptions, such as transitivity and consistency. Comment on any concerns regarding network geometry (e.g., avoidance of certain comparisons).	10-11
Conclusions	31	Provide a general interpretation of the results in the context of other evidence, and implications for future research.	11
**FUNDING**			
Funding	32	Describe sources of funding for the systematic review and other support (e.g., supply of data); role of funders for the systematic review. This should also include information regarding whether funding has been received from manufacturers of treatments in the network and/or whether some of the authors are content experts with professional conflicts of interest that could affect use of treatments in the network.	11

Search strategy

1. Search strategy of Pubmed

#1 Stomach Neoplasms [MeSH Terms]

#2 Stomach Neoplasm [Title/Abstract]

#3 Gastric Neoplasms [Title/Abstract]

#4 Gastric Neoplasm [Title/Abstract]

#5 Stomach Cancer* [Title/Abstract]

#6 Stomach Tumor* [Title/Abstract]

#7 Gastric Cancer* [Title/Abstract]

#8 Gastric Tumor* [Title/Abstract]

#9 Gastric Carcinoma [Title/Abstract]

#10 Stomach Carcinoma [Title/Abstract]

#11 #1 OR #2 OR #3 OR #4 OR #5 OR #6 OR #7 OR #8 OR #9 OR #10

#12 Paclitaxel [Title/Abstract]

#13 Anzatax [Title/Abstract]

#14 NSC-125973 [Title/Abstract]

#15 NSC 125973 [Title/Abstract]

#16 NSC125973 [Title/Abstract]

#17 Taxol [Title/Abstract]

#18 Taxol A [Title/Abstract]

#19 Bris Taxol [Title/Abstract]

#20 Paxene [Title/Abstract]

#21 Praxel [Title/Abstract]

#22 7-epi-Taxol [Title/Abstract]

#23 7 epi Taxol [Title/Abstract]

#24 Onxol [Title/Abstract]

#25 Docetaxel [Title/Abstract]

#26 Taxotere [Title/Abstract]

#27 docetaxel hydrate [Title/Abstract]

#28 docetaxel trihydrate [Title/Abstract]

#29 docetaxol [Title/Abstract]

#30 docetaxel anhydrous [Title/Abstract]

#31 N-debenzoyl-N-tert-butoxycarbonyl-10-deacetyltaxol [Title/Abstract]

#32 Taxoltere metro [Title/Abstract]

#33 Taxotere [Title/Abstract]

#34 NSC 628503 [Title/Abstract]

#35 RP 56976 [Title/Abstract]

#36 RP-56976 [Title/Abstract]

#37 #12 OR #13 OR #14 OR #15 OR #16 OR #17 OR #18 OR #19 OR #20 OR #21 OR #22 OR #23 OR #24 OR #25 OR #26 OR #27 OR #28 OR #29 OR #30 OR #31 OR #32 OR #33 OR #34 OR #35 OR #36 OR #37

OR #36

#38 randomized controlled trial[Publication Type]
#39 controlled clinical trial[Publication Type]
#40 randomized[Title/Abstract]
41 placebo[Title/Abstract]
#42 randomly[Title/Abstract]
#43 trial[Title/Abstract]
#44 groups[Title/Abstract]
#45 "drug therapy" [Subheading]
#46 #44 OR #45 OR #46 OR #47 OR #48 OR #49 OR #50 OR #51
#47 animals[MeSH Terms]
#48 humans[MeSH Terms]
#49 #47 NOT #48
#50 #46 NOT #49
#51 #11 AND #37 AND #50

2. Search strategy of Embase
#1 random*
#2 placebo*
#3 doubl*
#4 blind*
#5 singl*
#6 assign*
#7 allocat*
#8 “double-blind procedure”/exp
#9 “randomized controlled trial”/exp
#10 “single-blind procedure”/exp
#11 #1 or #2 or #3 or #4 or #5 or #6 or #7 or #8 or #9 or #10

#12 ' stomach tumor'/exp
#13 gastric neoplasm*
#14 stomach neoplasm*
#15 Stomach Cancer*
#16 Gastric Cancer*
#17 Gastric Carcinoma*
#18 Stomach Carcinoma*
#19 #12 OR #13 OR #14 OR #15 OR #16 OR #17 OR #18

#20 'paclitaxel'/exp
#21 anzatax
#22 'nsc 125973'
#23 taxol
#24 paxene
#25 praxel
#26 '7 epi taxol'
#27 onxol
#28 #20 OR #21 OR #22 OR #23 OR #24 OR #25 OR #26 OR #27

#29 'docetaxel'/exp
#30 docetaxel hydrate
#31 docetaxel trihydrate
#32 docetaxol
#33 docetaxel anhydrous
#34 N-debenzoyl-N-tert-butoxycarbonyl-10-deacetyltaxol
#35 taxoltere metro
#36 taxotere
#37 'nsc 628503l'
#38 'rp 56976'
#39 #29 OR #30 OR #31 OR #32 OR #33 OR #34 OR #35 OR #36 OR #37 OR #38

#40 #28 OR #39
#41 #11 AND #19 AND #40

List of the full-text excluded articles

	Study ID	Excluded reasons	Reference
1	Aapro M 2010	Review	Aapro M, Crawford J, Kamioner D. Prophylaxis of chemotherapy-induced febrile neutropenia with granulocyte colony-stimulating factors: Where are we now? SUPPORT CARE CANCER. 2010 2010-01-01;18(5):529-41.
2	Abbrederis K 2006	Single-arm trial	Abbrederis K, Bassermann F, Schuhmacher C,et al. Erythropoietin-alfa during neoadjuvant chemotherapy for locally advanced esophagogastric adenocarcinoma. Ann Thorac Surg. 2006 Jul;82(1):293-7. doi: 10.1016/j.athoracsur.2006.01.097.
3	Abbrederis K 2008	Population inconformity	Abbrederis K, Lorenzen S, von Weikersthal LF, et al. Weekly docetaxel monotherapy for advanced gastric or esophagogastric junction cancer. Results of a phase II study in elderly patients or patients with impaired performance status. Crit Rev Oncol Hematol. 2008 Apr;66(1):84-90. doi:10.1016/j.critrevonc.2007.12.001.
4	Abdel-Rahman O 2014	Study design inconformity	Abdel-Rahman O, Fouad M. Risk of mucocutaneous toxicities in patients with solid tumors treated with everolimus; A systematic review and meta-analysis. EXPERT REV ANTICANC. 2014 2014-01-01;14(12):1529-36.
5	Abouzaid S 2008	Population inconformity	Abouzaid S, Solimando JDA, Waddell JA. Docetaxel, cisplatin, and fluorouracil (DCF) regimen for head, neck, and advanced gastric cancers. Hospital Pharmacy. 2008 2008-01-01;43(4):266-76.
6	Aharon IB 2012	Single-arm trial	Aharon IB, Purim O, Kundel Y, et al. The combination of docetaxel, cisplatin, and 5-fluorouracil in advanced gastric cancer: A single-institution experience. ANTI-CANCER DRUG. 2012 2012-01-01;23(3):313-20.
7	Ajani JA 1996	Single-arm trial	Ajani JA, Ilson DH, Kelsen DP. Paclitaxel in the treatment of patients with upper gastrointestinal carcinomas. Semin Oncol. 1996 Oct;23(5 Suppl 12):55-8.
8	Ajani JA 1996	Single-arm trial	Ajani JA, Ilson DH, Kelsen DP. Paclitaxel in the treatment of patients with upper gastrointestinal carcinomas. SEMIN ONCOL. 1996 1996-01-01;23(SUPPL. 12):55-8.
9	Ajani JA 1997	Single-arm trial	Ajani JA. Treatment of patients with upper gastrointestinal carcinomas. Semin Oncol. 1997 Dec;24(6 Suppl 19):S19-72-S19-76.
10	Ajani JA 1998	Review	Ajani JA.Chemotherapy for gastric carcinoma: new and old options.Oncology (Williston Park). 1998 Oct;12(10 Suppl 7):44-7.
11	Ajani JA 1998	Single-arm trial	Ajani JA, Fairweather J, Dumas P, et al. Phase II study of Taxol in patients with advanced gastric carcinoma. Cancer J Sci Am. 1998 Jul-Aug;4(4):269-74.

12	Ajani JA 2002	Review	Ajani JA.Docetaxel for gastric and esophageal carcinomas.Oncology (Williston Park). 2002 Jun;16(6 Suppl 6):89-96.
13	Ajani JA 2002	Repeated article	Ajani JA. Docetaxel in combination for advanced gastric cancer. GASTRIC CANCER. 2002 2002-01-01;5(SUPPL. 1):31-4.
14	Ajani JA 2005	Repeated article	Ajani JA, Fodor MB, Tjulandin SA, et al. Phase II multi-institutional randomized trial of docetaxel plus cisplatin with or without fluorouracil in patients with untreated, advanced gastric, or gastroesophageal adenocarcinoma. J CLIN ONCOL. 2005 2005-01-01;23(24):5660-7.
15	Ajani JA 2006	Review	Ajani JA. Chemotherapy for advanced gastric or gastroesophageal cancer: defining the contributions of docetaxel. Expert Opin Pharmacother. 2006 Aug;7(12):1627-31. doi: 10.1517/14656566.7.12.1627.
16	Ajani JA 2006	Review	Ajani JA. The role of docetaxel in gastric cancer. European Journal of Cancer, Supplement. 2006 2006-01-01;4(10):4-9.
17	Ajani JA 2006	Review	Ajani JA. Chemotherapy for advanced gastric or gastroesophageal cancer: Defining the contributions of docetaxel. EXPERT OPIN PHARMACO. 2006 2006-01-01;7(12):1627-31.
18	Ajani JA 2006	Single-arm trial	Ajani JA, Winter K, Okawara GS, et al. Phase II trial of preoperative chemoradiation in patients with localized gastric adenocarcinoma (RTOG 9904): Quality of combined modality therapy and pathologic response. J CLIN ONCOL. 2006 2006-01-01;24(24):3953-8.
19	Ajani JA 2007	Repeated article	Ajani JA, Moiseyenko VM, Tjulandin S,et al. Quality of life with docetaxel plus cisplatin and fluorouracil compared with cisplatin and fluorouracil from a phase III trial for advanced gastric or gastroesophageal adenocarcinoma: the V-325 Study Group. J Clin Oncol. 2007 Aug 1;25(22):3210-6. doi: 10.1200/JCO.2006.08.3956.
20	Ajani JA 2007	Repeated article	Ajani JA, Moiseyenko VM, Tjulandin S, et al. Clinical benefit with docetaxel plus fluorouracil and cisplatin compared with cisplatin and fluorouracil in a phase III trial of advanced gastric or gastroesophageal cancer adenocarcinoma: The V-325 study group. J CLIN ONCOL. 2007 2007-01-01;25(22):3205-9.
21	Ajani JA 2007-2	Repeated article	Ajani JA, Moiseyenko VM, Tjulandin S, et al. Clinical benefit with docetaxel plus fluorouracil and cisplatin compared with cisplatin and fluorouracil in a phase III trial of advanced gastric or gastroesophageal cancer adenocarcinoma: the V-325 Study Group. J Clin Oncol. 2007 Aug 1;25(22):3205-9. doi: 10.1200/JCO.2006.10.4968.

22	Ajani JA 2008	Review	Ajani JA.Optimizing docetaxel chemotherapy in patients with cancer of the gastric and gastroesophageal junction: evolution of the docetaxel, cisplatin, and 5-fluorouracil regimen.Cancer. 2008 Sep 1;113(5):945-55. doi: 10.1002/cncr.23661.
23	Akaza H 2002	Review	Akaza H, Aiba K, Isonishi S, et al. Globalization of anti-cancer therapies. Gan To Kagaku Ryoho. 2002 Feb;29(2):205-14.
24	Al-Batran SE 2008	Single-arm trial	Al-Batran SE, Hartmann JT, Hofheinz R,et al.Biweekly fluorouracil, leucovorin, oxaliplatin, and docetaxel (FLOT) for patients with metastatic adenocarcinoma of the stomach or esophagogastric junction: a phase II trial of the Arbeitsgemeinschaft Internistische Onkologie.Ann Oncol. 2008 Nov;19(11):1882-7. doi: 10.1093/annonc/mdn403. Epub 2008 Jul 31.
25	Al-Batran SE 2012	Single-arm trial	Al-Batran SE, Hozaeel W, Jger E. Combination of trastuzumab and triple FLOT chemotherapy (5-fluorouracil/leucovorin, oxaliplatin, and docetaxel) in patients with her2-positive metastatic gastric cancer: Report of 3 Cases. ONKOLOGIE. 2012 2012-01-01;35(9):505-8.
26	Al-Batran SE 2013	Repeated article	Al-Batran SE, Pauligk C, Homann N, et al. The feasibility of triple-drug chemotherapy combination in older adult patients with oesophagogastric cancer: A randomised trial of the Arbeitsgemeinschaft Internistische Onkologie (FLOT65+). EUR J CANCER. 2013 2013-01-01;49(4):835-42.
27	Al-Batran SE 2016	Repeated article	Al-Batran SE, Van Cutsem E, Oh SC, et al. Quality-of-life and performance status results from the phase III RAINBOWstudy of ramucirumab plus paclitaxel versus placebo plus paclitaxel in patients with previously treated gastric or gastroesophageal junction adenocarcinoma. ANN ONCOL. 2016 2016-01-01;27(4):673-9.
28	Alberts DS 2004	Review	Alberts DS, Muggia FM, Carmichael J, et al. Efficacy and safety of liposomal anthracyclines in Phase I/II clinical trials. SEMIN ONCOL. 2004 2004-01-01;31(SUPPL. 13):53-90.
29	Alcindor T 2013	Single-arm trial	Alcindor T, Ferri LE, Marcus V, et al. Perioperative DCF chemotherapy protocol for patients with gastroesophageal adenocarcinoma: correlation between response to treatment and outcome. Med Oncol. 2013 Mar;30(1):377. doi: 10.1007/s12032-012-0377-7.
30	Al-Fakeeh A 2016	Single-arm trial	Al-Fakeeh A, Ferri L, Mulla N, Doerksen T, Al-Ruzug I, Santos F, Alcindor T. A pilot trial of FLOT neoadjuvant chemotherapy for resectable esophagogastric junction adenocarcinoma.Med Oncol. 2016 Jul;33(7):62. doi:10.1007/s12032-016-0774-4.
31	Andersen M 2010	Single-arm trial	Andersen M, Schønnemann KR, Yilmaz M,et al. Phase I study of docetaxel, oxaliplatin and capecitabine (TEX) as first line therapy to patients with advanced gastro-oesophageal cancer. Acta Oncol. 2010 Nov;49(8):1246-52. doi: 10.3109/02841861003767521.

32	Ando T 2012	Repeated article	Ando T, Hosokawa A, Kajiura S, et al. Efficacy of weekly paclitaxel in patients with advanced gastric cancer refractory to docetaxel-based chemotherapy. Gastric Cancer. 2012 Oct;15(4):427-32. doi: 10.1007/s10120-011-0135-0.
33	Ando T 2012	Repeated article	Ando T, Hosokawa A, Kajiura S, et al. Efficacy of weekly paclitaxel in patients with advanced gastric cancer refractory to docetaxel-based chemotherapy. GASTRIC CANCER. 2012 2012-01-01;15(4):427-32.
34	Añorve BD 2015	Study design inconformity	Añorve BD, Aldaco SF, Pérez PP, et al. Global survival of patients with advanced or metastatic gastric cancer in the last10 years at the Centro Médico Nacional «20 de noviembre, ISSSTE». Gaceta Mexicana de Oncologia. 2015 2015-01-01;14(6):313-8.
35	Anter AH 2013	Single-arm trial	Anter AH, Abdel-Latif RM. The safety and efficacy of fluorouracil, leucovorin, oxaliplatin, and docetaxel (FLOT) combination in the front-line treatment for patients with advanced gastric or gastroesophageal adenocarcinoma: phase II trial. Med Oncol. 2013 Mar;30(1):451. doi: 10.1007/s12032-012-0451-1.
36	Anter AH 2013	Single-arm trial	Anter AH, Abdel-Latif RM. The safety and efficacy of fluorouracil, leucovorin, oxaliplatin, and docetaxel (FLOT) combination in the front-line treatment for patients with advanced gastric or gastroesophageal adenocarcinoma: Phase II trial. MED ONCOL. 2013 2013-01-01;30(1).
37	Aoyama T 2014	Repeated article	Aoyama T, Nishikawa K, Takiguchi N, et al. Double-blind, placebo-controlled, randomized phase II study of TJ-14 (hangeshashinto) for gastric cancer chemotherapy-induced oral mucositis. CANCER CHEMOTH PHARM. 2014 2014-01-01;73(5):1047-54.
38	Aravantinos G 2003	Repeated article	Aravantinos G, Linardou H, Makridaki D, et al. Recombinant human erythropoietin for platinum-based chemotherapy-induced anaemia: A single-centre randomised study. Journal of B.U.ON. 2003 2003-01-01;8(2):127-32.
39	Arigami T 2016	Single-arm trial	Arigami T, Uenosono Y, Ishigami S, et al. A Novel Scoring System Based on Fibrinogen and the Neutrophil-Lymphocyte Ratio as a Predictor of Chemotherapy Response and Prognosis in Patients with Advanced Gastric Cancer. Oncology. 2016;90(4):186-92. doi: 10.1159/000444494.
40	Arigami T 2016	Single-arm trial	Arigami T, Uenosono Y, Ishigami S,et al. A Novel Scoring System Based on Fibrinogen and the Neutrophil-Lymphocyte Ratio as a Predictor of Chemotherapy Response and Prognosis in Patients with Advanced Gastric Cancer. Oncology. 2016;90(4):186-92. doi: 10.1159/000444494.
41	Arkenau HT 2012	Review	Arkenau HT, Saggese M, Lemech C. Advanced gastric cancer: Is there enough evidence to call second-line therapy standard? WORLD J GASTROENTERO. 2012 2012-01-01;18(44):6376-8.

42	Atmaca A 2012	Repeated article	Atmaca A, Werner D, Pauligk C, et al. The prognostic impact of epidermal growth factor receptor in patients with metastatic gastric cancer. BMC CANCER. 2012 2012-01-01;12.
43	Aykan NF 2008	Review	Aykan NF, Idelevich E. The role of UFT in advanced gastric cancer. ANN ONCOL. 2008 2008-01-01;19(6):1045-52.
44	Baba H 2003	Single-arm trial	Baba H, Kakeji Y, Oki E, et al. Chemotherapy for gastric cancer. Gan To Kagaku Ryoho. 2003 Nov;30(12):1881-8.
45	Baba H 2003	Single-arm trial	Baba H, Kakeji Y, Oki E, et al. Chemotherapy for gastric cancer. Gan to kagaku ryoho. Cancer & chemotherapy. 2003 2003-01-01;30(12):1881-8.
46	Baddi L 2003	Single-arm trial	Baddi L, Mulcahy M, Benson IA. Treatment of gastric cancer for survival-strategies in the USA and Japan. Annals of Cancer Research and Therapy. 2003 2003-01-01;11(1-2):15-30.
47	Bae SH 2008	Study design inconformity	Bae SH, Ryoo HM, Kim MK, Lee KH, Sin JI, Hyun MS. Effects of the proteasome inhibitor bortezomib alone, and in combination with chemotherapeutic agents in gastric cancer cell lines. ONCOL REP. 2008 2008-01-01;19(4):1027-32.
48	Baek SK 2012	Review	Baek SK, Kim SY, Jeong JH, Cho KS, Yoon HJ. Second-line chemotherapy for advanced gastric cancer in Korea. GASTRIC CANCER. 2012 2012-01-01;15(4):345-54.
49	Bajetta E 2010	Repeated article	Bajetta E, Floriani I, Di Bartolomeo M, et al. Intergroup Trial of Adjuvant Chemotherapy in Adenocarcinoma of the Stomach (ITACA-S) trial: Comparison of a sequential treatment with irinotecan (CPT-11) plus 5-fluorouracil (5FU)/folinic acid (LV) followed by docetaxel and cisplatin versus a 5-FU/LV regimen as postoperative treatment for radically resected gastric cancer: Tolerability and feasibility of a phase III study. J CLIN ONCOL. 2010 2010-01-01;28(15).
50	Bajetta E 2012	Single-arm trial	Bajetta E, Floriani I, Di Bartolomeo M, et al. Intergroup Trial of Adjuvant Chemotherapy in Adenocarcinoma of the Stomach (ITACA-S) trial: Comparison of a sequential treatment with irinotecan (CPT-11) plus 5-fluorouracil (5-FU)/folinic acid (LV) followed by docetaxel and cisplatin versus a 5-FU/LV regimen as postoperative treatment for radically resected gastric cancer. J CLIN ONCOL. 2012 2012-01-01;30(18).
51	Bajetta E 2014	Repeated article	Bajetta E, Floriani I, Di Bartolomeo M, et al. Randomized trial on adjuvant treatment with FOLFIRI followed by docetaxel and cisplatin versus 5-fluorouracil and folinic acid for radically resected gastric cancer.Ann Oncol. 2014 Jul;25(7):1373-8. doi: 10.1093/annonc/mdu146. Epub 2014 Apr 12.

52	Bajetta E 2014	Interventions inconformity	Bajetta E, Floriani I, Di Bartolomeo M, et al. Randomized trial on adjuvant treatment with FOLFIRI followed by docetaxel and cisplatin versus 5-fluorouracil and folinic acid for radically resected gastric cancer. ANN ONCOL. 2014 2014-01-01;25(7):1373-8.
53	Baker JS 2011	Single-arm trial	Baker JS, Qureshi A, Itri L, Sun W, Mulcahy MF, Ajani JA. Dose-ranging study of tesetaxel, a novel oral taxane, administered as second-line therapy at a flat dose to patients with advanced gastric cancer. J CLIN ONCOL. 2011 2011-01-01;29(15).
54	Bamias A 2010	Repeated article	Bamias A, Karina M, Papakostas P,et al. A randomized phase III study of adjuvant platinum/docetaxel chemotherapy with or without radiation therapy in patients with gastric cancer. Cancer Chemother Pharmacol. 2010 May;65(6):1009-21. doi:10.1007/s00280-010-1256-6.
55	Bamias A 2010	Repeated article	Bamias A, Karina M, Papakostas P, et al. A randomized phase iii study of adjuvant platinum/docetaxel chemotherapy with or without radiation therapy in patients with gastric cancer. CANCER CHEMOTH PHARM. 2010 2010-01-01;65(6):1009-21.
56	Bang Y 2016	Repeated article	Bang Y, Boku N, Chin K, et al. Olaparib in combination with paclitaxel in patients with advanced gastric cancer who have progressed following first-line therapy: Phase III GOLD study. ANN ONCOL. 2016 2016-01-01;27.
57	Bang YJ 2002	Single-arm trial	Bang YJ, Kang WK, Kang YK,et al.Docetaxel 75 mg/m(2) is active and well tolerated in patients with metastatic or recurrent gastric cancer: a phase II trial.Jpn J Clin Oncol. 2002 Jul;32(7):248-54.
58	Bang YJ 2011	Review	Bang YJ. Capecitabine in gastric cancer. EXPERT REV ANTICANC. 2011 2011-01-01;11(12):1791-806.
59	Bang YJ 2013	Single-arm trial	Bang YJ. A randomized, open-label, phase III study of lapatinib in combination with weekly paclitaxel versus weekly paclitaxel alone in the second-line treatment of HER2 amplified advanced gastric cancer (AGC) in Asian population: Tytan study. J CLIN ONCOL. 2013 2013-01-01;31(4).
60	Bang YJ 2015	Repeated article	Bang YJ, Im SA, Lee KW, et al. Randomized, double-blind phase II trial with prospective classification by ATM protein level to evaluate the efficacy and tolerability of olaparib plus paclitaxel in patients with recurrent or metastatic gastric cancer. J CLIN ONCOL. 2015 2015-01-01;33(33):3858-65.
61	Bang YJ 2015	Single-arm trial	Bang YJ, Van Cutsem E, Mansoor W, et al. A randomized, open-label phase II study of AZD4547 (AZD) versus Paclitaxel (P) in previously treated patients with advanced gastric cancer (AGC) with Fibroblast Growth Factor Receptor 2 (FGFR2) polysomy or gene amplification (amp): SHINE study. J CLIN ONCOL. 2015 2015-01-01;33(15).

62	Bapsi CA 2012	Single-arm trial	Bapsi CA, Catalano PJ, Mondschein JK, et al. Phase II trial of paclitaxel/cisplatin followed by surgery and adjuvant radiation therapy and 5-fluorouracil/leucovorin for gastric cancer (ECOG E7296). Gastrointestinal Cancer Research. 2012 2012-01-01;5(6):191-7.
63	Barni S 2014	Review	Barni S, Cascinu S, Zaniboni A. Gastric cancer: Toward a cisplatin-free disease? Journal of Gastrointestinal Oncology. 2014 2014-01-01;5(4):318-22.
64	Bar-Sela G 2003	Single-arm trial	Bar-Sela G, Tsalic M, Gaitini D, Steiner M, Haim N. Paclitaxel, carboplatin, and oral etoposide in advanced gastric adenocarcinoma: association with severe myelotoxicity. Med Oncol. 2003;20(3):291-4.
65	Bauer K 2015	Review	Bauer K, Schroeder M, Porzsolt F, Henne-Bruns D. Comparison of international guidelines on the accompanying therapy for advanced gastric cancer: Reasons for the differences. Journal of Gastric Cancer. 2015 2015-01-01;15(1):10-8.
66	Becker K 2012	Unavailable	Becker K, Reim D, Novotny A, et al. Proposal for a multifactorial prognostic score that accurately classifies 3 groups of gastric carcinoma patients with different outcomes after neoadjuvant chemotherapy and surgery. ANN SURG. 2012 2012-01-01;256(6):1002-7.
67	Becker K 2012	Unavailable	Becker K, Reim D, Novotny A, et al. Proposal for a multifactorial prognostic score that accurately classifies 3 groups of gastric carcinoma patients with different outcomes after neoadjuvant chemotherapy and surgery. ANN SURG. 2012 2012-01-01;256(6):1002-7.
68	Ben Aharon I 2012	Single-arm trial	Ben Aharon I, Purim O, Kundel Y,et al. The combination of docetaxel, cisplatin, and 5-fluorouracil in advanced gastric cancer: a single-institution experience. Anticancer Drugs. 2012 Mar;23(3):313-20. doi: 10.1097/CAD.0b013e32834fd780.
69	Ben-Aharon I 2010	Single-arm trial	Ben-Aharon I, Kundel Y, Brenner R, Purim O, Sulkes A, Brenner B. Using the combination of docetaxel, cisplatin and 5-fluorouracil (DCF) in advanced gastric cancer: Is it 'if' or simply 'how'? ANN ONCOL. 2010 2010-01-01;21:i91.
70	Berglund A 2010	Repeated article	Berglund A, Byström P, Johansson B, et al. An explorative randomised phase II study of sequential chemotherapy in advanced upper gastrointestinal cancer. Med Oncol. 2010 Mar;27(1):65-72. doi: 10.1007/s12032-009-9173-4.
71	Berretta S 2016	Review	Berretta S, Berretta M, Fiorica F, et al. Multimodal approach of advanced gastric cancer: Based therapeutic algorithm. EUR REV MED PHARMACO. 2016 2016-01-01;20(19):4018-31.
72	Bianchini D 2008	Population inconformity	Bianchini D, Wong R. Chemotherapy in advanced gastric cancer. Advances in Gastrointestinal Cancers. 2008 2008-01-01;6(3):8-12.

73	Biernacka R 2015	Single-arm trial	Biernacka R, Lewandowski T. Evaluation of prognostic and predictive factors in second-line treatment with irinotecan based chemotherapy in gastric cancer. ANN ONCOL. 2015 2015-01-01;26:v25.
74	Biffi R 2010	Single-arm trial	Biffi R, Fazio N, Luca F,et al.Surgical outcome after docetaxel-based neoadjuvant chemotherapy in locally-advanced gastric cancer.World J Gastroenterol. 2010 Feb 21;16(7):868-74.
75	Biffi R 2010	Interventions inconformity	Biffi R, Fazio N, Luca F, et al. Surgical outcome after docetaxel-based neoadjuvant chemotherapy in locally-advanced gastric cancer. WORLD J GASTROENTERO. 2010 2010-01-01;16(7):868-74.
76	Bilir C 2013	Single-arm trial	Bilir C, Engin H, Bakkal BH, Ilikhan SU, Malkoç D. Chemotherapy in elderly patients with metastatic gastric cancer; a single Turkish cancer center experience. Medicinski Glasnik. 2013 2013-01-01;10(2):298-303.
77	Bin YH 2016	Repeated article	Bin YH, Zheng HP, Lan D, Hu XH. Oxaliplatin and S-1 plus intraperitoneal infusion of docetaxel Versus DOS for the first-line treatment of patients with advanced gastric cancer accompanied by malignant ascites. Chinese Journal of Cancer Prevention and Treatment. 2016 2016-01-01;23(16):1085-9.
78	Blum M 2011	Review	Blum M, Suzuki A, Ajani JA. A comprehensive review of S-1 in the treatment of advanced gastric adenocarcinoma. FUTURE ONCOL. 2011 2011-01-01;7(6):715-26.
79	Boekhout AH 2011	Review	Boekhout AH, Beijnen JH, Schellens JHM. Trastuzumab. ONCOLOGIST. 2011 2011-01-01;16(6):800-10.
80	Bokemeyer C 1997	Single-arm trial	Bokemeyer C, Hartmann JT, Lampe CS, Clemens MR, Quietzsch D, Forkmann L, Kanz Paclitaxel and weekly 24-hour infusion of 5-fluorouracil/folinic acid in advanced gastric cancer.<br>L. Semin Oncol. 1997 Dec;24(6 Suppl 19):S19-96-S19-100.
81	Bokemeyer C 1997	Single-arm trial	Bokemeyer C, Lampe CS, Clemens MR,et al. A phase II trial of paclitaxel and weekly 24 h infusion of 5-fluorouracil/folinic acid in patients with advanced gastric cancer. Anticancer Drugs. 1997 Apr;8(4):396-9.
82	Boku N 2011	Review	Boku N. Past and present achievements, and future direction of the Gastrointestinal Oncology Study Group (GIOSG), a division of Japan Clinical Oncology Group (JCOG). JPN J CLIN ONCOL. 2011 2011-01-01;41(12):1315-21.
83	Boulikas T 2009	Population inconformity	Boulikas T. Clinical overview on Lipoplatin™: A successful liposomal formulation of cisplatin. EXPERT OPIN INV DRUG. 2009 2009-01-01;18(8):1197-218.
84	Breithaupt K 2010	Single-arm trial	Breithaupt K, Thuss-Patience PC. Metastatic gastric cancer: Does second-line chemotherapy make sense? DEUT MED WOCHENSCHR. 2010 2010-01-01;135(38):1872-6.

85	Brenner B 2004	Single-arm trial	Brenner B, Ilson DH, Minsky BD,et al. Phase I trial of combined-modality therapy for localized esophageal cancer: escalating doses of continuous-infusion paclitaxel with cisplatin and concurrent radiation therapy. J Clin Oncol. 2004 Jan 1;22(1):45-52. doi: 10.1200/JCO.2004.05.039.
86	Bugat R 2003	Single-arm trial	Bugat R. Irinotecan in the treatment of gastric cancer. ANN ONCOL. 2003 2003-01-01;14(SUPPL. 2):i37-40.
87	Canbay E 2014	Single-arm trial	Canbay E, Mizumoto A, Ichinose M, et al. Outcome data of patients with peritoneal carcinomatosis from gastric origin treated by a strategy of bidirectional chemotherapy prior to cytoreductive surgery and hyperthermic intraperitoneal chemotherapy in a single specialized center in Japan. ANN SURG ONCOL. 2014 2014-01-01;21(4):1147-52.
88	Cao ND 2008	Review	Cao ND, Zhao AG, Zhu YJ, Yang JK. Chinese reports on the effect and safety of systemic chemotherapy for advanced gastric cancer. World Chinese Journal of Digestology. 2008 2008-01-01;16(4):436-42.
89	Caparello C 2016	Review	Caparello C, Meijer LL, Garajova I, et al. FOLFIRINOX and translational studies: Towards personalized therapy in pancreatic cancer. WORLD J GASTROENTERO. 2016 2016-01-01;22(31):6987-7005.
90	Caponigro F 2005	Single-arm trial	Caponigro F, Facchini G, Nasti G,et al. Gastric cancer. Treatment of advanced disease and new drugs. Front Biosci. 2005 Sep 1;10:3122-6.
91	Cappetta A 2012	Population inconformity	Cappetta A, Lonardi S, Pastorelli D, Bergamo F, Lombardi G, Zagonel V. Advanced gastric cancer (GC) and cancer of the gastro-oesophageal junction (GEJ): Focus on targeted therapies. Critical Reviews in Oncology/Hematology. 2012 2012-01-01;81(1):38-48.
92	Casadei R 2009	Study design inconformity	Casadei R, Rega D, Pinto C, et al.Treatment of advanced gastric cancer with cetuximab plus chemotherapy followed by surgery. Report of a case.Tumori. 2009 Nov-Dec;95(6):811-4.
93	Casak SJ 2015	Repeated article	Casak SJ(1), Fashoyin-Aje I(2), Lemery SJ(2), et al. FDA Approval Summary: Ramucirumab for Gastric Cancer.Clin Cancer Res. 2015 Aug 1;21(15):3372-6. doi: 10.1158/1078-0432.CCR-15-0600.Epub 2015 Jun 5.
94	Chadha MK 2005	Single-arm trial	Chadha MK, Kuvshinoff BW, Javle MM. Neoadjuvant therapy for gastric cancer. ONCOLOGY-BASEL. 2005 2005-01-01;19(9):1219-27.
95	Chakravarthy A 2011	Single-arm trial	Chakravarthy A, Catalano PJ, Mondschein JK, et al. A phase II trial of neoadjuvant paclitaxel/cisplatin chemotherapy followed by surgery and adjuvant radiation therapy and 5-fluorouracil/leucovorin (FU/LV) for gastric cancer (ECOG E7296). INT J RADIAT ONCOL. 2011 2011-01-01;81(2):S67.

96	Chan MMK 2015	Review	Chan MMK, Sjoquist KM, Zalcberg JR. Clinical utility of ramucirumab in advanced gastric cancer. Biologics: Targets and Therapy. 2015 2015-01-01;9:93-105.
97	Chang HI 2012	Review	Chang HI, Yeh MK. Clinical development of liposome-based drugs: Formulation, characterization, and therapeutic efficacy. INT J NANOMED. 2012 2012-01-01;7:49-60.
98	Chao Y 2006	Single-arm trial	Chao Y(1), Li CP, Chao TY, et al. An open, multi-centre, phase II clinical trial to evaluate the efficacy and safety of paclitaxel, UFT, and leucovorin in patients with advanced gastric cancer.Br J Cancer. 2006 Jul 17;95(2):159-63. Epub 2006 Jun 27.DOI: 10.1038/sj.bjc.6603225
99	Chen JH 2015	Repeated article	Chen JH, Shen WX, Xia JX, Xu RL, Zhu MQ, Xu M. Comparative study between docetaxel, oxaliplatin plus S-1 and DCF regimen as first-line therapy in patients with advanced gastric cancer. Chinese Journal of Cancer Prevention and Treatment. 2015 2015-01-01;22(2):134-7.
100	Chen MH 2016	Single-arm trial	Chen MH, Lin J, Hsiao CF, et al.A Phase II Study of Sequential Capecitabine Plus Oxaliplatin Followed by Docetaxel Plus Capecitabine in Patients With Unresectable Gastric Adenocarcinoma: The TCOG 3211 Clinical Trial.Medicine (Baltimore). 2016 Jan;95(3):e2565. doi: 10.1097/MD.0000000000002565.
101	Chen XL 2013	Review	Chen XL, Chen XZ, Yang C, et al. Docetaxel, cisplatin and fluorouracil (DCF) regimen compared with non-taxane-containing palliative chemotherapy for gastric carcinoma: a systematic review and meta-analysis. PLoS One. 2013 Apr 4;8(4):e60320. doi: 10.1371/journal.pone.0060320. Print 2013.
102	Chen XL 2013	Review	Chen XL, Chen XZ, Yang C, et al. Docetaxel, Cisplatin and Fluorouracil (DCF) Regimen Compared with Non-Taxane-Containing Palliative Chemotherapy for Gastric Carcinoma: A Systematic Review and Meta-Analysis. PLOS ONE. 2013 2013-01-01;8(4).
103	Chen XZ 2008	Unavailable	Chen XZ, Jiang K, Hu JK, et al. Cost-effectiveness analysis of chemotherapy for advanced gastric cancer in China. WORLD J GASTROENTERO. 2008 2008-01-01;14(17):2715-22.
104	Chen Y 2014	Repeated article	Chen Y, Wang XJ, Lin RB, Chen L, Lin G, Guo ZQ. Efficacy of intraperitoneal and intravenous chemotherapy for advanced gastric cancer with peritoneal metastasis. TUMORI. 2014 2014-01-01;100(5):e180-8.
105	Chen Y 2015	Single-arm trial	Chen Y, Tang WF, Lin J, et al. Efficacy of paclitaxel-based doublet regimens combining with intraperitoneal chemotherapy for advanced gastric cancer with peritoneal metastasis. INT J CLIN EXP MED. 2015 2015-01-01;8(10):18620-8.

106	Cherny NI 2015	Review	Cherny NI, Sullivan R, Dafni U, et al. A standardised, generic, validated approach to stratify the magnitude of clinical benefit that can be anticipated from anti-cancer therapies: The European Society for Medical Oncology Magnitude of Clinical Benefit Scale (ESMO-MCBS). ANN ONCOL. 2015 2015-01-01;26(8):1547-73.
107	Chi Y 2011	Single-arm trial	Chi Y, Ren JH, Yang L, et al.Phase II clinical study on the modified DCF regimen for treatment of advanced gastric carcinoma.Chin Med J (Engl). 2011 Oct;124(19):2997-3002.
108	Chiuri VE 2009	Population inconformity	Chiuri VE, Silvestris N, Tinelli A, Lorusso V. Efficacy and safety of the combination of docetaxel (Taxotere®) with targeted therapies in the treatment of solid malignancies. CURR DRUG TARGETS. 2009 2009-01-01;10(10):982-1000.
109	Cho BC 2006	Single-arm trial	Cho BC, Kim JH, Kim CB,et al. Paclitaxel and leucovorin-modulated infusional 5-fluorouracil combination chemotherapy for metastatic gastric cancer. Oncol Rep. 2006 Mar;15(3):621-7.
110	Cho JH 2015	Repeated article	Cho JH, Lim JY, Choi AR, et al. Comparison of surgery plus chemotherapy and palliative chemotherapy alone for advanced gastric cancer with krukenberg tumor. CANCER RES TREAT. 2015 2015-01-01;47(4):697-705.
111	Cho JK 2014	Study design inconformity	Cho JK, Kuh HJ, Song SC. Injectable poly(organophosphazene) hydrogel system for effective paclitaxel and doxorubicin combination therapy. J Drug Target. 2014 Sep;22(8):761-7. doi: 10.3109/1061186X.2014.921923.
112	Choi YH 2012	Single-arm trial	Choi YH, Oh SC, Kim JS, et al. A phase II study of docetaxel and oxaliplatin combination in recurrent gastric cancer patients after fluoropyrimidine and/or cisplatin adjuvant treatment: a Korean Cancer Study Group Protocol ST06-02. Cancer Chemother Pharmacol. 2012 Nov;70(5):665-72. doi:10.1007/s00280-012-1956-1.
113	Chon H 2016	Single-arm trial	Chon H, Kim C, Jung M, et al. Randomized phase II trial comparing 4 doublets as a front-line treatment in Asian patients with recurrent/metastatic gastric. J CLIN ONCOL. 2016 2016-01-01;34.
114	Choong NW 2011	Single-arm trial	Choong NW, Mauer AM, Haraf DC, et al. Long-term outcome of a phase II study of docetaxel-based multimodality chemoradiotherapy for locally advanced carcinoma of the esophagus or gastroesophageal junction. Med Oncol. 2011 Dec;28 Suppl 1:S152-61. doi: 10.1007/s12032-010-9658-1.
115	Chrom P 2015	Single-arm trial	Chrom P, Stec R, Szczylik C. Second-line treatment of advanced gastric cancer: Current options and future perspectives. ANTICANCER RES. 2015 2015-01-01;35(9):4575-84.
116	Chung WH 2009	Study design inconformity	Chung WH, Sung BH, Kim SS, et al. Synergistic interaction between tetra-arsenic oxide and paclitaxel in human cancer cells in vitro. Int J Oncol. 2009 Jun;34(6):1669-79.

117	Coccolini F 2012	Review	Coccolini F, Lotti M, Manfredi R,et al. Ureteral stenting in cytoreductive surgery plus hyperthermic intraperitoneal chemotherapy as a routine procedure: evidence and necessity. Urol Int. 2012;89(3):307-10. doi: 10.1159/000339920.
118	Conde-Herrero V 2011	Single-arm trial	Conde-Herrero V, Soberino J, Martinez-Galan J, et al. Docetaxel plus irinotecan in gastric cancer stage IV.Review of 35 cases of a single hospital. ANN ONCOL. 2011 2011-01-01;22:v50.
119	Cortes JE 1995	Review	Cortes JE, Pazdur R. Docetaxel. J CLIN ONCOL. 1995 1995-01-01;13(10):2643-55.
120	Costa JWL 2012	Review	Costa JWL, Coimbra FJF, Ribeiro HSC, et al. Safety and preliminary results of perioperative chemotherapy and hyperthermic intraperitoneal chemotherapy (HIPEC) for high-risk gastric cancer patients. WORLD J SURG ONCOL. 2012 2012-01-01;10.
121	Cronin-Fenton DP 2008	Population inconformity	Cronin-Fenton DP, Mooney MM, Clegg LX, Harlan LC. Treatment and survival in a population-based sample of patients diagnosed with gastroesophageal adenocarcinoma. WORLD J GASTROENTERO. 2008 2008-01-01;14(20):3165-73.
122	Cui HB 2014	Repeated article	Cui HB, Ge HE, Bai XY, et al. Effect of neoadjuvant chemotherapy combined with hyperthermic intraperitoneal perfusion chemotherapy on advanced gastric cancer. EXP THER MED. 2014 2014-01-01;7(5):1083-8.
123	Cui Y 2013	Single-arm trial	Cui Y, Li Q, Yu Y, et al. Combination of low-dose docetaxel and standard-dose S-1 for the treatment of advanced gastric cancer: Efficacy, toxicity, and potential predictive factor. CANCER CHEMOTH PHARM. 2013 2013-01-01;71(1):145-52.
124	Dai X 2017	Repeated article	Dai X, Zhang X, Wang C, Jiang J, Wu C. Paclitaxel/oxaliplatin/fluorouracil (TOF) regimen versus S-1/ oxaliplatin (SOX) regimen for metastatic gastric cancer patients. ONCOTARGET. 2017 2017-01-01;8(18):30495-501.
125	Dalla CM 2010	Single-arm trial	Dalla CM, Poli R, Tomasello G, et al. Dose-dense chemotherapy (CT) with modified dose-dense TCF regimen (TCF-dd) in metastatic gastric cancer (MGC): Update of a phase II study. J CLIN ONCOL. 2010 2010-01-01;28(15).
126	Dan S 2013	Single-arm trial	Dan S, Bai L, Li-Jie W, Ting Z, Zhi-Yuan M. Pharmacogenetic angiogenesis profiling for first-line chemotherapy in patients with advanced gastric cancer. CANCER INVEST. 2013 2013-01-01;31(6):390-6.
127	Das P 2006	Population inconformity	Das P, Fukami N, Ajani JA. Combined modality therapy of localized gastric and esophageal cancers. JNCCN Journal of the National Comprehensive Cancer Network. 2006 2006-01-01;4(4):375-82.
128	Davidson M 2016	Review	Davidson M, Smyth EC, Cunningham D. Clinical role of ramucirumab alone or in combination with paclitaxel for gastric and gastro-esophageal junction adenocarcinoma. ONCOTARGETS THER. 2016 2016-01-01;9:4539-48.

129	De Vita F 2010	Single-arm trial	De Vita F, Silvestris N, Romano MP, et al. Epirubicin (E) in combination with cisplatin (CDDP) and capecitabine (C) versus docetaxel (D) combined with 5-fluorouracil (5-fu) by continuous infusion (C.I.) as front-line therapy in patients with advanced gastric cancer (AGC): Preliminary results of a randomised phase ii trial of the gruppo oncologico dell'italia meridionale. ANN ONCOL. 2010 2010-01-01;21:i227.
130	Deeks ED 2007	Repeated article	Deeks ED, Scott LJ. Docetaxel: in gastric cancer. Drugs. 2007;67(13):1893-901. PMID: 17722957
131	Deeks ED 2007	Repeated article	Deeks ED, Scott LJ. Docetaxel in gastric cancer. DRUGS. 2007 2007-01-01;67(13):1893-901.
132	Deng T 2013	Repeated article	Deng T, Xu N, Xiong JP, et al. Safety analysis of weekly paclitaxel plus S-1 versus paclitaxel plus 5-fluorouracil/calcium folinate as first-line therapy in advanced gastric cancer: a multicenter open random phase II trial. J Chemother. 2013 Feb;25(1):56-9. doi: 10.1179/1973947812Y.0000000057.
133	Deng W 2014	Repeated article	Deng W, Wang QW, Zhang XT, et al. Retrospective analysis of adjuvant chemotherapy for curatively resected gastric cancer. WORLD J GASTROENTERO. 2014 2014-01-01;20(12):3356-63.
134	Di Bartolomeo M 2006	Repeated article	Di Bartolomeo M, Buzzoni R, Mariani L, et al. Feasibility of sequential therapy with FOLFIRI followed by docetaxel/cisplatin inpatients with radically resected gastric adenocarcinoma. A randomized phase III trial. Oncology. 2006;71(5-6):341-6. doi: 10.1159/000108575.
135	Di Bartolomeo M 2016	Single-arm trial	Di Bartolomeo M, Pietrantonio F, Rulli E, et al. Impact on survival of timing and duration of adjuvant chemotherapy in radically resected gastric cancer. TUMORI. 2016 2016-01-01;102(4):e15-9.
136	Di Bartolomeo M 2016	Single-arm trial	Di Bartolomeo M, Niger M, Laterza MM, et al. Safety and efficacy profile of ramucirumab alone or combined with paclitaxel in metastatic gastric cancer (MGC): A real-life overview of compassionate-use named patients (pts) (RAMoss study). ANN ONCOL. 2016 2016-01-01;27.
137	Di Cosimo S 2003	Review	Di Cosimo S, Ferretti G, Fazio N, et al. Docetaxel in advanced gastric cancer--review of the main clinical trials. Acta Oncol. 2003;42(7):693-700.

138	Di Cosimo S 2003	Single-arm trial	Di Cosimo S, Ferretti G, Fazio N, et al. Docetaxel in Advanced Gastric Cancer: Review of the Main Clinical Trials. ACTA ONCOL. 2003 2003-01-01;42(7):693-700.
139	Di Lauro L 2005	Single-arm trial	Di Lauro L, Belli F, Arena MG,Epirubicin, cisplatin and docetaxel combination therapy for metastatic gastric cancer.Ann Oncol. 2005 Sep;16(9):1498-502. Epub 2005 Jun 14.
140	Di Lauro L 2005	Single-arm trial	Di Lauro L, Belli F, Arena MG, et al. Epirubicin, cisplatin and docetaxel combination therapy for metastatic gastric cancer. ANN ONCOL. 2005 2005-01-01;16(9):1498-502.
141	Di Lauro L 2013	Single-arm trial	Di Lauro L, Sergi D, Belli F, et al. Docetaxel, oxaliplatin, and capecitabine (DOX) combination chemotherapy for metastatic gastric or gastroesophageal junction (GEJ) adenocarcinoma. J CLIN ONCOL. 2013 2013-01-01;31(15).
142	Di Maio M 2013	Population inconformity	Di Maio M, Bria E, Banna GL, et al. Prevention of chemotherapy-induced nausea and vomiting and the role of neurokinin 1 inhibitors: from guidelines to clinical practice in solid tumors. ANTI-CANCER DRUG. 2013 2013-01-01;24(2):99-111.
143	Dikken JL 2012	Review	Dikken JL, Van De Velde CJH, Coit DG, Shah MA, Verheij M, Cats A. Treatment of resectable gastric cancer. Therapeutic Advances in Gastroenterology. 2012 2012-01-01;5(1):49-69.
144	Dirican A 2013	Single-arm trial	Dirican A, Kucukzeybek Y, Tarhan MO, et al. One-day DCF regimen in patients with metastatic gastric cancer. Tumori. 2013 Mar-Apr;99(2):145-8. doi: 10.1700/1283.14183.
145	Dirican A 2013	Single-arm trial	Dirican A, Kucukzeybek Y, Tarhan MO, et al. One-day DCF regimen in patients with metastatic gastric cancer. TUMORI. 2013 2013-01-01;99(2):145-8.
146	Doi T 2016	Single-arm trial	Doi T, Ishikawa T, Okayama T, et al. Outcome and Problems of Nab-Paclitaxel Therapy for Gastric Cancer in Clinical Practice. Gan To Kagaku Ryoho. 2016 Feb;43(2):211-4.
147	Doi T 2016	Study design inconformity	Doi T, Ishikawa T, Okayama T,et al. Outcome and Problems of Nab-Paclitaxel Therapy for Gastric Cancer in Clinical Practice. Gan To Kagaku Ryoho. 2016 Feb;43(2):211-4.
148	Dong L 2014	Repeated article	Dong L, Li J, Lou XP, et al. Comparison of short-term efficacy and safety of TIROX and DCF regimens for advanced gastric cancer. J Int Med Res. 2014 Jun;42(3):737-43. doi: 10.1177/0300060513510657.
149	Du N 2014	Single-arm trial	Du N., Ma J, Wang S, et al. The R0 resection rate after neoadjuvant bevacizumab (Bev) plus DOF versus DOF in local advanced gastric carcinoma (LAGC) and its association with circulating tumor cell (CTC). J CLIN ONCOL. 2014 2014-01-01;32(15).
150	Du Y 2015	Single-arm trial	Du Y., Yu P. Conversion chemotherapy combined with surgical treatment of unresectable advanced gastric cancer. J CLIN ONCOL. 2015 2015-01-01;33(3).

151	Einzig AI 1996	Single-arm trial	Einzig AI, Neuberg D, Remick SC,et al. Phase II trial of docetaxel (Taxotere) in patients with adenocarcinoma of the upper gastrointestinal tract previously untreated with cytotoxic chemotherapy: the Eastern Cooperative Oncology Group (ECOG) results of protocol E1293. Med Oncol. 1996 Jun;13(2):87-93.
152	Elimova E 2015	Single-arm trial	Elimova E, Ajani JA. Time-to-treatment failure as the primary end point of a first-line advanced gastric cancer randomized trial: How confused would you want us to be? J CLIN ONCOL. 2015 2015-01-01;33(21):2410.
153	El-Rayes BF 2010	Population inconformity	El-Rayes BF, Zalupski M, Bekai-Saab T, et al.A phase II study of bevacizumab, oxaliplatin, and docetaxel in locally advanced and metastatic gastric and gastroesophageal junction cancers.Ann Oncol. 2010 Oct;21(10):1999-2004. doi: 10.1093/annonc/mdq065.
154	El-Rayes BF 2010	Single-arm trial	El-Rayes BF, Zalupski M, Bekai-Saab T, et al. A phase II study of bevacizumab, oxaliplatin, and docetaxel in locally advanced and metastatic gastric and gastroesophageal junction cancers. ANN ONCOL. 2010 2010-01-01;21(10):1999-2004.
155	Emi Y 2004	Single-arm trial	Emi Y, Kakeji Y, Baba H, Ishida T, Maehara Y. Current combination chemotherapy containing paclitaxel for advanced, recurrent gastric cancer. Gan to kagaku ryoho. Cancer & chemotherapy. 2004 2004-01-01;31(12):1978-81.
156	Emi Y 2008	Single-arm trial	Emi Y, Yamamoto M, Takahashi I,et al. Phase II study of weekly paclitaxel by one-hour infusion for advanced gastric cancer. Surg Today. 2008;38(11):1013-20. doi: 10.1007/s00595-008-3769-8.
157	Emi Y 2008	Single-arm trial	Emi Y, Yamamoto M, Takahashi I, et al. Phase II study of weekly paclitaxel by one-hour infusion for advanced gastric cancer. SURG TODAY. 2008 2008-01-01;38(11):1013-20.
158	Emoto S 2012	Study design inconformity	Emoto S, Yamaguchi H, Kishikawa J, et al.Antitumor effect and pharmacokinetics of intraperitoneal NK105, a nanomicellar paclitaxel formulation for peritoneal dissemination.Cancer Sci. 2012 Jul;103(7):1304-10. doi: 10.1111/j.1349-7006.2012.02274.x.
159	Engin H 2012	Study design inconformity	Engin H, Bilir C. Chemotherapy in elderly patients with metastatic gastric cancer; a single Turkish cancer center experience. ANN ONCOL. 2013 2013-01-01;24:v60.
160	Enzinger PC 2009	Single-arm trial	Enzinger PC, Ryan DP, Clark JW,et al.Weekly docetaxel, cisplatin, and irinotecan (TPC): results of a multicenter phase II trial in patients with metastatic esophagogastric cancer.Ann Oncol. 2009 Mar;20(3):475-80. doi: 10.1093/annonc/mdn658.
161	Eren T 2016	Single-arm trial	Eren T, Yildirim NO, Yazici O, et al. The real life outcomes of modified docetaxel and cisplatin plus fluorourasil regimen in patients with metastatic gastric cancer. J CLIN ONCOL. 2016 2016-01-01;34.
162	Evans D 2007	Single-arm trial	Evans D, Miner T, Iannitti D, et al. Docetaxel, capecitabine and carboplatin in metastatic esophagogastric cancer: a phase II study. Cancer Invest. 2007 Sep;25(6):445-8.doi: 10.1080/07357900701358025.

163	Fantini M 2015	Single-arm trial	Fantini M, Stocchi L, Nicoletti S, et al. Second-line treatments in metastatic gastric cancer: Pooled analysis of randomized clinical trials. J CLIN ONCOL. 2015 2015-01-01;33(15).
164	Fazio N 2016	Repeated article	Fazio N, Biffi R, Maibach R, et al. Preoperative versus postoperative docetaxel-cisplatin-fluorouracil (TCF) chemotherapy in locally advanced resectable gastric carcinoma: 10-year follow-up of the SAKK 43/99 phase III trial. Ann Oncol. 2016 Apr;27(4):668-73. doi: 10.1093/annonc/mdv620.
165	Ferri LE 2012	Population inconformity	Ferri LE, Ades S, Alcindor T, et al.Perioperative docetaxel, cisplatin, and 5-fluorouracil (DCF) for locally advanced esophageal and gastric adenocarcinoma: a multicenter phase II trial.Ann Oncol. 2012 Jun;23(6):1512-7. doi: 10.1093/annonc/mdr465.
166	Fiteni F 2016	Repeated article	Fiteni F, Paget-Bailly S, Messager M, et al. Docetaxel, Cisplatin, and 5-Fluorouracil as perioperative chemotherapy compared with surgery alone for resectable gastroesophageal adenocarcinoma. Cancer Medicine. 2016 2016-01-01;5(11):3085-93.
167	Fonseca PJ 2010	Single-arm trial	Fonseca PJ, Vié IJM, Frunza M, et al. A phase II study with capecitabine and cisplatin as first-line therapy in advanced gastric cancer. ANN ONCOL. 2010 2010-01-01;21:i240.
168	Font A 2008	Single-arm trial	Font A, Salazar R, Maurel J, et al. Cisplatin plus weekly CPT-11/docetaxel in advanced esophagogastric cancer: a phase I study with pharmacogenetic assessment of XPD, XRCC3 and UGT1A1 polymorphisms. Cancer Chemother Pharmacol. 2008 Nov;62(6):1075-83. doi:10.1007/s00280-008-0700-3.
169	Foo M 2014	Review	Foo M, Leong T. Adjuvant therapy for gastric cancer: Current and future directions. WORLD J GASTROENTERO. 2014 2014-01-01;20(38):13718-27.
170	Ford HE 2014	Population inconformity	Ford HE, Marshall A, Bridgewater JA,et al. Docetaxel versus active symptom control for refractory oesophagogastric adenocarcinoma (COUGAR-02): an open-label, phase 3 randomised controlled trial.Lancet Oncol. 2014 Jan;15(1):78-86. doi: 10.1016/S1470-2045(13)70549-7. Epub 2013 Dec 10.
171	Fuchs CS 2015	Repeated article	Fuchs CS, Muro K, Tomasek J, et al. Prognostic factor analysis of overall survival (OS) in gastric cancer from two phase III studies of second-line ramucirumab (RAM) (REGARD and RAINBOW) using pooled individual patient (pt) data. J CLIN ONCOL. 2015 2015-01-01;33(15).
172	Fujii M 2008	Study design inconformity	Fujii M. Chemotherapy for advanced gastric cancer: ongoing phase III study of S-1 alone versus S-1 and docetaxel combination (JACCRO GC03 study). Int J Clin Oncol. 2008 Jun;13(3):201-5. doi: 10.1007/s10147-008-0775-1.

173	Fujii M 2008	Repeated article	Fujii M. Chemotherapy for advanced gastric cancer: Ongoing phase III study of S-1 alone versus S-1 and docetaxel combination (JACCRO GC03 study). INT J CLIN ONCOL. 2008 2008-01-01;13(3):201-5.
174	Fujii M 2010	Review	Fujii M, Kochi M, Takayama T. Recent advances in chemotherapy for advanced gastric cancer in Japan. SURG TODAY. 2010 2010-01-01;40(4):295-300.
175	Fujii M 2011	Repeated article	Fujii M, Kim YH, Satoh T, et al. Randomized phase III study of S-1 alone versus S-1 plus docetaxel (DOC) in the treatment for advanced gastric cancer (AGC): The START trial update. J CLIN ONCOL. 2011 2011-01-01;29(15).
176	Fujitani K 2010	Single-arm trial	Fujitani K, Takiuchi H, Sugimoto N, et al. Randomized phase II trial of S-1 plus irinotecan versus S-1 plus paclitaxel as first-line treatment for advanced gastric cancer (OGSG0402). ANN ONCOL. 2010 2010-01-01;21:i256.
177	Fujitani K 2013	Single-arm trial	Fujitani K, Takiuchi H, Sugimoto N, et al. Impact of pretreatment systemic inflammatory response on survival in AGC patients receiving first-line chemotherapy. J CLIN ONCOL. 2013 2013-01-01;31(4).
178	Fujiwara Y 2007	Review	Fujiwara Y, Doki Y, Takiguchi S,et al. Neoadjuvant chemotherapy for gastric cancer with peritoneal dissemination. Gan To Kagaku Ryoho. 2007 Nov;34(12):1923-5.
179	Fujiwara Y 2008	Review	Fujiwara Y, Takiguchi S, Nakajima K,et al. Clinical trials of neo-adjuvant chemotherapy for advanced gastric cancer with peritoneal dissemination. Gan To Kagaku Ryoho. 2008 Nov;35(12):1996-8.
180	Fumoleau P 1995	Single-arm trial	Fumoleau P, Perroucheau G, Maugard-Louboutin C, Lemevel B. Paclitaxel (Taxol®) and docetaxel (Taxotere®) as single agents: Results of phase II trials. B CANCER. 1995 1995-01-01;82(8):629-36.
181	Furukawa K 2005	Single-arm trial	Furukawa K, Watanabe K, Abe Y, et al. Usefulness of the outpatient chemotherapy regimen of S-1 followed by weekly paclitaxel for far advanced gastric cancer. Acta Medica et Biologica. 2005 2005-01-01;53(3):73-7.
182	Fushida S 2002	Single-arm trial	Fushida S, Nao F, Kinami S, et al. Pharmacologic study of intraperitoneal docetaxel in gastric cancer patients with peritoneal dissemination. Gan To Kagaku Ryoho. 2002 Oct;29(10):1759-63.
183	Fushida S 2002	Single-arm trial	Fushida S, Furui N, Kinami S,et al. Pharmacologic study of intraperitoneal paclitaxel in gastric cancer patients with peritoneal dissemination. Gan To Kagaku Ryoho. 2002 Nov;29(12):2164-7.
184	Fushida S 2012	Single-arm trial	Fushida S, Nashimoto A, Fukushima N,et al.Phase II trial of preoperative chemotherapy with docetaxel, cisplatin and S-1 for T4 locally advanced gastric cancer.Jpn J Clin Oncol. 2012 Feb;42(2):131-3. doi: 10.1093/jjco/hyr183. Epub 2011 Dec 13.
185	Fushida S 2013	Single-arm trial	Fushida S, Kinoshita J, Kaji M,et al.Phase I/II study of intraperitoneal docetaxel plus S-1 for the gastric cancer patients with peritoneal carcinomatosis.Cancer Chemother Pharmacol. 2013 May;71(5):1265-72. doi: 10.1007/s00280-013-2122-0.

186	Fushida S 2015	Single-arm trial	Fushida S, Kaji M, Oyama K, et al. Randomized Phase II trial of paclitaxel plus valproic acid vs paclitaxel alone as second-line therapy for patients with advanced gastric cancer. ONCOTARGETS THER. 2015 2015-01-01;8:939-41.
187	Fushida S 2016	Repeated article	Fushida S, Kinoshita J, Kaji M, et al. Paclitaxel plus valproic acid versus paclitaxel alone as second- or third-line therapy for advanced gastric cancer: a randomized Phase II trial.Drug Des Devel Ther. 2016 Jul 25;10:2353-8. doi: 10.2147/DDDT.S110425.
188	Gadgeel SM 2003	Single-arm trial	Gadgeel SM, Shields AF, Heilbrun LK, et al. Phase II study of paclitaxel and carboplatin in patients with advanced gastric cancer. American Journal of Clinical Oncology: Cancer Clinical Trials. 2003 2003-01-01;26(1):37-41.
189	Galdy S 2016	Review	Galdy S, Cella CA, Spada F, et al. Systemic therapy beyond first-line in advanced gastric cancer: An overview of the main randomized clinical trials. Crit Rev Oncol Hematol. 2016 Mar;99:1-12. doi: 10.1016/j.critrevonc.2015.09.004.
190	Garcia AA 2001	Single-arm trial	Garcia AA, Leichman CG, Lenz HJ,et al.Phase II trial of outpatient schedule of paclitaxel in patients with previously untreated metastatic, measurable adenocarcinoma of the stomach.Jpn J Clin Oncol. 2001 Jun;31(6):275-8.
191	Garrido M 2016	Population inconformity	Garrido M. The safety and efficacy of ramucirumab in combination with paclitaxel for the treatment of advanced gastric or gastro-esophageal junction adenocarcinoma. EXPERT REV ANTICANC. 2016 2016-01-01;16(10):1005-10.
192	Gemici C 2013	Single-arm trial	Gemici C. Cost minimization analysis of docetaxel + cisplatin + 5-fu vs capecitabine + cisplatin in patients with advanced gastric cancer. ANN ONCOL. 2013 2013-01-01;24:v62.
193	Ghosn M 2016	Review	Ghosn M, Tabchi S, Kourie HR, Tehfe M. Metastatic gastric cancer treatment: Second line and beyond. WORLD J GASTROENTERO. 2016 2016-01-01;22(11):3069-77.
194	Giordano KF 2006	Single-arm trial	Giordano KF, Jatoi A, Stella PJ, et al. Docetaxel and capecitabine in patients with metastatic adenocarcinoma of the stomach and gastroesophageal junction: a phase II study from the North Central Cancer Treatment Group.Ann Oncol. 2006 Apr;17(4):652-6.
195	Giuliani F 2003	Repeated article	Giuliani F, Gebbia V, De Vita F, et al. Docetaxel as Salvage Therapy in Advanced Gastric Cancer: A Phase II Study of the Gruppo Oncologico Italia Meridionale (G.O.I.M.). ANTICANCER RES. 2003 2003-01-01;23(5 B):4219-22.
196	Giuliani F 2008	Single-arm trial	Giuliani F, Romito S, Maiello E, et al. Epirubicin, taxotere and fluorouracil modulated by folinic acid in the treatment of advanced gastric cancer: A phase II study of the Gruppo Oncologico dell' Italia Meridionale (GOIM). European Journal of Cancer, Supplement. 2008 2008-01-01;6(14):107-12.
197	Goekkurt E 2009	Study design inconformity	Goekkurt E(1), Al-Batran SE, Mogck U,et al. Pharmacogenetic analyses of hematotoxicity in advanced gastric cancer patients receiving biweekly fluorouracil, leucovorin, oxaliplatin and docetaxel (FLOT): a translational study of the Arbeitsgemeinschaft Internistische Onkologie (AIO).Ann Oncol. 2009 Mar;20(3):481-5. doi: 10.1093/annonc/mdn667. Epub 2008 Dec 12.

198	Goekkurt E 2010	Single-arm trial	Goekkurt E, Al-Batran S, Kramer M, et al. Toxicogenomic profiling of fluorouracil/leucovorin/oxaliplatin/docetaxel (FLOT) in advanced gastric cancer: A translational study of the German Arbeitsgemeinschaft Internistische Onkologie (AIO). J CLIN ONCOL. 2010 2010-01-01;28(15).
199	Goetze TO 2017	Repeated article	Goetze TO, Hofheinz RD, Bechstein WO, et al. Improvement of histopathological regression after neoadjuvant chemotherapy with FLOT versus ECF in patients with resectable gastric or gastroesophageal junction adenocarcinoma: Results of the phase II part of the randomized phase II/III FLOT4 trial. EUR SURG RES. 2017 2017-01-01;58:8.
200	Gold JS 2007	Single-arm trial	Gold JS, Jaques DP, Bentrem DJ, et al. Outcome of patients with known metastatic gastric cancer undergoing resection with therapeutic intent. ANN SURG ONCOL. 2007 2007-01-01;14(2):365-72.
201	Gómez-Martin C 2012	Repeated article	Gómez-Martin C, Sánchez A, Irigoyen A, et al. Incidence of hand-foot syndrome with capecitabine in combination with chemotherapy as first-line treatment in patients with advanced and/or metastatic gastric cancer suitable for treatment with a fluoropyrimidine-based regimen. Clinical and Translational Oncology. 2012 2012-01-01;14(9):689-97.
202	Gong JF 2014	Single-arm trial	Gong JF, Lu M, Li J,et al. Efficacy of albumin-bound paclitaxel in advanced gastric cancer patients.Beijing Da Xue Xue Bao. 2014 Feb 18;46(1):144-8.
203	Gotoh M 2006	Single-arm trial	Gotoh M, Kawabe S, Takiuchi H. S-1 combined with weekly paclitaxel in patients with advanced gastric cancer. Gan To Kagaku Ryoho. 2006 Jun;33 Suppl 1:91-4.
204	Gotoh M 2006	Single-arm trial	Gotoh M, Kawabe S, Takiuchi H. S-1 combined with weekly paclitaxel in patients with advanced gastric cancer. Gan to kagaku ryoho. Cancer & chemotherapy. 2006 2006-01-01;33 Suppl 1:91-4.
205	Graziano F 2000	Single-arm trial	Graziano F, Catalano V, Baldelli AM,A phase II study of weekly docetaxel as salvage chemotherapy for advanced gastric cancer. Ann Oncol. 2000 Oct;11(10):1263-6.
206	Greig SL 2015	Repeated article	Greig SL, Keating GM. Ramucirumab: A Review in Advanced Gastric Cancer. BioDrugs. 2015 Oct;29(5):341-51. doi: 10.1007/s40259-015-0138-1.
207	Gu J 2004	Review	Gu J, Yamamoto H, Lu X, et al. Low-dose oxaliplatin enhances the antitumor efficacy of paclitaxel in human gastric cancer cell lines. Digestion. 2006;74(1):19-27. doi: 10.1159/000095826.
208	Gu J 2006	Review	Gu J, Yamamoto H, Lu X, et al. Low-dose oxaliplatin enhances the antitumor efficacy of paclitaxel in human gastric cancer cell lines. DIGESTION. 2006 2006-01-01;74(1):19-27.

209	Gubanski M 2010	Repeated article	Gubanski M, Johnsson A, Fernebro E, et al. Randomized phase II study of sequential docetaxel and irinotecan with 5-fluorouracil/folinic acid (leucovorin) in patients with advanced gastric cancer: the GATAC trial. Gastric Cancer. 2010 Aug;13(3):155-61. doi: 10.1007/s10120-010-0553-4.
210	Gubanski M 2014	Repeated article	Gubanski M, Glimelius B, Lind PA. Quality of life in patients with advanced gastric cancer sequentially treated with docetaxel and irinotecan with 5-fluorouracil and folinic acid (leucovin). Med Oncol. 2014 Apr;31(4):906. doi: 10.1007/s12032-014-0906-7.
211	Guerra ED 2016	Single-arm trial	Guerra ED, Campos DE, Alegre DRE, Villanueva JP, Marmesat RB, Márquez FE. Indirect comparison of ramucirumab and alternatives in advanced or metastatic gastric adenocarcinoma. INT J CLIN PHARM-NET. 2016 2016-01-01;38(6):584.
212	Guo M 2015	Repeated article	Guo M, Yu Y, Wang Y,et al. Low-dosed docetaxel showed equivalent efficacy but improved tolerability compared with oxaliplatin in the S-1-based first-line chemotherapy regimen for metastatic or recurrent gastric adenocarcinoma. Med Oncol. 2015 Sep;32(9):230. doi: 10.1007/s12032-015-0675-y.
213	Guo Z 2015	Repeated article	Guo Z, Wang X, Lin R,et al. Paclitaxel-based regimens as first-line treatment in advanced gastric cancer. J Chemother. 2015 Feb;27(2):94-8. doi: 10.1179/1973947814Y.0000000169.
214	Haag GM 2014	Single-arm trial	Haag GM, Apostolidis L, Jaeger D. Efficacy and safety of trastuzumab-based therapy in combination with different chemotherapeutic regimens in advanced esophagogastric cancer - A single cancer-center experience. TUMORI. 2014 2014-01-01;100(3):237-42.
215	Haibo M 2012	Single-arm trial	Haibo M, Fang W, Zheng Y, et al. A randomized phase II trial of biweekly S-1 with paclitaxel (SPA) or oxaliplatin (SOX) as first-line chemotherapy in advanced gastric cancer patients: Preliminary results. ANN ONCOL. 2012 2012-01-01;23:x256.
216	Haj MN 2015	Study design inconformity	Haj MN, ter Veer E, Ngai L, Mali R, van Oijen MGH, van Laarhoven HWM. Optimal first-line chemotherapeutic treatment in patients with locally advanced or metastatic esophagogastric carcinoma: triplet versus doublet chemotherapy: a systematic literature review and meta-analysis. CANCER METAST REV. 2015 2015-01-01;34(3):429-41.
217	Haller DG 2002	Single-arm trial	Haller DG, Misset JL. Docetaxel in advanced gastric cancer. Anticancer Drugs. 2002 Jun;13(5):451-60.
218	Haller DG 2002	Repeated article	Haller DG, Misset JL. Docetaxel in advanced gastric cancer. ANTI-CANCER DRUG. 2002 2002-01-01;13(5):451-60.
219	Han ME 2011	Study design inconformity	Han ME, Jeon TY, Hwang SH, et al. Cancer spheres from gastric cancer patients provide an ideal model system for cancer stem cell research. CELL MOL LIFE SCI. 2011 2011-01-01;68(21):3589-605.

220	Haruki K 2013	Study design inconformity	Haruki K, Shiba H, Fujiwara Y,et al. Inhibition of nuclear factor-κB enhances the antitumor effect of paclitaxel against gastric cancer with peritoneal dissemination in mice. Dig Dis Sci. 2013 Jan;58(1):123-31. doi: 10.1007/s10620-012-2311-4.
221	Hashemzadeh S 2014	Repeated article	Hashemzadeh S, Pourzand A, Somi MH, Zarrintan S, Javad-Rashid R, Esfahani A. The effects of neoadjuvant chemotherapy on resectability of locally-advanced gastric adenocarcinoma: A clinical trial. INT J SURG. 2014 2014-01-01;12(10):1061-9.
222	Hasovits C 2012	Review	Hasovits C, Clarke S. Pharmacokinetics and pharmacodynamics of intraperitoneal cancer chemotherapeutics. CLIN PHARMACOKINET. 2012 2012-01-01;51(4):203-24.
223	Hecht JR 2003	Single-arm trial	Hecht JR, Blanke CD, Benson AB,et al.Irinotecan and paclitaxel in metastatic adenocarcinoma of the esophagus and gastric cardia.Oncology (Williston Park). 2003 Sep;17(9 Suppl 8):13-5.
224	Hejna M 2008	Single-arm trial	Hejna M, Raderer M, Zacherl J, et al. Phase II study of docetaxel in combination with oxaliplatin in patients with metastatic or locally advanced esophagogastric cancer previously untreated with chemotherapy for advanced disease: results of the Central European Cooperative Oncology Group Study ESGAS.1.2.001. Anticancer Drugs. 2008 Jun;19(5):535-9. doi: 10.1097/CAD.0b013e3282fb178a.
225	Hess L 2014	Single-arm trial	Hess L, Mytelka D, Beyrer J, Nicol S. Costs and patterns of gastric cancer care in the US: A retrospective analysis of administrative claims data. ANN ONCOL. 2014 2014-01-01;25:i35.
226	Hideyuki U 2013	Review	Hideyuki U. How can we achieve effective chemotherapy in highly advanced gastric cancer patients? Journal of Tokyo Medical University. 2013 2013-01-01;71(3):255-65.
227	Higashi D 2012	Interventions inconformity	Higashi D, Seki K, Ishibashi Y, et al. The effect of lentinan combination therapy for unresectable advanced gastric cancer. Anticancer Res. 2012 Jun;32(6):2365-8.
228	Higuchi K 2014	Repeated article	Higuchi K, Tanabe S, Shimada K, et al. Biweekly irinotecan plus cisplatin versus irinotecan alone as second-line treatment for advanced gastric cancer: A randomised phase III trial (TCOG GI-0801/BIRIP trial). EUR J CANCER. 2014 2014-01-01;50(8):1437-45.
229	Hirayama Y 2015	Repeated article	Hirayama Y, Ishitani K, Sato Y, et al. Effect of duloxetine in Japanese patients with chemotherapy-induced peripheral neuropathy: a pilot randomized trial. INT J CLIN ONCOL. 2015 2015-01-01;20(5):866-71.
230	Hironaka S 2010	Single-arm trial	Hironaka S, Yamazaki K, Taku K, et al. Phase I study of docetaxel, cisplatin and S-1 in patients with advanced gastric cancer. JPN J CLIN ONCOL. 2010 2010-01-01;40(11):1014-20.

231	Hironaka S 2013	Repeated article	Hironaka S, Ueda S, Yasui H, et al. Randomized, open-label, phase III study comparing irinotecan with paclitaxel in patients with advanced gastric cancer without severe peritoneal metastasis after failure of prior combination chemotherapy using fluoropyrimidine plus platinum: WJOG 4007 trial. J Clin Oncol. 2013 Dec 10;31(35):4438-44. doi: 10.1200/JCO.2012.48.5805.
232	Hironaka S 2013	Repeated article	Hironaka S, Ueda S, Yasui H, et al. Randomized, open-label, phase III study comparing irinotecan with paclitaxel in patients with advanced gastric cancer without severe peritoneal metastasis after failure of prior combination chemotherapy using fluoropyrimidine plus platinum: WJOG 4007 trial. J CLIN ONCOL. 2013 2013-01-01;31(35):4438-44.
233	Hironaka S 2014	Single-arm trial	Hironaka S, Shimada Y, Sugimoto N, et al. RAINBOW: A global, phase III, randomized, double-blind study of ramucirumab (RAM) plus paclitaxel (PTX) versus placebo (PL) plus PTX in the treatment of metastatic gastroesophageal junction and gastric adenocarcinoma (mGC) following disease progression on first-line platinum- and fluoropyrimidine-containing combination therapy-Efficacy analysis in Japanese and Western patients. J CLIN ONCOL. 2014 2014-01-01;32(15).
234	Hironaka S 2016	Repeated article	Hironaka S, Sugimoto N, Yamaguchi K, et al. S-1 plus leucovorin versus S-1 plus leucovorin and oxaliplatin versus S-1 plus cisplatin in patients with advanced gastric cancer: A randomised, multicentre, open-label, phase 2 trial. The Lancet Oncology. 2016 2016-01-01;17(1):99-108.
235	Hisashige A 2013	Single-arm trial	Hisashige A, Sasako M, Nakajima T. Cost-effectiveness of adjuvant chemotherapy for curatively resected gastric cancer with S-1. BMC CANCER. 2013 2013-01-01;13.
236	Hosein PJ 2015	Population inconformity	Hosein PJ, Ray N, Anthony LB, et al. Paclitaxel, carboplatin, and capecitabine (TCX) with and without radiation in locally advanced and metastatic distal esophageal and esophagogastric junction cancer: A single-center retrospective review. J CLIN ONCOL. 2015 2015-01-01;33(3).
237	Hosokawa A 2007	Single-arm trial	Hosokawa A, Sugiyama T, Ohtsu A, et al. Long-term outcomes of patients with metastatic gastric cancer after initial S-1 monotherapy. J GASTROENTEROL. 2007 2007-01-01;42(7):533-8.
238	Hou A 2009	Repeated article	Hou A, Hu Y, Zhou W, et al. Weekly docetaxel, cisplatin and low dose fluorouracil for advanced gastric carcinoma: A randomized clinical trial. Chinese-German Journal of Clinical Oncology. 2009 2009-01-01;8(9):516-9.
239	Huang D 2013	Repeated article	Huang D, Ba Y, Xiong J,et al. A multicentre randomised trial comparing weekly paclitaxel + S-1 with weekly paclitaxel + 5-fluorouracil for patients with advanced gastric cancer. Eur J Cancer. 2013 Sep;49(14):2995-3002. doi: 10.1016/j.ejca.2013.05.021.

240	Huang D 2013	Repeated article	Huang D, Ba Y, Xiong J, et al. A multicentre randomised trial comparing weekly paclitaxel + S-1 with weekly paclitaxel + 5-fluorouracil for patients with advanced gastric cancer. EUR J CANCER. 2013 2013-01-01;49(14):2995-3002.
241	Huang DZ 2012	Repeated article	Huang DZ, Xiong JP, Xu N, et al. A phase II prospective randomized controlled trial of weekly paclitaxel combined with S-1 or fluorouracil for advanced gastric carcinoma. Zhonghua Zhong Liu Za Zhi. 2012 Nov;34(11):865-8. doi: 10.3760/cma.j.issn.0253-3766.2012.11.015.
242	Hultman B 2013	Single-arm trial	Hultman B, Lind P, Glimelius B, et al. Phase II study of patients with peritoneal carcinomatosis from gastric cancer treated with preoperative systemic chemotherapy followed by peritonectomy and intraperitoneal chemotherapy. ACTA ONCOL. 2013 2013-01-01;52(4):824-30.
243	Hwang JE 2012	Single-arm trial	Hwang JE, Kim DE, Shim HJ, Bae WK, Cho SH, Chung IJ. First-line single agent chemotherapy in recurred or metastatic gastric cancer patients with poor clinical condition. ANN ONCOL. 2012 2012-01-01;23:v48.
244	Hwang JE 2012	Single-arm trial	Hwang JE, Kim HN, Kim DE, et al. First-line single-agent chemotherapy for patients with recurrent or metastatic gastric cancer with poor performance status. EXP THER MED. 2012 2012-01-01;4(4):562-8.
245	Iacovelli R 2014	Review	Iacovelli R, Pietrantonio F, Farcomeni A,et al.Chemotherapy or targeted therapy as second-line treatment of advanced gastric cancer. A systematic review and meta-analysis of published studies.PLoS One. 2014 Sep 30;9(9):e108940. doi: 10.1371/journal.pone.0108940.eCollection 2014.
246	Ichikawa W 2005	Single-arm trial	Ichikawa W, Takahashi T, Suto K, Hirayama R. Prediction of anti-tumor effect and adverse event in gastrointestinal cancer chemotherapy. Biotherapy. 2005 2005-01-01;19(3):248-54.
247	Ichikawa W 2006	Review	Ichikawa W, Sasaki Y. Correlation between tumor response to first-line chemotherapy and prognosis in advanced gastric cancer patients. ANN ONCOL. 2006 2006-01-01;17(11):1665-72.
248	Ikeda R 2009	Single-arm trial	Ikeda R, Yoshida K, Satou Y, et al. Randomized phase II/III study of docetaxel/S-1 (DS-1) versus CDDP/5FU (FUP) in advanced or recurrent gastric cancer: Updated phase II results. J CLIN ONCOL. 2009 2009-01-01;27(15):4595.
249	Ilmberger C 2014	Single-arm trial	Ilmberger C, Von Dehn-Rotfelser K, Schirra J, Joka M, Jauch KW, Mayer B. Adding docetaxel to cisplatin based therapy represents a beneficial option for gastric cancer patients in the perioperative situation. Oncology Research and Treatment. 2014 2014-01-01;37:53.
250	Ilson DH 2003	Population inconformity	Ilson DH, Minsky B.Irinotecan in esophageal cancer.Oncology (Williston Park). 2003 Sep;17(9 Suppl 8):32-6.

251	Im CK 2008	Single-arm trial	Im CK, Jeung HC, Rha SY, et al. A phase II study of paclitaxel combined with infusional 5-fluorouracil and low-dose leucovorin for advanced gastric cancer. Cancer Chemother Pharmacol. 2008 Feb;61(2):315-21.
252	Imamoto H 2011	Single-arm trial	Imamoto H, Oba K, Sakamoto J, et al. Assessing clinical benefit response in the treatment of gastric malignant ascites with non-measurable lesions: a multicenter phase II trial of paclitaxel for malignant ascites secondary to advanced/recurrent gastric cancer. Gastric Cancer. 2011 Mar;14(1):81-90. doi: 10.1007/s10120-011-0016-6.
253	Imano M 2011	Single-arm trial	Imano M, Imamoto H, Itoh T, et al. Impact of intraperitoneal chemotherapy after gastrectomy with positive cytological findings in peritoneal washings. EUR SURG RES. 2011 2011-01-01;47(4):254-9.
254	Imano M 2012	Single-arm trial	Imano M, Peng YF, Itoh T, et al. A preliminary study of single intraperitoneal administration of paclitaxel followed by sequential systemic chemotherapy with S-1 plus paclitaxel for advanced gastric cancer with peritoneal metastasis. ANTICANCER RES. 2012 2012-01-01;32(9):4071-5.
255	Imano M 2012	Single-arm trial	Imano M, Yasuda A, Itoh T, et al. Phase II Study of Single Intraperitoneal Chemotherapy Followed by Systemic Chemotherapy for Gastric Cancer with Peritoneal Metastasis. J GASTROINTEST SURG. 2012 2012-01-01;16(12):2190-6.
256	Inada S 2009	Single-arm trial	Inada S, Tomidokoro T, Fukunari H, et al. Phase I/II trial of combination therapy with S-1 and weekly paclitaxel in patients with unresectable or recurrent gastric cancer. Cancer Chemother Pharmacol. 2009 Jan;63(2):267-73. doi:10.1007/s00280-008-0736-4.
257	Inal A 2012	Repeated article	Inal A, Kaplan MA, Kucukoner M,et al. Docetaxel and Cisplatin Plus Fluorouracil compared with Modified Docetaxel, Cisplatin, and 5-Fluorouracil as first-line therapy for advanced gastric cancer: a retrospective analysis of single institution. Neoplasma. 2012;59(2):233-6.
258	Inokuchi M 2007	Single-arm trial	Inokuchi M, Yamashita T, Yamada H,et al. Second-line chemotherapy in gastric cancer following S-1 with CPT-11 chemotherapy performed as clinical trial. Gan To Kagaku Ryoho. 2007 Jun;34(6):875-9.
259	Ishigami H 2016	Single-arm trial	Ishigami H, Fujiwara Y, Fukushima R, et al. Phase III study of intraperitoneal paclitaxel plus s-1/paclitaxel compared with s-1/cisplatin in gastric cancer patients with peritoneal metastasis: PHOENIX-GC trial. J CLIN ONCOL. 2016 2016-01-01;34.
260	Izuishi K 2016	Interventions inconformity	Izuishi K, Mori H. Recent strategies for treating stage IV gastric cancer: Roles of palliative gastrectomy, chemotherapy, and radiotherapy. J GASTROINTEST LIVER. 2016 2016-01-01;25(1):87-94.
261	Jary M 2014	Single-arm trial	Jary M, Ghiringhelli F, Jacquin M, et al. Phase II multicentre study of efficacy and feasibility of dose-intensified preoperative weekly cisplatin, epirubicin, and paclitaxel (PET) in resectable gastroesophageal cancer. Cancer Chemother Pharmacol. 2014

			Jul;74(1):141-50. doi:10.1007/s00280-014-2482-0.
262	Jatoi A 2002	Single-arm trial	Jatoi A, Tirona MT, Cha SS, et al. A phase II trial of docetaxel and CPT-11 in patients with metastatic adenocarcinoma of the esophagus, gastroesophageal junction, and gastric cardia. Int J Gastrointest Cancer. 2002;32(2-3):115-23.
263	Jatoi A 2002	Single-arm trial	Jatoi A, Tirona MT, Cha SS, et al. A phase II trial of docetaxel and CPT-11 in patients with metastatic adenocarcinoma of the esophagus, gastroesophageal junction, and gastric cardia. International Journal of Gastrointestinal Cancer. 2002 2002-01-01;32(2-3):115-23.
264	Jatoi A 2008	Population inconformity	Jatoi A, Dakhil SR, Foster NR,et al. Bortezomib, paclitaxel, and carboplatin as a first-line regimen for patients with metastatic esophageal, gastric, and gastroesophageal cancer: phase II results from the North Central Cancer Treatment Group (N044B). J Thorac Oncol. 2008 May;3(5):516-20. doi: 10.1097/JTO.0b013e31816de276.
265	Javle M 2010	Repeated article	Javle M, Hsueh CT. Recent advances in gastrointestinal oncology - Updates and insights from the 2009 annual meeting of the American Society of Clinical Oncology. Journal of Hematology and Oncology. 2010 2010-01-01;3.
266	Javle M 2014	Study design inconformity	Javle M, Smyth EC, Chau I. Ramucirumab: Successfully targeting angiogenesis in gastric cancer. CLIN CANCER RES. 2014 2014-01-01;20(23):5875-81.
267	Jeung HC 2011	Repeated article	Jeung HC, Rha SY, Im CK, et al.A randomized phase 2 study of docetaxel and S-1 versus docetaxel and cisplatin in advanced gastric cancer with an evaluation of SPARC expression for personalized therapy.Cancer. 2011 May 15;117(10):2050-7. doi: 10.1002/cncr.25729. Epub 2010 Nov 29.
268	Jeung HC 2011	Repeated article	Jeung HC, Rha SY, Im CK, et al. A randomized phase 2 study of docetaxel and S-1 versus docetaxel and cisplatin in advanced gastric cancer with an evaluation of SPARC expression for personalized therapy. CANCER-AM CANCER SOC. 2011 2011-01-01;117(10):2050-7.
269	Jiang H 2014	Single-arm trial	Jiang H, Zheng Y, Qian J, et al. A phase II study of biweekly paclitaxel and S-1 (SPA) as first-line combination therapy in patients with metastatic or locally advanced gastric cancer. J CLIN ONCOL. 2014 2014-01-01;32(15).
270	Jiang H 2015	Single-arm trial	Jiang H, Qian J, Zhao P, et al. A phase II study of biweekly S-1 and paclitaxel (SPA) as first-line chemotherapy in patients with metastatic or advanced gastric cancer. Cancer Chemother Pharmacol. 2015 Jul;76(1):197-203. doi:10.1007/s00280-015-2782-z.
271	Jiang H 2015	Single-arm trial	Jiang H, Qian J, Zhao P, et al. A phase II study of biweekly S-1 and paclitaxel (SPA) as first-line chemotherapy in patients with metastatic or advanced gastric cancer. CANCER CHEMOTH PHARM. 2015 2015-01-01;76(1):197-203.

272	Jiang L 2015	Population inconformity	Jiang L, Yang KH, Guan QL, Chen Y, Zhao P, Tian JH. Survival benefit of neoadjuvant chemotherapy for resectable cancer of the gastric and gastroesophageal junction. J CLIN GASTROENTEROL. 2015 2015-01-01;49(5):387-94.
273	Jin H 2013	Repeated article	Jin H, Tang Y. Clinical outcomes of patients with advanced gastric cancer receiving chemotherapy with docetaxel in combination with S-1 or 5-fluorouracil. Tumor. 2013 2013-01-01;33(7):640-4.
274	Jin H 2016	Repeated article	Jin H, Tang Y.Clinical outcomes of patients with advanced gastric cancer receiving chemotherapy with docetaxel in combination with S-1 or 5-fluorouracil.TUMOR Vol. 33, July 2013:641-644.doi: 10.3781/j.issn.1000-7431.2013.07.012.
275	Jin Y 2013	Single-arm trial	Jin Y, Qiu MZ, Wang DS, et al. Adjuvant Chemotherapy for Elderly Patients with Gastric Cancer after D2 Gastrectomy. PLOS ONE. 2013 2013-01-01;8(1).
276	Jung M 2012	Repeated article	Jung M, Kang SY, Kim BS, et al. Postoperation chemotherapy with S1 and docetaxel in curatively resected gastric cancer of stage III (POST trial). J CLIN ONCOL. 2012 2012-01-01;30(15).
277	Kai K 2007	Single-arm trial	Kai K, Kitajima Y, Hiraki M, et al. Quantitative double-fluorescence immunohistochemistry (qDFIHC), a novel technology to assess protein expression: a pilot study analyzing 5-FU sensitive markers thymidylate synthase, dihydropyrimidine dehydrogenase and orotate phosphoribosyl transferases in gastric cancer tissue specimens. Cancer Lett. 2007 Dec 8;258(1):45-54. doi: 10.1016/j.canlet.2007.08.008.
278	Kakeji Y 2015	Single-arm trial	Kakeji Y, Kodera Y, Yoshida K, et al. Pre-planned feasibility and safety analyses of docetaxel/S-1 combination in a phase III study comparing docetaxel/S-1with S-1 alone as postoperative adjuvant chemotherapy for stage III gastric cancer (JACCRO GC-07). J CLIN ONCOL. 2015 2015-01-01;33(15).
279	Kalinka-Warzocha E 2015	Review	Kalinka-Warzocha E, Plazas JG, Mineur L, et al. Chemotherapy treatment patterns and neutropenia management in gastric cancer. GASTRIC CANCER. 2015 2015-01-01;18(2):360-7.
280	Kanagavel D 2010	Single-arm trial	Kanagavel D, Pokataev IA, Fedyanin MY, et al. A prognostic model in patients treated for metastatic gastric cancer with second-line chemotherapy.Ann Oncol. 2010 Sep;21(9):1779-85. doi: 10.1093/annonc/mdq032. Epub 2010 Feb 11.
281	Kanagavel D 2010	Single-arm trial	Kanagavel D, Pokataev IA, Fedyanin MY, et al. A prognostic model in patients treated for metastatic gastric cancer with second-line chemotherapy. ANN ONCOL. 2010 2010-01-01;21(9):1779-85.

282	Kanazawa J 2000	Population inconformity	Kanazawa J, Morimoto M, Ohmori K. Properties of antitumor activity of vinorelbine tartrate, a new vinca alkaloid antitumor agent. Nihon Yakurigaku Zasshi. 2000 Oct;116(4):215-23.
283	Kandil MS 2014	Review	Kandil MS, Bazarbashi SN, Rahal MM, et al. Saudi Oncology Society clinical management guideline series: Gastric cancer 2014. SAUDI MED J. 2014 2014-01-01;35(12):1529-33.
284	Kang BW 2014	Review	Kang BW, Kim JG, Kwon OK, Chung HY, Yu W. Non-platinum-based chemotherapy for treatment of advanced gastric cancer: 5-fluorouracil, taxanes, and irinotecan. WORLD J GASTROENTERO. 2014 2014-01-01;20(18):5396-402.
285	Kang BW 2016	Review	Kang BW, Kwon OK, Chung HY, Yu W, Kim JG. Taxanes in the Treatment of Advanced Gastric Cancer. Molecules (Basel, Switzerland). 2016 2016-01-01;21(5).
286	Kang H 2009	Single-arm trial	Kang H, Oh S, Kim J, et al. A phase II study of docetaxel and oxaliplatin combination as first-line chemotherapy in recurrent gastric cancer patients after fluoropyrimidine and/or cisplatin adjuvant treatment. European Journal of Cancer, Supplement. 2009 2009-01-01;7(2-3):375.
287	Kang H 2011	Review	Kang H, Kauh JS. Chemotherapy in the treatment of metastatic gastric cancer: Is There a global standard? CURR TREAT OPTION ON. 2011 2011-01-01;12(1):96-106.
288	Kang HJ 2008	Single-arm trial	Kang HJ, Chang HM, Kim TW, et al. A phase II study of paclitaxel and capecitabine as a first-line combination chemotherapy for advanced gastric cancer. BRIT J CANCER. 2008 2008-01-01;98(2):316-22.
289	Kang JH 2012	Repeated article	Kang JH, Lee SI, Lim DH, et al. Salvage chemotherapy for pretreated gastric cancer: a randomized phase III trial comparing chemotherapy plus best supportive care with best supportive care alone. J Clin Oncol. 2012 May 1;30(13):1513-8. doi: 10.1200/JCO.2011.39.4585.
290	Kang YK 2013	Single-arm trial	Kang YK, Rha SY, Tassone P, et al. Safety of two different doses of pertuzumab (P) in combination with trastuzumab (T) and chemotherapy (CTx) in patients (pts) with HER2-positive advanced gastric cancer (aGC). EUR J CANCER. 2013 2013-01-01;49:S620-1.
291	Kang YK 2013	Single-arm trial	Kang YK, Rha SY, Tassone P, et al. Pertuzumab pharmacokinetics and safety in combination with trastuzumab and chemotherapy in patients with her2-positive advanced gastric cancer (AGC). ANN ONCOL. 2013 2013-01-01;24:v19.
292	Kang YK 2015	Single-arm trial	Kang YK, Yook JH, Ryu MH, et al. A randomized phase III study of neoadjuvant chemotherapy with docetaxel(D), oxaliplatin(O), and S-1(S) (DOS) followed by surgery and adjuvant S-1 vs. surgery and adjuvant S-1 for resectable advanced gastric cancer (PRODIGY). J CLIN ONCOL. 2015 2015-01-01;33(15).

293	Kantzou I 2011	Review	Kantzou I, Sarris G, Poulizi M, et al. Gastric cancer and adjuvant chemoradiotherapy: When and where, that's the question. Journal of B.U.ON. 2011 2011-01-01;16(3):473-7.
294	Kataoka H 2009	Interventions inconformity	Kataoka H, Shimura T, Mizoshita T, et al. Lentinan with S-1 and paclitaxel for gastric cancer chemotherapy improve patient quality of life. Hepatogastroenterology. 2009 Mar-Apr;56(90):547-50.
295	Katayama H 2012	Study design inconformity	Katayama H, Ito S, Sano T, et al.A Phase II study of systemic chemotherapy with docetaxel, cisplatin, and S-1 (DCS) followed by surgery in gastric cancer patients with extensive lymph node metastasis: Japan Clinical Oncology Group study JCOG1002.Jpn J Clin Oncol. 2012 Jun;42(6):556-9. doi: 10.1093/jjco/hys054. Epub 2012 Apr 23.
296	Kato K 2011	Single-arm trial	Kato K, Chin K, Yoshikawa T, et al. Phase II study of NK105, a paclitaxel-incorporating micellar nanoparticle, for previously treated advanced or recurrent gastric cancer. Invest New Drugs. 2012 Aug;30(4):1621-7. doi: 10.1007/s10637-011-9709-2.
297	Kato S 2014	Single-arm trial	Kato S. Treatment strategy of patients with unresectable advanced or recurrent gastric cancer. ANN ONCOL. 2014 2014-01-01;25:v14.
298	Katoh M 2004	Review	Katoh M, Katoh M. Pharmacogenomics on gastric cancer. Cancer Biology and Therapy. 2004 2004-01-01;3(6):566-7.
299	Khushalani NI 2012	Single-arm trial	Khushalani NI. Phase I and II clinical trials for gastric cancer. SURG ONCOL CLIN N AM. 2012 2012-01-01;21(1):113-28.
300	Kii T 2011	Repeated article	Kii T, Takiuchi H, Sugimoto N, et al. Randomized phase II trial of S-1 plus irinotecan versus S-1 plus paclitaxel as first-line treatment for advanced gastric cancer (OGSG0402): Final report. ANN ONCOL. 2011 2011-01-01;22:x60.
301	Kim B 2015	Repeated article	Kim B, Lee KW, Kim MJ, Han HS, Park YL, Park SR. A multicenter randomized phase II study of docetaxel vs. docetaxel plus cisplatin vs. docetaxel plus S-1 as second-line chemotherapy in metastatic gastric cancer patients who had progressed after cisplatin plus either S-1 or capecitabine. EUR J CANCER. 2015 2015-01-01;51:S432.
302	Kim HS 2013	Review	Kim HS, Kim HJ, Kim SY, et al. Second-line chemotherapy versus supportive cancer treatment in advanced gastric cancer: a meta-analysis.Ann Oncol. 2013 Nov;24(11):2850-4. doi: 10.1093/annonc/mdt351. Epub 2013 Aug 13.
303	Kim HS 2016	Single-arm trial	Kim HS, Ryu MH, Zang DY, et al. Phase II study of docetaxel, oxaliplatin, and S-1 therapy in patients with metastatic gastric cancer. Gastric Cancer. 2016 Apr;19(2):579-85. doi: 10.1007/s10120-015-0503-2.
304	Kim HS 2016	Review	Kim HS, Kim JH, Kim JW, Kim BC. Chemotherapy in elderly patients with gastric Cancer. J CANCER. 2016 2016-01-01;7(1):88-94.

305	Kim J 2011	Repeated article	Kim J, Park K, Yi S, Lee H. The efficacy and tolerability of modified docetaxel, cisplatin, and 5FU in patients with advanced or recurrent gastric cancer: A retrospective analysis. J CLIN ONCOL. 2011 2011-01-01;29(4).
306	Kim JA 2011	Repeated article	Kim JA, Lee J, Han B, et al. Docetaxel/cisplatin followed by FOLFIRI versus the reverse sequence in metastatic gastric cancer. Cancer Chemother Pharmacol. 2011 Jul;68(1):177-84. doi:10.1007/s00280-010-1452-4.
307	Kim JH 2010	Single-arm trial	Kim JH, Lee KW, Kim YH, et al.Individualized tumor response testing for prediction of response to Paclitaxel and Cisplatin chemotherapy in patients with advanced gastric cancer.J Korean Med Sci. 2010 May;25(5):684-90. doi: 10.3346/jkms.2010.25.5.684. Epub 2010 Apr 21.
308	Kim JW 2015	Single-arm trial	Kim JW, Choi IS, Kim YJ, et al. A phase I/II trial of second-line chemotherapy with paclitaxel and irinotecan in fluoropyrimidine- and platinum-pretreated patients with advanced gastric cancer. Cancer Chemother Pharmacol. 2015 Jun;75(6):1175-82. doi:10.1007/s00280-015-2732-9.
309	Kim JW 2016	Repeated article	Kim JW, Cho SH, Park YI, et al. Efficacy and safety findings from DREAM: A phase III study of DHP107 (oral paclitaxel) vs IV paclitaxel in patients with gastric cancer after failure of first-line chemotherapy. J CLIN ONCOL. 2016 2016-01-01;34.
310	Kim JY 2011	Single-arm trial	Kim JY, Do YR, Park KU,et al. Multicenter phase II trial of S-1, paclitaxel and cisplatin triplet combination chemotherapy in patients with advanced gastric cancer. Cancer Chemother Pharmacol. 2011 Mar;67(3):527-32. doi:10.1007/s00280-010-1353-6.
311	Kim JY 2012	Single-arm trial	Kim JY, Do YR, Park KU, et al. Multicenter randomized phase ii study of weekly docetaxel alone versus weekly docetaxel plus oxaliplatin as a second-line chemotherapy in patients with advanced gastric cancer: Preliminary response and safety results. ANN ONCOL. 2012 2012-01-01;23:i20.
312	Kim JY 2012	Single-arm trial	Kim JY, Do YR, Park KU, Kim MK, Ha EY, Song HS. Analysis of prognostic factors in elderly patients with metastatic gastric cancer given taxol, cisplatin, and S1 combination chemotherapy. EUR J CANCER. 2012 2012-01-01;48:S1.
313	Kim JY 2012	Single-arm trial	Kim JY, Do YR, Park K, Song HS. Analysis of prognostic factors in elderly patients with metastatic gastric cancer given Taxol, Cisplatin, and s1 combination chemotherapy. ANN ONCOL. 2012 2012-01-01;23:i45.
314	Kim JY 2015	Repeated article	Kim JY, Ryoo HM, Bae SH, et al. Multi-center Randomized Phase II Study of Weekly Docetaxel Versus Weekly Docetaxel-plus-Oxaliplatin as a Second-line Chemotherapy for Patients with Advanced Gastric Cancer. Anticancer Res. 2015 Jun;35(6):3531-6.

315	Kim JY 2015	Single-arm trial	Kim JY, Ryoo HM, Bae SH, et al. Multi-center randomized phase ii study of weekly docetaxel Versus weekly docetaxel-plus-oxaliplatin as a second-line chemotherapy for patients with advanced gastric cancer. ANTICANCER RES. 2015 2015-01-01;35(6):3531-6.
316	Kim KH 2009	Single-arm trial	Kim KH, Park YS, Chang MH, et al. A phase I/II trial of docetaxel and oxaliplatin in patients with advanced gastric cancer. Cancer Chemother Pharmacol. 2009 Jul;64(2):347-53. doi:10.1007/s00280-008-0878-4.
317	Kim R 1999	Study design inconformity	Kim R, Ohi Y, Inoue H, et al. Activation and the interaction of proapoptotic genes in modulating sensitivity to anticancer drugs in gastric cancer cells. Int J Oncol. 1999 Oct;15(4):751-6.
318	Kim S 2017	Repeated article	Kim S, Paget-Bailly S, Messager M, et al. Perioperative docetaxel, cisplatin, and 5-fluorouracil compared to standard chemotherapy for resectable gastroesophageal adenocarcinoma. Eur J Surg Oncol. 2017 Jan;43(1):218-225. doi:10.1016/j.ejso.2016.06.395.
319	Kim SG 2007	Single-arm trial	Kim SG, Oh SY, Kwon HC, et al. A phase II study of irinotecan with bi-weekly, low-dose leucovorin and bolus and continuous infusion 5-fluorouracil (modified FOLFIRI) as salvage therapy for patients with advanced or metastatic gastric cancer. JPN J CLIN ONCOL. 2007 2007-01-01;37(10):744-9.
320	Kim SM 2015	Review	Kim SM, Park SH. Chemotherapy beyond second-line in advanced gastric cancer. WORLD J GASTROENTERO. 2015 2015-01-01;21(29):8912-9.
321	Kim ST 2012	Single-arm trial	Kim ST, Park KH, Oh SC, et al. Is chemotherapy in elderly patients with metastatic or recurrent gastric cancer as tolerable and effective as in younger patients? ASIA-PAC J CLIN ONCO. 2012 2012-01-01;8(2):194-200.
322	Kim TY 2016	Repeated article	Kim TY, Yen CJ, Al-Batran SE, et al. Exposure-response relationship of second-line ramucirumab in east asian patients with advanced gastric cancer from RAINBOW, a global, randomized, double-blind, phase 3 study. ANN ONCOL. 2016 2016-01-01;27:i116-7.
323	Kim YH 2007	Study design inconformity	Kim YH, Yamaguchi K, Bang YJ, et al.Phase II study of biweekly paclitaxel and cisplatin combination chemotherapy in advanced gastric cancer: Korea Japan Collaborative Study Group Trial. Jpn J Clin Oncol. 2007 Jul;37(7):501-8. Epub 2007 Aug 2.
324	Kim YH 2008	Single-arm trial	Kim YH, Seo HY, Jeen YT, et al. Phase I dose escalation study of docetaxel with a fixed dose of S-1 in combination chemotherapy for advanced gastric cancer. Cancer Chemother Pharmacol. 2009 Jan;63(2):253-60. doi:10.1007/s00280-008-0734-6.

325	Kim YH 2009	Single-arm trial	Kim YH, Seo HY, Jeen YT, Kim HK, Shim BY, Yang J. Phase i dose escalation study of docetaxel with a fixed dose of S-1 in combination chemotherapy for advanced gastric cancer. CANCER CHEMOTH PHARM. 2009 2009-01-01;63(2):253-60.
326	Kim YH 2011	Repeated article	Kim YH, Fujii M, Kim HK, Nakajima T. Randomized phase III study of S-1 alone versus S-1 plus docetaxel (DOC) in the treatment for advanced gastric cancer (AGC): The start trial update. ANN ONCOL. 2011 2011-01-01;22:x24.
327	Kim YJ 2011	Single-arm trial	Kim YJ, Goh PG, Kim ES, et al. [Comparison of the toxicities and efficacies of the combination chemotherapy regimens in advanced gastric cancer patients who achieved complete response after chemotherapy]. The Korean journal of gastroenterology = Taehan Sohwagi Hakhoe chi. 2011 2011-01-01;58(6):311-7.
328	Kim YS 2014	Repeated article	Kim YS, Sym SJ, Park SH, et al. A randomized phase II study of weekly docetaxel/cisplatin versus weekly docetaxel/oxaliplatin as first-line therapy for patients with advanced gastric cancer. Cancer Chemother Pharmacol. 2014 Jan;73(1):163-9. doi: 10.1007/s00280-013-2334-3
329	Kim YW 2014	Single-arm trial	Kim YW, Ryu KW, Choi IJ, Kook MC, Park YI, Kim HK. Updated results of a randomized phase II trial for neoadjuvant versus adjuvant docetaxel/cisplatin chemotherapy in patients with locally advanced gastric cancer. J CLIN ONCOL. 2014 2014-01-01;32(3).
330	Kinoshita J 2015	Single-arm trial	Kinoshita J, Fushida S, Tsukada T, et al. Efficacy of conversion gastrectomy following docetaxel, cisplatin, and S-1 therapy in potentially resectable stage IV gastric cancer. European Journal of Surgical Oncology. 2015 2015-01-01;41(10):1354-60.
331	Kinoshita J 2016	Repeated article	Kinoshita J, Fushida S, Kaji M, et al. Paclitaxel plus valproic acid versus paclitaxel alone as second or third line therapy for advanced gastric cancer: A randomized phase 2 trial. ANN ONCOL. 2016 2016-01-01;27:i82.
332	Kishimoto T 2010	Review	Kishimoto T, Imamura H, Uedou F, et al. Randomized phase II trial of S-1 plus irinotecan versus S-1 plus paclitaxel as first-line treatment for advanced gastric cancer (OGSG0402): Final report. J CLIN ONCOL. 2010 2010-01-01;28(15).
333	Kitajima M 2009	Single-arm trial	Kitajima M, Satoh G, Marusasa T, et al. A case of gastric cancer with peritoneal dissemination-efficacy of combination therapy with S-1 and docetaxel. Gan To Kagaku Ryoho. 2009 Sep;36(9):1545-8.
334	Kobayashi M 2007	Single-arm trial	Kobayashi M, Oba K, Sakamoto J, et al. Pharmacokinetic study of weekly administration dose of paclitaxel in patients with advanced or recurrent gastric cancer in Japan. GASTRIC CANCER. 2007 2007-01-01;10(1):52-7.
335	Kobayashi M 2012	Repeated article	Kobayashi M, Tsuburaya A, Yoshida K, et al. Adjuvant paclitaxel followed by oral fluoropyrimidines for gastric cancer: Safety data of the factorial phase III SAMIT trial. J CLIN ONCOL. 2012 2012-01-01;30(4).

336	Kodera Y 2006	Study design inconformity	Kodera Y, Ito S, Fujiwara M, et al. In vitro chemosensitivity test to predict chemosensitivity for paclitaxel, using human gastric carcinoma tissues. Int J Clin Oncol. 2006 Dec;11(6):449-53. doi: 10.1007/s10147-006-0618-x.
337	Kodera Y 2007	Single-arm trial	Kodera Y, Ito S, et al. Mochizuki Y,A phase II study of weekly paclitaxel as second-line chemotherapy for advanced gastric Cancer (CCOG0302 study).Anticancer Res. 2007 Jul-Aug;27(4C):2667-71.
338	Kodera Y 2007	Single-arm trial	Kodera Y, Ito Y, Ito S, et al. Intraperitoneal paclitaxel: a possible impact of regional delivery for prevention of peritoneal carcinomatosis in patients with gastric carcinoma. Hepatogastroenterology. 2007 Apr-May;54(75):960-3.
339	Kodera Y 2011	Study design inconformity	Kodera Y, Imano M, Yoshikawa T,et al.A randomized phase II trial to test the efficacy of intra-peritoneal paclitaxel for gastric cancer with high risk for the peritoneal metastasis (INPACT trial). Jpn J Clin Oncol. 2011 Feb;41(2):283-6. doi: 10.1093/jjco/hyq187. Epub 2010 Oct 14.
340	Kodera Y 2011	Single-arm trial	Kodera Y, Ito Y, Ohashi N, et al. Impact of clinical response to first-line chemotherapy on gastric cancer patients treated with second-line and third-line chemotherapy. HEPATO-GASTROENTEROL. 2011 2011-01-01;58(107-108):1041-5.
341	Kodera Y 2011	Single-arm trial	Kodera Y, Imano M, Yoshikawa T, et al. A randomized phase II trial to test the efficacy of intra-peritoneal paclitaxel for gastric cancer with high risk for the peritoneal metastasis (INPACT trial). JPN J CLIN ONCOL. 2011 2011-01-01;41(2):283-6.
342	Kodera Y 2017	Repeated article	Kodera Y, Takahashi N, Yoshikawa T, et al. Feasibility of weekly intraperitoneal versus intravenous paclitaxel therapy delivered from the day of radical surgery for gastric cancer: a preliminary safety analysis of the INPACT study, a randomized controlled trial. Gastric Cancer. 2017 Jan;20(1):190-199. doi: 10.1007/s10120-016-0598-0.
343	Koizumi W 2009	Single-arm trial	Koizumi W, Akiya T, Sato A,et al. Second-line chemotherapy with biweekly paclitaxel after failure of fluoropyrimidine-based treatment in patients with advanced or recurrent gastric<br>cancer: a report from the gastrointestinal oncology group of the Tokyo cooperative oncology group, TCOG GC-0501 trial.Jpn J Clin Oncol. 2009 Nov;39(11):713-9. doi: 10.1093/jjco/hyp099. Epub 2009 Oct 6.
344	Koizumi W 2014	Population inconformity	Koizumi W, Kim YH, Fujii M,et al. Addition of docetaxel to S-1 without platinum prolongs survival of patients with advanced gastric cancer: a randomized study (START). J Cancer Res Clin Oncol. 2014 Feb;140(2):319-28. doi: 10.1007/s00432-013-1563-5.
345	Koizumi W 2014	Repeated article	Koizumi W, Kim YH, Fujii M, et al. Addition of docetaxel to S-1 without platinum prolongs survival of patients with advanced gastric cancer: A randomized study (START). J CANCER RES CLIN. 2014 2014-01-01;140(2):319-28.

346	Koizumi W 2015	Study design inconformity	Koizumi W, Morita S, Sakata Y.A randomized Phase III trial of weekly or 3-weekly doses of nab-paclitaxel versus weekly doses of Cremophor-based paclitaxel in patients with previously treated advanced gastric cancer (ABSOLUTE Trial).Jpn J Clin Oncol. 2015 Mar;45(3):303-6. doi: 10.1093/jjco/hyu205. Epub 2014 Dec 16.
347	Koizumi W 2015	Single-arm trial	Koizumi W, Morita S, Sakata Y. A randomized Phase III trial of weekly or 3-weekly doses of nab-paclitaxel versus weekly doses of Cremophor-based paclitaxel in patients with previously treated advanced gastric cancer (ABSOLUTE Trial). JPN J CLIN ONCOL. 2015 2015-01-01;45(3):303-6.
348	Kondo K 2005	Single-arm trial	Kondo K, Kobayashi M, Kojima H, et al.Phase I evaluation of continuous 5-fluorouracil infusion followed by weekly paclitaxel in patients with advanced or recurrent gastric cancer.Jpn J Clin Oncol. 2005 Jun;35(6):332-7. Epub 2005 Jun 16.
349	Kono K 2017	Review	Kono K, Yong WP, Okayama H, et al. Intraperitoneal chemotherapy for gastric cancer with peritoneal disease: experience from Singapore and Japan. GASTRIC CANCER. 2017 2017-01-01;20:122-7.
350	Kornek GV 2002	Study design inconformity	Kornek GV(1), Raderer M, Schüll B,et al. Effective combination chemotherapy with paclitaxel and cisplatin with or without human granulocyte colony-stimulating factor and/or erythropoietin in patients with advanced gastric cancer.Br J Cancer. 2002 Jun 17;86(12):1858-63.DOI: 10.1038/sj.bjc.6600345
351	Kos FT 2011	Repeated article	Kos FT, Uncu D, Ozdemir N,et al. Comparison of cisplatin-5-fluorouracil-folinic acid versus modified docetaxel-cisplatin-5-fluorouracil regimens in the first-line treatment of metastatic gastric cancer. Chemotherapy. 2011;57(3):230-5. doi: 10.1159/000327840.
352	Koutcher JA 2000	Study design inconformity	Koutcher JA, Motwani M, Zakian KL,et al. The in vivo effect of bryostatin-1 on paclitaxel-induced tumor growth, mitotic entry, and blood flow.Clin Cancer Res. 2000 Apr;6(4):1498-507.
353	Kramskaya L 2013	Single-arm trial	Kramskaya L. Results of combined treatment of patients with gastric cancer stages III-IV. ANN ONCOL. 2013 2013-01-01;24:v62.
354	Kripp M 2014	Repeated article	Kripp M, Al-Batran SE, Rosowski J, et al. Quality of life of older adult patients receiving docetaxel-based chemotherapy triplets for esophagogastric adenocarcinoma: a randomized study of the Arbeitsgemeinschaft Internistische Onkologie (AIO). Gastric Cancer. 2014 Jan;17(1):181-7. doi: 10.1007/s10120-013-0242-1.
355	Kruijtzer CMF 2003	Single-arm trial	Kruijtzer CMF, Boot H, Beijnen JH, et al. Weekly oral paclitaxel as first-line treatment in patients with advanced gastric cancer. ANN ONCOL. 2003 2003-01-01;14(2):197-204.

356	Kubota T 2000	Review	Kubota T. Recent advance in gastric cancer chemotherapy. Gan To Kagaku Ryoho. 2000 Nov;27(13):2043-7.
357	Kubota T 2008	Review	Kubota T. The role of S-1 in the treatment of gastric cancer. BRIT J CANCER. 2008 2008-01-01;98(8):1301-4.
358	Kucukzeybek Y 2012	Single-arm trial	Kucukzeybek Y, Dirican A, Erten C, et al. Second-line irinotecan after cisplatin, fluoropyrimidin and docetaxel for chemotherapy of metastatic gastric cancer. ASIAN PAC J CANCER P. 2012 2012-01-01;13(6):2771-4.
359	Kumagai K 2015	Population inconformity	Kumagai K, Rouvelas I, Tsai JA, et al. Survival benefit and additional value of preoperative chemoradiotherapy in resectable gastric and gastro-oesophageal junction cancer: A direct and adjusted indirect comparison meta-analysis. European Journal of Surgical Oncology. 2015 2015-01-01;41(3):282-94.
360	Kunisaki C 2008	Single-arm trial	Kunisaki C, Takahashi M, Nagahori Y, et al. Phase I study of biweekly docetaxel and S-1 combination chemotherapy for advanced gastric cancer.Anticancer Res. 2008 Jul-Aug;28(4C):2473-8.
361	Kunisaki C 2008	Review	Kunisaki C, Makino H, Takagawa R, et al. Impact of palliative gastrectomy in patients with incurable advanced gastric cancer. ANTICANCER RES. 2008 2008-01-01;28(2 B):1309-15.
362	Kunisaki C 2012	Single-arm trial	Kunisaki C, Ono HA, Hasegawa S, et al. Low-dose docetaxel and cisplatin combination chemotherapy for stage II/III gastric cancer showing resistance to S-1 adjuvant chemotherapy: a phase I study. J Chemother. 2012 Dec;24(6):364-8. doi: 10.1179/1973947812Y.0000000042.
363	Kunisaki C 2012	Single-arm trial	Kunisaki C, Takahashi M, Ono HA,et al. Inflammation-based prognostic score predicts survival in patients with advanced gastric cancer receiving biweekly docetaxel and s-1 combination chemotherapy. Oncology. 2012;83(4):183-91. doi: 10.1159/000341346.
364	Kunisaki C 2013	Study design inconformity	Kunisaki C, Takahashi M, Ono HA, et al. Biweekly Docetaxel and S-1 combination chemotherapy as first-line treatment for elderly patients with advanced gastric cancer. Anticancer Res. 2013 Feb;33(2):697-704.
365	Kurokawa Y 2015	Single-arm trial	Kurokawa Y, Hamakawa T, Miyazaki Y, et al. Preoperative systemic and intraperitoneal chemotherapy consisting of S-1, cisplatin and docetaxel in patients with marginally resectable gastric cancer. Anticancer Res. 2015 Apr;35(4):2223-8.
366	Kurokawa Y 2015	Population inconformity	Kurokawa Y, Takiguchi S, Miyazaki Y, et al. Adjuvant treatment for esophagogastric junction cancer. Nihon Geka Gakkai Zasshi. 2015 Jan;116(1):50-4.
367	Kusumoto T 2013	Single-arm trial	Kusumoto T, Ando K, Ida S, et al. S-1/docetaxel compared with the other standard S-1 based regimens as a first-line chemotherapy for patients with advanced gastric cancer. J CLIN ONCOL. 2013 2013-01-01;31(15).

368	Kusumoto T 2016	Repeated article	Kusumoto T, Egashira A, Hashimoto K, et al. Comparison of efficacy among nab-paclitaxel, paclitaxel and irinotecan monotherapies as the second-line chemotherapy following S-1-containing regimens in patients with advanced gastric cancer. J CLIN ONCOL. 2016 2016-01-01;34.
369	Laface R 2014	Single-arm trial	Laface R, Giommoni E, Di Costanzo F. S-1 in the treatment of advanced gastric cancer: Up-date. EUR J ONCOL. 2014 2014-01-01;19(1):26-9.
370	Lee CK 2014	Single-arm trial	Lee CK, Jung M, Kang SY, et al. Randomized, multicenter, phase III trial to compare S-1 plus docetaxel (DS) with S-1 plus cisplatin (SP) in gastric cancer patients with stage III (POST trial). J CLIN ONCOL. 2014 2014-01-01;32(15).
371	Lee HJ 2008	Single-arm trial	Lee HJ, Cho DY, Park JC, et al. Phase II trial of biweekly paclitaxel plus infusional 5-fluorouracil and leucovorin in patients with advanced or recurrent inoperable gastric cancer. Cancer Chemother Pharmacol. 2009 Feb;63(3):427-32. doi:10.1007/s00280-008-0752-4.
372	Lee HJ 2012	Study design inconformity	Lee HJ, Park JC, Kim JH, et al. The ATP-based doublet chemotherapy response assay for un-resectable advanced gastric cancer; a single center, prospective, randomized controlled study. GASTROINTEST ENDOSC. 2012 2012-01-01;75(4):B236.
373	Lee JL 2008	Single-arm trial	Lee JL, Kang YK. Capecitabine in the treatment of advanced gastric cancer. FUTURE ONCOL. 2008 2008-01-01;4(2):179-98.
374	Lee KW 2005	Single-arm trial	Lee KW, Im SA, Yun T,et al. Phase II trial of low-dose paclitaxel and cisplatin in patients with advanced gastric cancer.Jpn J Clin Oncol. 2005 Dec;35(12):720-6. Epub 2005 Dec 6.
375	Lee SH 2004	Single-arm trial	Lee SH(1), Kang WK, Park J,et al.Combination chemotherapy with epirubicin, docetaxel and cisplatin (EDP) in metastatic or recurrent, unresectable gastric cancer. Br J Cancer. 2004 Jul 5;91(1):18-22.DOI: 10.1038/sj.bjc.6601891
376	Lee SH 2004	Single-arm trial	Lee SH, Kang WK, Park J, et al. Combination chemotherapy with epirubicin, docetaxel and cisplatin (EDP) in metastatic or recurrent, unresectable gastric cancer. BRIT J CANCER. 2004 2004-01-01;91(1):18-22.
377	Li J 2009	Repeated article	Li J, Li B, Zhong M. The efficacy of different combined chemotherapy regimens for advanced gastric carcinoma. Chinese Journal of Clinical Oncology. 2009 2009-01-01;36(8):205-7.
378	Li XD 2011	Repeated article	Li XD, Shen H, Jiang JT,et al.Paclitaxel based vs oxaliplatin based regimens for advanced gastric cancer.World J Gastroenterol. 2011 Feb 28;17(8):1082-7. doi: 10.3748/wjg.v17.i8.1082.
379	Li XD 2011	Repeated article	Li XD, Shen H, Jiang JT, et al. Paclitaxel based vs oxaliplatin based regimens for advanced gastric cancer. WORLD J GASTROENTERO. 2011 2011-01-01;17(8):1082-7.

380	Li Y 2016	Population inconformity	Li Y, Zhou YF, Liang H, et al. Chinese expert consensus on cytoreductive surgery and hyperthermic intraperitoneal chemotherapy for peritoneal malignancies. WORLD J GASTROENTERO. 2016 2016-01-01;22(30):6906-16.
381	Li Z 2015	Repeated article	Li Z, Fan B, Shan F， et al. Gastrectomy in comprehensive treatment of advanced gastric cancer with synchronous liver metastasis: a prospectively comparative study.World J Surg Oncol. 2015 Jul 1;13:212. doi: 10.1186/s12957-015-0627-1.
382	Liao G 2012	Interventions inconformity	Liao G, Qu Y, Wang H, Liu P, Li L. Clinical research on continuous hyperthermic perfusion in the treatment of peritoneal effusion induced by gastric carcinoma. Chinese Journal of Clinical Oncology. 2012 2012-01-01;39(8):452-4.
383	Liepa A 2015	Study design inconformity	Liepa A, Mitchell S, Batson S, et al. Systematic review and meta-analysis of recommended second-line therapies for advanced gastric cancer (GC). EUR J CANCER. 2015 2015-01-01;51:S437.
384	Lim D 2009	Repeated article	Lim D, Lee D, Park D, et al. A retrospective study of first-line platinum-based combination chemotherapy in patients with recurrent and advanced gastric cancer. European Journal of Cancer, Supplement. 2009 2009-01-01;7(2-3):371.
385	Lim DH 2010	Repeated article	Lim DH, Park SH, Park KW, et al. Retrospective analyses of cisplatin-based doublet combination chemotherapy in patients with advanced gastric cancer. BMC CANCER. 2010 2010-01-01;10.
386	Lin R 2009	Single-arm trial	Lin R, Chen Q, Fan N, et al. Phase IIb trial of fluorouracil, leucovorin, oxaliplatin, and paclitaxel (POF) compared with fluorouracil, feucovorin, and irinotecan (IF) as first-line treatment for advanced gastric cancer (AGC). J CLIN ONCOL. 2009 2009-01-01;27(15):e15642.
387	Liu GF 2014	Study design inconformity	Liu GF, Tang D, Li P, et al. S-1-based combination therapy vs S-1 monotherapy in advanced gastric cancer: A meta-analysis. WORLD J GASTROENTERO. 2014 2014-01-01;20(1):310-8.
388	Liu H 2014	Review	Liu H, Chen X, Sun J, et al. The efficacy and toxicity of paclitaxel plus S-1 compared with paclitaxel plus 5-Fu for advanced gastric cancer: A PRISMA systematic review and meta-analysis of randomized controlled trials. Medicine (United States). 2014 2014-01-01;93(25).
389	Liu L 2013	Repeated article	Liu L, Yang X, Wang H, et al. Efficacy and perioperative effect of docetaxel plus oxaliplatin and S-1 in treating advanced gastric cancer. Chinese Journal of Clinical Oncology. 2013 2013-01-01;40(13):788-91.
390	Liu Y 2010	Single-arm trial	Liu Y, Ma T, Ye ZB, et al. Efficacy and safety evaluation of docetaxel plus oxaliplatin and capecitabine in the treatment of advanced gastric adenocarcinoma: a single center non-controlled phase II clinical trial. Zhonghua Wei Chang Wai Ke Za Zhi. 2010 Mar;13(3):177-80.

391	Liu Y 2010	Single-arm trial	Liu Y, Ma T, Ye ZB, Zhang J, Zhu ZG. Efficacy and safety evaluation of docetaxel plus oxaliplatin and capecitabine in the treatment of advanced gastric adenocarcinoma: a single center non-controlled phase II clinical trial. Zhonghua wei chang wai ke za zhi = Chinese journal of gastrointestinal surgery. 2010 2010-01-01;13(3):177-80.
392	Liu Y 2015	Review	Liu Y, Ling Y, Qi Q, et al. Trastuzumab increases the sensitivity of HER2-amplified human gastric cancer cells to oxaliplatin and cisplatin by affecting the expression of telomere-associated proteins. ONCOL LETT. 2015 2015-01-01;9(2):999-1005.
393	Liu Y 2017	Single-arm trial	Liu Y, Zhao G, Xu Y, et al. Phase II Study of Adjuvant Chemoradiotherapy Using Docetaxel/Cisplatin/5-Fluorouracil Before and After Intensity-modulated Radiotherapy With Concurrent Docetaxel in Patients With Completely (R0) Resected Gastric Carcinoma. American Journal of Clinical Oncology: Cancer Clinical Trials. 2017 2017-01-01.
394	Liu YP 2015	Repeated article	Liu YP, Li GQ, Chen HH, et al. Capecitabine for treatment of patients with advanced gastric cancer: Curative efficacy and effect on serum levels of MMP-2 and MMP-9. World Chinese Journal of Digestology. 2015 2015-01-01;23(7):1136-40.
395	Lo SS 2010	Single-arm trial	Lo SS, Khorana AA, Javle M,et al. A phase II study of weekly docetaxel in combination with capecitabine in advanced gastric and gastroesophageal adenocarcinomas. Oncology. 2010;78(2):125-9. doi: 10.1159/000312654.
396	Lokich JJ 1999	Single-arm trial	Lokich JJ, Sonneborn H, Anderson NR, et al. Combined paclitaxel, cisplatin, and etoposide for patients with previously untreated esophageal and gastroesophageal carcinomas. Cancer. 1999 Jun 1;85(11):2347-51.
397	Lokich JJ 1999	Single-arm trial	Lokich JJ, Sonneborn H, Anderson NR, et al. Combined paclitaxel, cisplatin, and etoposide for patients with previously untreated esophageal and gastroesophageal carcinomas. CANCER-AM CANCER SOC. 1999 1999-01-01;85(11):2347-51.
398	London S 2014	Review	London S. Ramucirumab boosts chemotherapy in advanced gastric cancer. Oncology Report. 2014 2014-01-01(FEB):5.
399	Long HJ 1994	Review	Long HJ. Paclitaxel (Taxol): A novel anticancer chemotherapeutic drug. MAYO CLIN PROC. 1994 1994-01-01;69(4):341-5.
400	Lordick F 2005	Study design inconformity	Lordick F, Siewert JR. Neoadjuvant therapy for locally advanced gastric cancer. ONKOLOGE. 2005 2005-01-01;11(6):640-9.
401	Lordick F 2008	Single-arm trial	Lordick F, Ridwelski K, Al-Batran SE, Trarbach T, Schlag PM, Piso P. Treatment of gastric cancer. ONKOLOGIE. 2008 2008-01-01;31(SUPPL. 5):32-9.
402	Lordick F 2014	Review	Lordick F, Lorenzen S, Yamada Y, Ilson D. Optimal chemotherapy for advanced gastric cancer: Is there a global consensus? GASTRIC CANCER. 2014 2014-01-01;17(2):213-25.

403	Lordick F 2016	Single-arm trial	Lordick F. First-line therapy for metastatic gastric cancer - What treatment for which patient? Oncology Research and Treatment. 2016 2016-01-01;39:127-8.
404	Lorenzen S 2005	Single-arm trial	Lorenzen S(1), Duyster J, Lersch C, et al. Capecitabine plus docetaxel every 3 weeks in first- and second-line metastatic oesophageal cancer: final results of a phase II trial.Br J Cancer. 2005 Jun 20;92(12):2129-33.DOI: 10.1038/sj.bjc.6602645.
405	Lorenzen S 2007	Single-arm trial	Lorenzen S, Hentrich M, Haberl C,et al.Split-dose docetaxel, cisplatin and leucovorin/fluorouracil as first-line therapy in advanced gastric cancer and adenocarcinoma of the gastroesophageal junction: results of a phase II trial.Ann Oncol. 2007 Oct;18(10):1673-9. Epub 2007 Jul 28.
406	Lorenzen S 2012	Single-arm trial	Lorenzen S, Blank S, Lordick F, et al. Prediction of response and prognosis by a score including only pretherapeutic parameters in 410 neoadjuvant treated gastric cancer patients. Ann Surg Oncol. 2012 Jul;19(7):2119-27. doi: 10.1245/s10434-012-2254-1.
407	Lorenzen S 2013	Population inconformity	Lorenzen S, Pauligk C, Homann N,et al.Feasibility of perioperative chemotherapy with infusional 5-FU, leucovorin, and oxaliplatin with (FLOT) or without (FLO) docetaxel in elderly patients with locally advanced esophagogastric cancer.Br J Cancer. 2013 Feb 19;108(3):519-26. doi: 10.1038/bjc.2012.588. Epub 2013 Jan 15.
408	Lorenzen S 2013	Repeated article	Lorenzen S, Pauligk C, Homann N, Schmalenberg H, Jäger E, Al-Batran SE. Feasibility of perioperative chemotherapy with infusional 5-FU, leucovorin, and oxaliplatin with (FLOT) or without (FLO) docetaxel in elderly patients with locally advanced esophagogastric cancer. BRIT J CANCER. 2013 2013-01-01;108(3):519-26.
409	Lorenzen S 2016	Single-arm trial	Lorenzen S, Lordick F. Advanced gastric cancer: Status quo of systemic therapy and influence on the quality of life. ONKOLOGE. 2016 2016-01-01;22(6):406-12.
410	Lu B 2015	Population inconformity	Lu B, Bao LB, Sun Z, Hua ZL, Wang X, Qu CP. Efficacy and safety of capecitabine as maintenance therapy after capecitabine-based combination chemotherapy for patients with advanced esophagogastric junction adenocarcinoma. EUR REV MED PHARMACO. 2015 2015-01-01;19(19):3605-12.
411	Lu M 2016	Repeated article	Lu M, Wang T, Wang J. Effects of paclitaxel liposome and capecitabine in the treatment of advanced gastric cancer by clinical observation.Int J Clin Pharmacol Ther. 2016 Sep;54(9):693-7. doi: 10.5414/CP202568.
412	Luo Y 2012	Study design inconformity	Luo Y, Ge R, Shen C, Qi Y, Wang L, Mao Z. Individualized chemotherapeutic regimen with docetaxel, cisplatin, and 5-FU guided by combined detection of ERCC1, TUBB3, and TYMS genes in patients with advanced gastric cancer. Chinese Journal of Clinical Oncology. 2012 2012-01-01;39(22):1787-91.

413	Ma H 2014	Repeated article	Ma H, Liu L, Li J, Zhang F, Yang H. The clinical curative efficacy of docetaxel plus S-1 in the treatment of advanced gastric cancer. Anti-Tumor Pharmacy. 2014 2014-01-01;4(4):298-301.
414	Ma J 2015	Repeated article	Ma J, Yao S, Li XS,et al. Neoadjuvant Therapy of DOF Regimen Plus Bevacizumab Can Increase Surgical Resection Ratein Locally Advanced Gastric Cancer: A Randomized, Controlled Study.Medicine (Baltimore). 2015 Oct;94(42):e1489. doi: 10.1097/MD.0000000000001489.
415	Ma J 2015	Review	Ma J, Yao S, Li XS, Kang HR, Yao FF, Du N. Neoadjuvant therapy of DOF Regimen plus bevacizumab can increase surgical resection rate in locally advanced gastric cancer: A randomized, controlled study. Medicine (United States). 2015 2015-01-01;94(42):e1489.
416	Ma Q 2014	Repeated article	Ma Q, Qu Y, Tang Y. Clinical outcomes of advanced gastric cancer patients treated with chemotherapy of paclitaxel liposome combined with S-l or oxaliplatin. Chinese Journal of Clinical Oncology. 2014 2014-01-01;41(3):200-3.
417	Machida N 2012	Single-arm trial	Machida N, Hironaka S, Shinozaki K, et al. Randomized phase iii study of irinotecan (CPT-11) versus weekly paclitaxel (WPTX) for advanced gastric cancer (AGC) refractory to combination chemotherapy (CT) of fluoropyrimidine plus platinum (FP): WJOG4007 trial. ANN ONCOL. 2012 2012-01-01;23:i7.
418	Maeng CH 2013	Single-arm trial	Maeng CH, Yi JH, Lee J, et al. Effects of single nucleotide polymorphisms on treatment outcomes and toxicity in patients treated with sunitinib. ANTICANCER RES. 2013 2013-01-01;33(10):4619-26.
419	Mahipal A 2015	Review	Mahipal A, Choi M, Kim R. Second-line treatment of advanced gastric cancer: Where do we stand? JNCCN Journal of the National Comprehensive Cancer Network. 2015 2015-01-01;13(10):1281-92.
420	Mai M 1999	Single-arm trial	Mai M, Sakata Y, Kanamaru R,et al. A late phase II clinical study of RP56976 (docetaxel) in patients with advanced or recurrent gastric cancer: a cooperative study group trial (group B). Gan To Kagaku Ryoho. 1999 Mar;26(4):487-96.
421	Maiello E 2011	Repeated article	Maiello E, De Vita F, Gebbia V, et al. Epirubicin (E) in combination with cisplatin (CDDP) and capecitabine (C) versus docetaxel (D) combined with 5-fluorouracil (5-FU) by continuous infusion as front-line therapy in patients with advanced gastric cancer (AGC): Preliminary results of a randomized phase II trial of the Gruppo Oncologico Dell'Italia Meridionale. J CLIN ONCOL. 2011 2011-01-01;29(4).
422	Makatsoris T 2007	Single-arm trial	Makatsoris T, Papakostas P, Kalofonos HP, et al. Intensive weekly chemotherapy with docetaxel, epirubicin and carboplatin with G-CSF support in patients with advanced gastric cancer: A Hellenic Cooperative Oncology Group (HeCOG) phase II study. MED ONCOL. 2007 2007-01-01;24(3):301-7.

423	Malik I 2010	Single-arm trial	Malik I, Bernal P, Byrd J. A phase I study of docetaxel, oxaliplatin, & capecitabine (DOC) as first-line therapy of patients with locally advanced or metastatic adenocarcinoma of stomach and GE junction. Cancer Invest. 2010 Oct;28(8):833-8. doi: 10.3109/07357901003630942.
424	Malingré MM 2001	Single-arm trial	Malingré MM, Beijnen JH, Rosing H, et al. The effect of different doses of cyclosporin A on the systemic exposure of orally administered paclitaxel. Anticancer Drugs. 2001 Apr;12(4):351-8.
425	Malingré MM 2001	Single-arm trial	Malingré MM, Beijnen JH, Rosing H, et al. The effect of different doses of cyclosporin A on the systemic exposure of orally administered paclitaxel. ANTI-CANCER DRUG. 2001 2001-01-01;12(4):351-8.
426	Mansour JC 2007	Single-arm trial	Mansour JC, Tang L, Shah M, et al. Does graded histologic response after neoadjuvant chemotherapy predict survival for completely resected gastric cancer? ANN SURG ONCOL. 2007 2007-01-01;14(12):3412-8.
427	Mao ZY 2015	Repeated article	Mao ZY, Guo XC, Su D, et al. Retrospective evaluation of the efficacy of first-line treatment of advanced gastric cancer with docetaxel and oxaliplatin. Cancer Invest. 2015 Jan;33(1):16-21. doi: 10.3109/07357907.2014.988342.
428	Mao ZY 2015	Review	Mao ZY, Guo XC, Su D, Wang LJ, Zhang TT, Bai L. Retrospective evaluation of the efficacy of first-line treatment of advanced gastric cancer with docetaxel and oxaliplatin. CANCER INVEST. 2015 2015-01-01;33(1):16-21.
429	Marcelo G 2014	Population inconformity	Marcelo G, María S, Paula F, et al. Evaluation of docetaxel, cisplatin and capecitabine (DCX) as perioperative chemotherapy for resectable gastric and esophago-gastric cancer. ANN ONCOL. 2014 2014-01-01;25:i26-7.
430	Martin RP 2013	Single-arm trial	Martin RP, Arbea L, Chopitea A, et al. A three-step neoadjuvant strategy for locally advanced gastric cancer: A single institutional experience. EUR J CANCER. 2013 2013-01-01;49:S589.
431	Maruta F 2007	Repeated article	Maruta F, Ishizone S, Hiraguri M, et al. A clinical study of docetaxel with or without 5'DFUR as a second-line chemotherapy for advanced gastric cancer. Med Oncol. 2007;24(1):71-5. PMID: 17673814.
432	Maruta F 2007	Repeated article	Maruta F, Ishizone S, Hiraguri M, et al. A clinical study of docetaxel with or without 5′DFUR as a second-line chemotherapy for advanced gastric cancer. MED ONCOL. 2007 2007-01-01;24(1):71-5.
433	Massa E 2009	Single-arm trial	Massa E, Astara G, Madeddu C, et al. Palonosetron plus dexamethasone effectively prevents acute and delayed chemotherapy-induced nausea and vomiting following highly or moderately emetogenic chemotherapy in pre-treated patients who have failed to respond to a previous antiemetic treatment: Comparison between elderly and non-elderly patient response. Critical Reviews in Oncology/Hematology. 2009 2009-01-01;70(1):83-91.

434	Matano D 2017	Study design inconformity	Matano D, Moriwaki T, Tange Y, et al. Successful long-term management with a single administration of tri-weekly nab-paclitaxel in a patient with advanced gastric cancer with peritoneal dissemination. INTERNAL MED. 2017 2017-01-01;56(8):921-3.
435	Matsubara J 2011	Single-arm trial	Matsubara J, Shimada Y, Kato K, et al. Phase II study of bolus 5-fluorouracil and leucovorin combined with weekly paclitaxel as first-line therapy for advanced gastric cancer. Phase II study of bolus 5-fluorouracil and leucovorin combined with weekly paclitaxel as first-line therapy for advanced gastric cancer.
436	Matsubara J 2011	Single-arm trial	Matsubara J, Shimada Y, Kato K, et al. Phase II study of bolus 5-fluorouracil and leucovorin combined with weekly paclitaxel as first-line therapy for advanced gastric cancer. ONCOLOGY-BASEL. 2011 2011-01-01;81(5-6):291-7.
437	Matsutani T 2012	Single-arm trial	Matsutani T, Yoshida H, Sasajima K, et al. A successful resected case of advanced esophageal cancer with early gastric cancer responding to neoadjuvant chemotherapy of docetaxel, CDDP and 5-FU. Japanese Journal of Cancer and Chemotherapy. 2012 2012-01-01;39(4):645-8.
438	Matula K 2015	Study design inconformity	Matula K, Collie-Duguid E, Murray G, et al. Regulation of cellular sphingosine-1-phosphate by sphingosine kinase 1 and sphingosine-1-phopshate lyase determines chemotherapy resistance in gastroesophageal cancer. BMC CANCER. 2015 2015-01-01;15(1).
439	Maugeri-Saccà M 2013	Study design inconformity	Maugeri-Saccà M, Pizzuti L, Sergi D, et al. FOLFIRI as a second-line therapy in patients with docetaxel-pretreated gastric cancer: a historical cohort.J Exp Clin Cancer Res. 2013 Sep 17;32:67. doi: 10.1186/1756-9966-32-67.
440	Maugeri-Saccà M 2013	Single-arm trial	Maugeri-Saccà M, Pizzuti L, Sergi D, et al. FOLFIRI as a second-line therapy in patients with docetaxel-pretreated gastric cancer: A historical cohort. Journal of Experimental and Clinical Cancer Research. 2013 2013-01-01;32(1).
441	Mavroudis D 2000	Single-arm trial	Mavroudis D, Kourousis C, Androulakis N,et al. Frontline treatment of advanced gastric cancer with docetaxel and granulocyte colony-stimulating factor (G-CSF): a phase II trial. Am J Clin Oncol. 2000 Aug;23(4):341-4.
442	Mayer RJ 2001	Population inconformity	Mayer RJ. Efficacy of neo- and adjuvant treatment modalities in gastrointestinal cancer patients. Swiss Surgery. 2001 2001-01-01;7(6):239-42.
443	Men HT 2016	Single-arm trial	Men HT, Gou HF, Liu JY, et al. Prognostic factors of intraperitoneal chemotherapy for peritoneal carcinomatosis of gastric cancer: A retrospective study from a single center. ONCOL LETT. 2016 2016-01-01;11(5):3501-7.
444	Meulendijks D 2016	Single-arm trial	Meulendijks D, Beerepoot LV, Boot H, et al. Trastuzumab and bevacizumab combined with docetaxel, oxaliplatin and capecitabine as first-line treatment of advanced HER2-positive gastric cancer: a multicenter phase II study. Invest New Drugs. 2016 Feb;34(1):119-28. doi: 10.1007/s10637-015-0309-4.

445	Meulendijks D 2016	Single-arm trial	Meulendijks D, de Groot JW, Los M, et al. Bevacizumab combined with docetaxel, oxaliplatin, and capecitabine, followed by maintenance with capecitabine and bevacizumab, as first-line treatment of patients with advanced HER2-negative gastric cancer: A multicenter phase 2 study. Cancer. 2016 May 1;122(9):1434-43. doi: 10.1002/cncr.29864.
446	Meulendijks D 2016	Single-arm trial	Meulendijks D, De Groot JWB, Los M, et al. Bevacizumab combined with docetaxel, oxaliplatin, and capecitabine, followed by maintenance with capecitabine and bevacizumab, as first-line treatment of patients with advanced HER2-negative gastric cancer: A multicenter phase 2 study. CANCER-AM CANCER SOC. 2016 2016-01-01;122(9):1434-43.
447	Meulendijks D 2016	Single-arm trial	Meulendijks D, Beerepoot LV, Boot H, et al. Trastuzumab and bevacizumab combined with docetaxel, oxaliplatin and capecitabine as first-line treatment of advanced HER2-positive gastric cancer: A multicenter phase II study. INVEST NEW DRUG. 2016 2016-01-01;34(1):119-28.
448	Meza-Junco J 2009	Single-arm trial	Meza-Junco J, Au HJ, Sawyer MB. Trastuzumab for gastric cancer Meza-Junco, Au & Sawyer Trastuzumab. EXPERT OPIN BIOL TH. 2009 2009-01-01;9(12):1543-51.
449	Mezhir JJ 2010	Single-arm trial	Mezhir JJ, Pillarisetty VG, Shah MA, Coit DG. Randomized Clinical Trials in Gastric Cancer. SURG ONCOL CLIN N AM. 2010 2010-01-01;19(1):81-100.
450	Mitsui Y 2015	Single-arm trial	Mitsui Y, Sato Y, Miyamoto H, et al. Trastuzumab in combination with docetaxel/cisplatin/S-1 (DCS) for patients with HER2-positive metastatic gastric cancer: feasibility and preliminary efficacy. Cancer Chemother Pharmacol. 2015 Aug;76(2):375-82. doi:10.1007/s00280-015-2807-7.
451	Miura T 2010	Single-arm trial	Miura T, Nakamura J, Yamada S,et al. Retrospective analysis of generalized chemotherapy for unresectable advanced gastric cancer. Gan To Kagaku Ryoho. 2010 Jan;37(1):77-81.
452	Miura T 2010	Single-arm trial	Miura T, Nakamura J, Yamada S, et al. Retrospective analysis of generalized chemotherapy for unresectable advanced gastric cancer. Japanese Journal of Cancer and Chemotherapy. 2010 2010-01-01;37(1):77-81.
453	Miura T 2011	Single-arm trial	Miura T, Hosojima Y, Nakamura J,et al. Palliative anti-cancer chemotherapy is safely executable in a hemodialytic patient with unresectable advanced gastric cancer. Gan To Kagaku Ryoho. 2011 Mar;38(3):453-6.
454	Miyamoto K 2013	Interventions inconformity	Miyamoto K, Koyama T, Ohishi T, et al. Strategy for the treatment of Stage IV gastric cancer with preoperative chemotherapy. Gan To Kagaku Ryoho. 2013 Nov;40(12):1618-20.
455	Mizutani S 2006	Single-arm trial	Mizutani S, Oyama T, Hatanaka N, et al. Combined chemotherapy with weekly Paclitaxel and doxifluridine for advanced and recurrent gastric cancers. Gan to kagaku ryoho. Cancer & chemotherapy. 2006 2006-01-01;33(3):327-31.

456	Mochiki E 2006	Single-arm trial	Mochiki E, Ohno T, Kamiyama Y, et al. Phase I/II study of S-1 combined with paclitaxel in patients with unresectable and/or recurrent advanced gastric cancer.Br J Cancer. 2006 Dec 18;95(12):1642-7. Epub 2006 Nov 28.DOI: 10.1038/sj.bjc.6603497
457	Mochiki E 2012	Repeated article	Mochiki E, Ogata K, Ohno T, et al. Phase II multi-institutional prospective randomised trial comparing S-1+paclitaxel with S-1+cisplatin in patients with unresectable and/or recurrent advanced gastric cancer. Br J Cancer. 2012 Jun 26;107(1):31-6. doi: 10.1038/bjc.2012.222. Epub 2012 May 22.
458	Mochiki E 2012	Repeated article	Mochiki E, Ogata K, Ohno T, et al. Phase II multi-institutional prospective randomised trial comparing S-1paclitaxel with S-1cisplatin in patients with unresectable and/or recurrent advanced gastric cancer. BRIT J CANCER. 2012 2012-01-01;107(1):31-6.
459	Moehler M 2013	Population inconformity	Moehler M, Gockel I, Roessler HP, et al. Prospective, open, multi-centre phase I/II trial to assess safety and efficacy of neoadjuvant radiochemotherapy with docetaxel and oxaliplatin in patients with adenocarcinoma of the oesophagogastric junction.BMC Cancer. 2013 Feb 11;13:75. doi: 10.1186/1471-2407-13-75.
460	Molina R 2013	Single-arm trial	Molina R, Lamarca A, Mart ńez-Amores B, et al. Perioperative chemotherapy for resectable gastroesophageal cancer: A single-center experience. European Journal of Surgical Oncology. 2013 2013-01-01;39(8):814-22.
461	Morant R 2001	Review	Morant R. Neoadjuvant and adjuvant chemotherapy of locally advanced stomach cancer. ONKOLOGIE. 2001 2001-01-01;24(2):116-21.
462	Morgan RJ Jr 2003	Population inconformity	Morgan RJ Jr(1), Doroshow JH, Synold T, et al.Phase I trial of intraperitoneal docetaxel in the treatment of advanced malignancies primarily confined to the peritoneal cavity: dose-limiting toxicity and pharmacokinetics.Clin Cancer Res. 2003 Dec 1;9(16 Pt 1):5896-901.
463	Morita S 2006	Repeated article	Morita S, Sakamoto J. Application of an adaptive design to a randomized phase II selection trial in gastric cancer: A report of the study design. PHARM STAT. 2006 2006-01-01;5(2):109-18.
464	Morita S 2007	Study design inconformity	Morita S, Baba H, Tsuburaya A,et al. A randomized phase II selection trial in patients with advanced/recurrent gastric cancer: Trial for Advanced Stomach Cancer (TASC).Jpn J Clin Oncol. 2007 Jun;37(6):469-72. Epub 2007 Jun 21.
465	Morita S 2007	Single-arm trial	Morita S, Baba H, Tsuburaya A, et al. A randomized phase II selection trial in patients with advanced/recurrent gastric cancer: Trial for advanced stomach cancer (TASC). JPN J CLIN ONCOL. 2007 2007-01-01;37(6):469-72.
466	Moriwaki T 2005	Single-arm trial	Moriwaki T, Hyodo I, Nishina T, et al. A phase I study of doxifluridine combined with weekly paclitaxel for metastatic gastric cancer. Cancer Chemother Pharmacol. 2005 Aug;56(2):138-44.

467	Moriwaki T 2014	Repeated article	Moriwaki T, Hirai S, Hironaka S, et al. A randomized phase II study comparing S-1 plus weekly split-dose cisplatin with S-1 plus standard-dose cisplatin as first-line chemotherapy for advanced gastric cancer. Gastric Cancer. 2014 Apr;17(2):354-61. doi: 10.1007/s10120-013-0284-4.
468	Morley R 2015	Review	Morley R, Cardenas A, Hawkins P, et al. Safety of onartuzumab in patients with solid tumors: Experience to date from the onartuzumab clinical trial program. PLOS ONE. 2015 2015-01-01;10(10).
469	Motamed K 2015	Population inconformity	Motamed K, Said N. Polymeric albumin-free paclitaxel intraperitoneal therapy demonstrates superior efficacy over nab-paclitaxel intravenous therapy in a mouse model of metastatic ovarian cancer. CANCER RES. 2015 2015-01-01;75(15).
470	Mu Y 2016	Study design inconformity	Mu Y, Wang WH, Xie JP, Zhang YX, Yang YP, Zhou CH. Efficacy and safety of cord blood-derived dendritic cells plus cytokine-induced killer cells combined with chemotherapy in the treatment of patients with advanced gastric cancer: A randomized phase II study. ONCOTARGETS THER. 2016 2016-01-01;9:4617-27.
471	Munakata M 2008	Review	Munakata M, Sakata Y. RCT makes standalization of chemotherapy for advanced gastric cancers including outpatient-based chemotherapy. Japanese Journal of Gastroenterology. 2008 2008-01-01;105(3):351-60.
472	Murad AM 1999	Single-arm trial	Murad AM, Petroianu A, Guimaraes RC, et al. Phase II trial of the combination of paclitaxel and 5-fluorouracil in the treatment of advanced gastric cancer: a novel, safe, and effective regimen. Am J Clin Oncol. 1999 Dec;22(6):580-6.
473	Murad AM 1999	Single-arm trial	Murad AM, Petroianu A, Guimaraes RC, Aragao BC, Cabral LOM, Scalabrini-Neto AO. Phase II trial of the combination of paclitaxel and 5-fluorouracil in the treatment of advanced gastric cancer: A novel, safe, and effective regimen. American Journal of Clinical Oncology: Cancer Clinical Trials. 1999 1999-01-01;22(6):580-6.
474	Murad AM 2006	Single-arm trial	Murad AM, Skare NG, Vinholes J, et al. Phase II multicenter trial of docetaxel, epirubicin, and 5-fluorouracil (DEF) in the treatment of advanced gastric cancer: a novel, safe, and active regimen. Gastric Cancer. 2006;9(2):99-105.doi: 10.1007/s10120-006-0361-z
475	Muranaka T 2016	Single-arm trial	Muranaka T, Yuki S, Sawada K, et al. Phase II trial of bolus 5-FU/l-LV regimen as salvage line chemotherapy for oral fluorouracil resistant unresectable gastric cancer (HGCSG1502). J CLIN ONCOL. 2016 2016-01-01;34(4).
476	Muro K 2016	Repeated article	Muro K, Oh SC, Shimada Y, et al. Subgroup analysis of East Asians in RAINBOW: A phase 3 trial of ramucirumab plus paclitaxel for advanced gastric cancer. J Gastroenterol Hepatol. 2016 Mar;31(3):581-9. doi: 10.1111/jgh.13153.
477	Nagata N 2005	Single-arm trial	Nagata N, Kobayashi M, Kojima H, et al. Phase I study of paclitaxel and cisplatin for patients with advanced or recurrent gastric cancer. HEPATO-GASTROENTEROL. 2005 2005-01-01;52(66):1905-10.

478	Naitoh H 2014	Repeated article	Naitoh H, Yamamoto H, Murata S, et al.Stratified phase II trial to establish the usefulness of the collagen gel droplet embedded culture-drug sensitivity test (CD-DST) for advanced gastric cancer.Gastric Cancer. 2014 Oct;17(4):630-7. doi: 10.1007/s10120-013-0320-4. Epub 2013 Dec 8.
479	Nakamura M 2012	Repeated article	Nakamura M, Shitara K, Yuki S, et al. Preliminary safety data from randomized phase II study comparing dose-escalated weekly paclitaxel versus standard-dose weekly paclitaxel for patients with previously treated advanced gastric cancer. ANN ONCOL. 2012 2012-01-01;23:i99.
480	Nakamura R 2006	Study design inconformity	Nakamura R, Saikawa Y, Kubota T,et al. Role of the MTT chemosensitivity test in the prognosis of gastric cancer patients after postoperative adjuvant chemotherapy.Anticancer Res. 2006 Mar-Apr;26(2B):1433-7.
481	Nakanishi K 2016	Repeated article	Nakanishi K, Kobayashi D, Mochizuki Y, et al. Phase II multi-institutional prospective randomized trial comparing S-1 plus paclitaxel with paclitaxel alone as second-line chemotherapy in S-1 pretreated gastric cancer (CCOG0701). INT J CLIN ONCOL. 2016 2016-01-01;21(3):557-65.
482	Narahara H 2004	Single-arm trial	Narahara H, Sugimoto N, Iishi H, et al. CPT-11 combined chemotherapy for metastatic gastric cancer. Gan To Kagaku Ryoho. 2004 Nov;31(12):1973-7.
483	Narimanov M 2012	Single-arm trial	Narimanov M, Tryakin A, Bazin I, Kanagavel D, Tjulandin S. Combination of docetaxel, cisplatin, and capecitabine (DCX) as a first-line chemotherapy in patients with metastatic gastric carcinoma: A phase II study. J CLIN ONCOL. 2012 2012-01-01;30(15).
484	Narimanov M 2013	Repeated article	Narimanov M, Tryakin A, Zarkua V, Bazin I, Garin A, Tjulandin S. Cisplatin and capecitabine with and without docetaxel as a first-line chemotherapy in patients with metastatic gastric carcinoma: Single clinical experience. J CLIN ONCOL. 2013 2013-01-01;31(15).
485	Nawasreh M 2008	Study design inconformity	Nawasreh M. Chemo-, regio-, and stereoselectivity of F-ring opening reactions in the cephalostatin series. Bioorganic and Medicinal Chemistry. 2008 2008-01-01;16(1):255-65.
486	Nedergaard MK 2012	Study design inconformity	Nedergaard MK, Hedegaard CJ, Poulsen HS. Targeting the epidermal growth factor receptor in solid tumor malignancies. BIODRUGS. 2012 2012-01-01;26(2):83-99.
487	Newton AD 2015	Review	Newton AD, Datta J, Loaiza-Bonilla A, Karakousis GC, Roses RE. Neoadjuvant therapy for gastric cancer: Current evidence and future directions. Journal of Gastrointestinal Oncology. 2015 2015-01-01;6(5):534-43.

488	Ninomiya M 2007	Single-arm trial	Ninomiya M, Kondo K, Matsuo K,et al. Multicenter phase II trial of combination chemotherapy with weekly paclitaxel and 5-fluorouracil for the treatment of advanced or recurrent gastric carcinoma. J Chemother. 2007 Aug;19(4):444-50. doi: 10.1179/joc.2007.19.4.444.
489	Nishikawa K 2012	Repeated article	Nishikawa K, Morita S, Matsui T, et al. A randomized phase-II trial comparing sequential and concurrent paclitaxel with oral or parenteral fluorinated pyrimidines for advanced or metastatic gastric cancer. Gastric Cancer. 2012 Oct;15(4):363-9. doi: 10.1007/s10120-011-0124-3.
490	Nishikawa K 2012	Repeated article	Nishikawa K, Morita S, Matsui T, et al. A randomized phase-II trial comparing sequential and concurrent paclitaxel with oral or parenteral fluorinated pyrimidines for advanced or metastatic gastric cancer. GASTRIC CANCER. 2012 2012-01-01;15(4):363-9.
491	Nishikawa K 2016	Repeated article	Nishikawa K, Yoshikawa T, Fujitani K, et al. Early results of a randomized two-by-two factorial phase II trial comparing neoadjuvant chemotherapy with 2 and 4 courses of cisplatin/S-1 (CS) and docetaxel/cisplatin/S-1 (DCS) as neoadjuvant chemotherapy for locally advanced gastric cancer. ANN ONCOL. 2016 2016-01-01;27.
492	Nishina T 2011	Single-arm trial	Nishina T, Sasaki Y, Yasui H, et al. Phase II study of abi-007 given as an every three weeks schedule for Japanese patients with unresectable or recurrent gastric cancer refractory to 5-fluorouracil (5-FU) containing regimen. EUR J CANCER. 2011 2011-01-01;47:S465.
493	Nishina T 2011	Single-arm trial	Nishina T, Takiuchi H, Boku N, et al. Randomized phase II study of second-line chemotherapy with best-available 5-fluorouracil (5-FU) versus weekly paclitaxel in far advanced gastric cancer (AGC) with peritoneal metastasis (PM) refractory to 5-Fu-containing regimens (JCOG0407). ANN ONCOL. 2011 2011-01-01;22:x60-1.
494	Nishina T 2012	Single-arm trial	Nishina T, Hironaka S, Tsuji A, et al. Final analysis of randomized phase III study WJOG4007 comparing irinotecan (CPT-11) with weekly paclitaxel (WPTX) in advanced gastric cancer (AGC) refractory to chemotherapy (CT) of fluoropyrimidine plus platinum (FP). ANN ONCOL. 2012 2012-01-01;23:x233.
495	Nishina T 2016	Repeated article	Nishina T, Boku N, Gotoh M, et al. Randomized phase II study of second-line chemotherapy with the best available 5-fluorouracil regimen versus weekly administration of paclitaxel in far advanced gastric cancer with severe peritoneal metastases refractory to 5-fluorouracil-containing regimens (JCOG0407). GASTRIC CANCER. 2016 2016-01-01;19(3):902-10.
496	Nishiyama M 2009	Review	Nishiyama M, Wada S. Docetaxel: its role in current and future treatments for advanced gastric cancer. Gastric Cancer. 2009;12(3):132-41. doi: 10.1007/s10120-009-0521-z.

497	Noh SH 2014	Repeated article	Noh SH, Park SR, Yang HK, et al. Adjuvant capecitabine plus oxaliplatin for gastric cancer after D2 gastrectomy (CLASSIC): 5-year follow-up of an open-label, randomised phase 3 trial. The Lancet Oncology. 2014 2014-01-01;15(12):1389-96.
498	Ochenduszko S 2015	Repeated article	Ochenduszko S, Puskulluoglu M, Konopka K, et al. Comparison of efficacy and safety of first-line palliative chemotherapy with EOX and mDCF regimens in patients with locally advanced inoperable or metastatic HER2-negative gastric or gastroesophageal junction adenocarcinoma: a randomized phase 3 trial. MED ONCOL. 2015 2015-01-01;32(10).
499	Ogawa M 1996	Review	Ogawa M. New systemic drugs in the treatment of gastrointestinal cancer. Curr Opin Oncol. 1996 Jul;8(4):317-20.
500	Oh SC 2015	Repeated article	Oh SC, Ryu MH, Park SH, et al. A phase III study to compare efficacy and safety of DHP107 (oral paclitaxel) versus IV paclitaxel in patients with metastatic or recurrent gastric cancer after failure of first-line chemotherapy (DREAM). J CLIN ONCOL. 2015 2015-01-01;33(15).
501	Ohtsu A 2002	Single-arm trial	Ohtsu A. Recent advances on chemotherapy for advanced gastric cancer. Biotherapy. 2002 2002-01-01;16(4):288-94.
502	Ohtsu A 2016	Single-arm trial	Ohtsu A, Tabernero J, Bang YJ, et al. Pembrolizumab versus paclitaxel as second-line therapy for advanced gastric or gastroesophageal junction (GEJ) adenocarcinoma: Phase 3 KEYNOTE-061 study. J CLIN ONCOL. 2016 2016-01-01;34.
503	Okazaki S 2013	Repeated article	Okazaki S, Nakajima TE, Hashimoto J, et al. A feasibility study of outpatient chemotherapy with S-1 + cisplatin in patients with advanced gastric cancer. GASTRIC CANCER. 2013 2013-01-01;16(1):41-7.
504	Oki E 2014	Single-arm trial	Oki E, Emi Y, Kusumoto T,et al. Phase II study of docetaxel and S-1 (DS) as neoadjuvant chemotherapy for clinical stage III resectable gastric cancer. Ann Surg Oncol. 2014 Jul;21(7):2340-6. doi: 10.1245/s10434-014-3594-9.
505	Okines A 2008	Single-arm trial	Okines A, Chau I, Cunningham D. Capecitabine in gastric cancer. DRUG TODAY. 2008 2008-01-01;44(8):629-40.
506	Okines AFC 2010	Review	Okines AFC, Cunningham D. Trastuzumab in gastric cancer. EUR J CANCER. 2010 2010-01-01;46(11):1949-59.
507	Okines AFC 2012	Population inconformity	Okines AFC, Cunningham D. Trastuzumab: A novel standard option for patients with HER-2-positive advanced gastric or gastro-oesophageal junction cancer. Therapeutic Advances in Gastroenterology. 2012 2012-01-01;5(5):301-18.
508	Okuyama T 2011	Single-arm trial	Okuyama T, Korenaga D, Koushi K, et al. The prognostic significance of chemotherapy for stage IV gastric cancer patients: A single-institution experience. SURG TODAY. 2011 2011-01-01;41(7):935-40.

509	Oppedijk V 2014	Repeated article	Oppedijk V, van der Gaast A, van Lanschot JJ, et al. Patterns of recurrence after surgery alone versus preoperative chemoradiotherapy and surgery in the CROSS trials. J Clin Oncol. 2014 Feb 10;32(5):385-91. doi: 10.1200/JCO.2013.51.2186.
510	Oshima T 2012	Single-arm trial	Oshima T, Kunisaki C, Sato T, et al. Usefulness of multidisciplinary therapy combining neoadjuvant chemotherapy with S-1 plus cisplatin and postoperative sequential chemotherapy in patients with scirrhous gastric cancer. Hepatogastroenterology. 2012 Jul-Aug;59(117):1638-42. doi: 10.5754/hge11704.
511	Ou SHI 2007	Single-arm trial	Ou SHI, Holcombe RF. Capecitabine in advanced gastric or oesophagogastric cancer: A viewpoint by Sai-Hong Ignatius Ou and Randall F. Holcombe. DRUGS. 2007 2007-01-01;67(4):611.
512	Oyan B 2014	Single-arm trial	Oyan B, Eren OO, Sonmez O. Capecitabine maintenance after first-line induction chemotherapy with docetaxel, cisplatin, and fluorouracil in patients with advanced gastric cancer. J CLIN ONCOL. 2014 2014-01-01;32(15).
513	Özdemir N 2013	Unavailable	Özdemir N, Aksoy S, Eren T, Abali H, Oksuzoglu OB, Zengin N. Retrospective review of modified dose docetaxel, cisplatin, and 5-flourouracil (DCF) for the treatment of first-line metastatic gastric carcinomas. J CLIN ONCOL. 2013 2013-01-01;31(15).
514	Ozdemir N 2014	Single-arm trial	Ozdemir N, Abali H, Vural M,et al. Docetaxel, cisplatin, and fluorouracil combination in neoadjuvant setting in the treatment of locally advanced gastric adenocarcinoma: Phase II NEOTAX study. Cancer Chemother Pharmacol. 2014 Dec;74(6):1139-47. doi:10.1007/s00280-014-2586-6.
515	Özdemir N 2014	Single-arm trial	Özdemir N, Abali H, Vural M, et al. Docetaxel, cisplatin, and fluorouracil combination in neoadjuvant setting in the treatment of locally advanced gastric adenocarcinoma: A phase II NEOTAX study. J CLIN ONCOL. 2014 2014-01-01;32(15).
516	Paoletti X 2010	Study design inconformity	Paoletti X, Oba K, Burzykowski T, et al. Benefit of adjuvant chemotherapy for resectable gastric cancer: A meta-analysis. Journal of the American Medical Association. 2010 2010-01-01;303(17):1729-37.
517	Park I 2013	Single-arm trial	Park I, Ryu MH, Choi YH,et al. A phase II study of neoadjuvant docetaxel, oxaliplatin, and S-1 (DOS) chemotherapy followed by surgery and adjuvant S-1 chemotherapy in potentially resectable gastric or gastroesophageal junction adenocarcinoma. Cancer Chemother Pharmacol. 2013 Oct;72(4):815-23. doi:10.1007/s00280-013-2257-z.
518	Park I 2013	Single-arm trial	Park I, Ryu MH, Choi YH, et al. A phase II study of neoadjuvant docetaxel, oxaliplatin, and S-1 (DOS) chemotherapy followed by surgery and adjuvant S-1 chemotherapy in potentially resectable gastric or gastroesophageal junction adenocarcinoma. CANCER CHEMOTH PHARM. 2013 2013-01-01;72(4):815-23.

519	Park JC 2010	Single-arm trial	Park JC, Lee YC, Kim JH, et al. ATP-based chemotherapy response assay based combination chemotherapy for unresectable advanced gastric cancer: Interim report of prospective controlled study. GASTROENTEROLOGY. 2010 2010-01-01;138(5):S503.
520	Park JC 2012	Single-arm trial	Park JC, Lee JH, Cheoi K, et al. Predictive value of pretreatment metabolic activity measured by fluorodeoxyglucose positron emission tomography in patients with metastatic advanced gastric cancer: The maximal SUV of the stomach is a prognostic factor. EUR J NUCL MED MOL I. 2012 2012-01-01;39(7):1107-16.
521	Park KW 2007	Single-arm trial	Park KW, Ahn JS, Park YS, et al. Phase II study of docetaxel and cisplatin combination chemotherapy in metastatic gastric cancer. CANCER CHEMOTH PHARM. 2007 2007-01-01;59(1):17-21.
522	Park S 2010	Single-arm trial	Park S, Lee S, Kang J, et al. A feasibility analysis from the patient preference randomized phase III clinical trial of second-line chemotherapy (SLC) in advanced gastric cancer (AGC) patients pretreated with both fluoropyrimidines and platinum. J CLIN ONCOL. 2010 2010-01-01;28(15).
523	Park SH 2004	Single-arm trial	Park SH, Kang WK, Lee HR, et al. Docetaxel plus cisplatin as second-line therapy in metastatic or recurrent advanced gastric cancer progressing on 5-fluorouracil-based regimen. Am J Clin Oncol. 2004 Oct;27(5):477-80.
524	Park SH 2006	Single-arm trial	Park SH, Lee WK, Chung M, et al. Paclitaxel versus docetaxel for advanced gastric cancer: a randomized phase II trial in combination with infusional 5-fluorouracil. Anticancer Drugs. 2006 Feb;17(2):225-9.
525	Park SH 2008	Review	Park SH, Cho MS, Kim YS, et al. Self-reported health-related quality of life predicts survival for patients with advanced gastric cancer treated with first-line chemotherapy. QUAL LIFE RES. 2008 2008-01-01;17(2):207-14.
526	Park SH 2011	Repeated article	Park SH, Lim DH, Park K, et al. A multicenter, randomized phase III trial comparing second-line chemotherapy (SLC) plus best supportive care (BSC) with BSC alone for pretreated advanced gastric cancer (AGC). J CLIN ONCOL. 2011 2011-01-01;29(15).
527	Park SR 2004	Single-arm trial	Park SR, Oh DY, Kim DW, et al. A multi-center, late phase II clinical trial of Genexol (paclitaxel) and cisplatin for patients with advanced gastric cancer. Oncol Rep. 2004 Nov;12(5):1059-64.
528	Park SR 2008	Study design inconformity	Park SR, Lee JS, Kim CG,et al. Endoscopic ultrasound and computed tomography in restaging and predicting prognosis after neoadjuvant chemotherapy in patients with locally advanced gastric cancer.Cancer. 2008 Jun;112(11):2368-76. doi: 10.1002/cncr.23483.
529	Park SR 2008	Single-arm trial	Park SR, Kim HK, Kim CG, et al.Phase I/II study of S-1 combined with weekly docetaxel in patients with metastatic gastric carcinoma.Br J Cancer. 2008 Apr 22;98(8):1305-11. doi: 10.1038/sj.bjc.6604312. Epub 2008 Mar 25.

530	Park YH 2004	Single-arm trial	Park YH, Ryoo BY, Choi SJ, Kim HT. A phase II study of capecitabine and docetaxel combination chemotherapy in patients with advanced gastric cancer. BRIT J CANCER. 2004 2004-01-01;90(7):1329-33.
531	Pasini F 2011	Review	Pasini F, Fraccon AP, De Manzoni G. The role of chemotherapy in metastatic gastric cancer. ANTICANCER RES. 2011 2011-01-01;31(10):3543-54.
532	Peng YF 2015	Single-arm trial	Peng YF, Imano M, Itoh T,et al. A phase II trial of perioperative chemotherapy involving a single intraperitoneal administration of paclitaxel followed by sequential S-1 plus intravenous paclitaxel for serosa-positive gastric cancer. J Surg Oncol. 2015 Jun;111(8):1041-6. doi: 10.1002/jso.23928.
533	Peng YF 2015	Single-arm trial	Peng YF, Imano M, Itoh T, et al. A phase II trial of perioperative chemotherapy involving a single intraperitoneal administration of paclitaxel followed by sequential S-1 plus intravenous paclitaxel for serosa-positive gastric cancer. J SURG ONCOL. 2015 2015-01-01;111(8):1041-6.
534	Pernot S 2010	Population inconformity	Pernot S, Mitry E, Lepere C, et al. Biweekly docetaxel, fluorouracil, leucovorin, oxaliplatin (TFOX) for advanced gastric and oesophageal adenocarcinoma (AGEC): Tolerance and response in 38 patients: Preliminary report. ANN ONCOL. 2010 2010-01-01;21:i227-8.
535	Pernot S 2013	Repeated article	Pernot S, Hitier S, Rougier P, Van Cutsem E. Comparison of docetaxel, fluorouracil and oxaliplatin or cisplatin with other modified schedules: A new therapeutic index in advanced gastric cancer. ANN ONCOL. 2013 2013-01-01;24:v19-20.
536	Pernot S 2014	Single-arm trial	Pernot S, Mitry E, Samalin E, et al. Biweekly docetaxel, fluorouracil, leucovorin, oxaliplatin (TEF) as first-line treatment for advanced gastric cancer and adenocarcinoma of the gastroesophageal junction: safety and efficacy in a multicenter cohort. Gastric Cancer. 2014 Apr;17(2):341-7. doi: 10.1007/s10120-013-0266-6.
537	Pernot S 2014	Population inconformity	Pernot S, Mitry E, Samalin E, et al. Biweekly docetaxel, fluorouracil, leucovorin, oxaliplatin (TEF) as first-line treatment for advanced gastric cancer and adenocarcinoma of the gastroesophageal junction: Safety and efficacy in a multicenter cohort. GASTRIC CANCER. 2014 2014-01-01;17(2):341-7.
538	Petrioli R	Study design inconformity	Petrioli R, Roviello G, Zanotti L, et al. Epirubicin-based compared with docetaxel-based chemotherapy for advanced gastric carcinoma: A systematic review and meta-analysis. Critical Reviews in Oncology/Hematology. 2016 2016-01-01;102:82-8.
539	Petrioli R 2015	Single-arm trial	Petrioli R, Francini E, Roviello F, et al. Sequential treatment with epirubicin, oxaliplatin and 5FU (EOF) followed by docetaxel, oxaliplatin and 5FU (DOF) in patients with advanced gastric or gastroesophageal cancer: a single-institution experience. CANCER CHEMOTH PHARM. 2015 2015-01-01.

540	Petrioli R 2016	Review	Petrioli R, Roviello G, Zanotti L,et al. Epirubicin-based compared with docetaxel-based chemotherapy for advanced gastric carcinoma: A systematic review and meta-analysis. Crit Rev Oncol Hematol. 2016 Jun;102:82-8. doi:10.1016/j.critrevonc.2016.04.001.
541	Piacentini P 2012	Single-arm trial	Piacentini P, Durante E, Trolese A, Mercanti A, Bonetti A. Weekly Taxotere and cisplatin with continuous-infusion 5-fluoruracil for the treatment of advanced gastric and esophageal cancer: A prospective, observational, single-institution experience. GASTRIC CANCER. 2012 2012-01-01;15(1):106-10.
542	Pieters A 2008	Single-arm trial	Pieters A, Laurent S, Dero I, Van Damme N, Peeters M. The role of oral fluoropyrimidines in the treatment of advanced gastric cancer. ACTA GASTRO-ENT BELG. 2008 2008-01-01;71(4):361-6.
543	Pinto C 2009	Population inconformity	Pinto C, Di Fabio F, Barone C, et al. Phase II study of cetuximab in combination with cisplatin and docetaxel in patients with untreated advanced gastric or gastro-oesophageal junction adenocarcinoma (DOCETUX study). BRIT J CANCER. 2009 2009-01-01;101(8):1261-8.
544	Polyzos A 2005	Single-arm trial	Polyzos A, Syrigos K, Stergiou J, et al. Phase I trial of weekly docetaxel with a 4-weekly cisplatin administration in patients with advanced gastric carcinoma. Cancer Chemother Pharmacol. 2005 May;55(5):466-70.
545	Pozzo C 2008	Review	Pozzo C, Barone C.Is there an optimal chemotherapy regimen for the treatment of advanced gastric cancer that will provide a platform for the introduction of new biological agents? Oncologist. 2008 Jul;13(7):794-806. doi: 10.1634/theoncologist.2008-0082. Epub 2008 Jul 9.
546	Pozzo C 2008	Review	Pozzo C, Barone C. Is there an optimal chemotherapy regimen for the treatment of advanced gastric cancer that will provide a platform for the introduction of new biological agents? ONCOLOGIST. 2008 2008-01-01;13(7):794-806.
547	Prithviraj GK 2014	Population inconformity	Prithviraj GK, Baksh KA, Fulp WJ, et al. Carboplatin and paclitaxel as first-line treatment of unresectable or metastatic esophageal or gastric cancer. J CLIN ONCOL. 2014 2014-01-01;32(15).
548	Qi WX 2013	Review	Qi WX, Shen Z, Lin F, et al. Overall survival benefits for irinotecan-containing regimens as first-line treatment for advanced gastric cancer: An updated meta-analysis of ten randomized controlled trials. INT J CANCER. 2013 2013-01-01;132(2):E66-73.
549	Qiu ZQ 2015	Study design inconformity	Qiu ZQ, Qiu ZR.Sensitivity of gastric cancer cells to chemotherapy drugs in elderly patients and its correlation with cyclooxygenase-2 expression.Asian Pac J Cancer Prev. 2015;16(8):3447-50.

550	Qu JJ 2010	Repeated article	Qu JJ, Shi YR, Liu FR, Ma SQ, Ma FY. A clinical study of paclitaxel combined with FOLFOX4 regimen as neoadjuvant chemotherapy for advanced gastric cancer. Zhonghua Wei Chang Wai Ke Za Zhi. 2010 Sep;13(9):664-7.
551	Qu JJ 2010	Population inconformity	Qu JJ, Shi YR, Liu FR, Ma SQ, Ma FY. [A clinical study of paclitaxel combined with FOLFOX4 regimen as neoadjuvant chemotherapy for advanced gastric cancer]. Zhonghua wei chang wai ke za zhi = Chinese journal of gastrointestinal surgery. 2010 2010-01-01;13(9):664-7.
552	Qu XL 2011	Repeated article	Qu XL, Chai YX, Wang YJ, Han Y, Zhang Y, Tao L. Preoperative neoadjuvant chemotherapy with recombinant human vascular endostatin and DCF regimen for advanced gastric cancer increases the rate of radical surgical resection. Tumor. 2011 2011-01-01;31(8):765-7.
553	Quan R 2016	Review	Quan R, Huang J, Chen H, et al. Comparison of efficacy in adjuvant chemotherapy regimens in patients with radically resected gastric cancer: A propensitymatched analysis. ONCOTARGET. 2016 2016-01-01;7(46):76316-26.
554	Quintero-Aldana G 2015	Single-arm trial	Quintero-Aldana G, Jorge M, Grande C, et al. Phase II study of first-line biweekly docetaxel and cisplatin combination chemotherapy in advanced gastric cancer. CANCER CHEMOTH PHARM. 2015 2015-01-01;76(4):731-7.
555	Rajdev L 2010	Review	Rajdev L. Treatment options for surgically resectable gastric cancer. CURR TREAT OPTION ON. 2010 2010-01-01;11(1-2):14-23.
556	Recchia F 2007	Single-arm trial	Recchia F, Saggio G, Candeloro G, et al. Chemoimmunotherapy in the treatment of metastatic gastric cancer. Anticancer Drugs. 2007 Jun;18(5):597-604.
557	Reed VK 2008	Interventions inconformity	Reed VK, Krishnan S, Mansfield PF, et al. Incidence, Natural History, and Patterns of Locoregional Recurrence in Gastric Cancer Patients Treated With Preoperative Chemoradiotherapy. INT J RADIAT ONCOL. 2008 2008-01-01;71(3):741-7.
558	Reim D 2016	Review	Reim D, Friess H, Novotny A. Outcome of secondary gastrectomy for stage IV gastroesophageal adenocarcinoma after induction-chemotherapy. Translational Gastroenterology and Hepatology. 2016 2016-01-01;5(1):8-15.
559	Richards D 2008	Single-arm trial	Richards D(1), McCollum D, Wilfong L,et al.Phase II trial of docetaxel and oxaliplatin in patients with advanced gastric cancer and/or adenocarcinoma of the gastroesophageal junction.Ann Oncol. 2008 Jan;19(1):104-8. Epub 2007 Sep 25.
560	Richards D 2013	Repeated article	Richards D, Kocs DM, Spira AI,et al. Results of docetaxel plus oxaliplatin (DOCOX) ± cetuximab in patients with metastatic gastric and/or gastroesophageal junction adenocarcinoma: results of a randomised Phase 2 study. Eur J Cancer. 2013 Sep;49(13):2823-31. doi: 10.1016/j.ejca.2013.04.022.

561	Richards D 2013	Repeated article	Richards D, Kocs DM, Spira AI, et al. Results of docetaxel plus oxaliplatin (DOCOX) ± cetuximab in patients with metastatic gastric and/or gastroesophageal junction adenocarcinoma: Results of a randomised Phase 2 study. EUR J CANCER. 2013 2013-01-01;49(13):2823-31.
562	Rino Y 2004	Single-arm trial	Rino Y, Imada T, Takanashi Y. Phase I study of concurrent therapy using TS1 and docetaxel for gastric cancer. J Clin Oncol. 2004 Jul 15;22(14_suppl):4152.
563	Rivera F 2015	Single-arm trial	Rivera F, Massutí B, Salcedo M,et al. Phase II trial of miniDOX (reduced dose docetaxel-oxaliplatin-capecitabine) in "suboptimal" patients with advanced gastric cancer (AGC). TTD 08-02. Cancer Chemother Pharmacol. 2015 Feb;75(2):319-24. doi:10.1007/s00280-014-2641-3.
564	Robb WB 2012	Single-arm trial	Robb WB, Mariette C. Predicting the response to chemotherapy in gastric adenocarcinoma: Who benefits from neoadjuvant chemotherapy? |.*196*196, 2012:241-68.
565	Roberto M 2016	Review	Roberto M, Romiti A, Onesti CE, Zullo A, Falcone R, Marchetti P. Evolving treatments for advanced gastric cancer: Appraisal of the survival trend. EXPERT REV ANTICANC. 2016 2016-01-01;16(7):717-29.
566	Rostom Y 2013	Single-arm trial	Rostom Y, Zaghloul H, Khedr G, et al. Docetaxel-based preoperative chemoradiation in localized gastric cancer: impact of pathological complete response on patient outcome. J Gastrointest Cancer. 2013 Jun;44(2):162-9. doi: 10.1007/s12029-012-9449-3.
567	Rostom Y 2013	Single-arm trial	Rostom Y, Zaghloul H, Khedr G, El-Shazly W, Abd-Allah D. Docetaxel-based preoperative chemoradiation in localized gastric cancer: Impact of pathological complete response on patient outcome. Journal of Gastrointestinal Cancer. 2013 2013-01-01;44(2):162-9.
568	Roth AD 2000	Single-arm trial	Roth AD, Maibach R, Martinelli G,et al.Docetaxel (Taxotere)-cisplatin (TC): an effective drug combination in gastric carcinoma. Swiss Group for Clinical Cancer Research (SAKK), and the European Institute of Oncology (EIO).Ann Oncol. 2000 Mar;11(3):301-6.
569	Roth AD 2002	Single-arm trial	Roth AD. European experience of docetaxel and cisplatin in advanced gastric cancer. Gastric Cancer. 2002;5 Suppl 1:27-9.
570	Roth AD 2002	Repeated article	Roth AD. European experience of docetaxel and cisplatin in advanced gastric cancer. GASTRIC CANCER. 2002 2002-01-01;5(SUPPL. 1):27-9.

571	Roth AD 2003	Repeated article	Roth AD, Ajani J.Docetaxel-based chemotherapy in the treatment of gastric cancer.Ann Oncol. 2003;14 Suppl 2:ii41-4.
572	Roth AD 2003	Repeated article	Roth AD, Ajani J. Docetaxel-based chemotherapy in the treatment of gastric cancer. ANN ONCOL. 2003 2003-01-01;14(SUPPL. 2):i41-4.
573	Roth AD 2004	Single-arm trial	Roth AD, Maibach R, Fazio N,et al.5-Fluorouracil as protracted continuous intravenous infusion can be added to full-dose docetaxel (Taxotere)-cisplatin in advanced gastric carcinoma: a phase I-II trial.Ann Oncol. 2004 May;15(5):759-64.
574	Roth AD 2004	Single-arm trial	Roth AD, Maibach R, Fazio N, et al. 5-Fluorouracil as protracted continuous intravenous infusion can be added to full-dose docetaxel (Taxotere®)-cisplatin in advanced gastric carcinoma: A phase I-II trial. ANN ONCOL. 2004 2004-01-01;15(5):759-64.
575	Roth AD 2007	Repeated article	Roth AD, Fazio N, Stupp R,et al. Docetaxel, cisplatin, and fluorouracil; docetaxel and cisplatin; and epirubicin, cisplatin, and fluorouracil as systemic treatment for advanced gastric carcinoma: a randomized phase II trial of the Swiss Group for Clinical Cancer Research. J Clin Oncol. 2007 Aug 1;25(22):3217-23. doi: 10.1200/JCO.2006.08.0135.
576	Rougier P 1995	Single-arm trial	Rougier P. Docetaxel delivers new management opportunities for gastrointestinal carcinomas. ANTI-CANCER DRUG. 1995 1995-01-01;6(SUPPL. 4):25-9.
577	Rougier P 2008	Single-arm trial	Rougier P. Feasibility of sequential therapy with FOLFIRI followed by docetaxel/cisplatin in patients with radically resected gastric adenocarcinoma: A randomized Phase III trial. Advances in Gastrointestinal Cancers. 2008 2008-01-01;6(1):15-6.
578	Roy A 2012	Population inconformity	Roy A, Cunningham D, Hawkins R,et al. Docetaxel combined with irinotecan or 5-fluorouracil in patients with advanced oesophago-gastric cancer: a randomised phase II study.Br J Cancer. 2012 Jul 24;107(3):435-41. doi: 10.1038/bjc.2012.286. Epub 2012 Jul 5.
579	Roy AC 2013	Repeated article	Roy AC, Park SR, Cunningham D, et al.A randomized phase II study of PEP02 (MM-398), irinotecan or docetaxel as a second-line therapy in patients with locally advanced or metastatic gastric or gastro-oesophageal junction adenocarcinoma.Ann Oncol. 2013 Jun;24(6):1567-73. doi: 10.1093/annonc/mdt002. Epub 2013 Feb 13.
580	Rulli E 2010	Single-arm trial	Rulli E, Bochicchio A, Fazio N, et al. Comparison of a sequential treatment versus a 5-FU/LV regimen as adjuvant therapy for resected gastric cancer Tolerability and feasibility analysis of itaca-s (intergroup trial of adjuvant chemotherapy in adenocarcinoma of the stomach) trial. ANN ONCOL. 2010 2010-01-01;21:i254.
581	Ryoo H 2011	Review	Ryoo H, Bae SH, Hyun MS, Kim MK, Lee KH. Effects of the proteasome inhibitor bortezomib alone and in combination with chemotherapeutic agents in gastric cancer cell lines. EUR J CANCER. 2011 2011-01-01;47:S444.

582	Ryu M 2011	Single-arm trial	Ryu M, Choi Y, Kim B, et al. A single-arm, phase II feasibility study of neoadjuvant docetaxel, oxaliplatin, and S-1 (DOS) chemotherapy in potentially resectable gastric or gastroesophageal junction adenocarcinoma. J CLIN ONCOL. 2011 2011-01-01;29(4).
583	Sadighi S 2006	Population inconformity	Sadighi S, Mohagheghi MA, Montazeri A, et al. Quality of life in patients with advanced gastric cancer: a randomized trial comparing docetaxel, cisplatin, 5-FU (TCF) with epirubicin, cisplatin, 5-FU (ECF). BMC Cancer. 2006 Dec 5;6:274.DOI: 10.1186/1471-2407-6-274
584	Sadighi S 2006	Repeated article	Sadighi S, Mohagheghi MA, Montazeri A, Sadighi Z. Quality of life in patients with advanced gastric cancer: A randomized trial comparing docetaxel, cisplatin, 5-FU (TCF) with epirubicin, cisplatin, 5-FU (ECF). BMC CANCER. 2006 2006-01-01;6.
585	Safran H 1997	Single-arm trial	Safran H, King TP, Choy H, et al. Paclitaxel and concurrent radiation for locally advanced pancreatic and gastric cancer: a phase I study. J Clin Oncol. 1997 Mar;15(3):901-7.
586	Saif MW 2009	Single-arm trial	Saif MW, Syrigos KN, Katirtzoglou NA. S-1: A promising new oral fluoropyrimidine derivative. EXPERT OPIN INV DRUG. 2009 2009-01-01;18(3):335-48.
587	Saitoh S 2002	Single-arm trial	Saitoh S, Sakata Y. Docetaxel and cisplatin in patients with advanced gastric cancer: results of Japanese phase I/II study. Gastric Cancer. 2002;5 Suppl 1:23-6.
588	Sakamoto J 2003	Study design inconformity	Sakamoto J, Morita S, Yumiba T,et al. A phase II clinical trial to evaluate the effect of paclitaxel in patients with ascites caused by advanced or recurrent gastric carcinoma: a new concept of clinical benefit response for non-measurable type of gastric cancer.Jpn J Clin Oncol. 2003 May;33(5):238-40.
589	Sakamoto J 2003	Single-arm trial	Sakamoto J. Neoadjuvant chemotherapy: A standard treatment for locally advanced gastric cancer in the near future? GASTRIC CANCER. 2003 2003-01-01;6(3):131-3.
590	Sakamoto J 2009	Review	Sakamoto J, Matsui T, Kodera Y. Paclitaxel chemotherapy for the treatment of gastric cancer. Gastric Cancer. 2009;12(2):69-78. doi: 10.1007/s10120-009-0505-z.
591	Sakamoto J 2009	Study design inconformity	Sakamoto J, Matsui T, Kodera Y. Paclitaxel chemotherapy for the treatment of gastric cancer. GASTRIC CANCER. 2009 2009-01-01;12(2):69-78.

592	Sasaki K 2007	Single-arm trial	Sasaki K, Natsugoe S, Aridome K, Ishigami S, Hokita S, Aikou T. The successfully curative treatment of advanced gastric adenocarcinoma with multiple liver metastases and paraaortic lymph node metastases by salvage operation following the biweekly paclitaxel and S-1 combination chemotherapy: A case report. HEPATO-GASTROENTEROL. 2007 2007-01-01;54(80):2421-4.
593	Sasaki K 2013	Review	Sasaki K, Fujiwara Y, Kishi K, et al. Repeated occlusion of the intraperitoneal access port for intraperitoneal chemotherapy in a patient with gastric cancer with peritoneal dissemination. Gan To Kagaku Ryoho. 2013 Nov;40(12):2319-21.
594	Sasako M 2014	Review	Sasako M. Ramucirumab: Second-line therapy for gastric cancer. The Lancet Oncology. 2014 2014-01-01;15(11):1182-4.
595	Sastre J 2014	Single-arm trial	Sastre J, García-Saenz JA, Díaz-Rubio E. Chemotherapy for gastric cancer. WORLD J GASTROENTERO. 2006 2006-01-01;12(2):204-13.
596	Sato A 2008	Single-arm trial	Sato A, Koizumi W, Akiya T, et al. Second-line therapy with biweekly paclitaxel after failure of fluoropyrimidine based treatment in patients with advanced or recurrent gastric cancer: Results of a multicenter phase II trial. ANN ONCOL. 2008 2008-01-01;19(S8):i175.
597	Sato Y 2013	Single-arm trial	Sato Y, Sagawa T, Oosuga T, Nakamura T, Fujikawa K, Takahashi Y. Efficacy of gastrectomy with curative intent for patients with metastatic gastric cancer after downstaging by current combination chemotherapy. GASTROENTEROLOGY. 2013 2013-01-01;144(5):S520.
598	Satoh T 2014	Repeated article	Satoh T, Xu RH, Chung HC, et al. Lapatinib plus paclitaxel versus paclitaxel alone in the second-line treatment of HER2-amplified advanced gastric cancer in Asian populations: TyTAN--a randomized, phase III study. J Clin Oncol. 2014 Jul 1;32(19):2039-49. doi: 10.1200/JCO.2013.53.6136.
599	Satoh T 2016	Single-arm trial	Satoh T. Ramucirumab for gastric Cancer. ANN ONCOL. 2016 2016-01-01;27:i12.
600	Scartozi M 2007	Single-arm trial	Scartozi M, Galizia E, Verdecchia L, et al. Chemotherapy for advanced gastric cancer: Across the years for a standard of care. EXPERT OPIN PHARMACO. 2007 2007-01-01;8(6):797-808.
601	Schüll B 2003	Single-arm trial	Schüll B, Kornek GV, Schmid K,et al. Effective combination chemotherapy with bimonthly docetaxel and cisplatin with or without hematopoietic growth factor support in patients with advanced gastroesophageal cancer. Oncology. 2003;65(3):211-7. doi: 74473.

602	Schultheis B 2010	Single-arm trial	Schultheis B, Riebeling J, Allali M, et al. Neoadjuvant treatment of adenocarcinomas of the gastroesophageal junction and stomach - a feasibility trial combining cisplatin and docetaxel with either 5-fluorouracil or capecitabine. Int J Clin Pharmacol Ther. 2010 Jul;48(7):451-2. PMID: 20557841
603	Schwartz GK 2009	Repeated article	Schwartz GK, Winter K, Minsky BD, et al.Randomized phase II trial evaluating two paclitaxel and cisplatin-containing chemoradiation regimens as adjuvant therapy in resected gastric cancer (RTOG-0114). J Clin Oncol. 2009 Apr 20;27(12):1956-62. doi: 10.1200/JCO.2008.20.3745. Epub 2009 Mar 9.
604	Schwartz GK 2009	Repeated article	Schwartz GK, Winter K, Minsky BD, et al. Randomized phase II trial evaluating two paclitaxel and cisplatin-containing chemoradiation regimens as adjuvant therapy in resected gastric cancer (RTOG-0114). J CLIN ONCOL. 2009 2009-01-01;27(12):1956-62.
605	Se HP 2006	Single-arm trial	Se HP, Woon KL, Chung M, et al. Paclitaxel versus docetaxel for advanced gastric cancer: A randomized phase II trial in combination with infusional 5-fluorouracil. ANTI-CANCER DRUG. 2006 2006-01-01;17(2):225-9.
606	Segawa Y 2009	Population inconformity	Segawa Y, Hotta K, Takigawa N, et al. A randomized phase II study of a combination of docetaxel and S-1 versus docetaxel monotherapy in pts with NSCLC previously treated with platinum-based chemotherapy: Results of Okayama Lung Cancer Study Group (OLCSG) trial 0503. J CLIN ONCOL. 2009 2009-01-01;27(15):8058.
607	Sehdev A 2013	Review	Sehdev A, Catenacci DV. Perioperative therapy for locally advanced gastroesophageal cancer: Current controversies and consensus of care. Journal of Hematology and Oncology. 2013 2013-01-01;6(1).
608	Selcukbiricik F 2013	Review	Selcukbiricik F, Buyukunal E, Tural D, Ozguroglu M, Demirelli F, Serdengecti S. Clinicopathological features and outcomes of patients with gastric cancer: A single-center experience. WORLD J GASTROENTERO. 2013 2013-01-01;19(14):2154-61.
609	Selcukbiricik F 2016	Review	Selcukbiricik F, Sag AA, Kanitez M, Bilici A, Mandel NM. Neoadjuvant systemic therapy for patients with gastric cancer: Current concepts and outcomes. Journal of Oncological Science. 2016 2016-01-01;1(1):25-30.
610	Seo HY 2009	Single-arm trial	Seo HY, Kim DS, Choi YS, et al. Treatment outcomes of oxaliplatin, 5-FU, and leucovorin as salvage therapy for patients with advanced or metastatic gastric cancer: A retrospective analysis. CANCER CHEMOTH PHARM. 2009 2009-01-01;63(3):433-9.
611	Shah MA 2010	Repeated article	Shah MA, Shibata S, Stoller RG, et al. Random assignment multicenter phase II study of modified docetaxel, cisplatin, fluorouracil (mDCF) versus DCF with growth factor support (GCSF) in metastatic gastroesophageal adenocarcinoma (GE). J CLIN ONCOL. 2010 2010-01-01;28(15).

612	Sharma A 2006	Single-arm trial	Sharma A, Raina V, Lokeshwar N,et al.Phase II study of cisplatin, etoposide and paclitaxel in locally advanced or metastatic adenocarcinoma of gastric/gastroesophageal junction.Indian J Cancer. 2006 Jan-Mar;43(1):16-9.
613	Shen L 2012	Single-arm trial	Shen L, Xu R, Wang J, et al. A randomized, controlled phase III trial of docetaxel, cisplatin and fluorouracil (DCF) versus cisplatin plus fluorouracil (CF) as first-line therapy in chinese advanced gastric cancer. ANN ONCOL. 2012 2012-01-01;23:x231.
614	Shen L 2012	Single-arm trial	Shen L, Zhang X, Lu Z, et al. Preliminary safety analysis from PAC-C study of capecitabine+paclitaxel→capecitabine maintenance (PX-X) vs capecitabine+cisplatin (XP) as first-line chemotherapy for recurrent/metastatic gastric cancer. ANN ONCOL. 2012 2012-01-01;23:v43-4.
615	Shinohara H 2006	Single-arm trial	Shinohara H, Okamoto S, Nishitai R, et al. A phase I study of intraperitoneal plus intravenous paclitaxel against gastric cancer with peritoneal dissemination (HGCG 0301). Gan To Kagaku Ryoho. 2006 Dec;33(13):2027-31.
616	Shitara K 2011	Study design inconformity	Shitara K, Oze I, Mizota A, et al.Randomized phase II study comparing dose escalated weekly paclitaxel vs. standard dose weekly paclitaxel for patients with previously treated advanced gastric cancer.Jpn J Clin Oncol. 2011 Feb;41(2):287-90. doi: 10.1093/jjco/hyq206. Epub 2010 Nov 9.
617	Shitara K 2011	Single-arm trial	Shitara K, Oze I, Mizota A, et al. Randomized phase II study comparing dose escalated weekly paclitaxel vs. standard dose weekly paclitaxel for patients with previously treated advanced gastric cancer. JPN J CLIN ONCOL. 2011 2011-01-01;41(2):287-90.
618	Shitara K 2013	Single-arm trial	Shitara K, Yuki S, Takahari D, et al. Randomized phase II study comparing dose-escalated weekly paclitaxel versus standard dose weekly paclitaxel for patients with previously treated advanced gastric cancer. J CLIN ONCOL. 2013 2013-01-01;31(15).
619	Shitara K 2014	Repeated article	Shitara K, Yuki S, Tahahari D,et al.Randomised phase II study comparing dose-escalated weekly paclitaxel vs standard-dose weekly paclitaxel for patients with previously treated advanced gastric cancer. Br J Cancer. 2014 Jan 21;110(2):271-7. doi: 10.1038/bjc.2013.726. Epub 2013 Nov 26.
620	Shitara K 2014	Study design inconformity	Shitara K, Ohtsu A. Ramucirumab for gastric cancer. Expert Review of Gastroenterology and Hepatology. 2014 2014-01-01;9(2):133-9.
621	Shitara K 2014	Repeated article	Shitara K, Yuki S, Tahahari D, et al. Randomised phase II study comparing dose-escalated weekly paclitaxel vs standard-dose weekly paclitaxel for patients with previously treated advanced gastric cancer. BRIT J CANCER. 2014 2014-01-01;110(2):271-7.

622	Shitara K 2016	Review	Shitara K, Muro K, Shimada Y, et al. Subgroup analyses of the safety and efficacy of ramucirumab in Japanese and Western patients in RAINBOW: a randomized clinical trial in second-line treatment of gastric cancer. GASTRIC CANCER. 2016 2016-01-01;19(3):927-38.
623	Shitara K 2017	Single-arm trial	Shitara K, Takashima A, Fujitani K, et al. Nab-paclitaxel versus solvent-based paclitaxel in patients with previously treated advanced gastric cancer (ABSOLUTE): an open-label, randomised, non-inferiority, phase 3 trial. The Lancet Gastroenterology and Hepatology. 2017 2017-01-01;2(4):277-87.
624	Sirohi B 2014	Single-arm trial	Sirohi B, Dawood S, Ostwal V, et al. Treatment of patients with advanced gastric cancer-single center experience from tata memorial center (TMC). ANN ONCOL. 2014 2014-01-01;25:i32.
625	Skoropad VY 2015	Repeated article	Vopr Onkol. 2015;61(3):339-45.[CURRENT STATE OF ADJUVANT TREATMENT FOR GASTRIC CANCER AFTER RADICAL SURGERY WITH EXTENDED LYMPH NODE DISSECTION].[Article in Russian]Skoropad VY.
626	Skoropad VY 2015	Repeated article	Vopr Onkol. 2015;61(3):339-45.[CURRENT STATE OF ADJUVANT TREATMENT FOR GASTRIC CANCER AFTER RADICAL SURGERY WITH EXTENDED LYMPH NODE DISSECTION].[Article in Russian]Skoropad VY.
627	Skoropad VY 2015	Single-arm trial	Skoropad VY. The current status of adjuvant treatment for gastric cancer after radical surgery with extended lymph node dissection. Voprosy Onkologii. 2015 2015-01-01;61(3):339-45.
628	Smith MB 2012	Population inconformity	Smith MB, Reardon J, Olson EM. Pertuzumab for the treatment of patients with previously untreated HER2-positive metastatic breast cancer. DRUG TODAY. 2012 2012-01-01;48(11):713-22.
629	Smyth EC 2010	Single-arm trial	Smyth EC, Schöder H, Coit DG, et al. Perioperative chemotherapy plus bevacizumab with early salvage therapy based on PET response in patients with locally advanced resectable gastric/GEJ cancer. J CLIN ONCOL. 2010 2010-01-01;28(15).
630	Smyth EC 2014	Review	Smyth EC, Tarazona N, Chau I. Ramucirumab: targeting angiogenesis in the treatment of gastric cancer. Immunotherapy. 2014;6(11):1177-86. doi: 10.2217/imt.14.85.
631	Smyth EC 2014	Single-arm trial	Smyth EC, Tarazona N, Chau I. Ramucirumab: Targeting angiogenesis in the treatment of gastric cancer. IMMUNOTHERAPY-UK. 2014 2014-01-01;6(11):1177-86.
632	Smyth EC 2016	Review	Smyth EC, Verheij M, Allum W, et al. Gastric cancer: ESMO clinical practice guidelines for diagnosis, treatment and follow-up. ANN ONCOL. 2016 2016-01-01;27:v38-49.
633	Smyth EC 2017	Single-arm trial	Smyth EC. Regorafenib in gastric cancer. Translational Gastroenterology and Hepatology. 2017 2017-01-01;2017(MAR).

634	So J 2012	Single-arm trial	So J. Clinical trials for treatment of advanced gastric cancer. Annals of the Academy of Medicine Singapore. 2012 2012-01-01;41(9):S11.
635	Sohma I 2011	Single-arm trial	Sohma I, Fujiwara Y, Sugita Y, et al. Parthenolide, an NF-κB inhibitor, suppresses tumor growth and enhances response to chemotherapy in gastric cancer. Cancer Genomics and Proteomics. 2011 2011-01-01;8(1):39-48.
636	Song H 2010	Study design inconformity	Song H, He R, Wang K, et al. Anti-HIF-1alpha antibody-conjugated pluronic triblock copolymers encapsulated with Paclitaxel for tumor targeting therapy. Biomaterials. 2010 Mar;31(8):2302-12. doi: 10.1016/j.biomaterials.2009.11.067.
637	Squadroni M 2015	Single-arm trial	Squadroni M, Sauta MG, Bonomi M, et al. Second line chemotherapy for gastric cancer: A single center experience. ANN ONCOL. 2015 2015-01-01;26.
638	Starodub AN 2015	Single-arm trial	Starodub AN, Ocean AJ, Shah MA, et al. First-in-human trial of a novel anti-trop-2 Antibody-SN-38 conjugate, sacituzumab govitecan, for the treatment of diverse metastatic solid tumors. CLIN CANCER RES. 2015 2015-01-01;21(17):3870-8.
639	Stathopoulos GP 2011	Population inconformity	Stathopoulos GP, Zoublios Ch, Stathopoulos J. Metastatic lung disease treated with pemetrexed-docetaxel combination chemotherapy. J BUON. 2011 Jan-Mar;16(1):166-9.
640	Stein A 2014	Single-arm trial	Stein A, Arnold D, Thuss-Patience PC, et al. Docetaxel, oxaliplatin and capecitabine (TEX regimen) in patients with metastatic gastric or gastro-esophageal cancer: results of a multicenter phase I/II study. Acta Oncol. 2014 Mar;53(3):392-8. doi: 10.3109/0284186X.2013.833346.
641	Strasser F 2008	Repeated article	Strasser F, Demmer R, Böhme C, et al. Prevention of docetaxel- or paclitaxel-associated taste alterations in cancer patients with oral glutamine: A randomized, placebo-controlled, double-blind study. ONCOLOGIST. 2008 2008-01-01;13(3):337-46.
642	Sudo K 2015	Single-arm trial	Sudo K, Yamada Y. Advancing pharmacological treatment options for advanced gastric cancer. EXPERT OPIN PHARMACO. 2015 2015-01-01;16(15):2293-305.
643	Sugimoto N 2014	Repeated article	Sugimoto N, Fujitani K, Imamura H, et al. Randomized phase II trial of S-1 plus irinotecan versus S-1 plus paclitaxel as first-line treatment for advanced gastric cancer (OGSG0402). Anticancer Res. 2014 Feb;34(2):851-7.
644	Sugimoto N 2014	Single-arm trial	Sugimoto N, Fujitani K, Imamura H, et al. Randomized phase II trial of S-1 plus irinotecan versus S-1 plus paclitaxel as first-line treatment for advanced gastric cancer (OGSG0402). ANTICANCER RES. 2014 2014-01-01;34(2):851-8.
645	Sugiyama K 2017	Single-arm trial	Sugiyama K, Narita Y, Kadowaki S, Ura T, Tajika M, Muro K. Platinum-based doublet chemotherapy for advanced gastric cancer with disseminated intravascular coagulation. ANTICANCER RES. 2017 2017-01-01;37(1):309-14.

646	Sulkes A 1994	Single-arm trial	Sulkes A, Smyth J, Sessa C,et al.Docetaxel (Taxotere) in advanced gastric cancer: results of a phase II clinical trial. EORTC Early Clinical Trials Group.Br J Cancer. 1994 Aug;70(2):380-3.
647	Sun DS 2015	Repeated article	Sun DS, Jeon EK, Won HS, et al. Outcomes in elderly patients treated with a single-agent or combination regimen as first-line chemotherapy for recurrent or metastatic gastric cancer. GASTRIC CANCER. 2015 2015-01-01;18(3):644-52.
648	Sun GP 2013	Single-arm trial	Sun GP, Sun Y, Xu RH, et al. The Chinese subgroup from a randomized phase III study of lapatinib in combination with weekly paclitaxel versus weekly paclitaxel alone as second-line treatment of HER2-amplified advanced gastric cancer (AGC) in Asian countries. J CLIN ONCOL. 2013 2013-01-01;31(15).
649	Sun Q 2009	Single-arm trial	Sun Q, Liu C, Zhong H, et al.Multi-center phase II trial of weekly paclitaxel plus cisplatin combination chemotherapy in patients with advanced gastric and gastro-esophageal cancer.Jpn J Clin Oncol. 2009 Apr;39(4):237-43. doi: 10.1093/jjco/hyp008. Epub 2009 Mar 4.
650	Sun X 2011	Single-arm trial	Sun X, Lin J, Ju AH. Treatment of Borrmann type IV gastric cancer with a neoadjuvant chemotherapy combination of docetaxel, cisplatin and 5-fluorouracil/leucovorin. J INT MED RES. 2011 2011-01-01;39(6):2096-102.
651	Sun XC 2011	Repeated article	Sun XC, Lin J, Ju AH. Treatment of Borrmann type IV gastric cancer with a neoadjuvant chemotherapy combination of docetaxel, cisplatin and 5-fluorouracil/leucovorin. J Int Med Res. 2011;39(6):2096-102.
652	Sym S 2009	Repeated article	Sym S, Park S, Park J, et al. A randomized phase II trial of weekly docetaxel plus either cisplatin or oxaliplatin in patients with previously untreated advanced gastric cancer: Preliminary results. European Journal of Cancer, Supplement. 2009 2009-01-01;7(2-3):383.
653	Taguchi T 1998	Single-arm trial	Taguchi T, Sakata Y, Kanamaru R, et al. Late phase II clinical study of RP56976 (docetaxel) in patients with advanced/recurrent gastric cancer: a Japanese Cooperative Study Group trial (group A). Gan To Kagaku Ryoho. 1998 Oct;25(12):1915-24.
654	Tahara M 2000	Single-arm trial	Tahara M, Ohtsu A. Latest progress on chemotherapy for advanced gastric cancer. Gan To Kagaku Ryoho. 2000 Nov;27(13):2048-58.
655	Takagi K 2011	Review	Takagi K, Chin K, Oba M, et al. Efficacy of paclitaxel for S-1-refractory gastric cancer (Japan). JPN J CLIN ONCOL. 2011 2011-01-01;41(3):i15.
656	Takahari D 2013	Single-arm trial	Takahari D, Shitara K, Yuki S, et al. Randomized phase II study comparing dose-escalated weekly paclitaxel (wPTX) versus standard-dose wPTX for patients with previously treated advanced gastric cancer (AGC). J CLIN ONCOL. 2013 2013-01-01;31(4).

657	Takahari D 2017	Review	Takahari D. Second-line chemotherapy for patients with advanced gastric cancer. GASTRIC CANCER. 2017 2017-01-01;20(3):395-406.
658	Takahashi I 2006	Single-arm trial	Takahashi I, Oki E, Egashira A, Morita M, Kakeji Y, Maehara Y. Combination chemotherapy of S-1 plus biweekly docetaxel for advanced and recurrent gastric cancer. Gan to kagaku ryoho. Cancer & chemotherapy. 2006 2006-01-01;33 Suppl 1:87-90.
659	Takahashi M 2010	Single-arm trial	Takahashi M, Koeda K, Fujiwara H,et al. Five cases of advanced gastroesophageal junction adenocarcinoma successfully treated with chemoradiotherapy followed by curative resection. Gan To Kagaku Ryoho. 2010 Nov;37(11):2169-71.
660	Takahashi S 2013	Study design inconformity	Takahashi S, Hirayama M, Kuroiwa G, et al. Diagnostic validity of CT gastrography versus gastroscopy for primary lesions in gastric cancer: evaluating the response to chemotherapy, a retrospective analysis. Gastric Cancer. 2013 Oct;16(4):543-8. doi: 10.1007/s10120-012-0217-7.
661	Takahashi T 2012	Single-arm trial	Takahashi T, Saikawa Y, Fukuda K,et al. Phase I study of combination chemotherapy consisting of paclitaxel, cisplatin, and S-1 in patients with unresectable gastric cancer (KOGC-02). Anticancer Res. 2012 Dec;32(12):5401-6.
662	Takashima A 2014	Repeated article	Takashima A, Boku N, Kato K, et al. Survival prolongation after treatment failure of first-line chemotherapy in patients with advanced gastric cancer: Combined analysis of the Japan Clinical Oncology Group Trials JCOG9205 and JCOG9912. GASTRIC CANCER. 2014 2014-01-01;17(3):522-8.
663	Takeuchi H 2010	Study design inconformity	Takeuchi H, Ueda M, Oyama T, et al. Molecular diagnosis and translymphatic chemotherapy targeting sentinel lymph nodes of patients with early gastrointestinal cancers. Digestion. 2010;82(3):187-91. doi: 10.1159/000309464.
664	Takiguchi N 2015	Repeated article	Takiguchi N, Kodera Y, Takahashi N, et al. Feasibility of weekly intraperitoneal versus intravenous paclitaxel delivered from the day of radical surgery for gastric cancer: A preliminary safety analysis of INPACT study, a randomized controlled trial. EUR J CANCER. 2015 2015-01-01;51:S408.
665	Takiuchi H 2005	Interventions inconformity	Takiuchi H, Goto M, Kawabe S,et al. Second-line chemotherapy in gastric cancer. Gan To Kagaku Ryoho. 2005 Jan;32(1):19-23.
666	Takiuchi H 2010	Review	Takiuchi H, Fukuda H, Boku N, et al. Randomized phase II study of best-available 5-fluorouracil (5-FU) versus weekly paclitaxel in gastric cancer (GC) with peritoneal metastasis (PM) refractory to 5-FU-containing regimens (JCOG0407). J CLIN ONCOL. 2010 2010-01-01;28(15).
667	Takiuchi H 2011	Review	Takiuchi H. Second-line chemotherapy for gastric cancer: A new issue lies ahead in global trials. GASTRIC CANCER. 2011 2011-01-01;14(3):206-11.

668	Tamura S 2009	Single-arm trial	Tamura S, Miki H, Okada K, et al. Outcome of therapy for type 4 gastric cancer with peritoneal metastasis--diagnosis by laparoscopy and effect of chemotherapy. Gan To Kagaku Ryoho. 2009 Nov;36(12):2000-2.
669	Tanabe K 2010	Single-arm trial	Tanabe K, Suzuki T, Tokumoto N, Yamamoto H, Yoshida K, Ohdan H. Combination therapy with docetaxel and S-1 as a first-line treatment in patients with advanced or recurrent gastric cancer: A retrospective analysis. WORLD J SURG ONCOL. 2010 2010-01-01;8.
670	Tanabe K 2013	Repeated article	Tanabe K, Yoshikawa T, Tsuburaya A, et al. Induction of pathologic complete response by long-term neoadjuvant chemotherapy for gastric cancer: Early results of a randomized phase II study-A COMPASS trial. J CLIN ONCOL. 2013 2013-01-01;31(4).
671	Tanabe K 2015	Repeated article	Tanabe K, Fujii M, Nishikawa K, et al. Phase II/III study of second-line chemotherapy comparing irinotecan-alone with S-1 plus irinotecan in advanced gastric cancer refractory to first-line treatment with S-1 (JACCRO GC-05). ANN ONCOL. 2015 2015-01-01;26(9):1916-22.
672	Tanaka R 2005	Study design inconformity	Tanaka R, Takii Y, Shibata Y,et al. In vitro sequence-dependent interaction between nedaplatin and paclitaxel in human cancer cell lines. Cancer Chemother Pharmacol. 2005 Sep;56(3):279-85.
673	Tanaka T 2017	Study design inconformity	Tanaka T, Suda K, Satoh S, et al. Effectiveness of laparoscopic stomach-partitioning gastrojejunostomy for patients with gastric outlet obstruction caused by advanced gastric cancer. SURG ENDOSC. 2017 2017-01-01;31(1):359-67.
674	Tebbutt NC 2010	Population inconformity	Tebbutt NC, Cummins MM, Sourjina T,et al.Randomised, non-comparative phase II study of weekly docetaxel with cisplatin and 5-fluorouracil or with capecitabine in oesophagogastric cancer: the AGITG ATTAX trial.Br J Cancer. 2010 Feb 2;102(3):475-81. doi:10.1038/sj.bjc.6605522. Epub 2010 Jan 12.
675	Tebbutt NC 2010	Single-arm trial	Tebbutt NC, Gebski VJ, Hall M, et al. Randomised, Non-Comparative phase II study of weekly docetaxel, cisplatin and 5-fluorouracil or capecitabine (wTCT/X) given with or without panitumumab in advanced oesophago-gastric cancer: The AGITG ATTAX 3 trial. ASIA-PAC J CLIN ONCO. 2010 2010-01-01;6:180.
676	Tebbutt NC 2013	Population inconformity	Tebbutt NC, Parry MM, Zannino D, et al. Docetaxel plus cetuximab as second-line treatment for docetaxel-refractory oesophagogastric cancer: the AGITG ATTAX2 trial.Br J Cancer. 2013 Mar 5;108(4):771-4. doi: 10.1038/bjc.2013.41.
677	Tebbutt NC 2013	Population inconformity	Tebbutt NC, Parry MM, Zannino D, et al. Docetaxel plus cetuximab as second-line treatment for docetaxel-refractory oesophagogastric cancer: The AGITG ATTAX2 trial. BRIT J CANCER. 2013 2013-01-01;108(4):771-4.
678	Tebbutt NC 2016	Repeated article	Tebbutt NC, Price TJ, Ferraro DA, et al. Panitumumab added to docetaxel, cisplatin and fluoropyrimidine in oesophagogastric cancer: ATTAX3 phase II trial. Br J Cancer. 2016 Mar 1;114(5):505-9. doi: 10.1038/bjc.2015.440.

679	Tebbutt NC 2016	Repeated article	Tebbutt NC, Price TJ, Ferraro DA, et al. Panitumumab added to docetaxel, cisplatin and fluoropyrimidine in oesophagogastric cancer: ATTAX3 phase II trial. Br J Cancer. 2016 Mar 1;114(5):505-9. doi: 10.1038/bjc.2015.440.
680	Tebbutt NC 2016	Population inconformity	Tebbutt NC, Price TJ, Ferraro DA,et al. Panitumumab added to docetaxel, cisplatin and fluoropyrimidine in oesophagogastric cancer: ATTAX3 phase II trial. Br J Cancer. 2016 Mar 1;114(5):505-9. doi: 10.1038/bjc.2015.440.
681	Teker F 2014	Repeated article	Teker F, Yilmaz B, Kemal Y,et al.Efficacy and safety of docetaxel or epirubicin, combined with cisplatin and fluorouracil (DCF and ECF), regimens as first line chemotherapy for advanced gastric cancer: a retrospective analysis from Turkey.Asian Pac J Cancer Prev. 2014;15(16):6727-32.
682	Teker F 2014	Repeated article	Teker F, Yilmaz B, Kemal Y, Kut E, Yucel I. Efficacy and safety of docetaxel or epirubicin, combined with cisplatin and fluorouracil (DCF and ECF), regimens as first line chemotherapy for advanced gastric cancer: a retrospective analysis from Turkey. Asian Pacific journal of cancer prevention : APJCP. 2014 2014-01-01;15(16):6727-32.
683	ter Veer E 2016	Study design inconformity	ter Veer E, Haj MN, van Valkenhoef G, et al. Second- and third-line systemic therapy in patients with advanced esophagogastric cancer: a systematic review of the literature. CANCER METAST REV. 2016 2016-01-01;35(3):439-56.
684	Thuss-Patience PC 2003	Single-arm trial	Thuss-Patience PC, Kretzschmar A, Krenn V,et al. Recurrent gastric adenocarcinoma with unusual metastatic localization and excellent response to docetaxel and 5-FU continuous infusion. Onkologie. 2003 Feb;26(1):63-5.doi: 10.1159/000069866.
685	Thuss-Patience PC 2005	Repeated article	Thuss-Patience PC, Kretzschmar A, Repp M, et al. Docetaxel and continuous-infusion fluorouracil versus epirubicin, cisplatin, and fluorouracil for advanced gastric adenocarcinoma: A randomized phase II study. J CLIN ONCOL. 2005 2005-01-01;23(3):494-501.
686	Thuss-Patience PC 2006	Review	Thuss-Patience PC, Kretzschmar A, Reichardt P. Docetaxel in the treatment of gastric cancer. Future Oncol. 2006 Oct;2(5):603-20. DOI: 10.2217/14796694.2.5.603.
687	Thuss-Patience PC 2006	Single-arm trial	Thuss-Patience PC, Kretzschmar A, Reichardt P. Docetaxel in the treatment of gastric cancer. FUTURE ONCOL. 2006 2006-01-01;2(5):603-20.
688	Thuss-Patience PC 2011	Repeated article	Thuss-Patience PC, Kretzschmar A, Dogan Y, et al.Docetaxel and capecitabine for advanced gastric cancer: investigating dose-dependent efficacy in two patient cohorts.Br J Cancer. 2011 Aug 9;105(4):505-12. doi: 10.1038/bjc.2011.278.
689	Thuss-Patience PC 2011	Repeated article	Thuss-Patience PC, Kretzschmar A, Bichev D, et al. Survival advantage for irinotecan versus best supportive care as second-line chemotherapy in gastric cancer - A randomised phase III study of the Arbeitsgemeinschaft Internistische Onkologie (AIO). EUR J CANCER. 2011 2011-01-01;47(15):2306-14.

690	Thuss-Patience PC 2012	Population inconformity	Thuss-Patience PC, Hofheinz RD, Arnold D,et al.Perioperative chemotherapy with docetaxel, cisplatin and capecitabine (DCX) in gastro-oesophageal adenocarcinoma: a phase II study of the Arbeitsgemeinschaft Internistische Onkologie (AIO){dagger}.Ann Oncol. 2012 Nov;23(11):2827-34. doi: 10.1093/annonc/mds129. Epub 2012 Jun 24.
691	Toh U 2009	Single-arm trial	Toh U, Yamana H, Koufuji K, et al. Phase II study of S-1 in combination with paclitaxel as a first-line treatment for patients with advanced/recurrent gastric cancer. J CLIN ONCOL. 2009 2009-01-01;27(15):e15615.
692	Tomasello G 2010	Single-arm trial	Tomasello G, Chiesa MD, Buti S,et al.Dose-dense chemotherapy in metastatic gastric cancer with a modified docetaxel-cisplatin-5-fluorouracil regimen.Tumori. 2010 Jan-Feb;96(1):48-53.
693	Tomasello G 2010	Study design inconformity	Tomasello G, Chiesa MD, Buti S, et al.Dose-dense chemotherapy in metastatic gastric cancer with a modified docetaxel-cisplatin-5-fluorouracil regimen.Tumori. 2010 Jan-Feb;96(1):48-53.
694	Tomasello G 2010	Single-arm trial	Tomasello G, Chiesa MD, Buti S, et al. Dose-dense chemotherapy in metastatic gastric cancer with a modified docetaxel-cisplatin-5-fluorouracil regimen. TUMORI. 2010 2010-01-01;96(1):48-53.
695	Tomasello G 2012	Single-arm trial	Tomasello G, Liguigli W, Poli R, et al. Sequential chemotherapy with dose-dense docetaxel, cisplatin, folinic acid and 5-fluorouracil (TCF-dd) followed by oxaliplatin, folinic acid, 5-fluorouracil, and irinotecan (COFFI) in locally advanced or metastatic gastric cancer (MGC). J CLIN ONCOL. 2012 2012-01-01;30(15).
696	Tomasello G 2012	Single-arm trial	Tomasello G, Liguigli W, Lazzarelli S, et al. Efficacy and safety of dose-dense chemotherapy with modified TCF regimen (TCF-DD) in elderly patients with metastatic gastric cancer (MGC). ANN ONCOL. 2012 2012-01-01;23:x232.
697	Tomasello G 2013	Single-arm trial	Tomasello G, Liguigli W, Poli R, et al. Efficacy and tolerability of chemotherapy with modified dose-dense TCF regimen (TCF-dd) in locally advanced or metastatic gastric cancer: final results of a phase II trial. GASTRIC CANCER. 2014 2014-01-01;17(4):711-7.
698	Tomasello G 2013	Single-arm trial	Tomasello G, Liguigli W, Poli R, et al. Efficacy and tolerability of chemotherapy with modified dose-dense TCF regimen (TCF-dd) in locally advanced or metastatic gastric cancer: final results of a phase II trial. GASTRIC CANCER. 2013 2013-01-01;17(4):711-7.
699	Tomasello G 2014	Single-arm trial	Tomasello G, Liguigli W, Poli R, et al. Efficacy and tolerability of chemotherapy with modified dose-dense TCF regimen (TCF-dd) in locally advanced or metastatic gastric cancer: final results of a phase II trial. Gastric Cancer. 2014 Oct;17(4):711-7. doi: 10.1007/s10120-013-0317-z.

700	Tomlinson BK 2015	Population inconformity	Tomlinson BK, Thomson JA, Bomalaski JS, et al. Phase i trial of arginine deprivation therapy with ADI-PEG 20 plus docetaxel in patients with advanced malignant solid tumors. CLIN CANCER RES. 2015 2015-01-01;21(11):2480-6.
701	Tsai JY 2003	Review	Tsai JY, Safran H. Status of treatment for advanced gastric carcinoma. CURR ONCOL REP. 2003 2003-01-01;5(3):210-8.
702	Tsai JY 2005	Single-arm trial	Tsai JY, Iannitti D, Berkenblit A, et al. Phase I study of docetaxel, capecitabine, and carboplatin in metastatic esophagogastric cancer. Am J Clin Oncol. 2005 Aug;28(4):329-33.
703	Tsuburaya A 2005	Repeated article	Tsuburaya A, Sakamoto J, Morita S,et al.A randomized phase III trial of post-operative adjuvant oral fluoropyrimidine versus sequential paclitaxel/oral fluoropyrimidine; and UFT versus S1 for T3/T4 gastric carcinoma: the Stomach Cancer Adjuvant Multi-institutional Trial Group (Samit) Trial. Jpn J Clin Oncol. 2005 Nov;35(11):672-5. Epub 2005 Nov 7.
704	Tsuburaya A 2005	Study design inconformity	Tsuburaya A, Sakamoto J, Morita S, et al. A randomized phase III trial of post-operative adjuvant oral fluoropyrimidine versus sequential paclitaxel/oral fluoropyrimidine; and UFT versus S1 for T3/T4 gastric carcinoma: The stomach cancer adjuvant multi-institutional trial group (Samit) trial. JPN J CLIN ONCOL. 2005 2005-01-01;35(11):672-5.
705	Tsuburaya A 2013	Single-arm trial	Tsuburaya A, Nagata N, Cho H, et al. Phase II trial of paclitaxel and cisplatin as neoadjuvant chemotherapy for locally advanced gastric cancer. Cancer Chemother Pharmacol. 2013 May;71(5):1309-14. doi:10.1007/s00280-013-2130-0.
706	Tsuburaya A 2014	Repeated article	Tsuburaya A, Yoshida K, Kobayashi M, et al.Sequential paclitaxel followed by tegafur and uracil (UFT) or S-1 versus UFT or S-1 monotherapy as adjuvant chemotherapy for T4a/b gastric cancer (SAMIT): a phase 3 factorial randomised controlled trial. Lancet Oncol. 2014 Jul;15(8):886-93. doi: 10.1016/S1470-2045(14)70025-7.
707	Tsuburaya A 2014	Interventions inconformity	Tsuburaya A, Yoshida K, Kobayashi M, et al. Sequential paclitaxel followed by tegafur and uracil (UFT) or S-1 versus UFT or S-1 monotherapy as adjuvant chemotherapy for T4a/b gastric cancer (SAMIT): A phase 3 factorial randomised controlled trial. The Lancet Oncology. 2014 2014-01-01;15(8):886-93.
708	Tural D 2012	Single-arm trial	Tural D, Selçukbiricik F, Serdengeçti S, Büyükünal E. A comparison of patient characteristics, prognosis, treatment modalities, and survival according to age group in gastric cancer patients. WORLD J SURG ONCOL. 2012 2012-01-01;10.
709	Ueda S 2012	Single-arm trial	Ueda S, Hironaka S, Yasui H, et al. Randomized phase III study of irinotecan (CPT-11) versus weekly paclitaxel (wPTX) for advanced gastric cancer (AGC) refractory to combination chemotherapy (CT) of fluoropyrimidine plus platinum (FP): WJOG4007 trial. J CLIN ONCOL. 2012 2012-01-01;30(15).
710	Ueda S 2015	Single-arm trial	Ueda S, Satoh T, Gotoh M, Gao L, Doi T. A phase Ib study of safety and pharmacokinetics of ramucirumab in combination with paclitaxel in patients with advanced gastric adenocarcinomas. ONCOLOGIST. 2015 2015-01-01;20(5):493-4.

711	Van Cutsem E 2004	Review	Van Cutsem E.The treatment of advanced gastric cancer: new findings on the activity of the taxanes.Oncologist. 2004;9 Suppl 2:9-15.
712	Van Cutsem E 2004	Review	Van Cutsem E. Docetaxel in gastric cancer. European Journal of Cancer, Supplement. 2004 2004-01-01;2(7):52-8.
713	Van Cutsem E 2004	Review	Van Cutsem E. The treatment of advanced gastric cancer: New findings on the activity of the taxanes. ONCOLOGIST. 2004 2004-01-01;9(SUPPL. 2):9-15.
714	Van Cutsem E 2006	Repeated article	Van Cutsem E, Moiseyenko VM, Tjulandin S, et al. Phase III study of docetaxel and cisplatin plus fluorouracil compared with cisplatin and fluorouracil as first-line therapy for advanced gastric cancer: a report of the V325 Study Group. J Clin Oncol. 2006 Nov 1;24(31):4991-7. doi: 10.1200/JCO.2006.06.8429.
715	Van Cutsem E 2006	Single-arm trial	Van Cutsem E, Moiseyenko VM, Tjulandin S, et al. Phase III study of docetaxel and cisplatin plus fluorouracil compared with cisplatin and fluorouracil as first-line therapy for advanced gastric cancer: A report of the V25 study group. J CLIN ONCOL. 2006 2006-01-01;24(31):4991-7.
716	Van Cutsem E 2009	Repeated article	Van Cutsem E. Current chemotherapy options for advanced disease. European Journal of Cancer, Supplement. 2009 2009-01-01;7(2-3):73-4.
717	Van Cutsem E 2011	Repeated article	Van Cutsem E, Boni C, Tabernero J, et al. Randomized phase II study (GATE study) of docetaxel plus oxaliplatin with or without fluorouracil or capecitabine in metastatic or locally recurrent gastric cancer. J CLIN ONCOL. 2011 2011-01-01;29(15).
718	Van Cutsem E 2015	Repeated article	Van Cutsem E, Boni C, Tabernero J,et al. Docetaxel plus oxaliplatin with or without fluorouracil or capecitabine in metastatic or locally recurrent gastric cancer: a randomized phase II study.Ann Oncol. 2015 Jan;26(1):149-56.DOI: 10.1093/annonc/mdu496
719	Vasile E 2014	Review	Vasile E, Caparello C, Caponi S, et al. Not only chemotherapy in the second-line treatment of metastatic gastric cancer. ANN ONCOL. 2014 2014-01-01;25(2):544-5.
720	Veer ET 2016	Population inconformity	Veer ET, Mohammad NH, Van Valkenhoef G, et al. The Efficacy and Safety of First-line Chemotherapy in Advanced Esophagogastric Cancer: A Network Meta-analysis. Journal of the National Cancer Institute. 2016 2016-01-01;108(10).
721	Verweij J 1994	Single-arm trial	Verweij J. Docetaxel (Taxotere®): A new anti-cancer drug with promising potential? BRIT J CANCER. 1994 1994-01-01;70(2):183-4.
722	Vickers M 2010	Review	Vickers M, Samson B, Colwell B, et al. Eastern Canadian colorectal cancer consensus conference: Setting the limits of resectable disease. CURR ONCOL. 2010 2010-01-01;17(3):70-7.

723	Vogl U 2016	Population inconformity	Vogl U, Vormittag L, Winkler T, et al. Ramucirumab and paclitaxel in platin refractory advanced or metastatic gastric or gastrooesophageal junction adenocarcinoma - A single center experience. ANN ONCOL. 2016 2016-01-01;27:i52.
724	Waddell T 2014	Review	Waddell T, Moorcraft SY, Cunningham D. Potential role of rilotumumab in the treatment of gastric cancer. IMMUNOTHERAPY-UK. 2014 2014-01-01;6(12):1243-53.
725	Wadler S 2002	Repeated article	Wadler S, Brain C, Catalano P, et al. Randomized phase II trial of either fluorouracil, parenteral hydroxyurea, interferon-alpha-2a, and filgrastim or doxorubicin/docetaxel in patients with advanced gastric cancer with quality-of-life assessment: eastern cooperative oncology group study E6296. Cancer J. 2002 May-Jun;8(3):282-6.
726	Wadler S 2002	Single-arm trial	Wadler S, Brain C, Catalano P, Einzig AI, Cella D, Benson IAB. Randomized phase II trial of either fluorouracil, parenteral hydroxyurea, interferon-α-2a, and filgrastim or doxorubicin/docetaxel in patients with advanced gastric cancer with quality-of-life assessment: Eastern Cooperative Oncology Group study E6296. CANCER J. 2002 2002-01-01;8(3):282-6.
727	Wagner AD 2008	Review	Wagner AD, Stahl M. Treatment options for chemotherapy in metastatic gastric cancer. ONKOLOGE. 2008 2008-01-01;14(4):381-8.
728	Wagner AD 2010	Unavailable	Wagner AD, Unverzagt S, Grothe W, et al. Chemotherapy for advanced gastric cancer. Cochrane database of systematic reviews (Online). 2010 2010-01-01;3:D4064.
729	Wahab MA 2011	Single-arm trial	Wahab MA, Ezzelarab L, El BS. Cetuximab plus capecitabine and oxaloplatin for chemonaive patients with advanced gastric cancer. ANN ONCOL. 2011 2011-01-01;22:v50.
730	Wahid M 2016	Review	Wahid M, Mandal RK, Dar SA, et al. Therapeutic potential and critical analysis of trastuzumab and bevacizumab in combination with different chemotherapeutic agents against metastatic breast/colorectal cancer affecting various endpoints. Critical Reviews in Oncology/Hematology. 2016 2016-01-01;104:124-30.
731	Wakahara T 2010	Single-arm trial	Wakahara T, Toyokawa A, Tomono A, et al. S-1-based chemotherapy for recurrent gastric cancer with peritoneal dissemination resulting in long-term survival-report of a case. Japanese Journal of Cancer and Chemotherapy. 2010 2010-01-01;37(7):1361-4.
732	Wang B 2008	Single-arm trial	Wang B, Zhang W, Hong X,et al. Phase I dose-escalating study of 24-h continuous infusion of 5-fluorouracil in combination with weekly docetaxel and cisplatin in patients with advanced gastric cancer. Cancer Chemother Pharmacol. 2009 Jan;63(2):213-8. doi: 10.1007/s00280-008-0728-4.
733	Wang HZ 2007	Interventions inconformity	Wang HZ, Wang HB, Gao H. Clinical observation on treatment of 34 advanced gastric carcinoma patients by chemotherapy of DCF regimen combined with Fuzheng Hewei Decoction. Zhongguo Zhong Xi Yi Jie He Za Zhi. 2007 Oct;27(10):927-9.

734	Wang J 2016	Repeated article	Wang J, Xu R, Li J, et al. Randomized multicenter phase III study of a modified docetaxel and cisplatin plus fluorouracil regimen compared with cisplatin and fluorouracil as first-line therapy for advanced or locally recurrent gastric cancer. GASTRIC CANCER. 2016 2016-01-01;19(1):234-44.
735	Wang L 2017	Study design inconformity	Wang L, Liu Y, Zhou W, Li W. Treatment-related severe and fatal adverse events with molecular targeted agents in the treatment of advanced gastric cancer: A meta-analysis. ONCOTARGETS THER. 2017 2017-01-01;10:2281-7.
736	Wang X 2013	Repeated article	Wang X, Wang ML, Zhou LY, et al. Randomized phase II study comparing paclitaxel with S-1 vs. S-1 as first-line treatment in patients with advanced gastric cancer. Clin Transl Oncol. 2013 Oct;15(10):836-42. doi: 10.1007/s12094-013-1012-6.
737	Watanabe T 2012	Study design inconformity	Watanabe T, Yoshikawa T, Kameda Y, et al. Pathological complete response of locally advanced gastric cancer after four courses of neoadjuvant chemotherapy with paclitaxel plus cisplatin: report of a case. Surg Today (2012) 42:983–987. doi: 10.1007/s00595-012-0155-3.
738	Watanabe T 2012	Study design inconformity	Watanabe T, Yoshikawa T, Kameda Y, et al. Pathological complete response of locally advanced gastric cancer after four courses of neoadjuvant chemotherapy with paclitaxel plus cisplatin: Report of a case. SURG TODAY. 2012 2012-01-01;42(10):983-7.
739	Wei J 2012	Study design inconformity	Wei J, Liu B, Yu L, et al. Overall survival (OS) to first-and second-line chemotherapy associated with mrna expression of multiple myeloma set (mmset) domain, p53-binding protein 1 (53BP1) and breast cancer susceptibility gene 1 (BRCA1) in advanced gastric cancer patients (P). ANN ONCOL. 2012 2012-01-01;23:x229-30.
740	Welz S 2007	Single-arm trial	Welz S, Hehr T, Kollmannsberger C, Bokemeyer C, Belka C, Budach W. Renal toxicity of adjuvant chemoradiotherapy with cisplatin in gastric cancer. Int J Radiat Oncol Biol Phys. 2007 Dec 1;69(5):1429-35.
741	Wesolowski R 2009	Single-arm trial	Wesolowski R, Lee C, Kim R. Is there a role for second-line chemotherapy in advanced gastric cancer? The Lancet Oncology. 2009 2009-01-01;10(9):903-12.
742	Wick MJ 2009	Single-arm trial	Wick MJ, Cooper JD, Kelly SG, et al. Preclinical bioanalytical development to optimize drug treatment regimens using orthotopic models of human gastric cancer. MOL CANCER THER. 2009 2009-01-01;8(12).
743	Wiernik PH 1987	Single-arm trial	Wiernik PH, Schwartz EL, Strauman JJ, et al. Phase I clinical and pharmacokinetic study of taxol.Cancer Res. 1987 May 1;47(9):2486-93.
744	Wilke H 2004	Review	Wilke H, Bouché O, Rougier P, Köhne CH. Irinotecan for the treatment of gastric cancer. European Journal of Cancer, Supplement. 2004 2004-01-01;2(7):48-51.

745	Wilke H 2012	Interventions inconformity	Wilke H, Cunningham D, Ohtsu A, Nuber U, Bruns R, Beate SB. A randomized, multicenter, double-blind, placebo (PBO)-controlled phase III study of paclitaxel (PTX) with or without ramucirumab (IMC-1121B; RAM) in patients (pts) with metastatic gastric adenocarcinoma, refractory to or progressive after first-line therapy with platinum (PLT) and fluoropyrimidine (FP). J CLIN ONCOL. 2012 2012-01-01;30(15).
746	Wilke H 2014	Repeated article	Wilke H, Muro K, Van Cutsem E, et al.Ramucirumab plus paclitaxel versus placebo plus paclitaxel in patients with previously treated advanced gastric or gastro-oesophageal junction adenocarcinoma (RAINBOW): a double-blind, randomised phase 3 trial.Lancet Oncol. 2014 Oct;15(11):1224-35. doi: 10.1016/S1470-2045(14)70420-6.
747	Wilke H 2014	Single-arm trial	Wilke H, Van Cutsem E, Cheul OS, et al. RAINBOW: A global, phase 3, randomized, double-blind study of ramucirumab plus paclitaxel versus placebo plus paclitaxel in the treatment of metastatic gastric adenocarcinoma following disease progression on first-line platinum- and fluoropyrimidine-containing combination therapy: Results of a multiple Cox regression analysis adjusting for prognostic factors. J CLIN ONCOL. 2014 2014-01-01;32(15).
748	Wilke H 2014	Repeated article	Wilke H, Van Cutsem E, Oh SC, et al. RAINBOW: A global, phase III, randomized, double-blind study of ramucirumab plus paclitaxel versus placebo plus paclitaxel in the treatment of metastatic gastroesophageal junction (GEJ) and gastric adenocarcinoma following disease progression on first-line platinum- and fluoropyrimidine-containing combination therapy rainbow IMCL CP12-0922 (I4T-IE-JVBE). J CLIN ONCOL. 2014 2014-01-01;32(3).
749	Woell E 2011	Single-arm trial	Woell E, Keil F, Thaler J, et al. Oxaliplatin, irinotecan, and bevacizumab followed by docetaxel and bevacizumab in inoperable gastric cancer: First results of a multicenter phase II trial (AGMT Gastric-3) of the Arbeitsgemeinschaft Medikamentoese Tumortherapie (AGMT). J CLIN ONCOL. 2011 2011-01-01;29(15).
750	Woell E 2012	Single-arm trial	Woell E, Thaler J, Keil F, et al. Oxaliplatin, irinotecan, bevacizumab followed by docetaxel, bevacizumab in inoperable gastric cancer: First efficacy results of a multicenter phase II trial (AGMT Gastric-3) of the Arbeitsgemeinschaft Medikamentöse Tumortherapie. J CLIN ONCOL. 2012 2012-01-01;30(15).
751	Woell E 2013	Single-arm trial	Woell E, Keil F, Thaler J, et al. Oxaliplatin, irinotecan, bevacizumab followed by docetaxel, bevacizumab in inoperable gastric cancer: Final efficacy results of a multicenter phase II trial (AGMT Gastric-3) of the arbeitsgemeinschaft Medikamentöse tumortherapie (AGMT). J CLIN ONCOL. 2013 2013-01-01;31(15).

752	Wöll E 2012	Review	Wöll E, Keil F, Thaler J, et al. Oxaliplatin, irinotecan, bevacizumab followed by docetaxel, bevacizumab in inoperable Gastric cancer. A multicenter phase II trial (AGMT Gastric-3) of the arbeitsgemeinschaft medikamentöse tumortherapie (AGMT). ANN ONCOL. 2012 2012-01-01;23:x249-50.
753	Wu J 2013	Study design inconformity	Wu J, Zhu YJ, Chen F, et al. Amorphous calcium silicate hydrate/block copolymer hybrid nanoparticles: synthesis and application as drug carriers. Dalton Trans. 2013 May 21;42(19):7032-40. doi: 10.1039/c3dt50143d.
754	Wu JY 2010	Unavailable	Wu JY, Wu XN, Ding L, et al. Phase I safety and pharmacokinetic study of bevacizumab in chinese patients with advanced cancer. CHINESE MED J-PEKING. 2010 2010-01-01;123(7):901-6.
755	Wu P 2013	Study design inconformity	Wu P, Liu Q, Li R, et al. ACS Appl Mater Interfaces. Facile preparation of paclitaxel loaded silk fibroin nanoparticles for enhanced antitumor efficacy by locoregional drug delivery. 2013 Dec 11;5(23):12638-45. doi: 10.1021/am403992b.
756	Wyrwicz L 2015	Single-arm trial	Wyrwicz L, Rybski S, Temnyk M, Kokoszynska K. Second-line palliative chemotherapy for gastric cancer: A single center cohort analysis. ANN ONCOL. 2015 2015-01-01;26:v24.
757	Xia L 2009	Review	Xia L, Guo G, Zhang B, et al. Short-term efficacy of Cetuximab-contained regimen on patients with advanced gastrointestinal (noncolorectal) cancer: Experiences of 16 patients in single institute. Chinese-German Journal of Clinical Oncology. 2009 2009-01-01;8(11):669-74.
758	Xiao J 2015	Single-arm trial	Xiao J, Chen Y, Li W, et al. Dose-dense biweekly docetaxel combined with 5-fluorouracil as first-line treatment in advanced gastric cancer: a phase II trial. Med Oncol. 2015 Feb;32(2):334. doi: 10.1007/s12032-014-0334-8.
759	Xie J 2015	Interventions inconformity	Xie J, Liang N, Qiao L, et al. Docetaxel, capecitabine and concurrent radiotherapy for gastric cancer patients with postoperative locoregional recurrence. Tumori. 2015 Jul-Aug;101(4):433-9. doi: 10.5301/tj.5000336.
760	Xie X 2013	Study design inconformity	Xie X, Huang X, Li J, et al. Efficacy and safety of Huachansu combined with chemotherapy in advanced gastric cancer: A meta-analysis. MED HYPOTHESES. 2013 2013-01-01;81(2):243-50.
761	Xu CD 2013	Single-arm trial	Xu CD. Clinical study of nimotuzumab combined with chemotherapy in the treatment of late stage gastric cancer. Asian Pacific journal of cancer prevention : APJCP. 2014 2014-01-01;15(23):10273-6.
762	Xu CD 2014	Repeated article	Xu CD.Clinical study of nimotuzumab combined with chemotherapy in the treatment of late stage gastric cancer.Asian Pac J Cancer Prev. 2014;15(23):10273-6.
763	Xu JW 2011	Repeated article	Xu JW, Li CG, Huang XE, Ubenimex capsule improves general performance and chemotherapy related toxicity in advanced gastric cancer cases.Asian Pac J Cancer Prev. 2011;12(4):985-7.

764	Xu JW 2011	Single-arm trial	Xu JW, Li CG, Huang XE, Li Y, Huo JG. Ubenimex capsule improves general performance and chemotherapy related toxicity in advanced gastric cancer cases. ASIAN PAC J CANCER P. 2011 2011-01-01;12(4):985-7.
765	Xu S 2016	Review	Xu S, Suzarte MR, Bai X, Xu B. Treatment outcome of nimotuzumab plus chemotherapy in advanced cancer patients: A single institute experience. ONCOTARGET. 2016 2016-01-01;7(22):33391-407.
766	Xu X 2013	Repeated article	Xu X, Wang L, Xu HQ, et al. Clinical comparison between paclitaxel liposome (Lipusu®) and paclitaxel for treatment of patients with metastatic gastric cancer.Asian Pac J Cancer Prev. 2013;14(4):2591-4.
767	Yakabe T 2011	Study design inconformity	Yakabe T, Noshiro H, Ikeda O, et al. Second-line chemotherapy with paclitaxel and doxifluridine after failure of S-1 in elderly patients with unresectable advanced or recurrent gastric cancer. J Cancer Res Clin Oncol. 2011 Oct;137(10):1499-504. doi:10.1007/s00432-011-1025-x.
768	Yamada K 2005	Single-arm trial	Yamada K, Sugiyama Y, Seino K, et al. A case of a nonresected gastric cancer with peritoneal dissemination maintained on TS-1 and docetaxel combination chemotherapy with good QOL. Gan To Kagaku Ryoho. 2005 Feb;32(2):223-6.
769	Yamada N 2016	Single-arm trial	Yamada N, Akai A, Nomura Y, Tanaka N. The impact and optimal indication of non-curative gastric resection for stage IV advanced gastric cancer diagnosed during surgery: 10 years of experience at a single institute. WORLD J SURG ONCOL. 2016 2016-01-01;14(1).
770	Yamada Y 2001	Single-arm trial	Yamada Y, Shirao K, Ohtsu A,et al. Phase II trial of paclitaxel by three-hour infusion for advanced gastric cancer with short premedication for prophylaxis against paclitaxel-associated hypersensitivity reactions.Ann Oncol. 2001 Aug;12(8):1133-7.
771	Yamaguchi H 2013	Single-arm trial	Yamaguchi H, Kitayama J, Ishigami H, et al.A phase 2 trial of intravenous and intraperitoneal paclitaxel combined with S-1 for treatment of gastric cancer with macroscopic peritoneal metastasis.Cancer. 2013 Sep 15;119(18):3354-8. doi: 10.1002/cncr.28204. Epub 2013 Jun 24.
772	Yamaguchi K 2002	Single-arm trial	Yamaguchi K, Tada M, Horikoshi N, et al. Phase II study of paclitaxel with 3-h infusion in patients with advanced gastric cancer. Gastric Cancer. 2002;5(2):90-5.
773	Yamamoto Y 2011	Study design inconformity	Yamamoto Y, Yoshida M, Sato M, et al. Feasibility of tailored, selective and effective anticancer chemotherapy by direct injection of docetaxel-loaded immunoliposomes into Her2/neu positive gastric tumor xenografts. Int J Oncol. 2011 Jan;38(1):33-9.
774	Yang JW 2005	Single-arm trial	Yang JW, Chen YG, Chen Q, Fan NF, Guo ZQ, Cai XC, Wu XA, Xu S, Lu X, Zhang YH, Ouyang XN. A randomized controlled trail of taxol-based combination regimens for advanced gastric cancer. Ai Zheng. 2005 Dec;24(12):1531-6.

775	Yang JW 2005	Repeated article	Yang JW, Chen YG, Chen Q, et al. A randomized controlled trail of taxol-based combination regimens for advanced gastric cancer. Ai zheng = Aizheng = Chinese journal of cancer. 2005 2005-01-01;24(12):1531-6.
776	Yano T 2009	Population inconformity	Yano T, Yamane H, Fukuoka R,et al. Evaluation of efficacy and safety of adjuvant analgesics for peripheral neuropathy induced by cancer chemotherapy in digestive cancer patients-a pilot study. Gan To Kagaku Ryoho. 2009 Jan;36(1):83-7.
777	Ye JX 2014	Review	Ye JX, Liu AQ, Ge LY, Zhou SZ, Liang ZG. Effectiveness and safety profile of S-1-based chemotherapy compared with capecitabine-based chemotherapy for advanced gastric and colorectal cancer: A meta-analysis. EXP THER MED. 2014 2014-01-01;7(5):1271-8.
778	Ye S 2008	Repeated article	Ye S, Rong J, Lin TY,et al. FOLFOX versus PLF regimen in treatment of advanced gastric adenocarcinoma.Nan Fang Yi Ke Da Xue Xue Bao. 2008 Aug;28(9):1599-602.
779	Yeh KH 2004	Single-arm trial	Yeh KH, Cheng AL. Recent advances in therapy for gastric cancer. J FORMOS MED ASSOC. 2004 2004-01-01;103(3):171-85.
780	Yeh KH 2012	Single-arm trial	Yeh KH, Chen JS, Sobrero A, et al. Safety and tolerability data from a phase II study of AUY922 compared with chemotherapy in patients with advanced gastric cancer. ANN ONCOL. 2012 2012-01-01;23:v118.
781	Yi JH 2012	Repeated article	Yi JH, Lee J, Lee J,et al.Randomised phase II trial of docetaxel and sunitinib in patients with metastatic gastric cancer who were previously treated with fluoropyrimidine and platinum.Br J Cancer. 2012 Apr 24;106(9):1469-74. doi: 10.1038/bjc.2012.100.
782	Yi JH 2012	Repeated article	Yi JH, Lee J, Lee J, et al. Randomised phase II trial of docetaxel and sunitinib in patients with metastatic gastric cancer who were previously treated with fluoropyrimidine and platinum. BRIT J CANCER. 2012 2012-01-01;106(9):1469-74.
783	Yoon S 2013	Review	Yoon S, Ryu MH, Ryoo BY, et al. Five-year outcomes of a phase II study of adjuvant chemotherapy with docetaxel, capecitabine, and cisplatin in stage IIIB-IV(M0) gastric cancer patients. EUR J CANCER. 2013 2013-01-01;49:S633.
784	Yoon S 2017	Single-arm trial	Yoon S, Yoo C, Ryu MH, et al. Phase 2 study of adjuvant chemotherapy with docetaxel, capecitabine, and cisplatin in patients with curatively resected stage IIIB–IV gastric cancer. GASTRIC CANCER. 2017 2017-01-01;20(1):182-9.
785	Yoshida K 2006	Single-arm trial	Yoshida K, Ninomiya M, Takakura N, et al. Phase II study of docetaxel and S-1 combination therapy for advanced or recurrent gastric cancer. Clin Cancer Res. 2006 Jun 1;12(11 Pt 1):3402-7.
786	Yoshida K 2006	Single-arm trial	Yoshida K, Ninomiya M, Takakura N, et al. Phase II study of docetaxel and S-1 combination therapy for advanced or recurrent gastric cancer. CLIN CANCER RES. 2006 2006-01-01;12(11 I):3402-7.

787	Yoshida K 2008	Study design inconformity	Yoshida K, Yamaguchi K, Osada S, et al. Challenge for a better combination with basic evidence. Int J Clin Oncol. 2008 Jun;13(3):212-9. doi: 10.1007/s10147-008-0793-z.
788	Yoshida K 2012	Single-arm trial	Yoshida K, Fujii M, Koizumi W, et al. S-1 plus docetaxel versus S-1 for advanced gastric cancer (start trial) update 2012 (Jaccro and KCSG study group). ANN ONCOL. 2012 2012-01-01;23:x12.
789	Yoshida K 2016	Review	Yoshida K, Yamaguchi K, Okumura N, Tanahashi T, Kodera Y. Is conversion therapy possible in stage IV gastric cancer: the proposal of new biological categories of classification. GASTRIC CANCER. 2016 2016-01-01;19(2):329-38.
790	Yoshida M 2009	Single-arm trial	Yoshida M, Sato T, Takiuchi H, et al. Phase II study of weekly paclitaxel as thirrd line chemotherapy for advanced or recurrent gastric cancer (Osaka gastrointestinal cancer chemotherapy study group: OGSG0602). European Journal of Cancer, Supplement. 2009 2009-01-01;7(2-3):376-7.
791	Yoshida M 2010	Study design inconformity	Yoshida M, Watanabe Y, Sato M, et al.Feasibility of chemohyperthermia with docetaxel-embedded magnetoliposomes as minimally invasive local treatment for cancer. Int J Cancer. 2010 Apr 15;126(8):1955-65. doi: 10.1002/ijc.24864.
792	Yoshida M 2012	Study design inconformity	Yoshida M, Sato M, Yamamoto Y, et al. Tumor local chemohyperthermia using docetaxel-embedded magnetoliposomes:Interaction of chemotherapy and hyperthermia. J Gastroenterol Hepatol. 2012 Feb;27(2):406-11. doi:10.1111/j.1440-1746.2011.06972.x.
793	Yoshikawa T 2010	Study design inconformity	Yoshikawa T, Tsuburaya A, Morita S,et al.A comparison of multimodality treatment: two or four courses of paclitaxel plus cisplatin or S-1 plus cisplatin followed by surgery for locally advanced gastric cancer, a randomized Phase II trial (COMPASS).Jpn J Clin Oncol. 2010 Apr;40(4):369-72. doi: 10.1093/jjco/hyp178. Epub 2010 Mar 18.
794	Yoshikawa T 2010	Review	Yoshikawa T, Tsuburaya A, Morita S, et al. A comparison of multimodality treatment: Two or four courses of paclitaxel plus cisplatin or S-1 plus cisplatin followed by surgery for locally advanced gastric cancer, a randomized phase II trial (COMPASS). JPN J CLIN ONCOL. 2010 2010-01-01;40(4):369-72.
795	Yoshikawa T 2012	Study design inconformity	Yoshikawa T, Taguri M, Sakuramoto S, et al.A comparison of multimodality treatment: two and four courses of neoadjuvant chemotherapy using S-1/CDDP or S-1/CDDP/docetaxel followed by surgery and S-1 adjuvant chemotherapy for macroscopically resectable serosa-positive gastriccancer: a randomized phase II trial (COMPASS-D trial).Jpn J Clin Oncol. 2012 Jan;42(1):74-7. doi: 10.1093/jjco/hyr166. Epub 2011 Nov 17.

796	Yoshikawa T 2012	Review	Yoshikawa T, Taguri M, Sakuramoto S, et al. A comparison of multimodality treatment: Two and four courses of neoadjuvant chemotherapy using S-1/CDDP or S-1/CDDP/Docetaxel followed by surgery and S-1 adjuvant chemotherapy for macroscopically resectable serosa-positive gastric cancer: A randomized phase ii trial (COMPASS-D trial). JPN J CLIN ONCOL. 2012 2012-01-01;42(1):74-7.
797	Yoshikawa T 2014	Repeated article	Yoshikawa T, Tanabe K, Nishikawa K,et al. Induction of a pathological complete response by four courses of neoadjuvant chemotherapy for gastric cancer: early results of the randomized phase II COMPASS trial. Ann Surg Oncol. 2014 Jan;21(1):213-9. doi: 10.1245/s10434-013-3055-x.
798	Yoshikawa T 2014	Single-arm trial	Yoshikawa T, Tanabe K, Nishikawa K, et al. Accuracy of CT staging of locally advanced gastric cancer after neoadjuvant chemotherapy: cohort evaluation within a randomized phase II study. Ann Surg Oncol. 2014 Jun;21 Suppl 3:S385-9. doi: 10.1245/s10434-014-3615-8.
799	Yoshikawa T 2015	Repeated article	Yoshikawa T, Tanabe K, Ito Y, et al. Subset analysis of COMPASS: A randomized 2X2 phase II trial comparing two and four courses of S-1/cisplatin (SC) and paclitaxel/cisplatin (PC) as neoadjuvant chemotherapy for locally advanced gastric cancer. J CLIN ONCOL. 2015 2015-01-01;33(15).
800	Yoshikawa T 2015	Repeated article	Yoshikawa T, Fujitani K, Nishikawa K, et al. Comparison of chemotherapy-related toxicities in a randomized 2X2 phase II trial comparing two and four courses of cisplatin/S-1 (CS) and docetaxel/cisplatin/S-1 (DCS) as neoadjuvant chemotherapy for locally advanced gastric cancer. EUR J CANCER. 2015 2015-01-01;51:S407.
801	Yoshikawa T 2015	Repeated article	Yoshikawa T, Tanabe K, Nishikawa K, et al. A randomized 2X2 phase II trial comparing two and four courses of S-1/cisplatin (SC) and paclitaxel/cisplatin (PC) as neoadjuvant chemotherapy for locally resectable advanced gastric cancer: Survival results of COMPASS. J CLIN ONCOL. 2015 2015-01-01;33(3).
802	Yoshikawa T 2016	Repeated article	Yoshikawa T, Morita S, Tanabe K, et al. Survival results of a randomised two-by-two factorial phase II trial comparing neoadjuvant chemotherapy with two and four courses of S-1 plus cisplatin (SC) and paclitaxel plus cisplatin (PC) followed by D2 gastrectomy for resectable advanced gastric cancer. EUR J CANCER. 2016 2016-01-01;62:103-11.
803	Yoshino S 2012	Single-arm trial	Yoshino S, Tsuburaya A, Kobayashi M, et al. Long-term follow up of a feasibility study of the factorial phase III samit trial: Adjuvant paclitaxel followed by S1 for gastric cancer. ANN ONCOL. 2012 2012-01-01;23:x228.
804	Yoshino S 2013	Single-arm trial	Yoshino S, Furuya T, Shimizu R, et al. Combination phase II study of weekly paclitaxel and 5'-DFUR for unresectable or recurrent gastric cancer. ANTICANCER RES. 2013 2013-01-01;33(6):2629-34.

805	Yoshioka T 2003	Single-arm trial	Yoshioka T, Sakata Y, Terashima M, et al. Biweekly administration regimen of docetaxel combined with CPT-11 in patients with inoperable or recurrent gastric cancer. Gastric Cancer. 2003;6(3):153-8.
806	Yu J 2012	Study design inconformity	Yu J, Lee HJ, Hur K,et al. The antitumor effect of a thermosensitive polymeric hydrogel containing paclitaxel in a peritoneal carcinomatosis model. Invest New Drugs. 2012 Feb;30(1):1-7. doi: 10.1007/s10637-010-9499-y.
807	Yu J 2016	Study design inconformity	Yu J, Zhang Y, Leung LH, Liu L, Yang F, Yao X. Efficacy and safety of angiogenesis inhibitors in advanced gastric cancer: A systematic review and meta-analysis. Journal of Hematology and Oncology. 2016 2016-01-01;9(1).
808	Yue J 2012	Study design inconformity	Yue J, Liu S, Wang R, et al. Transferrin-conjugated micelles: enhanced accumulation and antitumor effect for transferrin-receptor-overexpressing cancer models. Mol Pharm. 2012 Jul 2;9(7):1919-31. doi: 10.1021/mp300213g.
809	Zahir MN 2017	Review	Zahir MN, Shaikh Q, Shabbir-Moosajee M, Jabbar AA. Incidence of Venous Thromboembolism in cancer patients treated with Cisplatin based chemotherapy - a cohort study. BMC CANCER. 2017 2017-01-01;17(1).
810	Zang D 2011	Review	Zang D, Kang Y, Ryoo B, et al. Phase II study with docetaxel, oxaliplatin and S-1 (DOS) combination chemotherapy for patients with metastatic gastric cance. ANN ONCOL. 2010 2010-01-01;21:i89.
811	Zang DY 2009	Single-arm trial	Zang DY, Yang DH, Lee HW, et al. Phase I/II trial with docetaxel and S-1 for patients with advanced or recurrent gastric cancer with consideration to age. Cancer Chemother Pharmacol. 2009 Feb;63(3):509-16. doi:10.1007/s00280-008-0768-9.
812	Zang DY 2010	Single-arm trial	Zang DY, Kang Y, Ryoo B, et al. Phase II study with docetaxel, oxaliplatin and s-1 combination chemotherapy for patients with metastatic gastric cancer. ANN ONCOL. 2010 2010-01-01;21:i261.
813	Zhan YP 2012	Interventions inconformity	Zhan YP, Huang XE, Cao J,et al. Clinical safety and efficacy of Kanglaite® (Coix Seed Oil) injection combined with chemotherapy in treating patients with gastric cancer. Asian Pac J Cancer Prev. 2012;13(10):5319-21.
814	Zhang CH 2013	Study design inconformity	Zhang CH, Awasthi N, Schwarz MA,et al.The dual PI3K/mTOR inhibitor NVP-BEZ235 enhances nab-paclitaxel antitumor response in experimental gastric cancer.Int J Oncol. 2013 Nov;43(5):1627-35. doi: 10.3892/ijo.2013.2099. Epub 2013 Sep 13.
815	Zhang J 2009	Repeated article	Zhang J, Xiao Y, Lu M, et al. Retrospective study on regimens of capecitabine-based chemotherapy in the treatment for advanced gastric cancer. Zhonghua Zhong Liu Za Zhi. 2009 Apr;31(4):312-5.
816	Zhang J 2015	Single-arm trial	Zhang J, Xie J, Liang N, et al. Docetaxel, capecitabine and concurrent radiotherapy for gastric cancer patients with postoperative locoregional recurrence. TUMORI. 2015 2015-01-01;101(4):433-9.
817	Zhang Q 2011	Interventions inconformity	Zhang Q, Tey J, Peng L, et al. Adjuvant chemoradiotherapy with or without intraoperative radiotherapy for the treatment of resectable locally advanced gastric adenocarcinoma. Radiother Oncol. 2012 Jan;102(1):51-5. doi: 10.1016/j.radonc.2011.10.008.

818	Zhang Q 2012	Repeated article	Zhang Q, Tey J, Peng L, et al. Adjuvant chemoradiotherapy with or without intraoperative radiotherapy for the treatment of resectable locally advanced gastric adenocarcinoma. RADIOTHER ONCOL. 2012 2012-01-01;102(1):51-5.
819	Zhang X 2011	Single-arm trial	Zhang X, Shen L, Lu Z, et al. A randomized, multicenter, open-label, phase III study to compare the efficacy and safety of capecitabine plus paclitaxel followed by capecitabine maintenance (PX-X) with capecitabine plus cisplatin (XP) as a first-line chemotherapy for recurrent or metastatic gastric cancer (PAC-C study). EUR J CANCER. 2011 2011-01-01;47:S471-2.
820	Zhang X 2015	Single-arm trial	Zhang X, Shen L, Lu Z, et al. Comparison of efficacy and safety of paclitaxel and capecitabine followed by capecitabine as maintenance therapy versus cisplatin and capecitabine therapy for advanced gastric cancer: A multicentre, randomised, active-controlled phase III study. ANN ONCOL. 2015 2015-01-01;26:x44.
821	Zhang XT 2013	Single-arm trial	Zhang XT, Li J, Bai Y,et al.A phase II study of triweekly paclitaxel and capecitabine combination therapy in patients with fluoropyrimidine-platinum-resistant metastatic gastric adenocarcinoma. J Cancer Res Ther. 2013 Nov;9 Suppl:S153-7. doi: 10.4103/0973-1482.122512.
822	Zhang XT 2013	Single-arm trial	Zhang XT, Li J, Bai Y, et al. A phase II study of triweekly paclitaxel and capecitabine combination therapy in patients with fluoropyrimidine-platinum-resistant metastatic gastric adenocarcinoma. J CANCER RES THER. 2013 2013-01-01;9(SUPPL.3):S151-5.
823	Zhang XT 2016	Interventions inconformity	Zhang XT, Zhang Z, Liu L, Xin YN, Xuan SY. Comparative study of different neoadjuvant therapy regimen in locally advanced gastric cancer. Chinese Journal of Cancer Prevention and Treatment. 2016 2016-01-01;23(11):739-43.
824	Zhao AG 2010	Interventions inconformity	Zhao AG, Cao W, Xu Y, et al. Survival benefit of an herbal formula for invigorating spleen for elderly patients with gastric cancer. Journal of Chinese Integrative Medicine. 2010 2010-01-01;8(3):224-30.
825	Zhao JM 2007	Interventions inconformity	Zhao JM, Wu AZ, Shi LR. Clinical observation on treatment of advanced gastric cancer by combined use of Shenqi Fuzheng injection, docetaxel, flurouracil and calcium folinate. Zhongguo Zhong Xi Yi Jie He Za Zhi. 2007 Aug;27(8):736-8.
826	Zhao L 2010	Single-arm trial	Zhao L, Ying H, Ning X, et al. Effect of adjuvant chemotherapy with docetaxel, cisplatin, and continuous infusion fluorouracil for gastric cancer: A phase II study. J CLIN ONCOL. 2010 2010-01-01;28(15).
827	Zhao XL 2016	Repeated article	Zhao XL, Zhang XY, Gao JH. Clinical efficacy of paclitaxel in the treatment of mid-stage and advanced Malignant gastric cancer, and effect of nursing interventions. TROP J PHARM RES. 2016 2016-01-01;15(9):2035-9.

828	Zheng LZ 2011	Study design inconformity	Zheng LZ, Gu JC, Gong JF, et al. Relationship between single nucleotide polymorphisms of candidate genes and survival of patients with gastric cancer undergoing chemotherapy with capecitabine and paclitaxel. Journal of Shanghai Jiaotong University (Medical Science). 2011 2011-01-01;31(5):598-603.
829	Zheng Y 2014	Single-arm trial	Zheng Y, Fang W, Mao C,et al. Biweekly S-1 plus paclitaxel (SPA) as second-line chemotherapy after failure from fluoropyrimidine and platinum in advanced gastric cancer: a phase II study. Cancer Chemother Pharmacol. 2014 Sep;74(3):503-9. doi: 10.1007/s00280-014-2537-2.
830	Zheng Y 2014	Single-arm trial	Zheng Y, Fang W, Mao C, et al. Biweekly S-1 plus paclitaxel (SPA) as second-line chemotherapy after failure from fluoropyrimidine and platinum in advanced gastric cancer: A phase II study. CANCER CHEMOTH PHARM. 2014 2014-01-01;74(3):503-9.
831	Zhibing W 2013	Interventions inconformity	Zhibing W, Qinghua D, Shenglin M, et al. Clinical study of cisplatin hyperthermic intraperitoneal perfusion chemotherapy in combination with docetaxel, 5-flourouracil and leucovorin intravenous chemotherapy for the treatment of advanced-stage gastric carcinoma. Hepatogastroenterology. 2013 Jul-Aug;60(125):989-94. doi: 10.5754/hge13038.
832	Zhibing W 2013	Population inconformity	Zhibing W, Qinghua D, Shenglin M, et al. Clinical study of cisplatin hyperthermic intraperitoneal perfusion chemotherapy in combination with docetaxel, 5-flourouracil and leucovorin intravenous chemotherapy for the treatment of advanced-stage gastric carcinoma. HEPATO-GASTROENTEROL. 2013 2013-01-01;60(125):989-94.
833	Zhong H 2008	Single-arm trial	Zhong H, Zhang Y, Ma S,et al. Docetaxel plus oxaliplatin (DOCOX) as a second-line treatment after failure of fluoropyrimidine and platinum in Chinese patients with advanced gastric cancer. Anticancer Drugs. 2008 Nov;19(10):1013-8. doi: 10.1097/CAD.0b013e328314b5ab.
834	Zou ZY 2012	Study design inconformity	Zou ZY, Wei J, Li XL, et al. Enhancement of anticancer efficacy of chemotherapeutics by gambogic acid against gastric cancer cells. CANCER BIOTHER RADIO. 2012 2012-01-01;27(5):299-306.
835	Zuo PY 2014	Single-arm trial	Zuo PY, Chen XL, Liu YW, Xiao CL, Liu CY. Increased risk of cerebrovascular events in patients with cancer treated with bevacizumab: A meta-analysis. PLOS ONE. 2014 2014-01-01;9(7).

The network meta-analysis results of regimens for outcomes (OR/95%CI)

Comparison	OS	PFS	ORR	neutropenia	leukopenia	vomiting	fatigue
CFvs.ECF	1.04(0.49,2.16)	1.38(0.254,7.32	1.12(0.64,1.94)	6.69(0.52,84.02)	2.16(0.28,15.81)	0.37(0.13,1.001)	0.88(0.081,10.75)
CFvs.EOF	1.15(0.50,2.67)	2.56(0.45,14.49)	**3.26(1.13,9.66)**	1.56(0.1289,18.05)	**40.09(1.01,1741)**	0.16(0.0047,1.26)	5.52(0.45,94.63)
CFvs.F	1.14(0.3699,2.839)	0.11(0.00096,5.99)	0.28(0.033,1.29)	3.91(0.25,56.9)	0.63(0.12,3.30)	3.53(0.28,113.7)	2.11(0.022,204.6)
CFvs.I	0.56(0.13,1.99)	2.38(0.050,95.94)	0.78(0.25,2.48)	4.03(0.23,64.87)	0.99(0.065,15.75)	0.22(0.0035,20.92)	--
CFvs.IC	0.37(0.0077,5.88)	1.15(0.0099,122.6)	2.15(0.77,6.25)	--	1.38(0.10,16.97)	1.96(0.23,24.22)	4.11(0.073,338.1)
CFvs.IF	1.41(0.35,5.31)	--	1.45(0.84,2.59)	--	2.43(0.21,29.71)	0.11(0.01257,0.939)	10.56(0.09519,1200)
CFvs.OF	1.28(0.60,2.78)	3.36(0.13,105.6)	1.89(0.52,6.46)	1.73(0.21,15.37)	0.95(0.19,4.40)	0.67(0.213,2.09)	2.89(0.027,401.7)
CFvs.RT	1.30(0.39,3.77)	2.80(0.10,66.41)	0.94(0.27,2.99)	**13.6(1.31,134.9)**	3.19(0.36,29.96)	0.99(0.060,46.41)	7.67(0.18,562.3)
CFvs.T	0.79(0.26,2.15)	1.09(0.055,18.95)	1.14(0.73,1.81)	2.53(0.31,19.39)	1.07(0.16,7.45)	1.06(0.069,45.32)	3.25(0.10,175.9)
CFvs.TC	0.78(0.42,1.44)	1.00(0.084,10.94)	**1.84(1.33,2.60)**	3.53(0.82,14.3)	1.45(0.31,6.01)	0.68(0.28,1.64)	2.72(0.15,71.33)
CFvs.TCF	1.32(0.81,2.10)	1.22(0.33,4.55)	**1.81(1.06,3.15)**	3.17(0.96,10.08)	1.93(0.66,5.14)	0.81(0.49,1.45)	2.53(0.40,18.35)
CFvs.TF	2.16(0.93,4.76)	1.48(0.16,14.69)	**2.63(1.57,4.42)**	3.78(0.23,57.39)	1.81(0.51,6.51)	0.49(0.14,2.06)	3.05(0.076,119.2)
CFvs.TO	0.53(0.24,1.23)	1.16(0.10,14.46)	**2.57(1.43,4.59)**	**9.09(2.01,41.02)**	2.40(0.56,8.88)	0.29(0.11,0.90)	4.08(0.13,159.9)
CFvs.TOF	1.53(0.67,3.55)	4.45(0.36,70.37)	1.58(0.73,3.49)	**8.58(1.10,71.92)**	2.40(0.50,10.95)	2.01(0.58,8.24)	4.83(0.11,262)
CFvs.mCF	0.76(0.31,1.78)	--	1.97(1.19,3.37)	--	--	--	--
CFvs.mTCF	1.62(0.87,2.85)	1.72(0.36,9.13)	0.83(0.36,1.87)	0.58(0.16,1.95)	**75.34(7.90,1085)**	0.44(0.17,1.116)	2.4(0.017,258.6)
CFvs.mTF	1.34(0.50,3.48)	1.10(0.10,11.81)	1.69(0.72,3.94)	16(0.46,569.1)	1.25(0.16,9.26)	0.37(0.041,2.96)	0.60(0.017,20.36)
CFvs.mTOF	0.60(0.20,1.83)	4.41(0.25,114.9)	--	**174.7(12.56,3152)**	2.21(0.25,18.32)	0.44(0.011,5.46)	6.34(0.099,511.9)
ECFvs.EOF	1.10(0.42,2.91)	1.87(0.29,12.24)	2.92(0.99,8.883)	0.23(0.0074,6.88)	18.55(0.28,142)	0.44(0.011,3.89)	6.35(0.45,105.9)
ECFvs.F	1.09(0.35,2.89)	0.081(0.00071,4.27)	0.25(0.028,1.22)	0.58(0.019,16.84)	0.29(0.031,2.92)	9.70(0.71,318.1)	2.44(0.030,170.7)

ECFvs.I	0.53(0.13,1.99)	1.73(0.037,67.4)	0.69(0.20,2.33)	0.60(0.018,18.53)	0.45(0.021,10.8)	0.61(0.0086,59.78)	--
ECFvs.IC	0.35(0.0076,5.98)	0.83(0.0072,89.85)	1.91(0.662,5.85)	--	0.64(0.032,11.95)	5.43(0.58,68.7)	4.71(0.080,344.3)
ECFvs.IF	1.35(0.33,5.36)	--	1.30(0.69,2.52)	--	1.13(0.068,20.01)	0.29(0.030,2.93)	12(0.13,1052)
ECFvs.OF	1.23(0.52,3.07)	2.45(0.10,70.48)	1.68(0.45,6.20)	0.26(0.015,4.98)	0.44(0.050,3.67)	1.81(0.48,7.67)	3.28(0.034,352.9)
ECFvs.RT	1.25(0.36,3.89)	2.04(0.077,44.92)	0.83(0.24,2.87)	2.03(0.093,43.29)	1.49(0.1062,21.75)	2.71(0.14,146.9)	8.78(0.22,518.1)
ECFvs.T	0.76(0.23,2.2)	0.79(0.041,12.81)	1.02(0.59,1.81)	0.38(0.020,6.59)	0.50(0.045,5.84)	2.92(0.17,146.5)	3.72(0.13,154.4)
ECFvs.TC	0.74(0.33,1.72)	0.72(0.063,7.64)	1.65(1.02,2.67)	0.53(0.045,5.76)	0.67(0.077,5.41)	1.88(0.66,5.40)	3.13(0.18,67.3)
ECFvs.TCF	1.26(0.66,2.46)	0.89(0.22,3.62)	1.60(0.88,3.08)	0.47(0.049,4.43)	0.89(0.14,5.28)	2.23(0.92,5.97)	2.89(0.39,21.48)
ECFvs.TF	2.07(0.87,4.86)	1.09(0.13,8.90)	**2.36(1.24,4.47)**	0.56(0.018,16.79)	0.84(0.13,5.66)	1.34(0.31,6.63)	3.47(0.11,93.61)
ECFvs.TO	0.51(0.21,1.31)	0.86(0.078,9.62)	**2.29(1.17,4.53)**	1.36(0.10,17.73)	1.10(0.14,8.60)	0.81(0.22,3.20)	4.63(0.17,138.9)
ECFvs.TOF	1.47(0.58,3.76)	3.27(0.28,45.84)	1.42(0.60,3.49)	1.29(0.071,24.74)	1.10(0.13,9.46)	5.50(1.31,29.62)	5.50(0.15,221.5)
ECFvs.mCF	0.72(0.24,2.11)	--	1.77(0.87,3.69)	--	--	1.19(0.32,4.48)	--
ECFvs.mTCF	1.55(0.64,3.64)	1.25(0.20,9.09)	0.74(0.35,1.56)	0.086(0.0057,1.22)	35.27(1.73,1004)	--	2.72(0.018,287.9)
ECFvs.mTF	1.28(0.54,2.99)	0.80(0.11,5.64)	1.51(0.60,3.76)	2.39(0.040,148.8)	0.57(0.11,3.18)	0.99(0.12,7.74)	0.68(0.033,11.78)
ECFvs.mTOF	0.57(0.18,1.88)	3.23(0.19,75.81)	--	26.44(0.90,919.9)	1.02(0.074,13.98)	1.19(0.028,17.23)	7.25(0.12,451.1)
EOFvs.F	0.98(0.27,3.11)	0.043(0.00033,2.66)	**0.085(0.0079,0.53)**	2.5(0.07101,86.32)	**0.016(0.00025,0.88)**	25.08(0.80,229)	0.38(0.0026,40.39)
EOFvs.I	0.49(0.099,2.05)	0.93(0.017,42.44)	0.24(0.049,1.08)	2.54(0.069,99.47)	0.025(0.00023,2.54)	1.52(0.014,527.2)	--
EOFvs.IC	0.31(0.0065,5.75)	0.45(0.0034,54.02)	0.66(0.15,2.78)	--	0.034(0.00034,2.92)	13.69(0.61,782.7)	0.74(0.0081,71.95)
EOFvs.IF	1.23(0.27,5.52)	--	0.4(0.14,1.38)	--	0.061(0.00066,5.25)	0.71(0.035,38.87)	1.89(0.011,249.1)
EOFvs.OF	1.12(0.40,3.16)	1.32(0.045,45.93)	0.58(0.10,2.71)	1.12(0.050,27.56)	0.024(0.00038,1.24)	4.18(0.42,164.9)	0.52(0.0031,76.27)
EOFvs.RT	1.13(0.28,4.16)	1.09(0.033,29.91)	0.29(0.053,1.27)	8.66(0.33,235)	0.079(0.0010,5.91)	6.78(0.20,1336)	1.37(0.021,120.1)
EOFvs.T	0.69(0.18,2.37)	0.43(0.017,8.64)	0.35(0.12,1.06)	1.61(0.072,36.27)	0.027(0.00039,1.71)	7.22(0.23,1351)	0.58(0.012,38.51)

EOFvs.TC	0.67(0.26,1.79)	0.39(0.027,5.23)	0.56(0.19,1.60)	2.25(0.15,33.92)	0.036(0.00060,1.84)	4.35(0.48,153.5)	0.49(0.016,17.1)
EOFvs.TCF	1.14(0.50,2.62)	0.48(0.092,2.46)	0.55(0.17,1.69)	2.02(0.16,27.03)	0.048(0.00092,2.11)	5.23(0.66,175.8)	0.46(0.034,5.10)
EOFvs.TF	1.87(0.63,5.38)	0.58(0.051,6.91)	0.80(0.26,2.45)	2.42(0.065,86.26)	0.045(0.00082,2.23)	3.20(0.28,137.5)	0.55(0.0090,25.92)
EOFvs.TO	0.46(0.16,1.40)	0.46(0.032,6.73)	0.79(0.24,2.49)	5.82(0.37,94.65)	0.060(0.0010,2.92)	1.83(0.19,73.55)	0.73(0.014,35.09)
EOFvs.TOF	1.34(0.45,4.04)	1.75(0.12,32.05)	0.49(0.13,1.73)	5.52(0.26,131.7)	0.060(0.00096,3.17)	**13.35(1.15,518.5)**	0.87(0.013,56.51)
EOFvs.mCF	0.66(0.20,2.11)	--	0.60(0.19,1.98)	--	--	--	--
EOFvs.mTCF	1.39(0.60,3.22)	0.67(0.13,3.91)	**0.25(0.072,0.91)**	0.37(0.044,3.04)	1.92(0.13,34.16)	2.78(0.29,103.3)	0.43(0.0059,17.26)
EOFvs.mTF	1.17(0.35,3.74)	0.43(0.034,5.38)	0.52(0.13,1.88)	10.25(0.15,748.8)	0.031(0.00041,2.11)	2.34(0.12,146.9)	0.11(0.0020,4.30)
EOFvs.mTOF	0.52(0.14,1.97)	1.73(0.081,50.02)	--	**113.3(3.41,4490)**	0.055(0.00069,3.74)	2.93(0.043,173.8)	1.13(0.011,104.2)
Fvs.I	0.49(0.16,1.63)	21.26(0.53,1662)	2.76(0.41,30.96)	1.04(0.081,13.11)	1.57(0.11,21.2)	0.060(0.00036,9.13)	--
Fvs.IC	0.33(0.0069,5.58)	10.74(0.051,3488)	7.83(1.34,78.44)	--	2.20(0.13,33.22)	0.57(0.010,17.12)	1.92(0.018,327.9)
Fvs.IF	1.25(0.38,4.58)	--	5.13(1.12,46.13)	--	3.87(0.30,50.37)	**0.029(0.00083,0.46)**	4.98(0.090,304.5)
Fvs.OF	1.13(0.46,3.45)	30.54(0.50,44)	6.73(1.01,70.23)	0.45(0.024,9.42)	1.50(0.22,9.19)	0.19(0.0053,2.79)	1.35(0.016,150.2)
Fvs.RT	1.15(0.47,2.94)	24.38(1.07,1227)	3.32(0.52,33.54)	3.49(0.46,27.35)	5.09(0.65,39.74)	0.28(0.0051,22.73)	3.52(0.068,358.4)
Fvs.T	0.70(0.31,1.62)	9.42(0.58,370.1)	4.08(0.85,36.44)	0.65(0.11,3.73)	1.71(0.29,9.63)	0.30(0.0059,23.48)	1.50(0.038,112.1)
Fvs.TC	0.68(0.28,1.96)	8.77(0.30,567.7)	**6.49(1.44,57.02)**	0.91(0.079,10.42)	2.30(0.34,13.47)	0.19(0.0059,2.57)	1.27(0.030,84.02)
Fvs.TCF	1.15(0.50,3.23)	10.99(0.241,1097)	**6.44(1.46,54.55)**	0.82(0.067,10.17)	3.05(0.57,14.79)	0.23(0.0076,2.83)	1.20(0.018,93.62)
Fvs.TF	1.88(1.09,3.91)	13.42(0.33,1179)	**9.32(2.02,81.72)**	0.97(0.19,4.73)	2.88(0.71,11.33)	0.15(0.0064,1.25)	1.43(0.093,22.64)
Fvs.TO	0.46(0.22,1.25)	10.46(0.34,714.9)	**9.00(1.96,80.7)**	2.34(0.21,27.68)	3.81(0.63,20.77)	0.086(0.0027,1.08)	1.89(0.063,77.3)
Fvs.TOF	1.35(0.62,3.69)	**41.09(1.09,3852)**	**5.69(1.03,54.36)**	2.22(0.13,41.2)	3.80(0.59,22.72)	0.61(0.021,6.70)	2.27(0.069,97.08)
Fvs.mCF	0.67(0.21,2.47)	--	**7.08(1.38,62.19)**	--	--	--	--
Fvs.mTCF	1.42(0.50,4.60)	15.66(0.28,2070)	2.99(0.58,27.89)	0.15(0.0084,2.606)	**120.6(7.203,2709)**	0.13(0.0037,1.74)	1.10(0.0021,592)

Fvs.mTF	1.18(0.44,3.58)	10.06(0.17,1158)	5.94(1.10,57.33)	4.07(0.26,68.78)	1.98(0.22,17.27)	0.10(0.0023,1.991)	0.28(0.0048,15.51)
Fvs.mTOF	0.52(0.19,1.87)	41.09(0.87,49)	--	45.58(1.66,1546)	3.49(0.31,35.72)	0.12(0.00099,3.40)	2.96(0.053,216.5)
Ivs.IC	0.64(0.013,13.07)	0.49(0.0028,78.46)	2.77(0.62,12.36)	--	1.41(0.041,38.95)	8.62(0.064,1060)	--
Ivs.IF	2.49(0.53,12.9)	--	1.90(0.57,6.27)	--	2.48(0.085,67.65)	0.46(0.0034,49.14)	--
Ivs.OF	2.31(0.66,9.41)	1.41(0.032,95)	2.36(0.45,12.31)	0.43(0.021,9.93)	0.96(0.053,15.68)	2.87(0.032,201.5)	--
Ivs.RT	2.33(0.93,5.91)	1.19(0.068,18.42)	1.18(0.23,5.83)	3.38(0.40,30.05)	3.24(0.34,30.28)	4.22(0.36,112.9)	--
Ivs.T	1.42(0.62,3.31)	0.47(0.039,4.93)	1.47(0.51,4.24)	0.63(0.095,4.09)	1.08(0.15,7.57)	4.43(0.42,117)	--
Ivs.TC	1.40(0.41,5.36)	0.42(0.019,9.93)	2.38(0.78,7.36)	0.88(0.068,11.18)	1.47(0.089,20.21)	3.07(0.032,203.3)	--
Ivs.TCF	2.34(0.71,9.25)	0.51(0.015,19.96)	2.33(0.70,7.77)	0.79(0.058,10.82)	1.96(0.12,26.67)	3.71(0.040,231)	--
Ivs.TF	**3.88(1.27,12.98)**	0.62(0.022,22.52)	**3.42(1.04,11.04)**	0.94(0.066,12.66)	1.85(0.13,23.58)	2.17(0.024,153.4)	--
Ivs.TO	0.95(0.31,3.45)	0.49(0.022,13.12)	3.33(0.97,11.07)	2.27(0.18,29.1)	2.43(0.15,35.98)	1.30(0.018,72.53)	--
Ivs.TOF	2.74(0.86,10.41)	1.89(0.070,70.36)	2.05(0.56,7.49)	2.15(0.11,43.65)	2.43(0.14,38.37)	9.15(0.094,597.8)	--
Ivs.mCF	1.34(0.32,6.60)	--	2.54(0.74,8.94)	--	--	--	--
Ivs.mTCF	2.86(0.74,12.87)	0.72(0.017,40.32)	1.08(0.27,4.02)	0.14(0.0074,2.74)	**78.15(2.09,3545)**	1.97(0.019,134.2)	--
Ivs.mTF	2.42(0.62,10.17)	0.47(0.010,23.36)	2.19(0.55,8.24)	3.98(0.13,134.4)	1.27(0.057,27.14)	1.56(0.010,166)	--
Ivs.mTOF	1.06(0.28,4.98)	1.90(0.056,99.26)	--	**44.00(1.53,1576)**	2.22(0.086,53.48)	1.74(0.0078,208.7)	--
ICvs.IF	3.88(0.19,216.4)	--	0.68(0.24,1.88)	--	1.76(0.067,52.36)	0.053(0.0025,1.09)	2.54(0.014,334.7)
ICvs.OF	3.56(0.22,174.2)	3.01(0.022,480.8)	0.88(0.18,3.79)	--	0.68(0.048,10.28)	0.34(0.024,3.55)	0.69(0.0047,92.41)
ICvs.RT	3.57(0.20,170.3)	2.41(0.022,275.4)	0.44(0.094,1.81)	--	2.30(0.12,51.1)	0.53(0.011,35.77)	1.85(0.035,117.8)
ICvs.T	2.18(0.12,101.5)	0.94(0.011,87.14)	0.53(0.18,1.51)	--	0.77(0.050,13.80)	0.56(0.0133,34.77)	0.78(0.020,36.24)
ICvs.TC	2.18(0.14,96.01)	0.86(0.016,53.07)	0.86(0.30,2.33)	--	1.04(0.13,8.43)	0.35(0.033,2.49)	0.66(0.036,11.81)
ICvs.TCF	3.62(0.23,170.6)	1.06(0.012,105.2)	0.84(0.33,2.05)	--	1.39(0.13,15.70)	0.42(0.036,3.47)	0.62(0.012,25.35)

ICvs.TF	5.84(0.37,280.10)	1.30(0.014,136.2)	1.22(0.43,3.31)	--	1.31(0.11,17.28)	0.25(0.017,3.13)	0.74(0.010,34.62)
ICvs.TO	1.44(0.092,70.06)	1.02(0.013,87.48)	1.20(0.41,3.24)	--	1.73(0.14,21.75)	0.15(0.012,1.56)	0.981(0.023,36.19)
ICvs.TOF	4.25(0.26,208.1)	3.94(0.045,426.7)	0.74(0.21,2.58)	--	1.72(0.12,24.85)	1.02(0.076,12.45)	1.17(0.018,62.64)
ICvs.mCF	2.05(0.12,102.9)	--	0.9241(0.2923,2.862)	--	--	--	--
ICvs.mTCF	4.45(0.26,213.9)	1.50(0.014,195.6)	0.39(0.12,1.21)	--	55.87(1.88,2292)	0.22(0.016,2.20)	0.57(0.0011,187.9)
ICvs.mTF	3.64(0.21,188.9)	0.96(0.0082,120.5)	0.79(0.23,2.56)	--	0.90(0.047,18.41)	0.19(0.0086,3.55)	0.14(0.0014,10.72)
ICvs.mTOF	1.68(0.090,82.67)	3.97(0.039,548)	--	--	1.58(0.079,33.58)	0.21(0.0036,6.02)	1.52(0.018,119.4)
IFvs.OF	0.92(0.24,3.64)	--	1.28(0.35,4.51)	--	0.39(0.030,4.72)	6.34(0.65,61.32)	0.27(0.0026,34.28)
IFvs.RT	0.93(0.22,3.66)	--	0.64(0.18,2.07)	--	1.31(0.070,25.1)	9.81(0.34,758.1)	0.72(0.011,80.2)
IFvs.T	0.56(0.14,2.12)	--	0.78(0.43,1.40)	--	0.44(0.029,6.72)	10.16(0.39,762.6)	0.31(0.0062,25.46)
IFvs.TC	0.56(0.15,2.13)	--	1.26(0.78,2.07)	--	0.60(0.042,7.24)	6.49(0.69,58.91)	0.26(0.0047,19.14)
IFvs.TCF	0.93(0.26,3.46)	--	1.24(0.75,2.04)	--	0.79(0.067,8.51)	7.73(0.92,64.78)	0.24(0.0030,21.12)
IFvs.TF	1.53(0.52,4.52)	--	1.81(1.04,3.06)	--	0.74(0.086,6.26)	4.65(0.96,28.41)	0.29(0.014,5.45)
IFvs.TO	0.38(0.11,1.36)	--	1.76(1.06,2.83)	--	0.99(0.079,11.05)	2.80(0.34,25.12)	0.38(0.0097,17.77)
IFvs.TOF	1.10(0.32,3.91)	--	1.09(0.45,2.67)	-	0.99(0.078,11.59)	**19.34(2.70,157.5)**	0.46(0.011,22.52)
IFvs.mCF	0.54(0.12,2.49)	-	1.36(0.65,2.81)	--	--	--	--
IFvs.mTCF	1.14(0.27,4.81)	--	0.57(0.24,1.33)	--	**31.71(1.06,1145)**	4.13(0.41,42.56)	0.22(0.00036,129.9)
IFvs.mTF	0.95(0.24,3.69)	--	1.16(0.50,2.56)	--	0.51(0.032,7.82)	3.43(0.24,51.27)	0.058(0.00075,3.67)
IFvs.mTOF	0.43(0.10,1.86)	--	--	--	0.91(0.046,16.28)	4.18(0.084,86.25)	0.60(0.0088,51.32)
OFvs.RT	1.02(0.31,2.90)	0.84(0.020,22.01)	**0.49(0.33,0.75)**	7.80(0.52,105.2)	3.38(0.35,37.26)	1.53(0.078,70.99)	2.66(0.041,238)
OFvs.T	0.62(0.20,1.65)	0.33(0.011,6.39)	0.60(0.17,2.21)	1.45(0.12,15.75)	1.13(0.15,9.31)	1.61(0.090,68.28)	1.13(0.023,76.24)
OFvs.TC	0.60(0.28,1.31)	0.30(0.013,4.92)	0.98(0.29,3.47)	2.03(0.26,14.07)	1.53(0.28,8.01)	1.03(0.30,3.49)	0.96(0.018,55.76)

OFvs.TCF	1.02(0.54,1.89)	0.36(0.015,7.32)	0.97(0.29,3.33)	1.81(0.27,11.18)	2.02(0.51,8.05)	1.23(0.45,3.44)	0.89(0.0095,73)
OFvs.TF	1.68(0.73,3.65)	0.45(0.028,5.92)	1.42(0.43,4.76)	2.15(0.095,42.92)	1.91(0.52,7.57)	0.74(0.17,3.74)	1.07(0.023,37.91)
OFvs.TO	0.41(0.19,0.93)	0.35(0.027,3.78)	1.38(0.39,4.83)	5.24(0.74,34.14)	2.53(0.59,10.84)	0.440.12,1.69)	1.40(0.047,43.38)
OFvs.TOF	1.200.58,2.45)	1.33(0.18,10.17)	0.84(0.20,3.77)	4.95(0.91,26.33)	2.52(0.76,8.63)	3.06(0.78,14.03)	1.67(0.097,28.65)
OFvs.mCF	0.59(0.20,1.66)	--	1.05(0.28,4.23)	--	--	--	--
OFvs.mTCF	1.26(0.50,2.97)	0.51(0.016,15)	0.44(0.11,1.81)	0.33(0.030,3.19)	**79.97(5.26,1821)**	0.65(0.16,2.69)	0.80(0.00121,439.3)
OFvs.mTF	1.05(0.35,2.86)	0.33(0.011,7.53)	0.91(0.22,3.72)	9.18(0.20,405.9)	1.31(0.17,11.13)	0.54(0.052,5.14)	0.21(0.0019,17.23)
OFvs.mTOF	0.4614(0.1652,1.358)	1.318(0.08731,22.41)		100.2(7.098,1751)	2.326(0.2936,18.51)	0.662(0.01574,9.238)	2.188(0.05023,100.1)
RTvs.T	**0.61(0.41,0.91)**	0.39(0.094,1.62)	1.22(0.37,4.15)	0.19(0.063,0.53)	0.34(0.11,1.01)	1.06(0.52,2.12)	0.42(0.090,2.00)
RTvs.TC	0.60(0.22,1.85)	0.36(0.034,4.15)	1.98(0.63,6.64)	0.26(0.034,1.93)	0.45(0.048,3.72)	0.69(0.014,12.36)	0.35(0.019,5.63)
RTvs.TCF	1.001(0.38,3.08)	0.44(0.024,9.48)	1.95(0.62,6.37)	0.23(0.029,1.91)	0.60(0.067,4.85)	0.83(0.017,13.76)	0.33(0.00704,10.04)
RTvs.TF	1.66(0.67,4.27)	0.53(0.035,10.85)	2.85(0.93,9.27)	0.28(0.032,2.26)	0.57(0.0743,4.15)	0.50(0.0093,9.82)	0.40(0.011,7.74)
RTvs.TO	0.41(0.17,1.15)	0.42(0.038,5.85)	2.77(0.84,9.24)	0.67(0.091,5.05)	0.75(0.077,6.55)	0.30(0.0074,4.27)	0.53(0.028,7.40)
RTvs.TOF	1.18(0.46,3.48)	1.59(0.11,34.9)	1.69(0.43,7.15)	0.64(0.052,8.27)	0.75(0.071,7.11)	2.07(0.037,38.94)	0.64(0.020,13.78)
RTvs.mCF	0.58(0.16,2.33)	--	2.11(0.60,8.07)	--	--	--	--
RTvs.mTCF	1.22(0.39,4.45)	0.61(0.025,20.3)	0.89(0.23,3.46)	0.042(0.0034,0.51)	23.98(0.98,747.2)	0.43(0.0089,8.71)	0.30(0.00066,80.93)
RTvs.mTF	1.03(0.31,3.54)	0.39(0.016,12.03)	1.83(0.484,7.14)	1.17(0.053,27.09)	0.39(0.029,5.21)	0.35(0.0045,12.45)	0.077(0.0010,3.26)
RTvs.mTOF	0.46(0.15,1.725)	1.59(0.086,49.86)	--	13(0.65,325.5)	0.69(0.042,10.3)	0.40(0.0032,16.82)	0.83(0.017,27.89)
Tvs.TC	0.98(0.39,2.79)	0.91(0.13,6.78)	**1.62(1.08,2.33)**	1.40(0.25,7.90)	1.36(0.19,8.27)	0.64(0.015,10.65)	0.84(0.070,8.67)
Tvs.TCF	1.65(0.67,4.70)	1.12(0.085,17.24)	1.58(0.90,2.80)	1.25(0.21,7.90)	1.80(0.27,10.64)	0.78(0.017,12.01)	0.79(0.024,17.21)
Tvs.TF	2.72(1.19,6.33)	1.36(0.13,19.37)	**2.31(1.34,3.95)**	1.49(0.23,9.41)	1.70(0.31,8.94)	0.47(0.0095,8.26)	0.96(0.038,12.7)
Tvs.TO	0.67(0.30,1.70)	1.07(0.15,9.54)	**2.25(1.21,4.07)**	3.61(0.66,20.87)	2.24(0.30,14.64)	0.28(0.0075,3.68)	1.25(0.10,11.14)

Tvs.TOF	1.93(0.81,5.23)	4.11(0.42,60.67)	1.39(0.68,2.93)	3.43(0.36,36.01)	2.24(0.28,16.15)	1.96(0.037,33.56)	1.5(0.070,22.8)
Tvs.mCF	0.95(0.29,3.57)	--	1.73(0.90,3.35)	--	--	--	--
Tvs.mTCF	2.01(0.68,6.84)	1.58(0.087,38.37)	0.73(0.31,1.67)	0.23(0.024,2.23)	**71.42(3.58,1889)**	0.41(0.0089,7.51)	0.71(0.0019,159.2)
Tvs.mTF	1.693(0.5413,5.415)	1.005(0.05482,21.94)	1.48(0.62,3.43)	6.34(0.35,124)	1.16(0.11,12.15)	0.33(0.0046,10.78)	0.18(0.0034,5.80)
Tvs.mTOF	0.75(0.25,2.66)	4.10(0.30,89.65)	--	**69.65(4.26,1502)**	2.05(0.16,24.49)	0.37(0.0032,14.46)	1.97(0.059,47.13)
TCvs.TCF	1.69(0.98,2.89)	1.22(0.16,10.15)	0.98(0.60,1.60)	0.89(0.37,2.25)	1.33(0.41,4.46)	1.19(0.60,2.53)	0.94(0.068,9.89)
TCvs.TF	2.79(1.26,5.87)	1.49(0.19,13.32)	1.44(0.88,2.30)	1.06(0.085,13.1)	1.26(0.31,5.76)	0.73(0.18,3.37)	1.12(0.051,16.43)
TCvs.TO	0.68(0.33,1.46)	1.17(0.22,7.01)	1.40(0.81,2.34)	2.57(0.75,9.58)	1.65(0.42,6.93)	0.43(0.13,1.51)	1.48(0.14,13.88)
TCvs.TOF	1.98(0.90,4.38)	4.49(0.60,44.78)	0.86(0.40,1.91)	2.44(0.37,17.92)	1.65(0.33,8.94)	2.98(0.77,13.35)	1.78(0.093,29.03)
TCvs.mCF	0.97(0.42,2.21)	--	1.08(0.61,1.90)	--	--	--	--
TCvs.mTCF	2.08(0.92,4.52)	1.72(0.14,24.54)	**0.45(0.20,0.97)**	**0.16(0.030,0.87)**	**52.76(3.68,115)**	0.63(0.19,2.18)	0.86(0.0035,132.5)
TCvs.mTF	1.73(0.63,4.57)	1.11(0.084,15.09)	0.92(0.40,2.04)	4.51(0.16,137.6)	0.86(0.11,7.64)	0.53(0.058,4.49)	0.22(0.0057,5.67)
TCvs.mTOF	0.77(0.27,2.24)	4.49(0.41,74.03)	1.46(0.89,2.35)	**49.29(4.02,837.2)**	1.51(0.18,14.15)	0.64(0.015,8.58)	2.33(0.076,61.07)
TCFvs.TF	1.65(0.80,3.26)	1.22(0.18,8.47)	--	1.19(0.088,15.42)	0.94(0.32,3.03)	0.61(0.17,2.36)	1.19(0.042,29.78)
TCFvs.TO	**0.40(0.20,0.86)**	0.96(0.11,8.62)	1.42(0.86,2.28)	2.87(0.84,10.15)	1.25(0.38,4.16)	0.36(0.13,1.02)	1.60(0.075,37.23)
TCFvs.TOF	1.18(0.57,2.44)	3.67(0.38,43.4)	0.88(0.37,2.125)	2.73(0.44,18.26)	1.25(0.32,5.02)	2.48(0.74,9.45)	1.91(0.062,65.08)
TCFvs.mCF	0.58(0.23,1.38)	--	1.10(0.53,2.23)	--	--	--	--
TCFvs.mTCF	1.23(0.63,2.32)	1.40(0.33,7.05)	**0.46(0.21,0.98)**	**0.18(0.041,0.77)**	**39.38(3.426,699)**	0.53(0.20,1.39)	0.94(0.0070,90.64)
TCFvs.mTF	1.02(0.40,2.47)	0.90(0.11,7.44)	0.94(0.41,2.05)	5.04(0.17,161)	0.65(0.10,4.34)	0.44(0.051,3.5)	0.23(0.0088,5.46)
TCFvs.mTOF	0.45(0.17,1.29)	3.62(0.26,73.6)	--	**55.13(4.7,883.8)**	1.14(0.15,8.95)	0.54(0.013,6.47)	2.49(0.051,133.5)
TFvs.TO	**0.25(0.14,0.48)**	0.78(0.14,4.24)	0.98(0.61,1.52)	2.42(0.21,30.76)	1.32(0.36,4.44)	0.60(0.15,2.09)	1.33(0.16,14.73)
TFvs.TOF	0.71(0.38,1.42)	2.96(0.57,19.76)	0.60(0.25,1.44)	2.31(0.13,46.96)	1.32(0.35,4.81)	**4.18(1.29,12.63)**	1.58(0.17,19.18)

TFvs.mCF	0.35(0.12,1.04)	--	0.75(0.374,1.53)	--	--	--	--
TFvs.mTCF	0.75(0.30,1.92)	1.16(0.11,13.08)	**0.32(0.14,0.72)**	0.15(0.0085,2.84)	**41.55(3.137,819.4)**	0.87(0.17,4.141)	0.76(0.0025,214.7)
TFvs.mTF	0.62(0.27,1.44)	0.73(0.10,5.07)	0.64(0.31,1.30)	4.19(0.47,44.54)	0.69(0.12,3.84)	0.73(0.091,5.43)	0.20(0.0089,3.77)
TFvs.mTOF	**0.28(0.11,0.77)**	2.96(0.32,38.18)	--	47.1(1.65,1685)	1.21(0.16,8.93)	0.89(0.023,10.69)	2.07(0.10,55.09)
TOvs.TOF	**2.90(1.63,5.04)**	**3.80(1,17.87)**	0.62(0.25,1.55)	0.95(0.19,5.04)	1.00(0.30,3.41)	**6.87(2.15,25.18)**	1.19(0.17,8.06)
TOvs.mCF	1.43(0.47,4.07)	--	0.77(0.37,1.67)	--	--	--	--
TOvs.mTCF	**3.04(1.13,7.75)**	1.47(0.11,20.73)	**0.32(0.14,0.77)**	**0.063(0.010,0.37)**	**31.77(2.36,629.7)**	1.50(0.37,5.65)	0.57(0.0019,129.3)
TOvs.mTF	2.54(0.91,6.58)	0.94(0.078,10.39)	0.66(0.32,1.34)	1.75(0.061,52.67)	0.52(0.069,4.10)	1.23(0.13,11.29)	0.15(0.0043,3.84)
TOvs.mTOF	1.12(0.48,2.63)	3.79(0.61,32.52)	--	19.06(2.04,249.3)	0.91(0.15,5.60)	1.50(0.039,17.81)	1.55(0.13,19.43)
TOFvs.mCF	0.49(0.160,1.43)	--	1.25(0.50,3.17)	--	--	--	--
TOFvs.mTCF	1.05(0.39,2.67)	0.38(0.023,5.79)	0.53(0.17,1.54)	**0.067(0.0064,0.62)**	**31.66(2.09,705.4)**	**0.21(0.042,1.00)**	0.48(0.0013,135.7)
TOFvs.mTF	0.87(0.31,2.32)	0.25(0.017,2.77)	1.06(0.35,3.11)	1.84(0.044,75.15)	0.52(0.064,4.33)	0.18(0.017,1.58)	0.12(0.0029,3.99)
TOFvs.mTOF	**0.39(0.17,0.89)**	0.99(0.16,6.95)	--	**19.9(2.18,258)**	0.92(0.15,5.49)	0.22(0.0063,2.06)	1.30(0.11,17.03)
mCFvs.mTCF	2.13(0.77,5.93)	--	0.42(0.16,1.06)	--	--	--	--
mCFvs.mTF	1.77(0.52,6.09)	--	0.85(0.32,2.23)	--	--	--	--
mCFvs.mTOF	0.79(0.21,3.02)	--	--	--	--	--	--
mTCFvs.mTF	0.83(0.28,2.43)	0.64(0.048,7.42)	2.04(0.70,5.81)	27.82(0.71,1151)	**0.016(0.00058,0.34)**	0.84(0.082,8.19)	0.25(0.001036,68.77)
mTCFvs.mTOF	0.37(0.12,1.28)	2.59(0.12,68.13)	--	**305.8(18.62,6592)**	0.029(0.00093,0.63)	0.99(0.022,15.53)	2.75(0.0068,1270)
mTFvs.mTOF	0.45(0.13,1.60)	4.04(0.24,96.65)	--	11.19(0.19,740.1)	1.77(0.13,23.35)	1.17(0.021,28.99)	10.64(0.202,732.4)
