# Supplementary figures and images for: A Bayesian Network Meta-Analysis for Identifying the Optimal Taxane-Based Chemotherapy Regimens for Treating Gastric Cancer
Source: Front Pharmacol. 2019 Jul 5;10:717. doi: 10.3389/fphar.2019.00717 (PMC6624233; doi:10.3389/fphar.2019.00717)

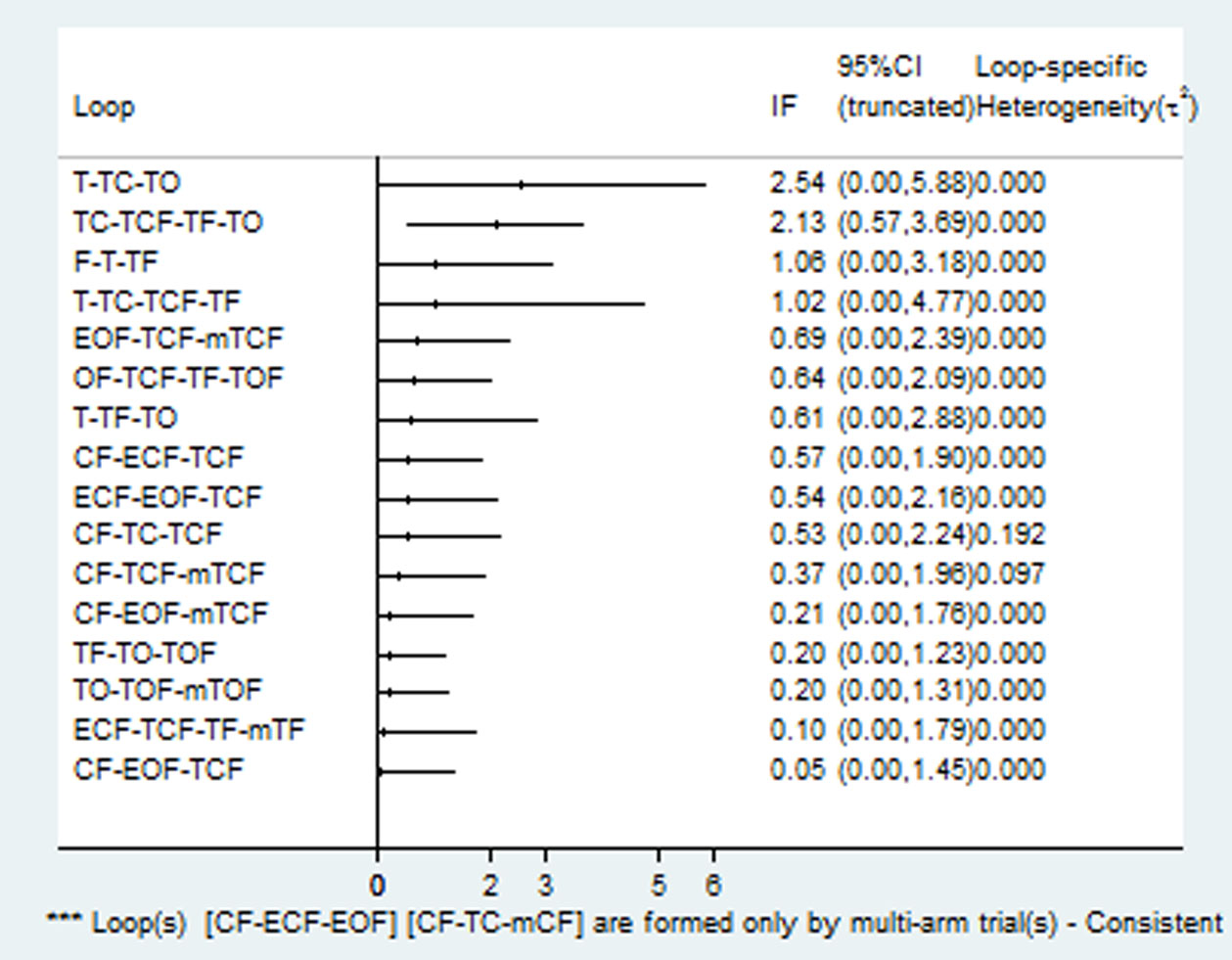

Supplement: Supplementary file 2 [file Image_1.jpeg]
